# Iridium-catalysed regioselective borylation of carboranes via direct B–H activation

**DOI:** 10.1038/ncomms14827

**Published:** 2017-03-16

**Authors:** Ruofei Cheng, Zaozao Qiu, Zuowei Xie

**Affiliations:** 1 Shanghai-Hong Kong Joint Laboratory in Chemical Synthesis, Shanghai Institute of Organic Chemistry, Chinese Academy of Sciences, 345 Lingling Road, Shanghai 200032, China; 2 Department of Chemistry and State Key Laboratory of Synthetic Chemistry, The Chinese University of Hong Kong, Shatin, N.T., Hong Kong, China

## Abstract

Carboranes are carbon–boron molecular clusters, which can be viewed as three-dimensional analogues to benzene. They are finding many applications in medicine, materials and organometallic chemistry. On the other hand, their exceptional thermal and chemical stabilities, as well as 3D structures, make them very difficult to be functionalized, in particular the regioselective functionalization of BH vertex among ten similar B–H bonds. Here we report a very efficient iridium-catalysed borylation of cage B(3,6)–H bonds of *o*-carboranes with excellent yields and regioselectivity using bis(pinacolato)diboron (B_2_pin_2_) as a reagent. Selective cage B(4)–H borylation has also been achieved by introducing a bulky TBDMS (*tert*-butyldimethylsilyl) group to one cage carbon vertex. The resultant 3,6-(Bpin)_2_-*o*-carboranes are useful synthons for the synthesis of a wide variety of B(3,6)-difunctionalized *o*-carboranes bearing cage B–*X* (*X*=O, N, C, I and Br) bonds.

## Abstract (web summary)

Functionalization of the multiple, similar, boron-hydrogen bonds in carboranes is difficult to achieve with selectivity. Here the authors report an iridium-catalysed borylation of *o*-carboranes with high yields and regioselectivities.

Icosahedral carboranes are carbon–boron molecular clusters, sharing many features with benzene such as aromaticity, high thermal and chemical stability^1^,^2^. On the other hand, carboranes have their own unique characteristics such as spherical geometry and hydrophobic molecular surface^1^,^2^, which make them attractive building blocks for boron neutron capture therapy agents in medicine^3^,^4^,^5^,^6^, functional units in supramolecular design/materials^7^,^8^,^9^,^10^,^11^,^12^,^13^,^14^,^15^,^16^,^17^ and versatile ligands in coordination/organometallic chemistry^18^,^19^,^20^,^21^,^22^,^23^. These research activities have drawn growing interests in the selective functionalization of carboranes^1^,^2^,^24^,^25^,^26^.

Classic routes to functionalized carboranes rely on the polarized cage C–H/B–H bonds: the weakly acidic C–H proton (pKa ∼23) and basic B–H hydride^1^. Accordingly, cage C–H bonds can be deprotonated by strong bases, followed by reactions with electrophiles to give carbon-substituted carboranes^1^,^2^, and cage B–H bonds are subjected to electrophilic substitution reactions, leading to the formation of cage boron-substituted carborane derivatives with the reaction rate B(9,12)–H>B(8,10)–H> B(4,5,7,11)–H (refs 1, 27). However, the B(3,6)-disubstituted *o*-carboranes cannot be prepared by electrophilic substitution reactions. They are generally achieved via multistep reaction of deboration–capping–deboration–capping (Fig. 1)^1^,^28^,^29^.

Very recently, we have developed –COOH guided transition metal-catalysed regioselective B(4)-alkenylation^30^, -alkynylation^31^, -amination^32^ and -hydroxylation^33^, as well as B(4,5)-dialkenylation^34^ and -diarylation^35^ of *o*-carboranes. In contrast, transition metal-catalysed B(3,6)-difunctionalization of *o*-carboranes is much less studied^36^,^37^, although transition metal promoted B(3)–H activation in *o*-carboranes has been well documented^38^,^39^,^40^,^41^,^42^,^43^,^44^.

Encouraged by transition metal-catalysed C–H borylation and application of the resultant boronate esters/boronic acids in C–C/C–O/C–N/C–halogen bond forming process^45^,^46^,^47^, we initiated a research program to study transition metal-catalysed direct cage B–H borylation of *o*-carboranes and the results are reported in this study (Fig. 1).

## Results

### B(3,6)-diborylation of *o*-carboranes

The optimization of reaction conditions for the following reactions was summarized in Supplementary Tables 1 and 2. The initial reaction of *o*-carborane (**1a**) with B_2_pin_2_ ([B(OCMe_2_CMe_2_O)]_2_) in the presence of 3.5 mol% [(cod)IrCl]_2_ (cod=1,5-cyclooctadiene) in tetrahydrofuran (THF) gave 3-Bpin-1,2-C_2_B_10_H_11_ (**2a**) in 60% gas chromatography (GC) yield. It was later found that the ligands played an important role in the reaction^48^,^49^. Addition of 0.21 equiv. of pyridine (Py) significantly increased the reaction efficiency, leading to the formation of **2a** and 3,6-(Bpin)_2_-1,2-C_2_B_10_H_10_ (**3a**) in 23% and 67% GC yields, respectively. Replacement of Py by 2-Me- and 4-Me-Py resulted in 98% and 95% GC yields of **3a**. Increasing the steric hindrance of Py derivatives led to much lower yields of **3a**. It was noted that bipyridine ligands commonly used in C–H borylation led to the formation of inseparable geometrical isomers of mono-, di- and tri-borylated products (see Supplementary Table 2). Other Ir(I) complexes such as [(cod)Ir(OMe)]_2_, (cod)Ir(acac), (cod)_2_IrBF_4_ and (cod)_2_IrB[3,5-(CF_3_)_2_C_6_H_3_]_4_ also worked well, giving very good to excellent yields of **3a**, whereas the Ir(III) complexes such as [Cp*IrCl_2_]_2_ and IrCl_3_, as well as [(cod)RhCl]_2_ and Pd(OAc)_2_ showed poor or no catalytic activity. On the other hand, HBpin did not give any borylation product. Extensive screening of solvents, catalyst loadings, reaction temperatures and molar ratios of ligand/B_2_pin_2_ led to the optimal reaction conditions shown entry 6 of Supplementary Table 1.

The substrate scope was then examined under the optimized reaction conditions and the results were compiled in Table 1. The borylation efficiency was generally very high regardless of the nature of substituents on cage B(9,12) of *o*-carboranes (**3a**–**3m**). It was noted that the double bond in 9-vinyl-*o*-carborane (**1k**) underwent hydroboration with HBpin, a byproduct of B–H borylation (vide infra), to afford **3k** in 91% isolated yield with excellent regioselectivity, probably owing to steric effect of *o*-carboranyl moiety. The B(3,6)-diborylation efficiency of 4-I-*o*-C_2_B_10_H_11_ (**1n**) was lower than other substrates likely to be due to steric effect of vicinal iodo group. In fact, both the mono- and diborylation products **2n** and **3n** were observed by GC–mass spectrometry with a molar ratio of 25:75. The bulkier substituents at the B(4) position such as 4-Ph and 4-(Ph)CH= (Ph)C or at B(4,7) positions such as 4,7-I_2_ can block the B(3)-borylation, giving **2o**, **2p** and **2q** in 76–89% isolated yields, respectively. For 3-Ph-*o*-C_2_B_10_H_11_, the expected monoborylation product **2r** was isolated in 89% yield. It was noteworthy that substituents on cage *C* had a significant impact on the borylation reaction. For example, 1-Me-*o*-C_2_B_10_H_11_ gave an inseparable mixture of geometrical isomers and no borylation with 1,2-Me_2_-*o*-C_2_B_10_H_10_ was observed.

We also examined the gram-scale borylation reaction. Under the optimal reaction conditions, treatment of **1a** (1.44 g, 10 mmol) with B_2_pin_2_ (10.16 g, 40 mmol) in THF (50 ml) afforded 3.75 g of **3a** (95% isolated yield).

In a similar manner, reaction of *m*-C_2_B_10_H_12_ (**4**) with 1.5 equiv. of B_2_pin_2_ in the presence of 3.5 mol% [(cod)IrCl]_2_ and 21 mol% 2-methylpyridine (2-MePy) in THF at 80 °C for 5 h gave 2-Bpin-*m*-carborane (**5**) in 74% isolated yield (see Supplementary Table 3 and Supplementary Fig. 6). On the other hand, under the same reaction conditions, *p*-carborane afforded an inseparable mixture of mono-, di- and triborylated products.

### B(4)-borylation of *o*-carboranes

**Figure 2:**
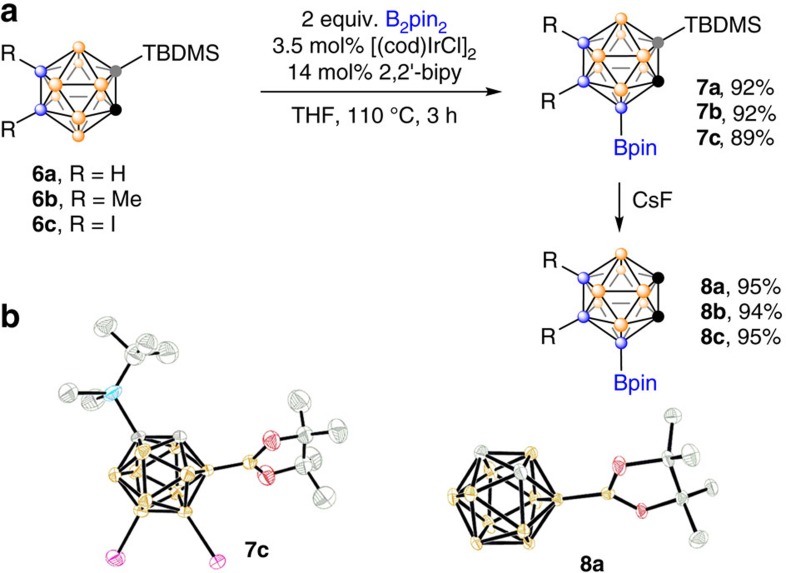
Synthesis of 4-Bpin-*o*-carboranes. (**a**) Ir-catalysed regioselective B(4)−H borylation in *o*-carboranes by introducing a bulky substituent at the cage *C* position. (**b**) Molecular structures of **7c** and **8a**.

The aforementioned results clearly show that bulky substituents such as Ph (**2o** in Table 1) and C(Ph)=CH(Ph) (**2p** in Table 1) can completely block the borylation of *ortho*-BH vertices, suggesting the importance of steric factors. We wondered whether Ir-catalysed regioselective cage B(4)–H borylation in *o*-carboranes could be achieved by introducing a bulky substituent at the cage *C* position. Accordingly, 1-trimethylsilyl-*o*-carborane was chosen as the model substrate for initial screening and the results were compiled in Supplementary Table 4. It was found that 2,2′-bipyridine (2,2′-bipy) derivatives were better ligands than monodentate Pys as the latters caused partial desilylation of 1-trimethylsilyl-*o*-carborane. The screening results indicated clearly that substrates with bulkier silyl groups can efficiently block the *ortho*-B–H activation, resulting in higher regioselectivity. If 2-TBDMS-*o*-carboranes **6** (TBDMS=*tert*-butyldimethylsilyl) were used as starting materials and 2,2′-bipy as the ligand, the desired product 4-Bpin-2-TBDMS-*o*-carboranes **7a**–**c** were isolated in 89–92% yields. Subsequently, the TBDMS group can be easily removed by caesium fluoride (CsF) under very mild condition to give the corresponding compound **8** in *ca.* 94% isolated yield (Fig. 2).

### Transformation of 3a

**Figure 3:**
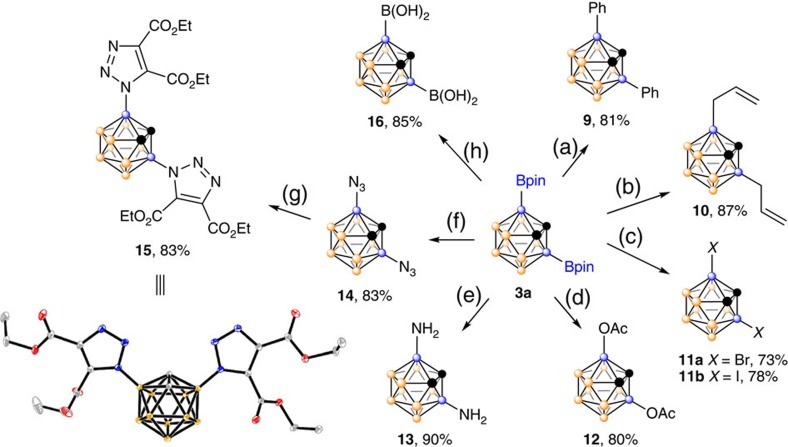
Chemical transformations of 3a. Reaction conditions: (a) PhBr (3 equiv.), Pd(PPh_3_)_4_ (20 mol%), Cs_2_CO_3_ (3 equiv.), cyclohexane, 150 °C (bath), 8 h. (b) Allyl chloride (6 equiv.), Pd(dba)_2_ (20 mol%), Cs_2_CO_3_ (3 equiv.), toluene, room temperature, 24 h. (c) PhX (3 equiv.), Pd(PPh_3_)_4_ (10 mol%), ^*t*^BuOK (3 equiv.), THF, 80 °C, 24 h. (d) Cu(OAc)_2_ (6 equiv.), KF (6 equiv.), CH_3_CN, 80 °C, 12 h, under 1 atm of O_2_. (e) MeONHLi, THF, 80 °C, 8 h. (f) TMSN_3_ (2.4 equiv.), CuCl (2.1 equiv.), KF (2.4 equiv.), THF, 60 °C, 24 h. (g) Diethyl acetylenedicarboxylate (2.4 equiv.), toluene, 95 °C. (h) 1) DEA (diethanolamine, 2.5 equiv.), Et_2_O, room temperature, 18 h, 2) HCl aq. (0.5 M, excess).

Although it has been well documented that Bpin can be replaced by a wide variety of functional groups^45^,^46^,^47^, the chemical properties of cage B–Bpin bonds have not been explored thus far. To illustrate the synthetic applications of B-borylated-*o*-carboranes, various transformations of **3** in an example of **3a** were studied and the results were outlined in Fig. 3. Suzuki–Miyaura cross-coupling of **3a** with PhBr in the presence of 20 mol% Pd(PPh_3_)_4_ and 3 equiv. of Cs_2_CO_3_ gave 3,6-diphenyl-*o*-carborane (**9**) in 81% isolated yield. Treatment of **3a** with CH_2_=CHCH_2_Cl in the presence of Pd(dba)_2_ (dba, dibenzylideneacetone) at room temperature afforded **10** in 87% yield. Surprisingly, replacement of Cs_2_CO_3_ by ^*t*^BuOK, reaction of **3a** with PhBr in the presence of Pd(PPh_3_)_4_ produced 3,6-Br_2_-*o*-C_2_B_10_H_10_ (**11a**) in 73% yield. Similarly, 3,6-I_2_-*o*-C_2_B_10_H_10_ (**11b**) was prepared in 78% isolated yield if PhI was used as coupling agent. It is not clear at this stage why ^*t*^BuOK can alter the coupling partner in these cross-coupling reactions. The two Bpin moieties in **3a** were readily replaced by acetoxy groups using Cu(OAc)_2_/KF in CH_3_CN under 1 atm of O_2_. 3,6-(NH_2_)_2_-*o*-C_2_B_10_H_10_ (**13**) was prepared in 90% isolated yield by treatment of **3a** with *in situ* generated MeONH^-^ in THF. Reaction of **3a** with TMSN_3_ in the presence of KF and CuCl gave 3,6-(N_3_)_2_-*o*-C_2_B_10_H_10_ (**14**) in 83% yield. Double click reaction of **14** with EtO_2_CC≡CCO_2_Et afforded 3,6-ditriazolyl-*o-*carborane (**15**) in 83% yield. In addition, carboranylboronic acid 3,6-[B(OH)_2_]_2_-*o*-C_2_B_10_H_10_ (**16**) was also synthesized from **3a** in 85% isolated yield.

Compounds **2**, **3**, **5** and **7**–**16** were fully characterized by ^1^H, ^13^C and ^11^B nuclear magnetic resonance (NMR) spectroscopy, as well as elemental analyses. The molecular structures of **2a**, **2p**, **2q**, **3a**, **3l**, **5**, **7c**, **8a** and **15** were further confirmed by single-crystal X-ray analyses.

### Mechanistic study

**Figure 4:**
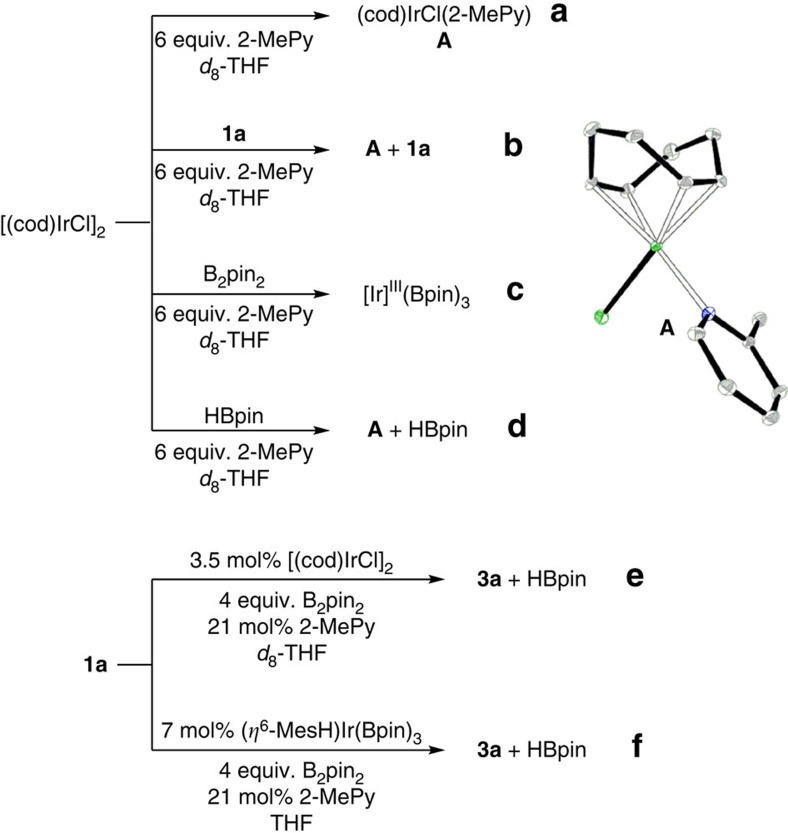
Mechanistic investigations. Control experiments. (**a**) Stoichiometric reaction of [(cod)IrCl]_2_ with 2-MePy. (**b**) Stoichiometric reaction of [(cod)IrCl]_2_ with 2-MePy and *o*-carborane (**1a**). (**c**) Stoichiometric reaction of [(cod)IrCl]_2_ with 2-MePy and B_2_pin_2_. (**d**) Stoichiometric reaction of [(cod)IrCl]_2_ with 2-MePy and HBpin. (**e**) Standard catalytic borylation reaction of **1a** monitored by ^1^H and ^11^B NMR. (**f**) (*η*^6^-MesH)Ir(Bpin)_3_ catalysed borylation reaction of **1a**.

To shed some light on the reaction mechanism of the first Ir-catalysed regioselective cage B–H borylation, NMR reactions (Fig. 4) were carried out in *d*_*8*_-THF, which were monitored by ^1^H and ^11^B NMR spectra (see Supplementary Figs 12–16 for detail). The following results were observed: (1) dissociation of [(cod)IrCl]_2_ in the presence of 2-MePy generated a monomeric species (cod)IrCl(2-MePy) (**A**) (Fig. 4, eq. a). (2) B_2_pin_2_ underwent rapid oxidative addition reaction on Ir(I) species in the presence of 2-MePy to generate a Ir(III) species^50^,^51^ and release ClBpin that was trapped by THF to form ROBpin (see Supplementary Fig. 14)^52^,^53^. However, no reaction was observed by treatment of [(cod)IrCl]_2_ with **1a** or HBpin under the same reaction conditions (Fig. 4, eq. b and d). (3) Under the optimal reaction conditions, both **2a** and HBpin were observed by ^1^H and ^11^B NMR at the initial stage. As the reaction proceeded, **3a** gradually appeared at the expense of **2a**. These results suggested that the borylation proceeded stepwise (Fig. 4, eq. e). (4) The Ir(III) complex (*η*^6^-MesH)Ir(Bpin)_3_ (ref. 54) was found to catalyse the diborylation of **1a** equally well as [(cod)IrCl]_2_ did to give **3a** in 98% GC yield (Fig. 4, eq. f). (5) B(3,6)–H bonds were more reactive than B(4,5,7,11)–H ones in the above borylation, suggesting that the activation of cage B–H bond may proceed via oxidative addition pathway^1^,^36^,^37^,^38^,^39^,^55^,^56^,^57^, instead of electrophilic substitution mechanism^30^,^31^,^32^,^33^,^34^,^35^ as the electron density in *o*-carborane follows the trend: B(3,6)<B(4,5,7,11)<B(8,10)<B(9,12)^1^,^27^.

**Figure 5:**
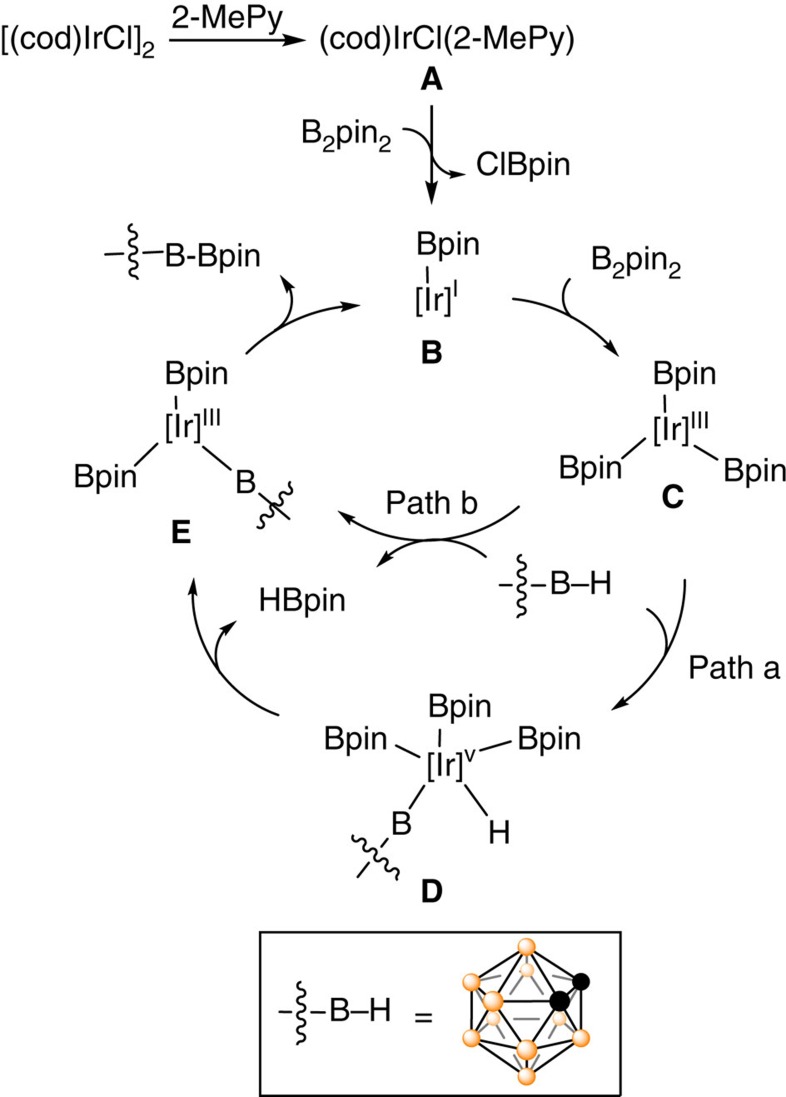
Proposed reaction mechanism. The ligand on iridium has been omitted for clarity.

On the basis of the aforementioned experimental results and literature work^30^,^36^,^45^,^46^,^47^,^48^,^49^,^50^,^51^,^52^,^53^,^54^,^55^,^56^,^57^, a proposed mechanism for the borylation reaction is shown in Fig. 5. Dissociation of [(cod)IrCl]_2_ in the presence of 2-MePy ligand generates a monomeric active species (cod)IrCl(2-MePy) (**A**), which undergoes oxidative addition with B_2_pin_2_, followed by reductive elimination to form Ir(I)-Bpin **B** and release ClBpin^50^,^58^,^59^, entering the catalytic cycle. Oxidative addition of B_2_pin_2_ on **B** gives the Ir(III) intermediate **C**^48^, followed by another oxidative addition of the most electron-deficient cage B(3)–H bond^1^,^36^,^37^ to afford **D** (path a). Reductive elimination yields the intermediate **E** and HBpin. Alternatively, electrophilic substitution of Ir(III) species in **C** on cage B–H yields the intermediate **E** and release one equivalent of HBpin (path b)^30^. Reductive elimination generates cage boron-borylated product **2** (ref. 48). Compound **2** undergoes another catalytic borylation cycle to afford cage B(3,6)-diborylated product **3**. As the electron-deficient cage B(3,6)–H bonds preferentially undergo oxidative addition reaction with transition metal species over other more electron-rich cage B–H bonds^1^,^36^,^37^, path a is believed to be more favourable over path b.

In summary, a very efficient and regioselective Ir-catalysed diborylation of cage B(3,6)–H bonds in carboranes has been developed. This serves as a new methodology for the regioselective generation of a series of B(3,6)-diborylated- or B(3)-borylated-*o*-carboranes. Selective B(4)-borylation of cage B(4)–H bond has also been achieved by introducing a TBDMS group to the cage carbon position. The resultant *B*-borylated carboranes can be conveniently converted to a variety of functionalized carboranes bearing cage B–*X* (*X*=Br, I), B–O, B–C(*sp*^2^), B–C(*sp*^3^), B–NH_2_ and B–N_3_ bonds that otherwise cannot be prepared by other known methods. This work opens up a new way for efficient and regioselective functionalization of carboranes, which may be extended to other boron cluster systems.

## Methods

### Preparation of B(3,6)-diborylated- or B(3)-borylated-*o*-carboranes (3 or 2)

An oven-dried Schlenk flask was charged with *o*-carborane (**1**) (0.5 mmol), B_2_pin_2_ (508 mg, 2.0 mmol), [(cod)IrCl]_2_ (12 mg, 0.0175, mmol) and 2-MePy (10.3 mg, 0.105 mmol), followed by dry THF (5 ml). The flask was closed under an atmosphere of nitrogen and stirred at 110 °C (bath temperature) for 5 h. After hydrolysis with water (10 ml) and extraction with diethyl ether (10 ml × 3), the ether solutions were combined and concentrated to dryness *in vacuo*. The residue was subjected to flash column chromatography on silica gel (230–400 mesh) using *n*-hexane and ethyl acetate (10/1 in v/v) as eluent to give a mixture of product and B_2_pin_2_. Removal of B_2_pin_2_ via sublimation at 90 °C under vacuum (0.1 torr) gave a pure product **2o**–**r** or **3a**–**n**.

### B(4)-borylated-carboranes (**8**)

Compound **7** was prepared from 1-TBDMS-*o*-carboranes **6** (0.5 mmol), B_2_pin_2_ (254 mg, 1.0 mmol), [(cod)IrCl]_2_ (12 mg, 0.0175, mmol) and 2,2′-bipy (22 mg, 0.07 mmol) in THF (5 ml) at 110 °C (bath temperature) for 3 h, using the same procedure reported for **3**. To a solution (2 ml) of **7** (0.3 mmol) (acetone for **7a** and **7b**; MeOH/DCM (2/1 in v/v) for **7c**) was added CsF (182 mg, 1.2 mmol). The mixture was stirred at room temperature (for 1 h for **7a** and **7b**, and 20 min for **7c**). After filtration and removal of the solvent under vacuo, the residue was subjected to flash column chromatography on silica gel (230–400 mesh) using *n*-hexane/Et_3_N (5/1 in v/v) as eluent to give product **8**.

For NMR spectra and single-crystal X-ray structures of the compounds in this study, see Supplementary Figs 1–5,7–11 and 17–178.

### Data availability

X-ray crystallographic data for compounds **2a**, **2p**, **2q**, **3a**, **3l**, **5**, **7c**, **8a** and **15**, and complex **A** have been deposited at the Cambridge Crystallographic Data Centre as CCDC 1500326–1500335, respectively (http://www.ccdc.cam.ac.uk/pages/Home.aspx). The authors declare that the data supporting the findings of this study are available within the article (and Supplementary Information files) and also are available from the corresponding author on request.

## Additional information

**How to cite this article:** Cheng, R. *et al*. Iridium-catalysed regioselective borylation of carboranes via direct B–H activation. *Nat. Commun.* **8,** 14827 doi: 10.1038/ncomms14827 (2017).

**Publisher's note:** Springer Nature remains neutral with regard to jurisdictional claims in published maps and institutional affiliations.

## Supplementary Material

Supplementary Information Supplementary figures, supplementary tables, supplementary discussion, supplementary methods, and supplementary references.

## Figures and Tables

**Figure 1:**
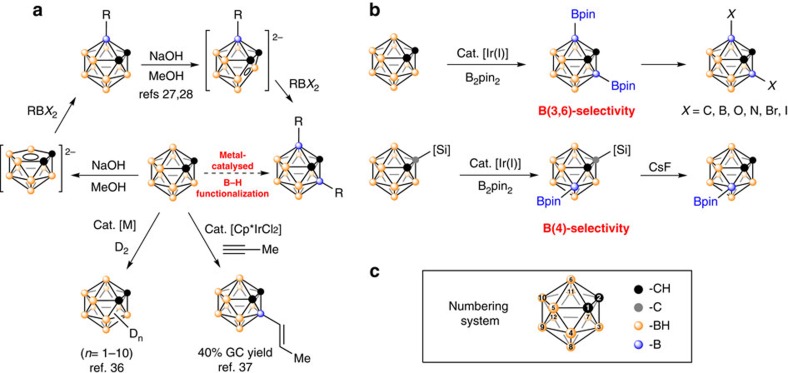
Functionalization of B(3,6)-H bonds in *o*-carboranes (Bpin=B(OCMe_2_CMe_2_O), B_2_pin_2_=pinB-Bpin). (**a**) Known methods for B(3) and B(3,6) functionalization. (**b**) This work: Iridium-catalysed regioselective borylation of carboranes via direct B−H activation. (**c**) Numbering system of *o*-carborane.

**Table 1:**
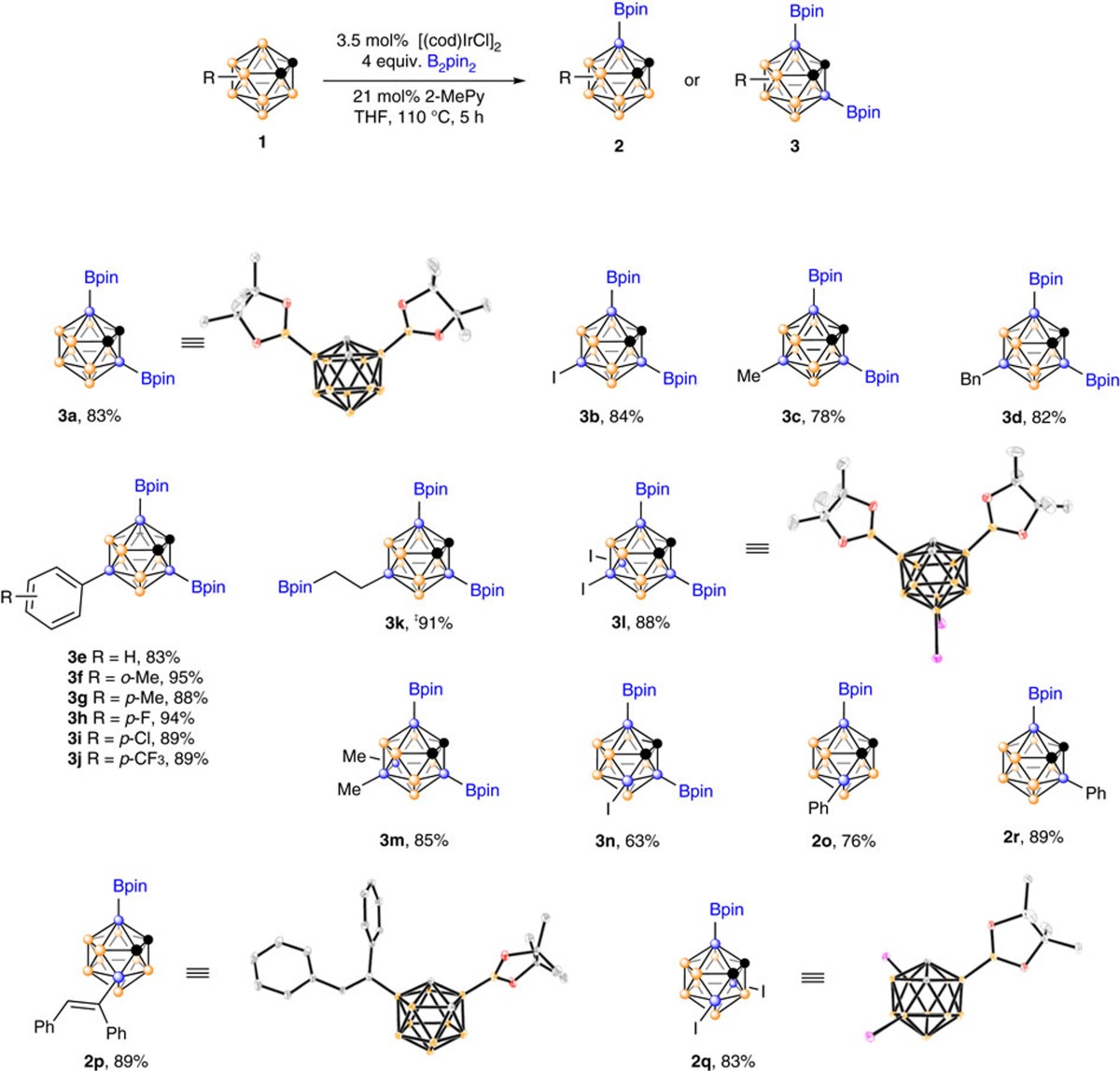
Substrate scope for selective cage B–H borylation of *o*-carboranes* †.

^*^Reactions were conducted on 0.5 mmol scale in a closed flask at 110 °C (bath temperature) for 5 h.

^†^Isolated yields.

^‡^9-Vinyl-*o*-carborane (**1k**) was used as a starting material.
