# Supplementary material for: Iridium-catalysed regioselective borylation of carboranes via direct B–H activation
Source: Nat Commun. 2017 Mar 16;8:14827. doi: 10.1038/ncomms14827 (PMC5357313; doi:10.1038/ncomms14827)
Supplement: Supplementary Information — Supplementary figures, supplementary tables, supplementary discussion, supplementary methods, and supplementary references. [file ncomms14827-s1.pdf]

Supplementary Information

Supplementary Methods

General Procedures. All reactions were carried out in oven-dried glassware under an atmosphere of dry N_2 with the rigid exclusion of air and moisture using standard Schlenk techniques or in a glovebox. Organic solvents were freshly distilled from sodium benzophenone ketyl immediately prior to use. Compounds **1b**,[1] **1c**,[2,3] **1e**,[2,3,4] **1g**,[4] **1i**,[4] **1j**,[4] **1k**,[5] **1l**,[2] **1m,**[2,3] **1n**,[6] **1o**,[2,3] **1p**,[7] **1q**,[6] **1r**,[8] **6a**,[9] and (η^6-MesH)Ir(Bpin)$_3$[10] were prepared according to literature procedures. All other chemicals were purchased from either Aldrich or J&K Chemical Co. and used as received unless otherwise specified. ^{1}H and ^{19}F NMR spectra were recorded on a Varian Inova 300 spectrometer at 300 MHz and 282 MHz, respectively. ^{13}C{^{1}H} NMR spectra were recorded on either a Varian Inova 300 spectrometer at 75 Hz or a Bruker 400 spectrometer at 100 Hz. ^{11}B{^{1}H} NMR spectra were recorded on a Bruker 400 spectrometer at 128 MHz. All signals were reported in ppm unit with references to the residual solvent resonances of the deuterated solvents for proton and carbon chemical shifts, to external BF_3 OEt_2 (0.00) for boron chemical shifts and to external $CFCl_3$ (0.00) for fluorine chemical shifts. Mass spectra were obtained on a Thermo Finnigan MAT 95 XL spectrometer. Elemental analyses were performed with an elementary VARIO EL III elemental analyzer, Shanghai Institute of Organic Chemistry, CAS.

Preparation of Starting Materials.

(1) Preparation of 1d, 1f, 1h. A representative procedure.

To a THF (15 mL) solution of 9-iodo-*o*-carborane (2.700 g, 10.0 mmol) and [$(PPh_3)_2PdCl_2$] (280 mg,

0.4 mmol) was added RMgBr (1.0 M in THF, 50.0 mL, 50.0 mmol) and CuI (76 mg, 0.4 mmol), respectively, under an atmosphere of dry nitrogen at 0 ^{o}C. The resulting mixture was stirred at 45 ^{o}C till the completion of the reaction as monitored by GC-MS. The reaction was slowly quenched with water at 0 ^{o}C and extracted with diethyl ether (20 mL x 3). The ether solutions were combined and concentrated to dryness in vacuo. The residue was subjected to flash column chromatography on silica gel (230-400 mesh) using *n*-hexane as eluent to give product **1**.

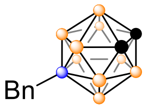

1d

1d: Yield: 81%. White solid. ^{1}H NMR ($CDCl_3$, 300 MHz): δ 7.21 (m, 2H), 7.04 (m, 3H) (aromatic C*H*), 3.44 (s, 1H), 3.39 (s, 1H) (cage C*H*), 3.21 (s, 2H) (B_{cage}–CH_2); ^{13}C{^{1}H} NMR ($CDCl_3$, 75 MHz): δ 144.7, 128.5, 128.0, 124.1 (aromatic *C*), 53.3, 48.5 (cage *C*H), the B_{cage}–*C*H_2 was not observed; ^{11}B NMR ($CDCl_3$, 128 MHz): δ 8.4 (s, 1B) (B_{cage}–CH_2), -1.9 (d, J_{BH} = 148 Hz, 2B) , -8.7 (d, J_{BH} = 149 Hz, 2B), -13.7 (d, J_{BH} = 76 Hz, 2B), -14.3 (m, 2B), -15.4 (d, J_{BH} = 163 Hz, 2B) (B_{cage}H); analysis (calcd., found for $C_9H_{18}B_{10}$): C (46.13, 46.37), H (7.74, 7.69).

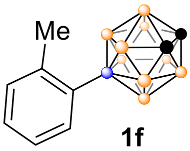

1f

1f: Yield: 70%. White solid. ^{1}H NMR ($CDCl_3$, 300 MHz): δ 7.50 (m, 1H), 7.08 (m, 3H) (aromatic C*H*), 3.58 (s, 1H), 3.55 (s, 1H) (cage C*H*), 2.49 (s, 3H) (CH_3). ^{13}C{^{1}H} NMR ($CDCl_3$, 75 MHz): δ 141.7, 136.0, 130.4, 127.5, 125.0 (aromatic *C*), 53.1, 50.5 (cage *C*H), 24.1 (*C*H_3), the B_{cage}–*C* was not observed. ^{11}B NMR ($CDCl_3$, 128 MHz): δ 7.7 (s, 1B) (B_{cage}–C), -1.7 (d, J_{BH} = 149 Hz, 2B) , -8.4 (d, J_{BH} = 149 Hz, 2B), -13.5 (d, J_{BH} = 91 Hz, 2B), -14.2 (d, J_{BH} = 75 Hz, 2B), -15.4 (d, J_{BH} = 163 Hz, 2B) (B_{cage}H); analysis (calcd., found for $C_9H_{18}B_{10}$): C (46.13, 46.25), H (7.74, 7.46).

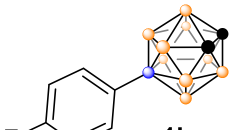

1h

1h: White solid, Yield: 83%. ^{1}H NMR ($CDCl_3$, 300 MHz): δ 7.33 (m, 2H), 6.92 (m, 2H) (aromatic C*H*), 3.64 (s, 1H), 3.53 (s, 1H) (cage C*H*). ^{13}C{^{1}H} NMR ($CDCl_3$, 75 MHz): δ 162.9 (d, $^{1}J_{CF}$ = 244 Hz), 134.1 (d, $^{3}J_{CF}$ = 7.4 Hz), 114.4 (d, $^{2}J_{CF}$ = 19.7 Hz) (aromatic *C*) 53.3, 50.0 (cage *C*H), the B_{cage}–*C* was not observed. ^{11}B NMR ($CDCl_3$, 128 MHz): δ 7.6 (s, 1B) (B_{cage}–C), -1.9 (d, J_{BH} = 149 Hz, 2B) , -8.4 (d, J_{BH} = 149 Hz, 2B), -13.5 (d, J_{BH} = 52 Hz, 2B), -14.0 (m, 2B), -15.1 (d, J_{BH} = 174 Hz, 2B) (B_{cage}H); analysis (calcd., found for $C_8H_{15}FB_{10}$): C (40.32, 40.16), H (6.34, 6.20).

(2) Preparation of 1-Silyl-*o*-carboranes (6b, 6c). A Representative Procedure.

To a diethyl ether solution (8 mL) of *o*-carborane (2.0 mmol) was added ^{n}BuLi (1.6 M in ether, 1.3 mL) under an atmosphere of dry nitrogen at 0 °C. After stirring for 2 h at 0 °C, TBDMSCl (452 mg, 3.0 mmol) was added at 0 °C and the resulting mixture was stirred overnight at room temperature. After hydrolysis with water (10 mL) and extraction with diethyl ether (10 mL x 3), the ether solutions were combined and concentrated to dryness in vacuo. The residue was subjected to flash column chromatography on silica gel (230-400 mesh) using *n*-hexane as eluent to give the product **6**.

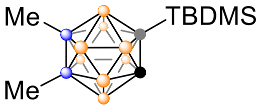

6b

6b: Yield 81%. White solid. ^{1}H NMR ($CDCl_3$, 300 MHz): δ 3.24 (s, 1H) (cage C*H*), 1.01 (s, 9H) (C(CH_3)$_3$), 0.21 (s, 6H) (SiCH_3), 0.18 (s, 6H) (B_{cage}–CH_3). ^{13}C{^{1}H} NMR ($CDCl_3$, 75 MHz): δ 57.1 (cage *C*), 53.6 (cage *C*H), 27.2 (C(*C*H$_3$)$_3$), 19.5 (*C*(CH$_3$)$_3$), -4.3 (Si*C*H$_3$), the two B_{cage}–*C*H$_3$ were not observed. ^{11}B NMR ($CDCl_3$, 128 MHz): δ 9.9 (s, 1B), 7.9 (s, 1B) (B_{cage}–CH$_3$), -5.2 (d, J_{BH} = 145 Hz, 2B) (B_{cage}H), -10.7 (d, J_{BH} = 177 Hz, 2B) (B_{cage}H), -12.4 (d, J_{BH} = 195 Hz, 2B) (B_{cage}H), -14.7 (d, J_{BH} = 172 Hz, 2B)

(B_{cage}H); analysis (calcd., found for $C_{10}H_{30}B_{10}Si$): C (41.92, 42.32), H (10.55, 10.56).

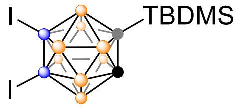

6c

6c: Yield 81%. White solid. ^{1}H NMR ($CDCl_3$, 300 MHz): δ 3.83 (s, 1H) (cage C*H*), 1.03 (s, 9H) (C(CH_3)$_3$), 0.25 (s, 6H) (SiCH_3). ^{13}C{^{1}H} NMR ($CDCl_3$, 75 MHz): δ 62.2 (cage *C*), 57.3 (cage *CH*), 27.0 (C(*C*H$_3$)$_3$), 19.4 (*C*(CH$_3$)$_3$), -4.3 (Si*C*H$_3$). ^{11}B NMR ($CDCl_3$, 128 MHz): δ -2.7 (d, J_{BH} = 156 Hz, 2B), -9.0 (m, 2B), -10.6 (m, 2B), -11.6 (m, 2B) (B_{cage}H), -12,4 (s, 2B) (B_{cage}–I); analysis (calcd., found for $C_8H_{24}B_{10}I_2Si$): C (18.83, 19.07), H (4.74, 4.74).

B(3,6)- or B(3)-borylation of *o*-carboranes.

Supplementary Table 1. Optimization of reaction conditions.*

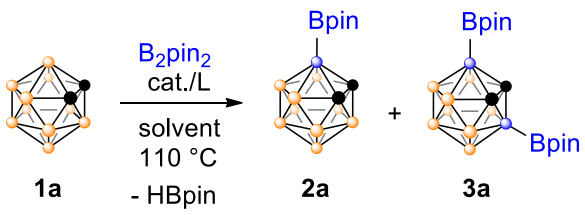

entry	cat. (mol%)	L (mol%)	B_2pin_2 (eq.)	solvent	yield (%)†		
					**1a**	**2a**	**3a**
1	$[(cod)IrCl]_2$ (3.5)	-	4	THF	40	60	-
2	$[(cod)IrCl]_2$ (3.5)	Py (21)	4	THF	10	23	67
3	$[(cod)IrCl]_2$ (5.0)	2-MePy (20)	4	THF	-	10	90
4	$[(cod)IrCl]_2$ (3.5)	2-MePy (14)	4	THF	-	10	90
5	$[(cod)IrCl]_2$ (2.5)	2-MePy (10)	4	THF	-	20	80
6	$[(cod)IrCl]_2$ (3.5)	2-MePy (21)	4	THF	-	2	98
7	$[(cod)IrCl]_2$ (3.5)	2-MePy (21)	2	THF	3	35	62
8	$[(cod)IrCl]_2$ (3.5)	2-MePy (21)	1	THF	12	65	23
9‡	$[(cod)IrCl]_2$ (3.5)	2-MePy (21)	4	THF	-	9	91
10	$[(cod)IrCl]_2$ (3.5)	2-MePy (32)	4	THF	-	2	98
11	$[(cod)IrCl]_2$ (3.5)	2-MePy (21)	4	toluene	63	37	-
12	$[(cod)IrCl]_2$ (3.5)	2-MePy (21)	4	DME	9	58	33
13	$[(cod)IrCl]_2$ (3.5)	2-MePy (21)	4	1,4-dioxane	-	33	67
14	$[(cod)IrCl]_2$ (3.5)	4-MePy (21)	4	THF	-	5	95
15	$[(cod)IrCl]_2$ (3.5)	4-^{t}BuPy (21)	4	THF	6	54	40
16	$[(cod)IrCl]_2$ (3.5)	2,6-Me_2Py (21)	4	THF	18	68	14
17	$[(cod)IrCl]_2$ (3.5)	2,4,6-Me_3Py (21)	4	THF	32	61	7
18	$[(cod)IrCl]_2$ (3.5)	2-PhPy (21)	4	THF	8	63	29
19	$[(cod)IrCl]_2$ (3.5)	4-CF_3Py (21)	4	THF	81	9	-
20	$[(cod)IrCl]_2$ (3.5)	2,6-F_2Py (21)	4	THF	71	29	-
21	$[(cod)Ir(OMe)]_2$ (3.5)	2-MePy (21)	4	THF	-	14	86
22	(cod)Ir(acac) (7.0)	2-MePy (21)	4	THF	-	3	97
23	$(cod)_2IrBF_4$ (7.0)	2-MePy (21)	4	THF	-	2	98
24	$(cod)_2IrB_{ArF}$ (7.0)	2-MePy (21)	4	THF	-	14	86
25	$[Cp*IrCl_2]_2$ (3.5)	2-MePy (21)	4	THF	100	-	-
26	$IrCl_3$ (7.0)	2-MePy (21)	4	THF	100	-	-
27	$[(cod)RhCl]_2$ (3.5)	2-MePy (21)	4	THF	60	40	-
28	$Pd(OAc)_2$ (7.0)	2-MePy (21)	4	THF	100	-	-

*Reaction conditions: **1a** (0.2 mmol), B_2pin_2, [M] catalyst, L, 110 °C (bath), 5 h; B_2pin_2 = $[B(OCMe_2CMe_2O)]_2$, Bpin = $B(OCMe_2CMe_2O)$, cod = 1,5-cyclooctadiene, acac = acetylacetonato, B_{ArF} = tetrakis[3,5-bis(trifluoromethyl)phenyl]borate, Py = pyridine. †GC yield. ‡80 °C (bath).

Supplementary Table 2. Optimization of reaction conditions with bipyridine ligands.*

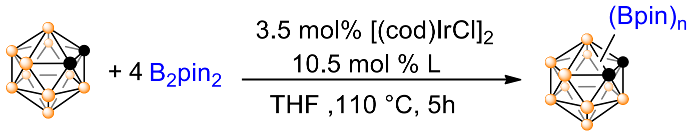

1a **CB-(Bpin)$_n$**

entry	L	yield (%)†			
		**1a**	**CB-(Bpin)$_n$**		
			n = 1	n = 2	n = 3
1	2,2'-bipy	2	3(**2a**) + 1	73(**3a**) + 5 + 4	8 + 4
2	4,4'-Me$_2$bipy	2	4(**2a**) + 2	62(**3a**) + 7 + 1	17 + 5
3	4,4'-^{t}Bu$_2$bipy	3	3(**2a**) + 3	27(**3a**) + 5 + 2	42 + 12 + 3
4	1,10-Phen	4	7(**2a**) + 4	57(**3a**) + 8 + 2	8 + 10
5	3,4,7,8-Me$_4$-1,10-Phen	9	8(**2a**) + 4	35(**3a**) + 11 + 2	18 + 13
6‡	Py	10	23(**2a**)	67(**3a**)	-
7‡	2-MePy	-	2(**2a**)	98(**3a**)	-

*Reaction condition: **1a** (0.1 mmol), 3.5 mol% [(cod)IrCl]$_2$, 10.5 mol% L. †The ratio and selectivity of borylation products were determined by GC-MS. Noted that there were several geometrical isomers of borylated species with the same MS using bipyridine ligands. ‡21 mol% L.

Preparation of B(3,6)-diborylated- or B(3)-borylated-*o*-carboranes (3 or 2). A Representative Procedure. An oven-dried Schlenk flask equipped with a stir bar was charged with *o*-carborane (**1**) (0.5 mmol), B$_2$pin$_2$ (508 mg, 2.0 mmol), [(cod)IrCl]$_2$ (11.8 mg, 0.0175 mmol), and 2-methylpyridine (9.8 mg, 0.105 mmol), followed by dry THF (5 mL). The flask was closed under an atmosphere of nitrogen and stirred at 110 ^{o}C (bath temperature) for 5 h. After hydrolysis with water (10 mL) and extraction with diethyl ether (10 mL x 3), the ether solutions were combined and concentrated to dryness in vacuo. The residue was subjected to flash column chromatography on silica gel (230-400 mesh) using *n*-hexane and ethyl acetate (10/1 in v/v) as eluent to give a mixture of product and B$_2$pin$_2$. Removal of B$_2$pin$_2$ via sublimation at 90 ^{o}C under vacuum (0.1 torr) afforded a pure product **2o-r** or **3a-n**.

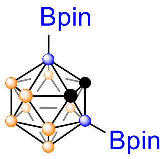

3a

3a: Yield 83%. White solid. ^{1}H NMR ($CDCl_3$, 300 MHz): δ 3.53 (s, 2H) (cage C*H*), 1.24 (s, 24H) (C*H*$_3$); ^{13}C{^{1}H} NMR ($CDCl_3$, 75 MHz): δ 84.2 (O*C*), 57.6 (cage *C*H), 24.9 (*C*H$_3$); ^{11}B NMR ($CDCl_3$, 128 MHz): δ 32.7 (s, 2B) (*B*–B$_{cage}$), -0.9 (d, J_{BH} = 151 Hz, 2B), -6.7 (d, J_{BH} = 156 Hz, 2B) (B_{cage}H), -11.8 (m, 6B) (B_{cage}H & B–B_{cage}); analysis (calcd., found for $C_{14}H_{34}B_{12}O_4$): C (42.45, 42.46), H (8.65, 8.71).

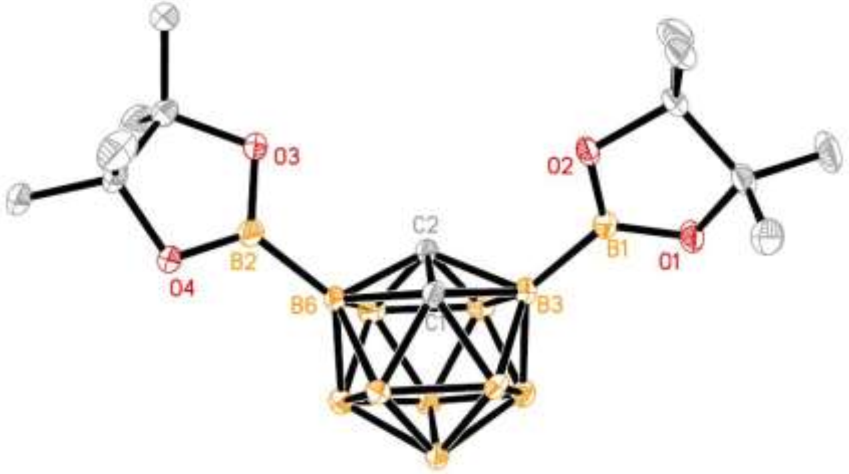

Supplementary Figure 1. Molecular structure of **3a** drawn with 30% probability ellipsoids.

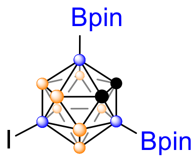

3b

3b: Yield 84%. White solid. ^{1}H NMR ($CDCl_3$, 300 MHz): δ 3.86 (s, 1H), 3.64 (s, 1H) (cage C*H*), 1.26 (s, 24H) (C*H*$_3$); ^{13}C{^{1}H} NMR ($CDCl_3$, 75 MHz): δ 84.6 (O*C*), 58.0, 53.8 (cage *C*H), 25.0 (*C*H$_3$); ^{11}B NMR ($CDCl_3$, 128 MHz): δ 32.4 (s, 2B) (*B*–B$_{cage}$), 0.8 (d, J_{BH} = 144 Hz, 1B), -4.9 (d, J_{BH} = 156 Hz, 2B), -11.2 (m, 5B) (B_{cage}H), -14.8 (s, 2B) (B_{cage}–I & B–B_{cage}); analysis (calcd., found for $C_{14}H_{33}B_{12}O_4I$): C (32.21, 32.48), H (6.37, 6.34).

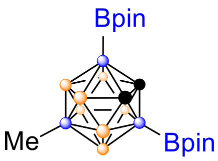

3c

3c: Yield 78%. White solid. ^{1}H NMR ($CDCl_3$, 300 MHz): δ 3.47 (s, 1H), 3.39 (s, 1H) (cage C*H*), 1.25 (s, 27H) (C*H*$_3$); ^{13}C{^{1}H} NMR ($CDCl_3$, 75 MHz): δ 84.2

(O*C*), 56.6, 50.4 (cage *C*H), 25.0 (C*C*H$_3$), the B$_{cage}$–*C*H$_3$ was not observed; ^{11}B NMR (CDCl$_3$, 128 MHz): δ 32.9 (s, 2B) (*B*–B$_{cage}$), 9.1 (s, 1B) (*B*$_{cage}$Me), 0.6 (d, J_{BH} = 42 Hz, 1B) (*B*$_{cage}$H), -5.5 (d, J_{BH} = 132 Hz, 2B) (*B*$_{cage}$H), -11.8 (m, 6B) (*B*$_{cage}$H & B–*B*$_{cage}$); analysis (calcd., found for $C_{15}H_{36}B_{12}O_4$): C (43.92, 43.93), H (8.85, 8.91).

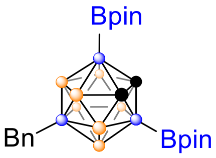

3d

3d: Yield 82%. White solid. ^{1}H NMR (CDCl$_3$, 300 MHz): δ 7.19 (m, 2H), 7.04 (m, 3H) (aromatic C*H*), 3.47 (s, 1H), 3.45 (s, 1H) (cage C*H*), 2.22 (s, 2H) (C*H*$_2$), 1.29 (s, 24H) (C*H*$_3$); ^{13}C{^{1}H} NMR (CDCl$_3$, 75 MHz): δ 144.8, 128.5, 127.8, 123.8 (aromatic *C*), 84.1 (O*C*), 56.4, 51.7 (cage *C*H), 24.9(*C*H$_3$), the B$_{cage}$–*C*H$_2$ was not observed; ^{11}B{^{1}H} NMR (CDCl$_3$, 128 MHz): δ 33.1 (s, 2B) (*B*–B$_{cage}$), 10.0 (s, 1B) (*B*$_{cage}$–Bn), -0.2 (d, J_{BH} = 61 Hz, 1B) (*B*H), -5.9 (d, J_{BH} = 113 Hz, 2B) (*B*$_{cage}$H), -11.9 (m, 6B) (*B*$_{cage}$H & B–*B*$_{cage}$); analysis (calcd., found for $C_{21}H_{40}B_{12}O_4$): C (51.87, 51.50), H (8.29, 8.34).

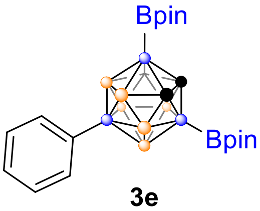

3e

3e: Yield 83%. White solid. 1H NMR (CDCl$_3$, 300 MHz): δ 7.40 (m, 2H) 7.20 (m, 3H) (aromatic C*H*), 3.66 (s, 1H), 3.57 (s, 1H) (cage C*H*), 1.29 (s, 24H) (C*H*$_3$); ^{13}C{^{1}H} NMR (CDCl$_3$, 75 MHz): δ 132.5, 127.3, 127.0 (aromatic *C*), 84.3 (O*C*), 56.4, 52.1 (cage *C*H), 25.0 (*C*H$_3$), the B$_{cage}$–*C* was not observed; ^{11}B NMR (CDCl$_3$, 128 MHz): δ 33.1 (s, 2B) (*B*–B$_{cage}$), 9.4 (s, 1B) (*B*$_{cage}$–Ph), 0.2 (d, J_{BH} = 50 Hz, 1B) (*B*$_{cage}$H), -6.1 (d, J_{BH} = 148 Hz, 2B) (*B*$_{cage}$H), -11.9 (m, 6B) (*B*$_{cage}$H & B–*B*$_{cage}$); analysis (calcd., found for $C_{20}H_{38}B_{12}O_4$): C (50.87, 51.22), H (8.11, 8.13).

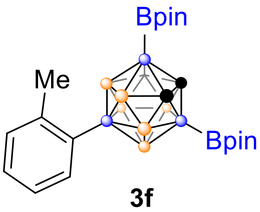

3f

3f: Yield 95%. White solid. ^{1}H NMR (CDCl$_3$, 300 MHz): δ 7.52 (m, 1H), 7.03

(m, 3H) (aromatic C*H*), 3.63 (s, 1H), 3.59 (s, 1H) (cage C*H*), 2.48 (s, 3H) (C_6H_4–C*H*$_3$), 1.28 (s, 24H) (OC–C*H*$_3$); ^{13}C{^{1}H} NMR ($CDCl_3$, 75 MHz): δ 141.6, 136.0, 130.1, 127.2, 124.7 (aromatic *C*), 84.3 (O*C*), 56.1, 53.6 (cage *C*H), 25.0 (OC–*C*H$_3$), 24.0 (C_6H_4–*C*H$_3$), the B_{cage}–*C* was not observed; ^{11}B NMR ($CDCl_3$, 128 MHz): δ 33.1 (s, 2B) (*B*–B_{cage}), 9.0 (s, 1B) (B_{cage}–C), -0.1 (d, J_{BH} = 121 Hz, 1B) (B_{cage}H), -6.0 (d, J_{BH} = 118 Hz, 2B) (B_{cage}H), -12.2 (m, 6B) (B_{cage}H & B–B_{cage}); analysis (calcd., found for $C_{21}H_{40}B_{12}O_4$): C (51.87, 51.68), H (8.29, 8.29).

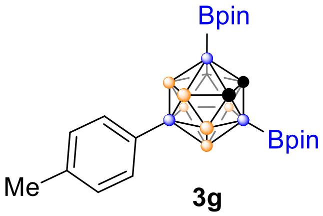

3g

3g: Yield 88%. White solid. ^{1}H NMR ($CDCl_3$, 300 MHz): δ 7.30 (d, *J* = 8.0 Hz, 2H), 7.03 (d, *J* = 8.0 Hz, 2H) (aromatic C*H*), 3.64 (s, 1H), 3.54 (s, 1H) (cage C*H*), 2.28 (s, 3H) (C_6H_4–C*H*$_3$), 1.28 (s, 24H) (OC–C*H*$_3$); ^{13}C{^{1}H} NMR ($CDCl_3$, 75 MHz): δ 136.5, 132.5, 128.1 (aromatic *C*), 84.3 (O*C*), 56.4, 51.9 (cage *C*H), 24.9, 21.3 (*C*H$_3$), the B_{cage}–*C* was not observed; ^{11}B NMR ($CDCl_3$, 128 MHz): δ 36.3 (s, 2B) (*B*–B_{cage}), 12.5 (s, 1B) (B_{cage}–C), 2.8 (d, J_{BH} = 110 Hz, 1B) (B_{cage}H), -2.9 (d, J_{BH} = 113 Hz, 2B) (B_{cage}H), -8.1 (m, 6B) (B_{cage}H & B–B_{cage}); analysis (calcd., found for $C_{21}H_{40}B_{12}O_4$): C (51.87, 51.76), H (8.29, 8.41).

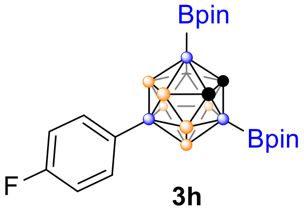

3h

3h: Yield 94%. White solid. ^{1}H NMR ($CDCl_3$, 300 MHz): δ 7.33 (m, 2H), 6.88 (m, 2H) (aromatic C*H*), 3.65 (s, 1H), 3.55 (s, 1H) (cage C*H*), 1.28 (s, 24H) (C*H*$_3$); ^{13}C{^{1}H} NMR ($CDCl_3$, 75 MHz): δ 162.7 (d, $^1J_{CF}$ = 263 Hz), 134.1 (d, $^3J_{CF}$ = 7.3 Hz), 114.1 (d, $^2J_{CF}$ = 19.6 Hz) (aromatic *C*), 84.3 (O*C*), 56.5, 52.1 (cage *C*H), 25.0 (*C*H$_3$), the B_{cage}–*C* was not observed; ^{11}B NMR ($CDCl_3$, 128 MHz): δ 32.5 (s, 2B) (*B*–B_{cage}), 8.9 (s, 1B) (B_{cage}–C), -0.3 (d, J_{BH} = 116 Hz, 1B) (B_{cage}H), -6.0 (d, J_{BH} = 108 Hz, 2B) (B_{cage}H), -11.9 (m,

6B) (B_{cage}H & B–B_{cage}); ^{19}F NMR ($CDCl_3$, 282 MHz): δ -116.7 (m, 1F); analysis (calcd., found for $C_{20}H_{37}B_{12}O_4F$): C (49.00, 48.98), H (7.61, 7.50).

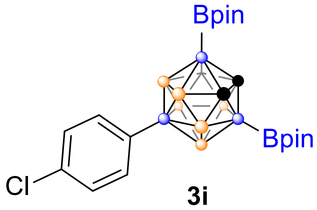

3i

3i: Yield 89%. White solid. ^{1}H NMR ($CDCl_3$, 300 MHz): δ 7.30 (d, J = 7.8 Hz, 2H), 7.15 (d, J = 7.8 Hz, 2H) (aromatic CH), 3.65 (s, 1H), 3.55 (s, 1H) (cage CH), 1.28 (s, 24H) (CH_3); ^{13}C{^{1}H} NMR ($CDCl_3$, 75 MHz): δ 133.9, 133.2, 127.4 (aromatic C), 84.4 (OC), 56.6, 52.4 (cage CH), 25.0 (CH$_3$), the $B_{cage–}C$ was not observed; ^{11}B NMR ($CDCl_3$, 128 MHz): δ 33.1 (s, 2B) (B–B_{cage}), 8.8 (s, 1B) (B_{cage}–C), -0.3 (d, J_{BH} = 137 Hz, 1B) (B_{cage}H), -6.0 (d, J_{BH} = 133 Hz, 2B) (B_{cage}H), -12.0 (m, 6B) (B_{cage}H & B–B_{cage}); analysis (calcd., found for $C_{20}H_{37}B_{12}O_4Cl$): C (47.41, 47.15), H (7.36, 7.24).

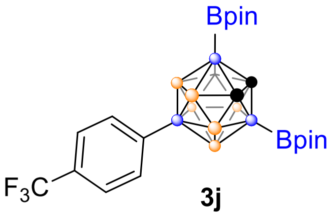

3j

3j: Yield 89%. White solid. ^{1}H NMR ($CDCl_3$, 300 MHz): δ 7.49 (d, J = 7.5 Hz, 2H), 7.42 (d, J = 7.5 Hz, 2H) (aromatic CH), 3.68 (s, 1H), 3.58 (s, 1H) (cage CH), 1.28 (s, 24H) (CH_3); ^{13}C{^{1}H} NMR ($CDCl_3$, 75 MHz): δ 132.8, 124.0, 107.8 (aromatic C), 84.5 (OC), 56.7, 52.9 (cage CH), 25.0 (CH$_3$), the $B_{cage–}C$ was not observed; and the CF$_3$ was not observed due to the poor solubility of **3j**; ^{11}B NMR ($CDCl_3$, 128 MHz): δ 32.4 (s, 2B) (B–B_{cage}), 8.4 (s, 1B) (B_{cage}–C), -0.6 (d, J_{BH} = 131 Hz, 1B) (B_{cage}H), -6.1 (d, J_{BH} = 115 Hz, 2B) (B_{cage}H), -12.1 (m, 6B) (B_{cage}H & B–B_{cage}); ^{19}F NMR ($CDCl_3$, 282 MHz): δ -62.5 (s, 3F); analysis (calcd., found for $C_{21}H_{37}B_{12}O_4F_3$): C (46.69, 46.67), H (6.90, 6.79).

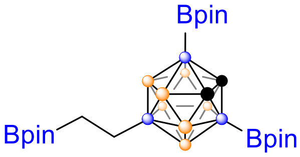

3k

3k: Yield 91%. White solid. ^{1}H NMR ($CDCl_3$, 300 MHz): δ 3.48 (s, 1H), 3.41 (s, 1H) (cage CH), 1.25 (s, 24H), 1.22 (s, 12H) (CH_3), 0.78 (m, 2H),

0.75 (m, 2H) (CH_2); ^{13}C{^{1}H} NMR ($CDCl_3$, 75 MHz): δ 84.1, 82.7 (O*C*), 56.2, 50.9 (cage *C*H), 24.9 (*C*H_3), the two B–*C*H_2 was not observed; ^{11}B NMR ($CDCl_3$, 128 MHz): δ 33.0 (s, 3B) (*B*pin), 11.5 (s, 1B) (B_{cage}–CH_2), -0.6 (d, J_{BH} = 125 Hz, 1B) (B_{cage}H), -6.3 (d, J_{BH} = 134 Hz, 2B) (B_{cage}H), -12.3 (m, 6B) (B_{cage}H & B–B_{cage}); analysis (calcd., found for $C_{22}H_{49}B_{13}O_6$): C (48.03, 48.01), H (8.98, 8.94).

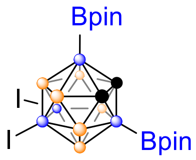

3l

3l: Yield 88%. White solid. ^{1}H NMR ($CDCl_3$, 300 MHz): δ 3.96 (s, 2H) (cage C*H*), 1.25 (s, 24H) (CH_3); ^{13}C{^{1}H} NMR ($CDCl_3$, 75 MHz): δ 84.8 (O*C*), 54.6 (cage *C*H), 25.0 (*C*H_3); ^{11}B NMR ($CDCl_3$, 128 MHz): δ 32.4 (s, 2B) (*B*–B_{cage}), -3.3 (d, J_{BH} = 152 Hz, 2B) (B_{cage}H), -11.0 (m, 4B) (B_{cage}H), -12.6 (s, 4B) (B_{cage}–I & B–B_{cage}); analysis (calcd., found for $C_{14}H_{32}B_{12}O_4I_2$): C (25.95, 26.07), H (4.98, 4.79).

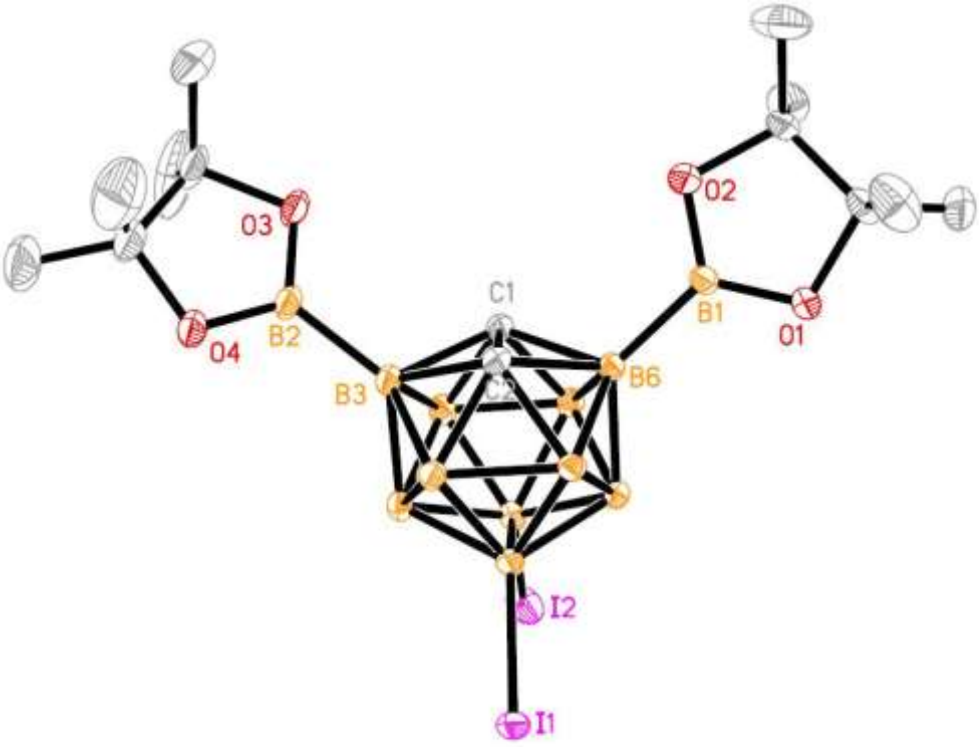

Supplementary Figure 2. Molecular structure of **3l** drawn with 30% probability ellipsoids.

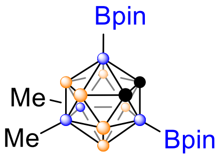

3m

3m: Yield 85%. White solid. ^{1}H NMR (CDCl$_3$, 300 MHz): δ 3.37 (s, 2H) (cage C*H*), 1.26 (s, 30H) (C*H*$_3$); ^{13}C{^{1}H} NMR (CDCl$_3$, 75 MHz): δ 84.1 (O*C*), 50.5 (cage *C*H), 25.0 (C*C*H$_3$), the two B$_{cage}$–*C*H$_3$ were not observed; ^{11}B NMR (CDCl$_3$, 128 MHz): δ 33.0 (s, 2B) (*B*–B$_{cage}$), 9.0 (s, 2B) (*B*$_{cage}$–CH$_3$), -4.6 (d, J_{BH} = 131 Hz, 2B) (*B*$_{cage}$H), -11.8 (d, J_{BH} = 138 Hz, 4B) (*B*$_{cage}$H), -15.1 (s, 2B) (B–*B*$_{cage}$); analysis (calcd., found for $C_{16}H_{38}B_{12}O_4$): C (45.30, 45.02), H (9.03, 8.70).

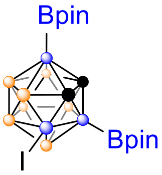

3n

3n: Yield 63%. White solid. ^{1}H NMR (CDCl$_3$, 300 MHz): δ 3.96 (s, 1H), 3.72 (s, 1H) (cage C*H*), 1.30 (s, 12H), 1.28 (s, 12H) (C*H*$_3$); ^{13}C{^{1}H} NMR (CDCl$_3$, 75 MHz): δ 84.7, 84.5 (O*C*), 59.8 (cage *C*H), 25.1, 25.0 (*C*H$_3$); ^{11}B NMR (CDCl$_3$, 128 MHz): δ 32.9 (s, 2B) (*B*–B$_{cage}$), 1.1 (d, J_{BH} = 123 Hz, 1B), 0.4 (d, J_{BH} = 96 Hz, 1B), -4.6 (d, J_{BH} = 179 Hz, 1B), -5.9 (d, J_{BH} = 148 Hz, 1B), -8.9 (d, J_{BH} = 161 Hz, 1B) (*B*$_{cage}$H), -11.6 (m, 3B) (*B*$_{cage}$H & B–*B*$_{cage}$), -25.1 (s, 2B) (*B*$_{cage}$–I & B–*B*$_{cage}$); analysis (calcd., found for $C_{14}H_{33}B_{12}O_4I$): C (32.21, 32.13), H (6.37, 6.40).

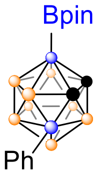

2o

2o: Yield 76%. White solid. ^{1}H NMR (CDCl$_3$, 300 MHz): δ 7.57 (m, 2H) 7.32 (m, 3H) (aromatic C*H*), 3.80 (s, 1H), 3.72 (s, 1H) (cage C*H*), 1.26 (s, 12H) (C*H*$_3$); ^{13}C{^{1}H} NMR (CDCl$_3$, 75 MHz): δ 133.4, 128.6, 127.9 (aromatic *C*), 84.4 (O*C*), 57.0, 56.6 (cage *C*H), 25.0 (*C*H$_3$), the B$_{cage}$–*C* were not observed; ^{11}B NMR (CDCl$_3$, 128 MHz): δ 32.9 (s, 1B) (*B*–B$_{cage}$), -1.4 (m, 4B) (*B*$_{cage}$H & *B*$_{cage}$–Ph), -6.8 (d, J_{BH} = 168 Hz, 1B) (*B*$_{cage}$H), -8.4 (d, J_{BH} = 154 Hz, 1B) (*B*$_{cage}$H), -11.6 (d, J_{BH} = 158 Hz, 1B) (*B*$_{cage}$H), -13.7 (m, 3B) (*B*$_{cage}$H & B–*B*$_{cage}$); analysis (calcd., found for $C_{14}H_{27}B_{11}O_2$): C (48.56, 48.37), H (7.86, 7.82).

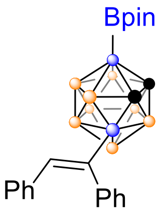

2p

2p: Yield 89%. White solid. ^{1}H NMR ($CDCl_3$, 300 MHz): δ 7.37 (m, 2H), 7.27 (m, 1H), 7.10 (m, 5H), 6.93 (m, 3H) (aromatic C*H* & olefinic C*H*), 3.53 (s, 1H), 3.36 (s, 1H) (cage C*H*), 1.26 (s, 12H) (CH_3); ^{13}C{^{1}H} NMR ($CDCl_3$, 75 MHz): δ 142.9, 138.0, 137.4, 129.4, 129.0, 128.0, 127.8, 127.0, 126.4 (aromatic and olefinic *C*), 84.3 (O*C*), 55.8, 55.5 (cage *C*H), 24.9 (*C*H_3), the B_{cage}-*C* was not observed; ^{11}B NMR ($CDCl_3$, 128 MHz): δ 32.9 (s, 1B) (*B*–B_{cage}), -1.6 (m, 3B) (B_{cage}H & B_{cage}–C), -6.7 (d, J_{BH} = 190 Hz, 1B), -8.3 (d, J_{BH} = 152 Hz, 1B) (B_{cage}H), -11.6 (m, 5B) (B_{cage}H & B–B_{cage}); analysis (calcd., found for $C_{22}H_{33}B_{11}O_2$): C (58.93, 59.05), H (7.42, 7.38).

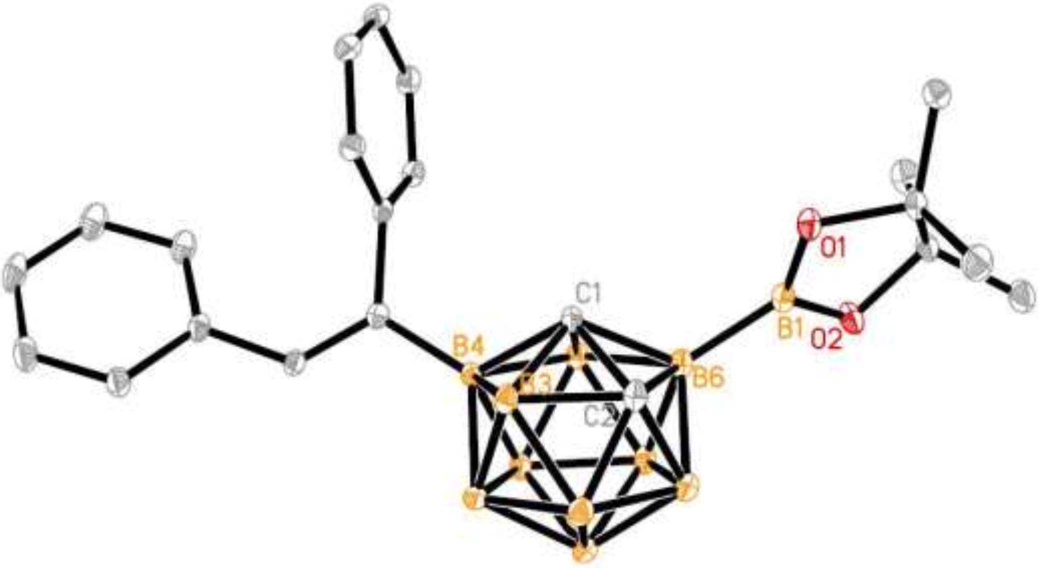

Supplementary Figure 3. Molecular structure of **2p** drawn with 30% probability ellipsoids.

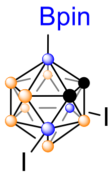

2q

2q: Yield 83%. White solid. ^{1}H NMR ($CDCl_3$, 300 MHz): δ 4.11 (s, 2H), (cage C*H*), 1.27 (s, 12H) (CH_3); ^{13}C{^{1}H} NMR ($CDCl_3$, 75 MHz): δ 84.9 (O*C*), 60.6 (cage *C*H), 25.0 (*C*H_3); ^{11}B NMR ($CDCl_3$, 128 MHz): δ 32.6 (s, 1B) (*B*–B_{cage}), 1.3 (d, J_{BH} = 149 Hz, 2B), -3.7 (d, J_{BH} = 124 Hz, 1B), -6.7 (d, J_{BH} = 151 Hz, 1B), -10.4 (d, J_{BH} = 169 Hz, 1B), -11.8 (d, J_{BH} =

161 Hz, 2B) (B_{cage}H), -26.0 (s, 3B) (B_{cage}–I & B–B_{cage}); analysis (calcd., found for $C_8H_{21}B_{11}O_2I_2$): C (18.41, 18.64), H (4.06, 4.04).

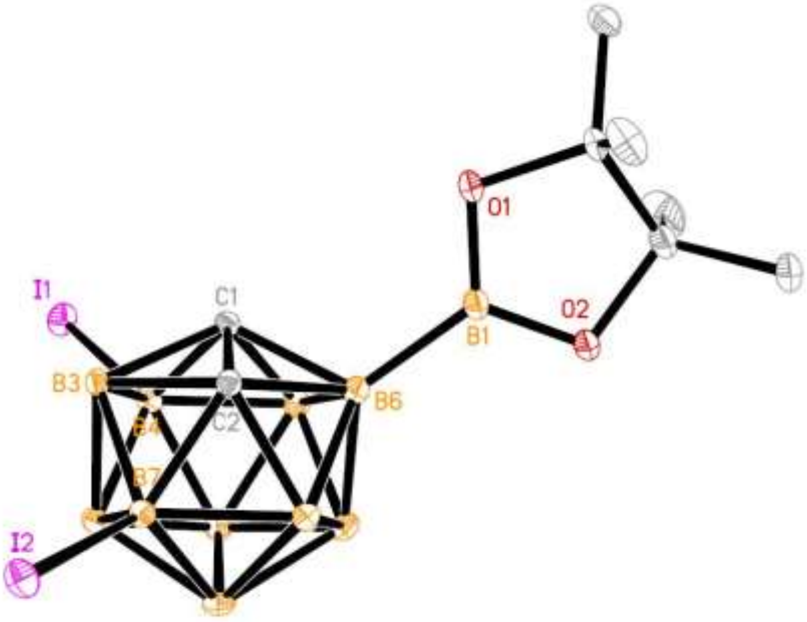

Supplementary Figure 4. Molecular structure of **2q** drawn with 30% probability ellipsoids.

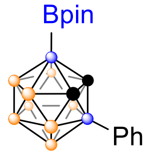

2r

2r: Yield 89%. White solid. ^{1}H NMR ($CDCl_3$, 300 MHz): δ 7.65 (m, 2H), 7.41 (m, 3H) (aromatic C*H*), 3.71 (s, 2H) (cage C*H*), 1.27 (s, 12H) (CH_3); ^{13}C{^{1}H} NMR ($CDCl_3$, 75 MHz): δ 133.4, 129.8, 128.3 (aromatic *C*), 84.4 (O*C*), 58.5 (cage *C*H), 25.0 (*C*H_3), the B_{cage}–*C* was not observed; ^{11}B NMR ($CDCl_3$, 128 MHz): δ 32.8 (s, 1B) (*B*–B_{cage}), -1.4 (d, J_{BH} = 145 Hz, 2B) (B_{cage}H), -4.5 (s, 1B) (B_{cage}–Ph), -6.7 (d, J_{BH} = 142 Hz, 1B) (B_{cage}H), -12.3 (m, 6B) (B_{cage}H & B–B_{cage}); analysis (calcd., found for $C_{14}H_{27}B_{11}O_2$): C (48.56, 48.93), H (7.86, 8.00).

Gram scale synthesis of 3a.

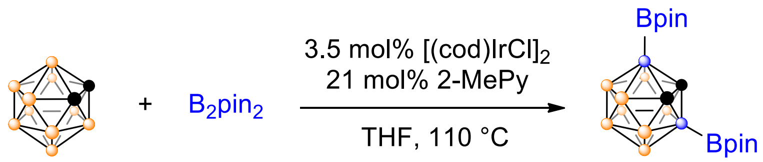

An oven-dried Schlenk flask equipped with a stir bar was charged with *o*-carborane (**1**) (1.44 g, 10 mmol), B_2pin_2 (10.16 g, 40 mmol), [(cod)IrCl]$_2$ (236 mg, 0.35 mmol), and 2-methylpyridine (195 mg, 2.1 mmol), followed by dry THF (80 mL). The flask was closed under an atmosphere of nitrogen and stirred at 110 ^{o}C (bath temperature) for 5 h. After hydrolysis with water (80 mL) and extraction with diethyl ether (80 mL x 3), the ether solutions were combined and concentrated to dryness in vacuo. The residue was subjected to flash column chromatography on silica gel (230-400 mesh) using *n*-hexane and ethyl acetate (10/1 in v/v) as eluent to give a mixture of product and B_2pin_2. Removal of B_2pin_2 via sublimation at 90 ^{o}C under vacuum (0.1 torr) afforded a pure product **3a** (3.75 g, 95%).

Preparation of 3-Bpin-*o*-$C_2B_{10}H_{11}$ (2a).

An oven-dried Schlenk flask equipped with a stir bar was charged with *o*-carborane (**1**) (72 mg, 0.50 mmol), B_2pin_2 (152 mg, 0.60 mmol), [(cod)IrCl]$_2$ (11.8 mg, 0.0175 mmol), and 2,6-dimethylpyridine (9.8 mg, 0.105 mmol), followed by dry THF (5 mL). The flask was closed under an atmosphere of nitrogen and stirred at 80 ^{o}C for 12 h. After hydrolysis with water (10 mL) and extraction with diethyl ether (10 mL x 3), the ether solutions were combined and concentrated to dryness in vacuo. The residue was subjected to flash column chromatography on silica gel (230-400 mesh) using *n*-hexane and ethyl acetate (10/1 in v/v) as eluent to give **2a** as a white solid (60 mg, 44%).

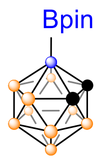

2a

2a: ^{1}H NMR ($CDCl_3$, 300 MHz): δ 3.56 (s, 2H) (cage C*H*), 1.26 (s, 12H) (CH_3); ^{13}C{^{1}H}

NMR ($CDCl_3$, 75 MHz): δ 84.4 (O*C*), 56.3 (cage *C*H), 25.0 (*C*H_3); ^{11}B NMR ($CDCl_3$, 128 MHz): δ 32.9 (s, 1B) (*B*–B_{cage}), -1.4 (d, J_{BH} = 147 Hz, 2B), -7.5 (d, J_{BH} = 157 Hz, 1B), -8.0 (d, J_{BH} = 80 Hz, 1B) (B_{cage}H), -12.5 (d, J_{BH} = 164 Hz, 5B) (B_{cage}H & B–B_{cage}), -11.8 (d, J_{BH} = 173 Hz, 1B) (B_{cage}H); analysis (calcd., found for $C_8H_{23}B_{11}O_2$): C (35.56, 35.41), H (8.58,8.55).

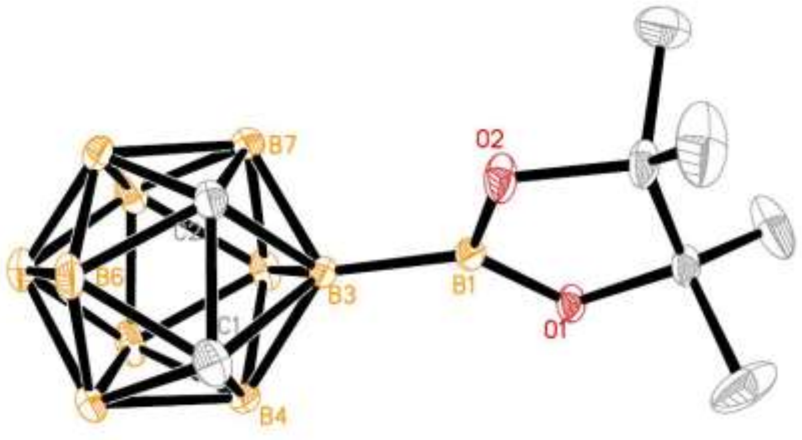

Supplementary Figure 5. Molecular structure of **2a** drawn with 30% probability ellipsoids.

B(2)-borylation of *m*-carborane.

Supplementary Table 3. Optimization of conditions for B(2)-borylation of *m*-carborane [a]

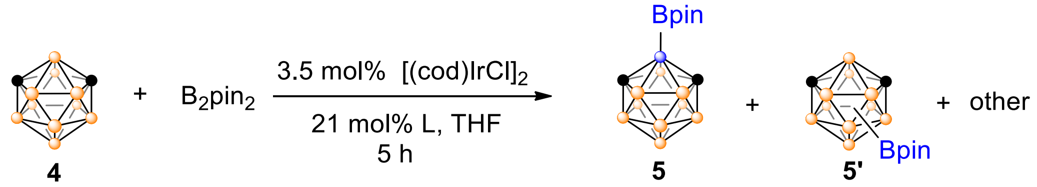

Entry	L (mol %)	T / °C	B_2pin_2 (equiv)	Yield (%)[b]			
				**4**	**5**	**5'**	other[c]
1	2-MePy	110	4	-	77	-	23
2	2-MePy	110	10	-	63	-	37
3	2-MePy	80	4	-	70	-	30
4	2-MePy	80	2	5	84	4	7
5	2-MePy	80	1.5	6	85	4	5
6	2,6-Me_2Py	80	2	6	85	4	5
7	4-^{t}BuPy	80	2	25	63	8	3

8	2-PhPy	80	2	46	50	4	-
9	2,6-F_2Py	80	2	39	60	1	-
10[d]	2,2'-bipy	80	2	20	29	31	20
11[d]	4,4'-Me_2bipy	80	2	10	47	19	24

[a]Reaction condition: **4** (0.2 mmol), 3.5 mol% [(cod)IrCl]$_2$, 21 mol% L. [b]GC yield. [c]Other: geometrical isomers of diborylated *o*-carboranes. [d]14 mol% L.

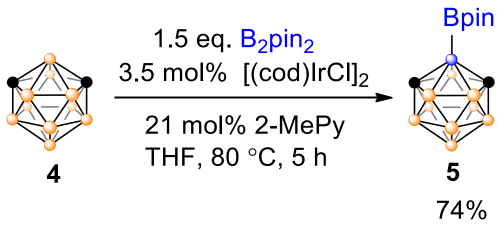

Supplementary Figure 6. Regioselective synthesis of 2-Bpin-*m*-carborane.

Preparation of 2-Bpin-*m*-$C_2B_{10}H_{11}$ (5). An oven-dried Schlenk flask equipped with a stir bar was charged with *m*-carborane (72 mg, 0.50 mmol), B_2pin_2 (191 mg, 0.75 mmol), [(cod)IrCl]$_2$ (11.8 mg, 0.0175 mmol), and 2-methylpyridine (9.8 mg, 0.105 mmol), followed by dry THF (5 mL). The flask was closed under an atmosphere of nitrogen and stirred at 80 ^oC for 5 h. After hydrolysis with water (10 mL) and extraction with diethyl ether (10 mL x 3), the ether solutions were combined and concentrated to dryness in vacuo. The residue was subjected to flash column chromatography on silica gel (230-400 mesh) using *n*-hexane and ethyl acetate (30/1 in v/v) as eluent to give **5** as a white solid (100 mg, 74%).

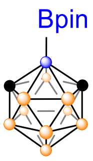

5

5: Yield 74%. White solid. ^{1}H NMR ($CDCl_3$, 300 MHz): δ 2.92 (s, 2H) (cage C*H*), 1.27 (s, 12H) (C*H*$_3$); $^{13}C\{^1H\}$ NMR ($CDCl_3$, 75 MHz): δ 84.2 (O*C*), 57.0 (cage *C*H), 24.9 (*C*H$_3$); ^{11}B NMR ($CDCl_3$, 128 MHz): δ 34.8 (s, 1B) (*B*–B$_{cage}$), -5.0 (d, J_{BH} = 162 Hz, 2B) (B_{cage}H), -8.0 (m, 2B) (B_{cage}H), -11.1 (d, J_{BH} = 163 Hz, 4B) (B_{cage}H), -15.2 (m, 2B) (B_{cage}H & B–B_{cage}); analysis (calcd., found for $C_8H_{23}B_{11}O_2$): C (35.56, 35.43), H (8.58, 8.74).

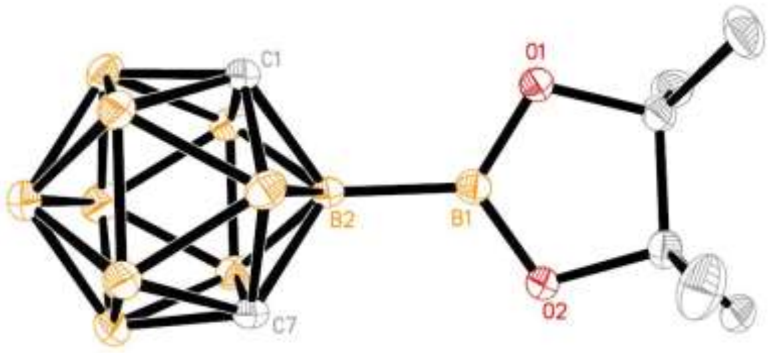

Supplementary Figure 7. Molecular structure of **5** drawn with 30% probability ellipsoids.

B(4)-borylation of *o*-carboranes.

Supplementary Table 4. Optimization of B(4)−H borylation reaction.*

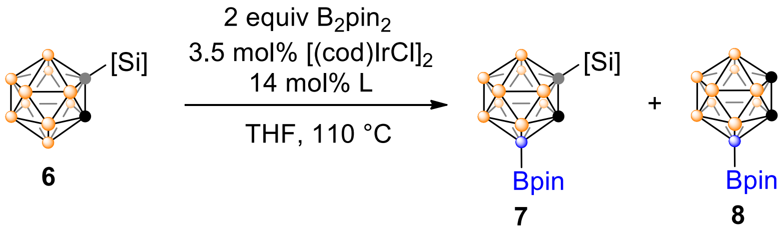

Entry	[Si]	L	Time (h)	Yield (%)†			
				**6**	**7**	**8**	other‡
1	TMS	4,4'-Me_2bipy	1	5	54	5	36
2	TMS	4,4'-dtbpy	1	4	43	12	41
3	TMS	2,2'-bipy	1	8	69	8	15
4	TES	2,2'-bipy	1	5	80	1	11
5	TBDMS	2,2'-bipy	1	10	86	-	4
6	TBDMS	2,2'-bipy	3	4	90	-	6
7	TBDMS	2,2'-bipy	5	-	88	-	12

*Reactions were conducted on 0.2 mmol scale in a closed flask. TMS = trimethylsilyl, TES = triethylsilyl, TBDMS = tert-butyldimethylsilyl; 4,4'-Me_2bipy = 4,4'-dimethyl-2,2'-bipyridine, 4,4'-dtbpy = 4,4'-di-tert-butyl-2,2'-bipyridine, 2,2'-bipy = 2,2'-bipyridine. †GC yield. ‡Other: geometrical isomers of diborylated *o*-carboranes.

Preparation of B(4)-borylated *o*-Carboranes (8). A Representative Procedure.

An oven-dried Schlenk flask equipped with a stir bar was charged with 1-TBDMS-*o*-carboranes (0.5

mmol), B_2pin_2 (254 mg, 1.0 mmol), $[(cod)IrCl]_2$ (11.8 mg, 0.0175 mmol), and 2,2'-bipyridine (10.9 mg, 0.07 mmol), followed by dry THF (5 mL). The flask was closed under an atmosphere of nitrogen and stirred at 110 ^{o}C (bath temperature) for 3 h. After hydrolysis with water (10 mL) and extraction with diethyl ether (10 mL x 3), the ether solutions were combined and concentrated to dryness in vacuo. The residue was subjected to flash column chromatography on silica gel (230-400 mesh) using *n*-hexane and ethyl acetate (10/1 in v/v) as eluent to give a mixture of product and B_2pin_2. Removal of B_2pin_2 by sublimation at 90 ^{o}C under vacuum (0.1 torr) afforded the pure product **7**.

To a solution (2 mL) of **7** (0.3 mmol) (acetone for **7a** and **7b**; MeOH/DCM (2/1 in v/v) for **7c**) was added CsF (182 mg, 1.2 mmol). The mixture was stirred at room temperature for 1 h for **7a**,**7b** and 20 min for **7c**. After filtration, the filtrate was concentrated to dryness in vacuo. The residue was subjected to flash column chromatography on silica gel (230−400 mesh) using *n*-hexane and ethyl acetate (10/1 in v/v) as eluent to give product **8**.

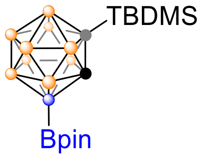

7a

7a: Yield 92%. White solid. ^{1}H NMR ($CDCl_3$, 300 MHz): δ 3.46 (s, 1H) (cage C*H*), 1.24 (s, 6H), 1.23 (s, 6H) ($OC(CH_3)_2$), 1.01 (s, 9H) ($C(CH_3)_3$), 0.23 (s, 3H), 0.22 (s, 3H) ($SiCH_3$); $^{13}C\{^{1}H\}$ NMR ($CDCl_3$, 75 MHz): δ 83.6 (O*C*), 67.6 (cage *C*), 61.9 (cage *C*H), 27.1 ($C(CH_3)_3$), 25.0, 24.8 ($OC(CH_3)_2$), 19.4 ($SiC(CH_3)_3$), -4.2 ($SiCH_3$); ^{11}B NMR ($CDCl_3$, 128 MHz): δ 34.3 (s, 1B) (B–B_{cage}), -0.7 (d, J_{BH} = 152 Hz, 1B), -1.0 (d, J_{BH} = 143 Hz, 1B), -5.9 (d, J_{BH} = 144 Hz, 2B), -9.8 (m, 2B), -10.9 (m, 2B) (B_{cage}H), -12.4 (m, 2B) (B_{cage}H & B–B_{cage}); analysis (calcd., found for $C_{14}H_{37}B_{11}O_2Si$): C (43.74, 43.73), H (9.70, 9.73).

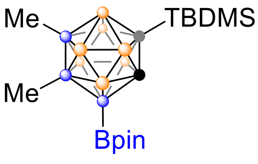

7b

7b: Yield 92%. White solid. ^{1}H NMR ($CDCl_3$, 300 MHz): δ 3.27 (s, 1H) (cage C*H*), 1.24 (s, 6H), 1.23 (s, 6H) ($OC(CH_3)_2$), 1.0 (s, 9H) ($C(CH_3)_3$), 0.21 (s, 3H), 0.20 (s, 3H) ($SiCH_3$), 0.20 (s, 3H), 0.16 (s, 3H) (B_{cage}-CH_3); ^{13}C{^{1}H} NMR ($CDCl_3$, 75 MHz): δ 83.3 (O*C*), 58.9 (cage *C*), 55.1 (cage *C*H), 27.1 ($C(CH_3)_3$), 25.2, 24.7 ($OC(CH_3)_2$), 19.4 ($SiC(CH_3)_3$), -4.3 ($SiCH_3$), the two B_{cage}–CH_3 were not observed; ^{11}B NMR ($CDCl_3$, 128 MHz): δ 33.8 (s, 1B) (*B*–B_{cage}), 10.9 (s, 1B), 8.6 (s, 1B) (B_{cage}–C), -4.1 (d, J_{BH} = 134 Hz, 2B), -9.8 (d, J_{BH} = 143 Hz, 2B), -11.1 (d, J_{BH} = 179 Hz, 2B) (B_{cage}H), -14.0 (m, 2B) (B_{cage}H & B–B_{cage}); analysis (calcd., found for $C_{16}H_{41}B_{11}O_2Si$): C (46.59, 47.11), H (10.02, 10.02).

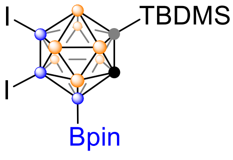

7c

7c: Yield 89%. White solid. ^{1}H NMR ($CDCl_3$, 300 MHz): δ 3.84 (s, 1H) (cage C*H*), 1.27 (s, 6H), 1.26 (s, 6H) ($OC(CH_3)_2$), 1.01 (s, 9H) ($C(CH_3)_3$), 0.24 (s, 6H) ($SiCH_3$); ^{13}C{^{1}H} NMR ($CDCl_3$, 75 MHz): δ 84.2 (O*C*), 63.9 (cage *C*), 58.6 (cage *C*H), 27.0 ($C(CH_3)_3$), 25.2, 24.7 ($OC(CH_3)_2$), 19.4 ($SiC(CH_3)_3$), -4.4 ($SiCH_3$); ^{11}B NMR ($CDCl_3$, 128 MHz): δ 33.4 (s, 1B) (*B*–B_{cage}), -2.8 (d, J_{BH} = 163 Hz, 2B), -9.1 (m, 2B), -10.5 (m, 1B) (B_{cage}H), -11.6 (m, 3B) (B_{cage}H & B–B_{cage}), -12.9 (s, 2B) (B_{cage}–I); analysis (calcd., found for $C_{14}H_{35}B_{11}I_2O_2Si$): C (26.43, 26.47), H (5.54, 5.82).

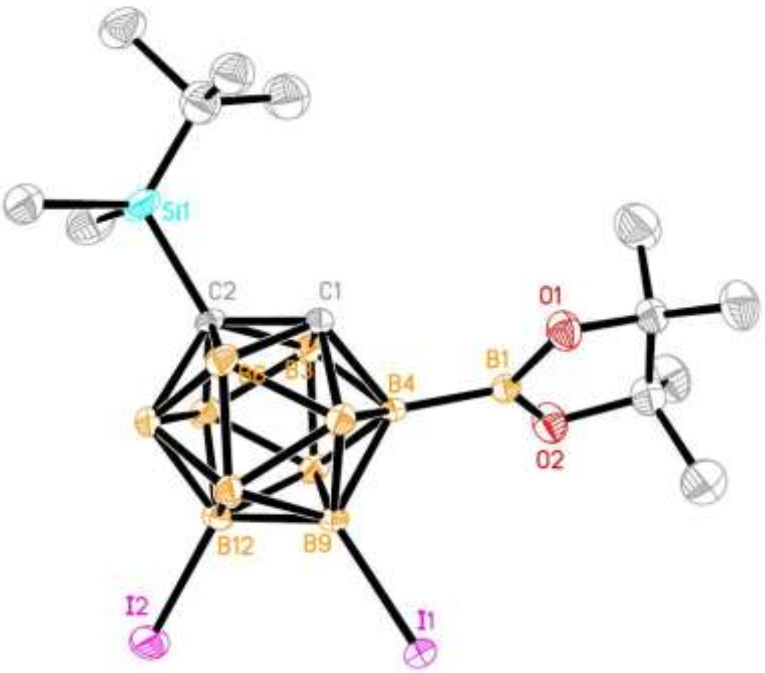

Supplementary Figure 8. Molecular structure of **7c** drawn with 30% probability ellipsoids.

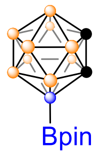

8a

8a: Yield 95%. White solid. ^{1}H NMR ($CDCl_3$, 300 MHz): δ 3.60 (s, 2H) (cage C*H*), 1.24 (s, 12H) (CH_3); ^{13}C{^{1}H} NMR ($CDCl_3$, 75 MHz): δ 83.7 (O*C*), 56.1, 55.9 (cage *CH*), 24.9 (*C*H_3); ^{11}B NMR ($CDCl_3$, 128 MHz): δ 33.9 (s, 1B) (*B*–B_{cage}), -1.5 (d, J_{BH} = 168 Hz, 2B) (B_{cage}H), -8.1 (d, J_{BH} = 137 Hz, 2B) (B_{cage}H), -12.1 (m, 3B) (B_{cage}H & B–B_{cage}), -13.9 (d, J_{BH} = 182 Hz, 3B) (B_{cage}H); analysis (calcd., found for $C_8H_{23}B_{11}O_2$): C (35.56, 35.65), H (8.58, 8.62).

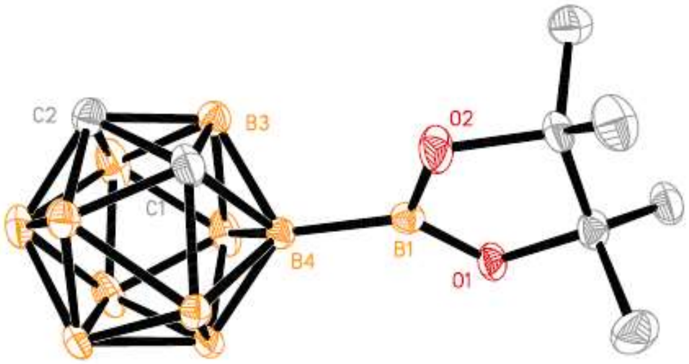

Supplementary Figure 9. Molecular structure of **8a** drawn with 30% probability ellipsoids.

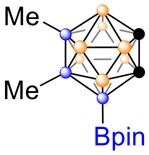

8b

8b: Yield 94%. White solid. ^{1}H NMR ($CDCl_3$, 300 MHz): δ 3.40 (s, 2H) (cage C*H*), 1.24 (s, 6H), 1.22 (s, 6H) (OC(CH_3)$_2$), 0.21 (s, 3H), 0.19 (s, 3H) (BCH_3); ^{13}C{^{1}H} NMR ($CDCl_3$, 75 MHz): δ 83.4 (O*C*), 49.2, 49.0 (cage *C*H), 25.1, 24.8 (OC(*C*H_3)$_2$), the two B_{cage}–*C*H_3 were not observed; ^{11}B NMR ($CDCl_3$, 128 MHz): δ 35.1 (s, 1B) (*B*–B_{cage}), 9.6 (s, 2B) (B_{cage}–CH_3), -5.0 (d, J_{BH} = 145 Hz, 2B) (B_{cage}H), -11.5 (m, 4B) (B_{cage}H & B–B_{cage}), -14.3 (d, J_{BH} = 169 Hz, 2B) (B_{cage}H); analysis (calcd., found for $C_{10}H_{27}B_{11}O_2$): C (40.27, 40.58), H (9.12, 9.19).

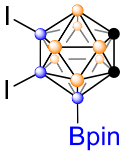

8c

8c: Yield 95%. White solid. ^{1}H NMR ($CDCl_3$, 300 MHz): δ 4.06 (s, 2H) (cage C*H*), 1.32 (s, 12H) (CH_3); ^{13}C{^{1}H} NMR ($CDCl_3$, 75 MHz): δ 84.4 (O*C*), 53.4, 53.1 (cage *C*H), 25.2, 24.8 (*C*H_3); ^{11}B NMR ($CDCl_3$, 128 MHz): δ 35.1 (s, 1B) (*B*–B_{cage}), -4.6 (d, J_{BH} = 161 Hz, 2B), -11.7 (m, 2B) (B_{cage}H), -13.1 (m, 6B) (B_{cage}H, B_{cage}–I & B–B_{cage}); analysis (calcd., found for $C_8H_{21}B_{11}I_2O_2$): C (18.41, 18.33), H (4.06, 3.94).

Transformation of 3,6-(Bpin)$_2$-*o*-carborane (3a)

Preparation of 3,6-Diphenyl-*o*-carborane (9)

An oven-dried Schlenk flask equipped with a stir bar was charged with **3a** (198 mg, 0.5 mmol), bromobenzene (236 mg, 1.5 mmol), $Pd(PPh_3)_4$ (115 mg, 0.1 mmol) and Cs_2CO_3 (490 mg, 1.5 mmol), followed by dry cyclohexane (5 mL). The flask was closed under an atmosphere of nitrogen and stirred at 150 ^{o}C (bath temperature) for 8 h. After cooled to room temperature, water (10 mL) and 30% H_2O_2 (5 mL) aqueous solution were successively added and the mixture was stirred at room temperature for 15 min. After extraction with diethyl ether (10 mL x 3), the ether solutions were

combined and concentrated to dryness in vacuo. The residue was subjected to flash column chromatography on silica gel (230-400 mesh) using *n*-hexane and ethyl acetate (100/1 in v/v) as eluent to give a white solid. Removal of biphenyl byproduct via sublimation at 90 °C under vacuum (0.1 torr) gave the pure 3,6-diphenyl-*o*-carborane (**9**) as a white solid (120 mg, 81%).

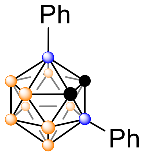

9

9: ^{1}H NMR ($CDCl_3$, 300 MHz): δ 7.62 (m, 4H) 7.40 (m, 6H) (aromatic C*H*), 3.85 (s, 2H) (cage C*H*); ^{13}C{^{1}H} NMR ($CDCl_3$, 75 MHz): *δ* 133.3, 130.0, 128.4 (aromatic *C*), 59.2 (cage *C*H), the two B_{cage}–*C* were not observed; ^{11}B NMR ($CDCl_3$, 128 MHz): *δ* -2.2 (d, J_{BH} = 149 Hz, 2B) (B_{cage}H), -3.7 (s, 2B) (B_{cage}–Ph), -11.2 (d, J_{BH} = 182 Hz, 2B) (B_{cage}H), -12.8 (d, J_{BH} = 173 Hz, 4B) (B_{cage}H); analysis (calcd., found for $C_{14}H_{20}B_{10}$): C (56.73, 56.18), H (6.80, 6.71).

Preparation of 3,6-Diallyl-*o*-carborane (10)

Compound **3a** (198 mg, 0.5 mmol), allyl chloride (230 mg, 3.0 mmol), $Pd(dba)_2$ (58 mg, 0.1 mmol) and Cs_2CO_3 (490 mg, 1.5 mmol) were mixed in toluene (6 mL). The resulting mixture was stirred at room temperature under nitrogen atmosphere for 8 h. After hydrolysis with water (10 mL) and extraction with diethyl ether (10 mL x 3), the ether solutions were combined and concentrated to dryness in vacuo. The residue was subjected to flash column chromatography on silica gel (230-400 mesh) using *n*-hexane as eluent to give **10** as a colorless liquid (97 mg, 87%).

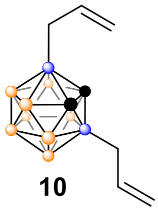
10

10: ^{1}H NMR ($CDCl_3$, 300 MHz): δ 5.80 (m, 2H) (C*H*=CH_2), 4.96 (m, 4H) (CH_2=CH), 3.25 (s, 2H) (cage C*H*), 2.01 (d, *J* = 6.9, 4H) (B_{cage}–CH_2); ^{13}C{^{1}H} NMR ($CDCl_3$, 75 MHz): *δ* 135.9, 115.7 (olefinic *C*), 57.6 (cage *C*H), the two B_{cage}-CH_2 were not observed; ^{11}B NMR ($CDCl_3$, 128 MHz): *δ* -2.3 (d, J_{BH} = 145 Hz, 2B) (B_{cage}H), -4.0 (s, 2B) (B_{cage}–C),

-12.4 (m, 6B) (B_{cage}H); analysis (calcd., found for $C_8H_{20}B_{10}$): C (42.83, 43.31), H (8.99, 8.94).

Preparation of 3,6-Dihalogen-*o*-carborane (11). A Representative Procedure.

An oven-dried Schlenk flask equipped with a stir bar was charged with **3a** (198 mg, 0.5 mmol), PhX (X = Br or I; 1.5 mmol), $Pd(PPh_3)_4$ (58 mg, 0.05 mmol) and ^{t}BuOK(168 mg, 1.5 mmol), followed by dry THF (7 mL). The flask was closed under an atmosphere of nitrogen and stirred at 80 ^{o}C for 24 h. After cooling to room temperature, water (10 mL) and 30% H_2O_2 (5 mL) aqueous solution were successively added and the mixture was stirred at room temperature for 15 min.. After extraction with diethyl ether (10 mL x 3), the ether solutions were combined and concentrated to dryness in vacuo. The residue was subjected to flash column chromatography on silica gel (230-400 mesh) using *n*-hexane as eluent to give **11**.

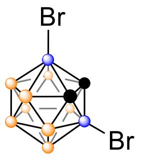

11a

11a: Yield 73%. White solid. ^{1}H NMR ($CDCl_3$, 300 MHz): δ 4.14 (s, 2H) (cage C*H*); ^{13}C{^{1}H} NMR ($CDCl_3$, 100 MHz): δ 63.4 (cage *C*H); ^{11}B{^{1}H} NMR ($CDCl_3$, 128 MHz): δ -1.8 (d, J_{BH} = 145 Hz, 2B) (B_{cage}H), -11.4 (m, 6B) (B_{cage}–Br & B_{cage}H), 12.3 (d, J_{BH} = 96 Hz, 2B) (B_{cage}H); analysis (calcd., found for $C_2H_{10}B_{10}Br_2$): C (7.95, 8.21), H (3.34, 3.33).

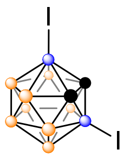

11b

11b: Yield 78%. White solid. ^{1}H NMR ($CDCl_3$, 300 MHz): δ 4.13 (s, 2H) (cage C*H*); ^{13}C{^{1}H} NMR ($CDCl_3$, 100 MHz): δ 64.3 (cage *C*H); ^{11}B NMR ($CDCl_3$, 128 MHz): δ -0.1 (d, J_{BH} = 151 Hz, 2B), -8.7 (d, J_{BH} = 134 Hz, 2B), -9.9 (d, J_{BH} = 162 Hz, 4B) (B_{cage}H), -27.8 (s, 2B) (B_{cage}–I); analysis (calcd., found for $C_2H_{10}B_{10}I_2$): C (6.07, 6.10), H (2.55, 2.53).

Preparation of 3,6-Diacetoxy-*o*-carborane (12).

Compound **3a** (198 mg, 0.5 mmol), $Cu(OAc)_2$ (545 mg, 3 mmol), KF (175 mg, 3 mmol) and 4 Å molecular sieve (150 mg) were mixed in CH_3CN (2.5 mL) in a 25 mL Schlenk flask. The mixture was heated at 80 ^{o}C under O_2 atmosphere (1 atm) for 12 h. After hydrolysis with water (10 mL) and extraction with diethyl ether (10 mL x 3), the ether solutions were combined and concentrated to dryness in vacuo. The residue was subjected to flash column chromatography on silica gel (230-400 mesh) using *n*-hexane/ethyl acetate (10/1 in v/v) as eluent to give **12** as a white solid (104 mg, 80%).

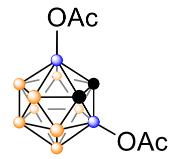

12

12: ^{1}H NMR ($CDCl_3$, 300 MHz): δ 4.79 (s, 2H) (cage C*H*), 2.17 (s, 6H) (COCH_3); $^{13}C\{^{1}H\}$ NMR ($CDCl_3$, 100 MHz): δ 170.0 (*C*=O), 55.7 (cage *C*H), 22.5 (CH_3); ^{11}B NMR ($CDCl_3$, 128 MHz): δ -4.3 (s, 2B) (B_{cage}–O), -7.4 (d, J_{BH} = 150 Hz, 2B) (B_{cage}H), -15.7 (d, J_{BH} = 164 Hz, 4B) (B_{cage}H), -18.1 (d, J_{BH} = 156 Hz, 2B) (B_{cage}H); analysis (calcd., found for $C_6H_{16}B_{10}O_4$): C (27.69, 27.66), H (6.20, 6.15).

Preparation of 3,6-Diamino-*o*-carborane (13).

To a THF solution of $MeONH_2$ (6.0 mL, 3.0 mmol), prepared in situ from the reaction of $MeONH_2 \cdot HCl$ (251 mg, 3.0 mmol) with NaH (85 mg, 3.5 mmol) at room temperature for 12 h, was slowly added ^{n}BuLi (1.6 M, 1.9 mL, 3.0 mmol) at -78 ^{o}C under an atmosphere of dry nitrogen. After stirring at room temperature for 30 min, a THF solution (4 mL) of **3a** (198 mg, 0.5 mmol) was added. The resulting mixture was heated in a closed flask at 80 ^{o}C for 8 h. After hydrolysis with water (10 mL) and extraction with diethyl ether (10 mL x 3), the ether solutions were combined and concentrated to dryness in vacuo. The residue was subjected to flash column chromatography on

silica gel (230-400 mesh) using *n*-hexane/ethyl acetate (4/1 in v/v) as eluent to give **13** as a white solid (78 mg, 90%).

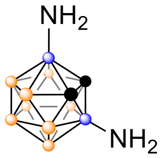

13

13: ^{1}H NMR ($CDCl_3$, 400 MHz): δ 3.58 (s, 2H) (cage C*H*), 1.45 (s, 4H) (N*H*$_2$); ^{13}C{^{1}H} NMR ($CDCl_3$, 125 MHz): δ 62.5 (cage *C*H); ^{11}B NMR ($CDCl_3$, 128 MHz): δ 1.1 (s, 2B) (B_{cage}–N), -6.4 (d, J_{BH} = 147 Hz, 2B) (B_{cage}H), -16.3 (d, J_{BH} = 158 Hz, 4B) (B_{cage}H), -19.1 (d, J_{BH} = 150 Hz, 2B) (B_{cage}H); analysis (calcd., found for $C_2H_{14}B_{10}N_2$): C (13.79, 13.98), H (8.10, 8.07), N (16.08, 15.77).

Preparation of 3,6-Diazide-*o*-carborane (14).

To a THF suspension (3 mL) of **3a** (198 mg, 0.5 mmol), CuCl (104 mg, 1.05 mmol) and KF (70 mg, 1.2 mmol) was added $TMSN_3$ (138 mg, 1.2 mmol). The resulting mixture was stirred at 60 ^{o}C for 24 h. After hydrolysis with water (10 mL) and extraction with diethyl ether (10 mL x 3), the ether solutions were combined and concentrated to dryness in vacuo. The residue was subjected to flash column chromatography on silica gel (230-400 mesh) using *n*-hexane/ethyl acetate (100/1 in v/v) as eluent to give the product **14** as a white solid (94 mg, 83%).

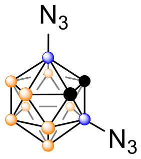

14

14: ^{1}H NMR ($CDCl_3$, 300 MHz): δ 3.67 (s, 2H) (cage C*H*); ^{13}C{^{1}H} NMR ($CDCl_3$, 75 MHz): δ 57.2 (cage *C*H); ^{11}B NMR ($CDCl_3$, 128 MHz): δ -4.2 (s, 2B) (B_{cage}–N), -4.6 (d, J_{BH} = 117 Hz, 2B) (B_{cage}H), -14.6 (d, J_{BH} = 166 Hz, 4B) (B_{cage}H), -17.0 (d, J_{BH} = 153 Hz, 2B) (B_{cage}H); analysis (calcd., found for $C_2H_{10}B_{10}N_6$): C (10.62, 10.46), H (4.45, 4.45), N (37.14, 37.05).

Preparation of 3,6-Ditriazolyl-*o*-carborane by Click Reaction.

Compound **14** (68 mg, 0.3 mmol) and diethyl but-2-ynedioate (123 mg, 0.72 mmol) were dissolved in toluene (1 mL). The resulting mixture was stirred at 95 °C for 5 h. After removal of the solvent in vacuo, the residue recrystallized from *n*-hexane/diethyl ether (5/1 in v/v) to give **15** as a white solid (141 mg, 83%).

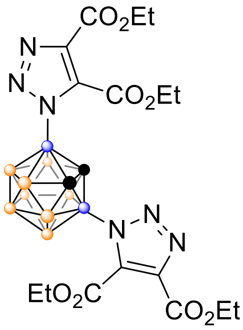

15

15: ^{1}H NMR ($CDCl_3$, 300 MHz): δ 5.33 (s, 2H) (cage C*H*), 4.52 (q, *J* = 7.2 Hz, 4H), 4.43 (q, *J* = 6.9 Hz, 4H) (CH_2), 1.45 (t, *J* = 7.2 Hz, 6H), 1.39 (t, *J* = 7.2 Hz, 6H) (CH_3), ^{13}C{^{1}H} NMR ($CDCl_3$, 75 MHz): δ 159.6, 159.2 (*C*=O), 139.2, 138.8 (olefinic *C*), 64.0, 62.1 (O*C*H_2), 60.6 (cage *C*H), 14.2, 13.9 (*C*H_3), ^{11}B NMR ($CDCl_3$, 128 MHz): δ -2.8 (m, 2B) (B_{cage}H), -7.1 (s, 2B) (B_{cage}–N), -12.9 (m, 6B) (B_{cage}H); analysis (calcd., found for $C_{18}H_{30}B_{10}N_6O_8$): C (38.16, 37.95), H (5.34, 5.31), N (14.83, 14.79).

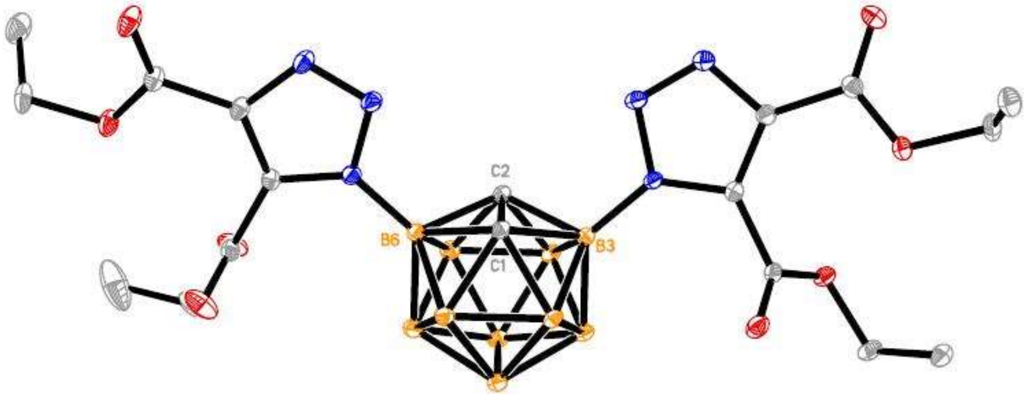

Supplementary Figure 10. Molecular structure of **15** drawn with 30% probability ellipsoids.

Preparation of 3,6-[B(OH)$_2$]$_2$-*o*-Carborane (16).

To a solution of **3a** (396 mg, 1.0 mmol) in ether (15 mL) was added diethanolamine (260 mg, 2.5

mmol). After a few minutes, a white precipitate was formed, and the reaction was allowed to continue until the starting material was completely consumed as monitored by TLC (~18 h). The precipitate was then filtered off, washed with ether, and dried to afford a white solid. The solid was dispersed in ether (20 mL), to which was added HCl (0.5 M, 16 mL). The mixture was stirred till it became homogeneous. The resulting solution was extracted with ether (10 mL x 3), and the organic portions were combined and concentrated to dryness in vacuo to give the pure product **16** as a white solid (198 mg, 85%).

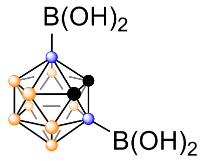

16

16: ^{1}H NMR (DMSO-d_6, 300 MHz): δ 8.11 (s, 4H) (O*H*), 4.32 (s, 2H) (cage C*H*); ^{13}C{^{1}H} NMR (DMSO-d_6, 100 MHz): δ 58.9 (cage *C*H); ^{11}B{^{1}H} NMR (DMSO-d_6, 128 MHz): δ 31.5 (s, 2B) (*B*–O), -2.0 (m, 2B), -6.9 (m, 2B), -11.3 (m, 6B) (B_{cage}H); analysis (calcd., found for $C_2H_{14}B_{12}O_4$): C (10.36, 10.24), H (6.09, 5.89).

Supplementary Discussion

Mechanistic Study (Control Experiments). NMR Tube Reactions

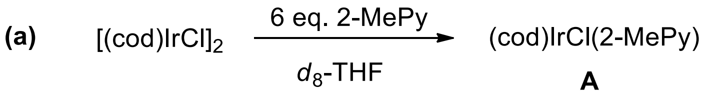

[(cod)IrCl]$_2$ (13.3 mg, 0.04 mmol) and 2-methylpyridine (2-MePy, 11.4 mg, 0.12 mmol) were mixed in d_8-THF (0.5 mL) in a J. Young valve NMR tube in glovebox. The reaction was monitored by ^{1}H NMR at room temperature. After the solvent and excess 2-MePy were removed in vacuo, a clean ^{1}H NMR spectra of (cod)IrCl(2-MePy) (**A**) was obtained in d_8-THF. Orange single crystals of complex **A** was obtained after this solution stood at room temperature for 1 day.

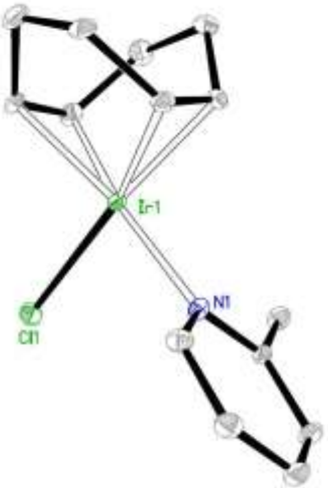

Supplementary Figure 11. Molecular structure of **A** drawn with 30% probability ellipsoids.

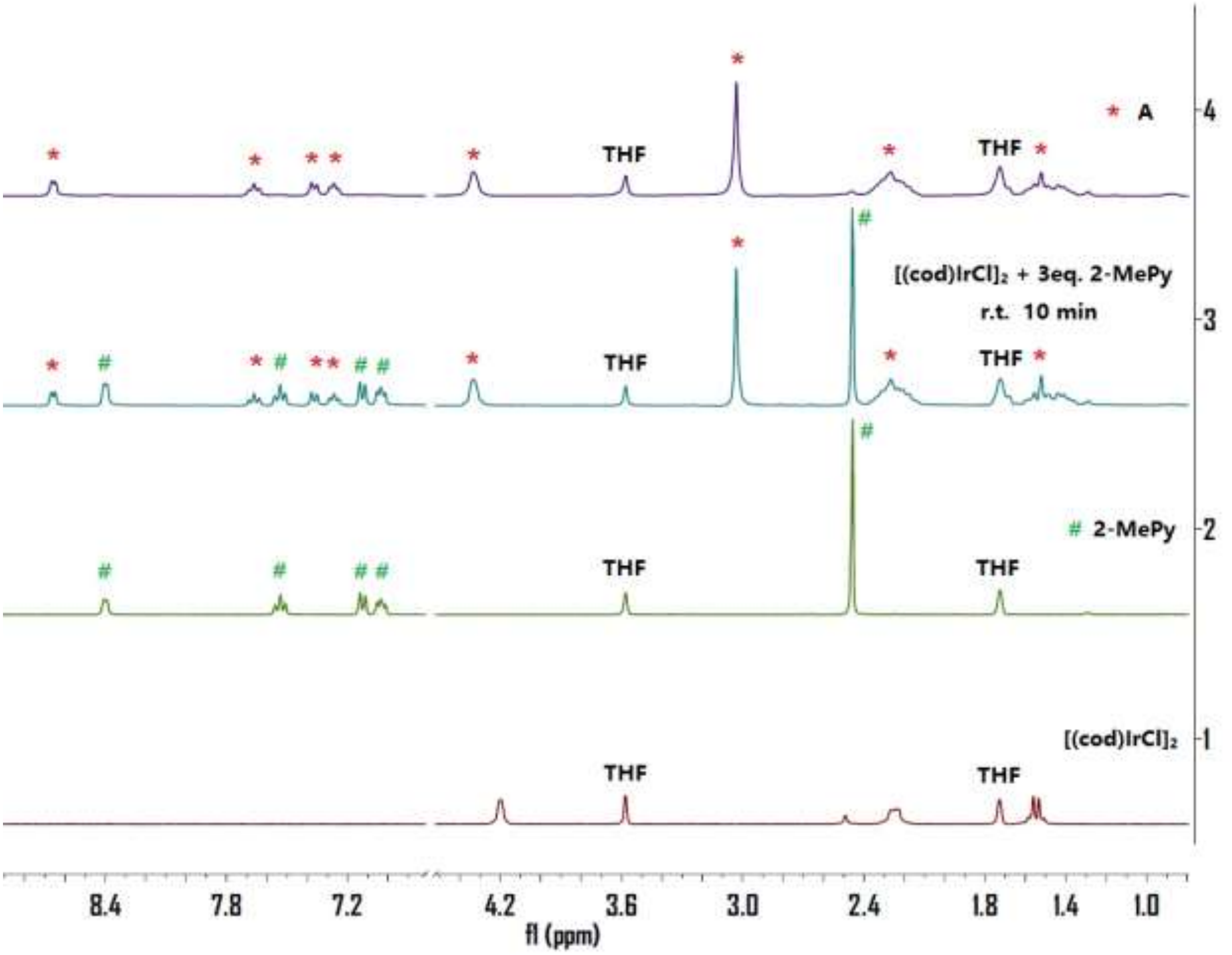

Supplementary Figure 12. ^{1}H NMR spectrum of experiment (a)

(c)

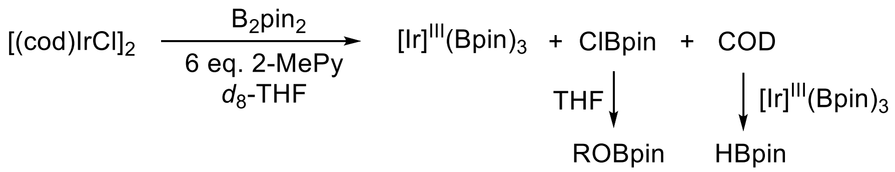

B_2pin_2 (10.1 mg, 0.04 mmol), $[(cod)IrCl]_2$ (13.3 mg, 0.04 mmol), and 2-methylpyridine (11.4 mg, 0.12 mmol) were mixed in d_8-THF (0.5 mL) in a J. Young valve NMR tube. The tube was closed and heated at 110 ^{o}C (bath temperature). The reaction was monitored by ^{11}B NMR.

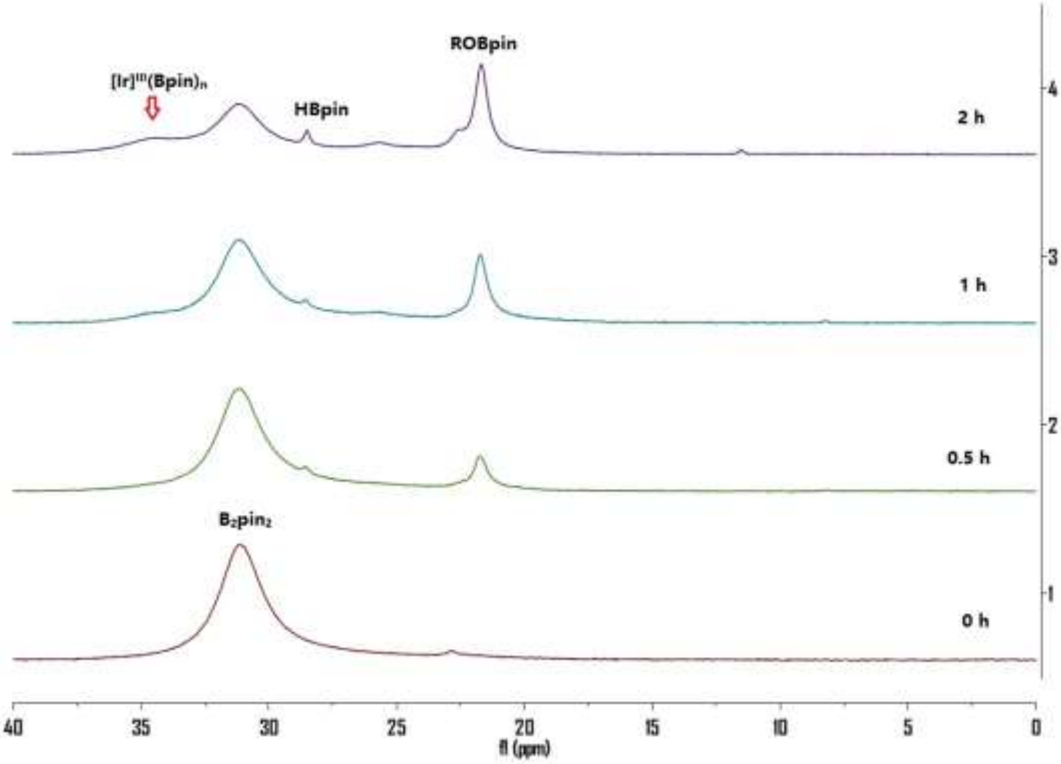

Supplementary Figure 13. Time-dependent $^{11}B\{^{1}H\}$ NMR spectra of experiment (c)

(c1)

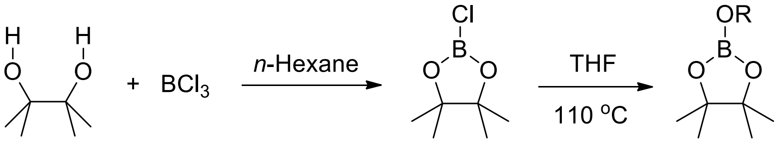

To a *n*-hexane solution (10 mL) of dry pinacol (60 mg, 0.50 mmol) was added dropwise BCl_3 solution (0.75 mL, 1 M in hexane, 0.75 mmol) via syringe at 0 ℃. The reaction mixture was stirred for 1 h at room temperature. The solution was condensed under vacuum to about 0.5 mL giving a

crude ClBpin solution.[11] The residue was dissolved in THF, which was monitored by ^{11}B NMR. The signal at 22 ppm corresponding to ROBpin resulted from the reaction of ClBpin with THF is in accordance with that observed in experiment (c).

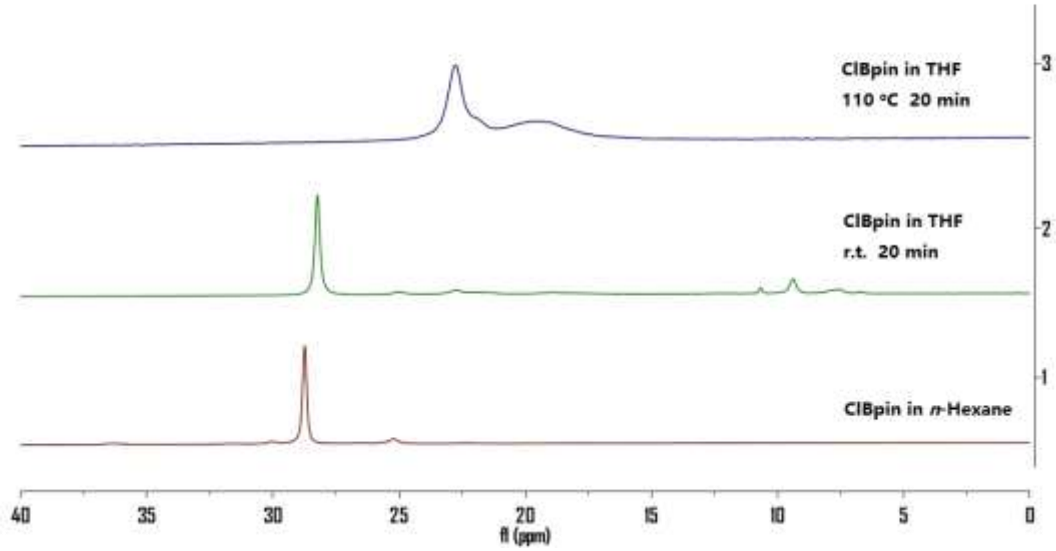

Supplementary Figure 14. Time-dependent ^{11}B{^{1}H} NMR spectra of reaction (c1)

(e)

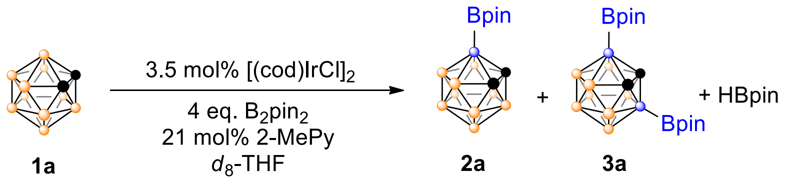

The reaction was conducted under standard conditions (4 equiv B_2pin_2, 3.5 mol% $[(cod)IrCl]_2$, 21 mol% 2-methylpyridine) in d_8-THF (0.5 mL) in a J. Young valve NMR tube and monitored by ^{1}H and ^{11}B NMR.

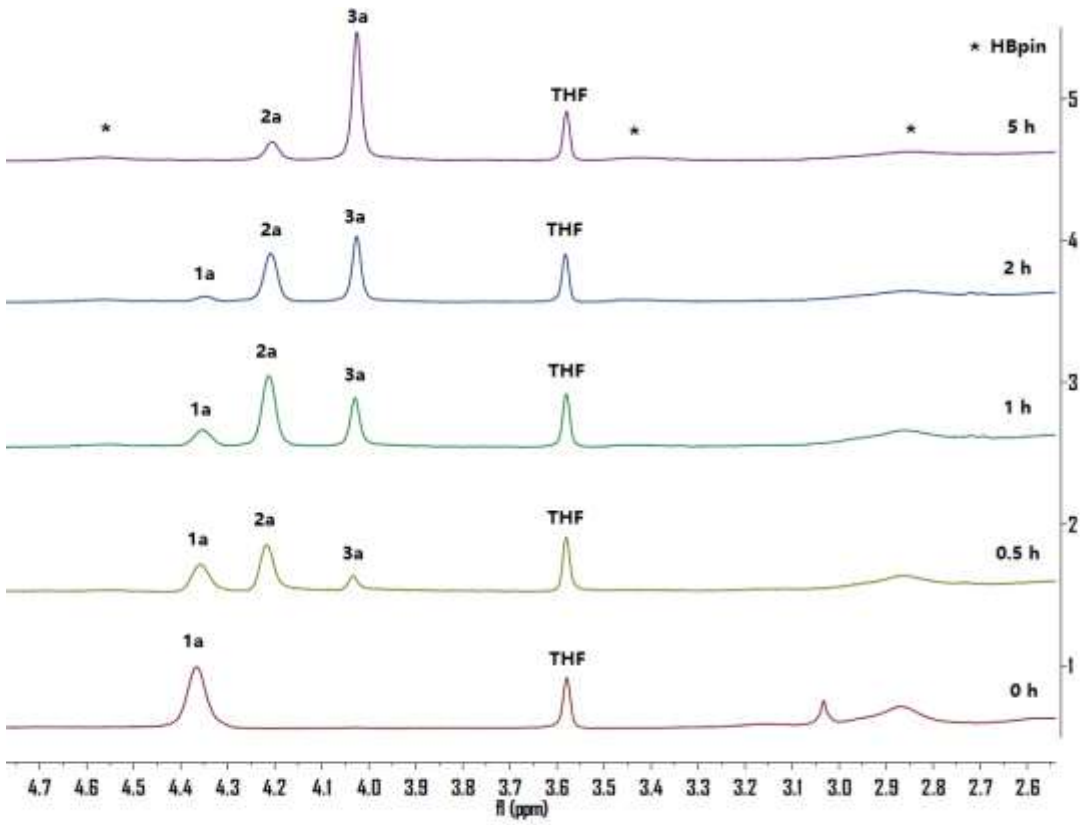

Supplementary Figure 15. Time-dependent ^{1}H NMR spectra of experiment (e)

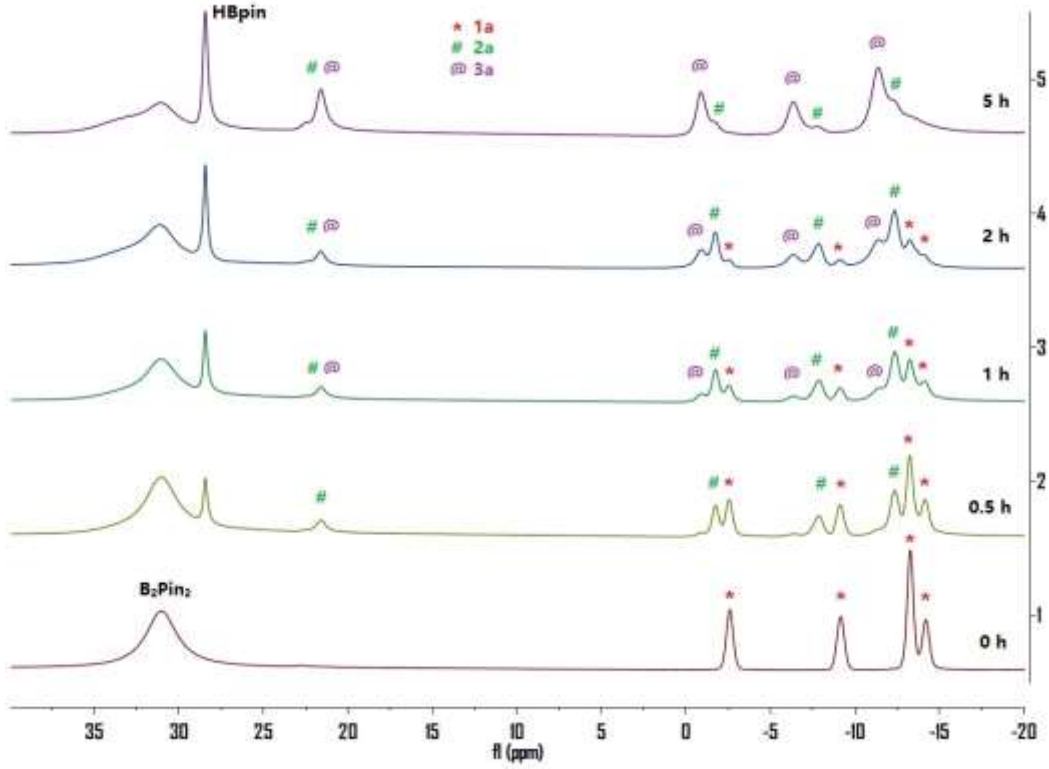

Supplementary Figure 16. Time-dependent $^{11}B\{^{1}H\}$ NMR spectra of experiment (e)

Borylation Reaction of 1a Catalyzed by (η^6-MesH)Ir(Bpin)$_3$

(f)

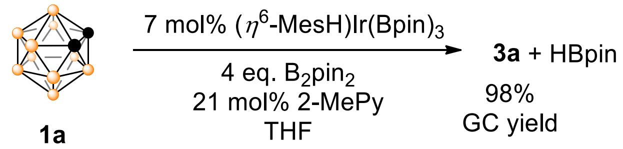

An oven-dried Schlenk flask equipped with a stir bar was charged with *o*-carborane **1a** (14.4 mg, 0.1 mmol), B_2pin_2 (101.6 mg, 0.4 mmol), (η^6-MesH)Ir(Bpin)$_3$ (4.9 mg, 0.007 mmol), and 2-methylpyridine (2.0 mg, 0.021 mmol), followed by dry THF (1 mL). The flask was closed under an atmosphere of nitrogen and stirred at 110 ^{o}C (bath temperature) for 5 h. The reaction was then quenched with wet hexane. The hexane solution was subjected to GC-MS analyses, showing that **3a** was formed in 98% GC yield.

X-ray Structure Determination. All data were collected at 293K on a Bruker SMART 1000 CCD diffractometer using Mo-Kα radiation. An empirical absorption correction was applied using the SADABS program.[12] All structures were solved by direct methods and subsequent Fourier difference techniques and refined anisotropically for all non-hydrogen atoms by full-matrix least squares calculations on F^2 using the SHELXTL program package.[13] All hydrogen atoms were geometrically fixed using the riding model. Crystal data and details of data collection and structure refinements are given in Table S5.

CCDC 1500326-1500335 (**2a**, **2p**, **2q**, **3a**, **3l**, **5**, **7c**, **8a**, **15** and complexes **A**) contains the supplementary crystallographic data for this paper. These data can be obtained free of charge from The Cambridge Crystallographic Data Centre via www.ccdc.cam.ac.uk/data_request/cif.

Supplementary Table 5. Crystal Data and Summary of Data Collection and Refinement.

compound	**3a**	**3l**	**2p**	**2q**	**2a**
formula	$C_{14}H_{34}B_{12}O_4$	$C_{14}H_{32}B_{12}I_2O_4$	$C_{22}H_{33}B_{11}O_2$	$C_8H_{21}B_{11}I_2O_2$	$C_8H_{23}B_{11}O_2$
crystal size (mm)	0.25x0.22x0.15	0.25x0.15x0.05	0.15x0.1x0.02	0.12x0.1x0.08	0.28x0.25x0.20
fw	396.13	647.91	448.39	521.96	270.17
crystal system	triclinic	monoclinic	triclinic	monoclinic	monoclinic
space group	P-1	$P2_1$/c	P-1	$P2_1$/c	$P2_1$/n
a, Å	11.531(6)	11.871(1)	9.444(2)	11.516(1)	6.734(2)
b, Å	12.628(7)	13.081(1)	11.410(3)	13.637(1)	20.302(5)
c, Å	18.211(10)	17.992(1)	13.233(3)	12.824(1)	12.248(3)
α, deg	91.95(1)	90	68.38(1)	90	90
β, deg	91.00(1)	101.31(10)	80.64(1)	102.71(1)	93.54(1)
γ, deg	116.46(1)	90	73.26(1)	90	90
V, Å^3	2371(2)	2739.6(3)	1266.8(5)	1964.6(3)	1671.4(7)
Z	4	4	2	4	4
D_{calcd}, Mg/m^3	1.110	1.571	1.176	1.765	1.074
radiation (λ) Å	0.71073	0.71073	0.71073	0.71073	0.71073
2θ range, deg	3.9 to 52.0	3.5 to 61.2	4.0 to 60.0	3.6 to 61.1	4.0 to 55.0
μ, mm^{-1}	0.066	2.314	0.065	3.197	0.058
F(000)	840	1256	472	984	568
no. of obsd reflns	9252	8418	7243	6019	3709
no. of params refnd	558	297	320	212	194
goodness of fit	1.026	1.027	1.045	1.034	0.955
R1	0.0858	0.0441	0.0716	0.0249	0.0881
wR2	0.1924	0.1121	0.1691	0.0548	0.2300

compound	**5**	**7c**	**8a**	**15**	**A**
formula	$C_8H_{23}B_{11}O_2$	$C_{14}H_{35}B_{11}I_2O_2Si$	$C_8H_{23}B_{11}O_2$	$C_{18}H_{30}B_{10}N_6O_8$	$C_{14}H_{19}ClIrN$
crystal size (mm)	0.25x0.2x0.18	0.18x0.165x0.12	0.32x0.28x0.25	0.25 x 0.22 x 0.2	0.15x0.1x0.05
fw	270.17	636.22	270.17	566.58	428.95
crystal system	monoclinic	orthorhombic	monoclinic	triclinic	monoclinic
space group	$P2_1/c$	*P*bca	$P2_1/n$	*P*-1	$P2_1/c$
a, Å	8.374(3)	12.192(2)	6.699(1)	7.088(1)	10.507(1)
b, Å	18.502(7)	14.089(2)	20.308(2)	13.346(1)	10.840(1)
c, Å	10.724(4)	33.150(5)	12.120(1)	15.578(2)	12.435(1)
α, deg	90	90	90	77.22(1)	90
β, deg	91.29(1)	90	93.87(1)	79.09(1)	107.49(1)
γ, deg	90	90	90	79.59(1)	90
V, Å^3	1661.1(11)	5694.0(15)	1645.1(3)	1396.2(3)	1350.6(2)
Z	4	8	4	2	4
D_{calcd}, Mg/m^3	1.080	1.484	1.091	1.348	2.110
radiation (λ) Å	0.71073	0.71073	0.71073	0.71073	0.71073
2θ range, deg	2.2 to 55.4	4.6 to 39.2	5.2 to 58.5	3.2 to 61.2	4.1 to 613
μ, mm^{-1}	0.059	2.261	0.059	0.096	10.061
F(000)	568	2480	568	588	816
no. of obsd reflns	3849	6601	5052	8460	4152
no. of params refnd	194	280	244	383	155
goodness of fit	1.007	1.169	1.050	1.044	1.024
R1	0.0692	0.0765	0.0770	0.0523	0.0242
wR2	0.1721	0.2001	0.2009	0.1254	0.0559

Supplementary References

(1) Andrews, J. S., Zayas, J. & Jones, M. Jr. 9-Iodo-*o*-carborane. *Inorg. Chem.* **24**, 3715–3716 (1985).

(2) (a) Li, J., Logan, C. F. & Jones, M. Jr. Simple syntheses and alkylation reactions of 3-iodo-*o*-carborane and 9,12-diiodo-*o*-carborane. *Inorg. Chem.* **30**, 4866–4868 (1991).

(3) Zheng, Z., Jiang, W., Zinn, A. A., Knobler, C. B. & Hawthorne, M. F. Facile electrophilic iodination of icosahedral carboranes. Synthesis of carborane derivatives with boron-carbon bonds via the palladium-catalyzed reaction of diiodocarboranes with grignard reagents. *Inorg. Chem.* 34, 2095–2100 (1995).

(4) Spokoyny, A. M., Li, T. C., Farha, O. K., Machan, C. W., She, C., Stern, C. L., Marks, T. J., Hupp, J. T. & Mirkin, C. A. Electronic tuning of nickel-based bis(dicarbollide) redox shuttles in dye-sensitized solar cells. *Angew. Chem. Int. Ed.* **49**, 5339–5343 (2010).

(5) Kabytaev, K. Z., Safronov, A. V., Jalisatgi, S. S. & Hawthorne, M. F. Synthesis and reactions of B-vinylcarboranes. *J. Organomet. Chem.* **749**, 106–108 (2014).

(6) Bhaskar, M. R., Carolyn, B. K. & Hawthorne, M. F. Synthesis and structural characterization of symmetrical *closo*-4,7-I_2-1,2-$C_2B_{10}H_{10}$ and [$(CH_3)_3$NH][*nido*-2,4-I_2-7,8-$C_2B_9H_{10}$]. *Inorg. Chem.* **45**, 336–340 (2006).

(7) Quan, Y. & Xie, Z. Iridium catalyzed regioselective cage boron alkenylation of *o*-carboranes via direct cage B–H activation. *J. Am. Chem. Soc.* **136**, 15513–15516 (2014).

(8) Zhao, D., Zhang, J. J. & Xie, Z. 1,3-Dehydro-*o*-carborane: generation and reaction with arenes. *Angew. Chem. Int. Ed.* **53**, 8488–8491 (2014).

(9) Gomezl, F. A. & Hawthorne, M. F. A simple route to C-monosubstituted carborane derivatives. *J. Org. Chem*. **57**, 1384–1390 (1992).

(10) Li, Q., Liskey, C. W. & Hartwig, J. F. Regioselective borylation of the C–H bonds in alkylamines and alkyl ethers. Observation and origin of high reactivity of primary C–H bonds beta to nitrogen and oxygen. *J. Am. Chem. Soc*. **136**, 8755–8765 (2014).

(11) Waltz, K. M. & Hartwig, J. F. Functionalization of alkanes by isolated transition metal boryl complexes. *J. Am. Chem. Soc*. **122**, 11358–11369 (2000).

(12) Sheldrick, G. M. S*ADABS: Program for Empirical Absorption Correction of Area Detector Data*. (University of Göttingen: Germany, 1996).

(13) Sheldrick, G. M. *SHELXTL 5.10 for Windows NT: Structure Determination Software Programs*. (Bruker Analytical X-ray Systems, Inc., Madison, Wisconsin, USA, 1997).

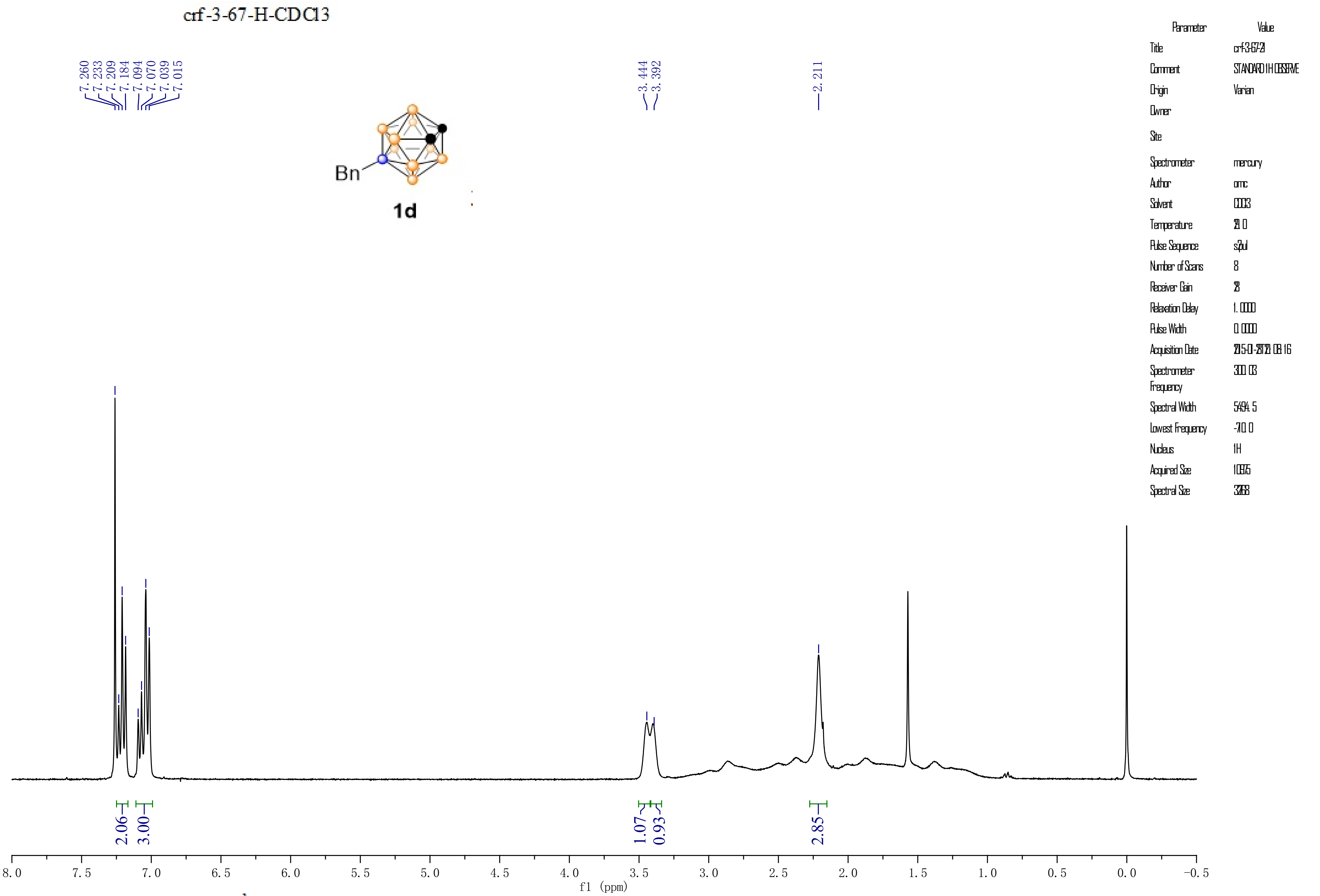

Supplementary Figure 17. ^{1}H NMR Spectrum of **1d**.

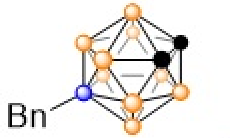

1d

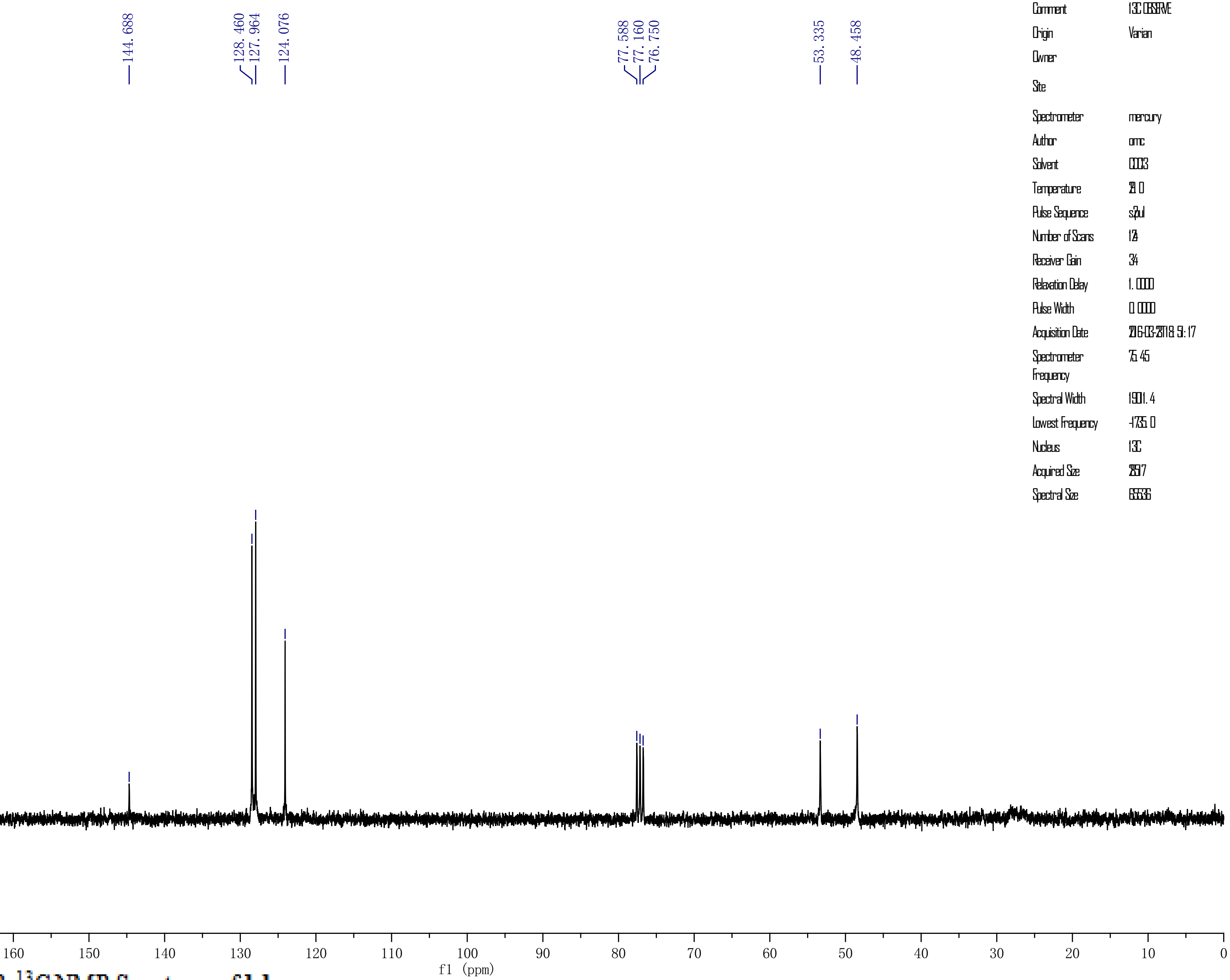

Parameter	Value
Title	crf-9-Bn-CB-C
Comment	13C OBSERVE
Origin	Varian
Owner	
Site	
Spectrometer	mercury
Author	omc
Solvent	CDCl3
Temperature	21.0
Pulse Sequence	s2pul
Number of Scans	124
Receiver Gain	34
Relaxation Delay	1.0000
Pulse Width	0.0000
Acquisition Date	2016-03-28T18:51:17
Spectrometer Frequency	75.45
Spectral Width	19011.4
Lowest Frequency	-1735.0
Nucleus	13C
Acquired Size	28517
Spectral Size	65536

Supplementary Figure 18. ^{13}C NMR Spectrum of **1d**.

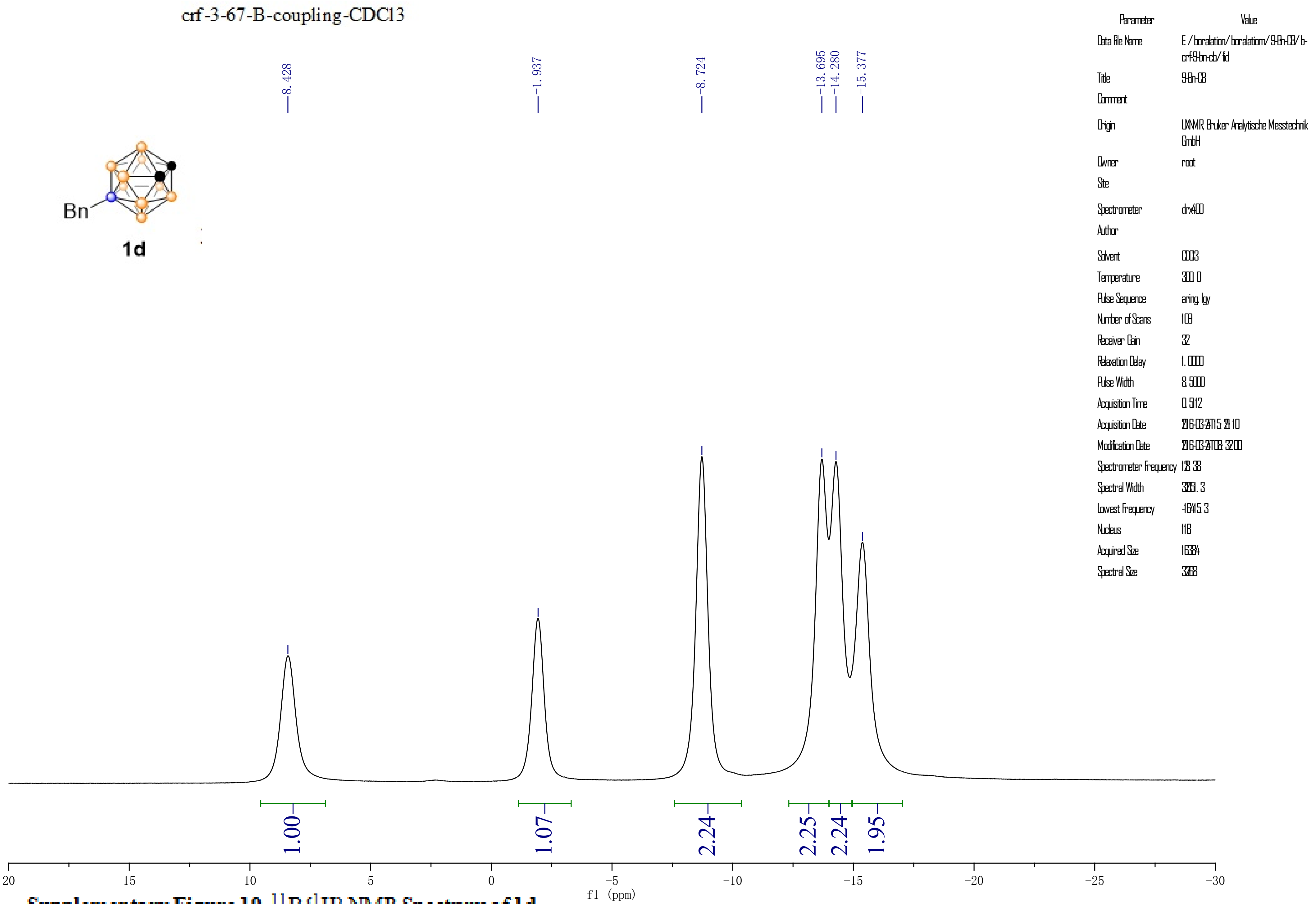

Supplementary Figure 19. $^{11}B\{^{1}H\}$ NMR Spectrum of **1d**.

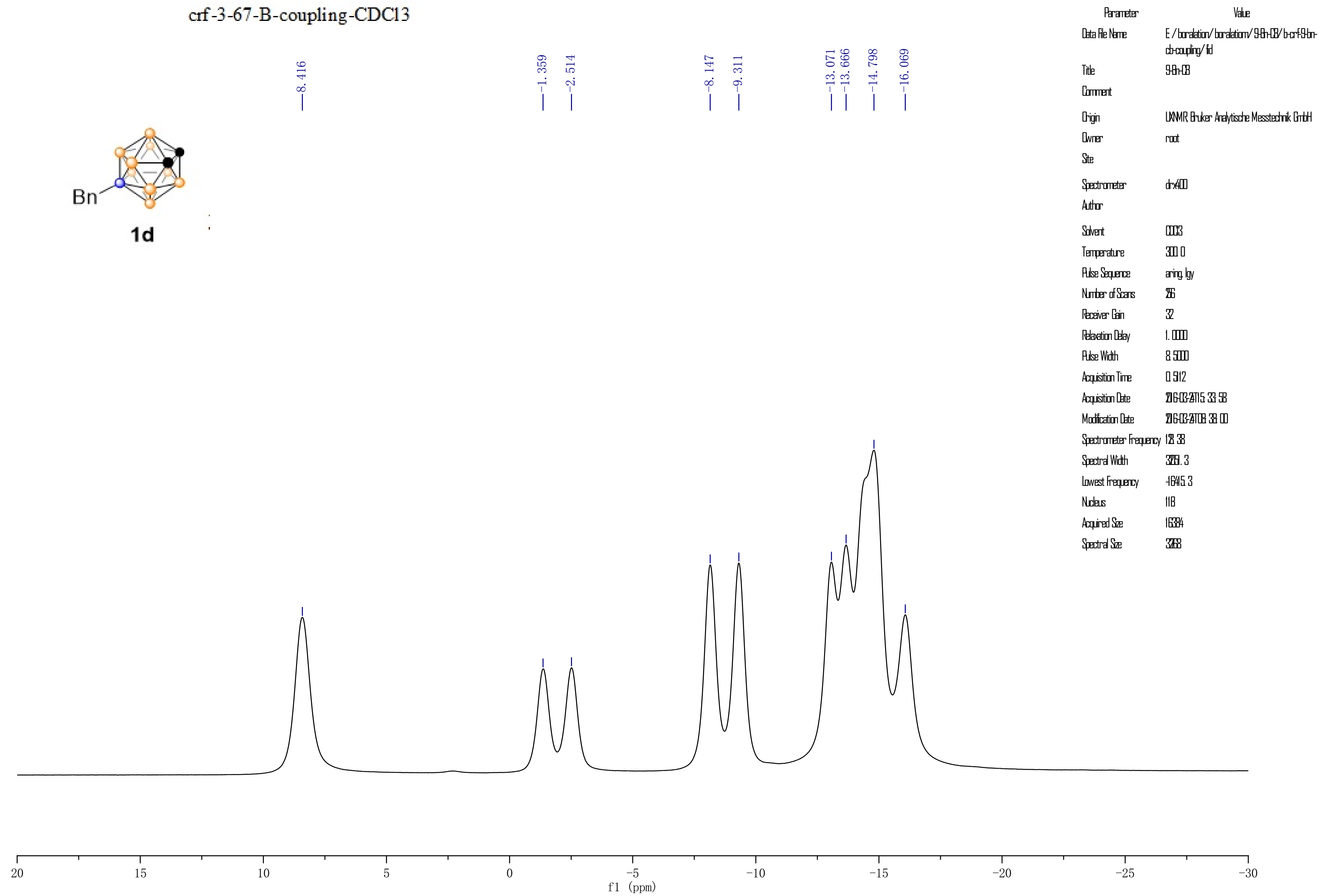

Supplementary Figure 20. ^{11}B NMR Spectrum of **1d**.

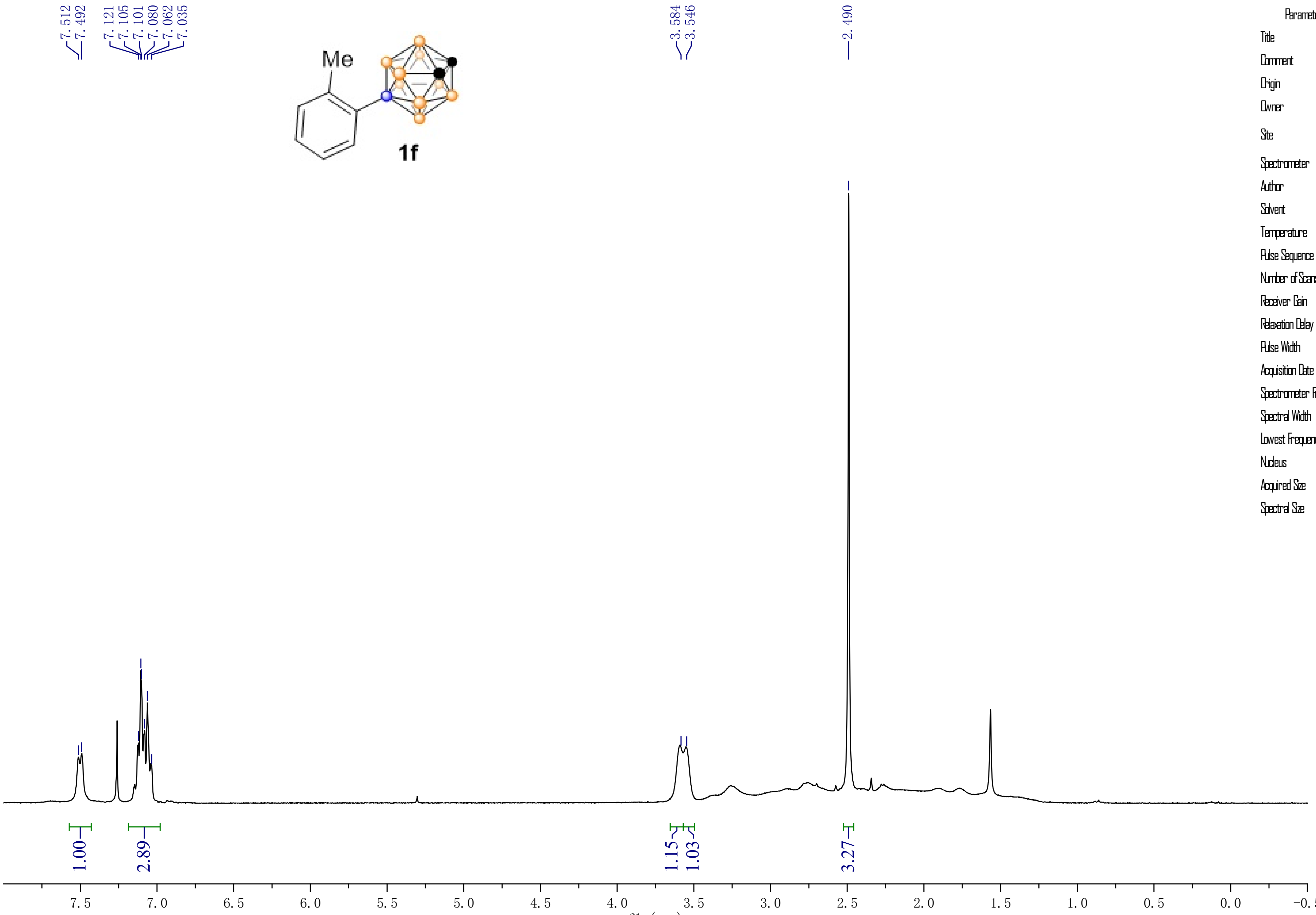

Parameter	Value
Title	crf-4-41
Comment	STANDARD 1H OBSERVE
Origin	Varian
Owner	
Site	
Spectrometer	mercury
Author	omc
Solvent	CDCl3
Temperature	[illegible]
Pulse Sequence	s2pul
Number of Scans	8
Receiver Gain	28
Relaxation Delay	1.0000
Pulse Width	0.0000
Acquisition Date	2015-04-17T15:59:26
Spectrometer Frequency	300.03
Spectral Width	5494.5
Lowest Frequency	-708.9
Nucleus	1H
Acquired Size	10975
Spectral Size	32768

Supplementary Figure 21. ^{1}H NMR Spectrum of **1f**.

crf-4-41-C-CDCl3

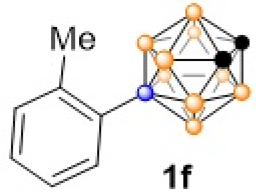

1f

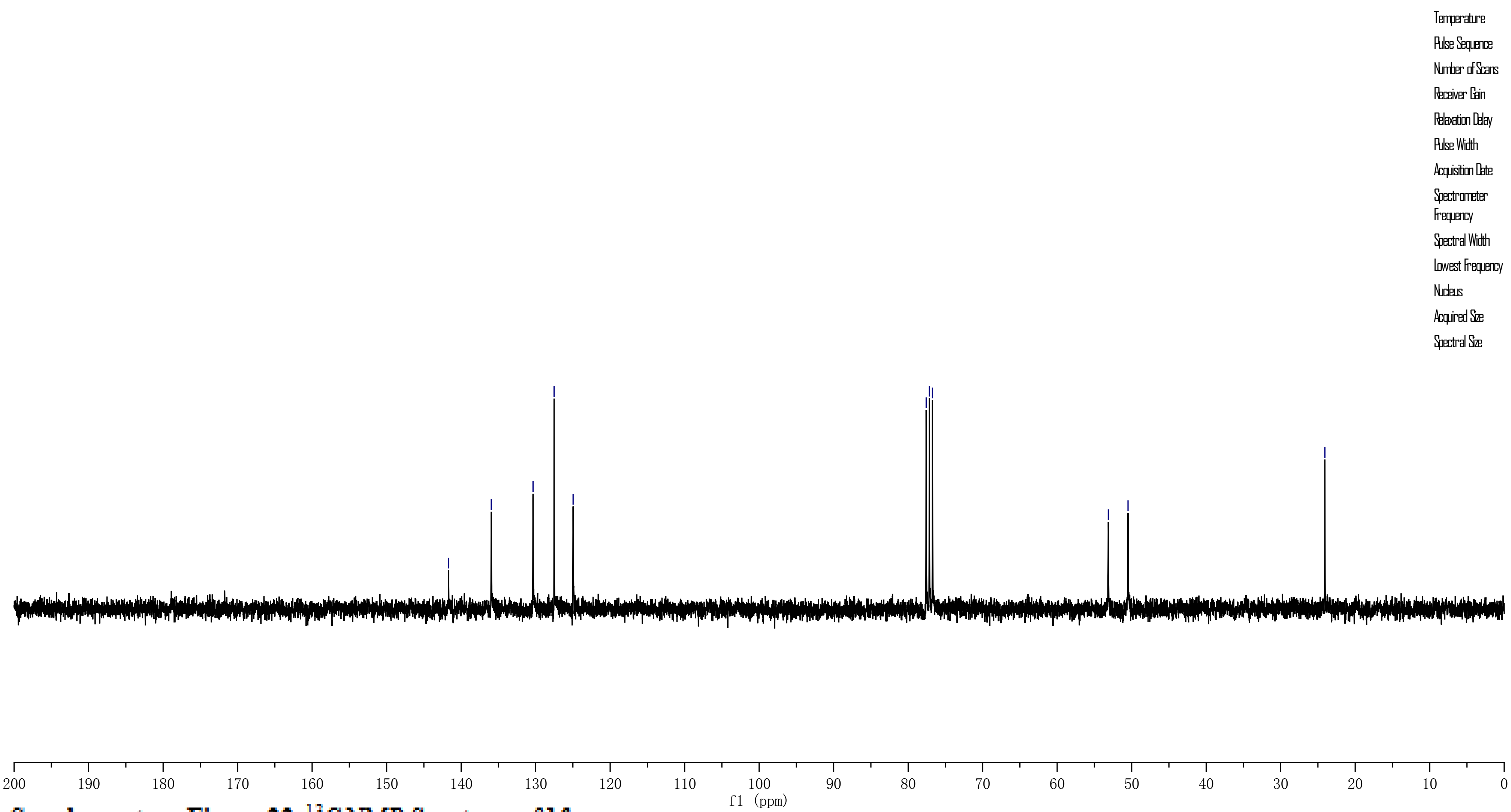

Parameter	Value
Title	crf-3-1g-C
Comment	13C OBSERVE
Origin	Varian
Owner	
Site	
Spectrometer	mercury
Author	omc
Solvent	CDCl3
Temperature	21.0
Pulse Sequence	s2pul
Number of Scans	120
Receiver Gain	34
Relaxation Delay	1.0000
Pulse Width	0.0000
Acquisition Date	2016-03-27T12:50:41
Spectrometer Frequency	75.45
Spectral Width	19011.4
Lowest Frequency	-1735.0
Nucleus	13C
Acquired Size	28517
Spectral Size	65536

Supplementary Figure 22. ^{13}C NMR Spectrum of **1f**.

crf-4-41-B-decoupling-CDCl3

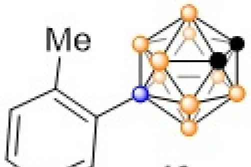

1f

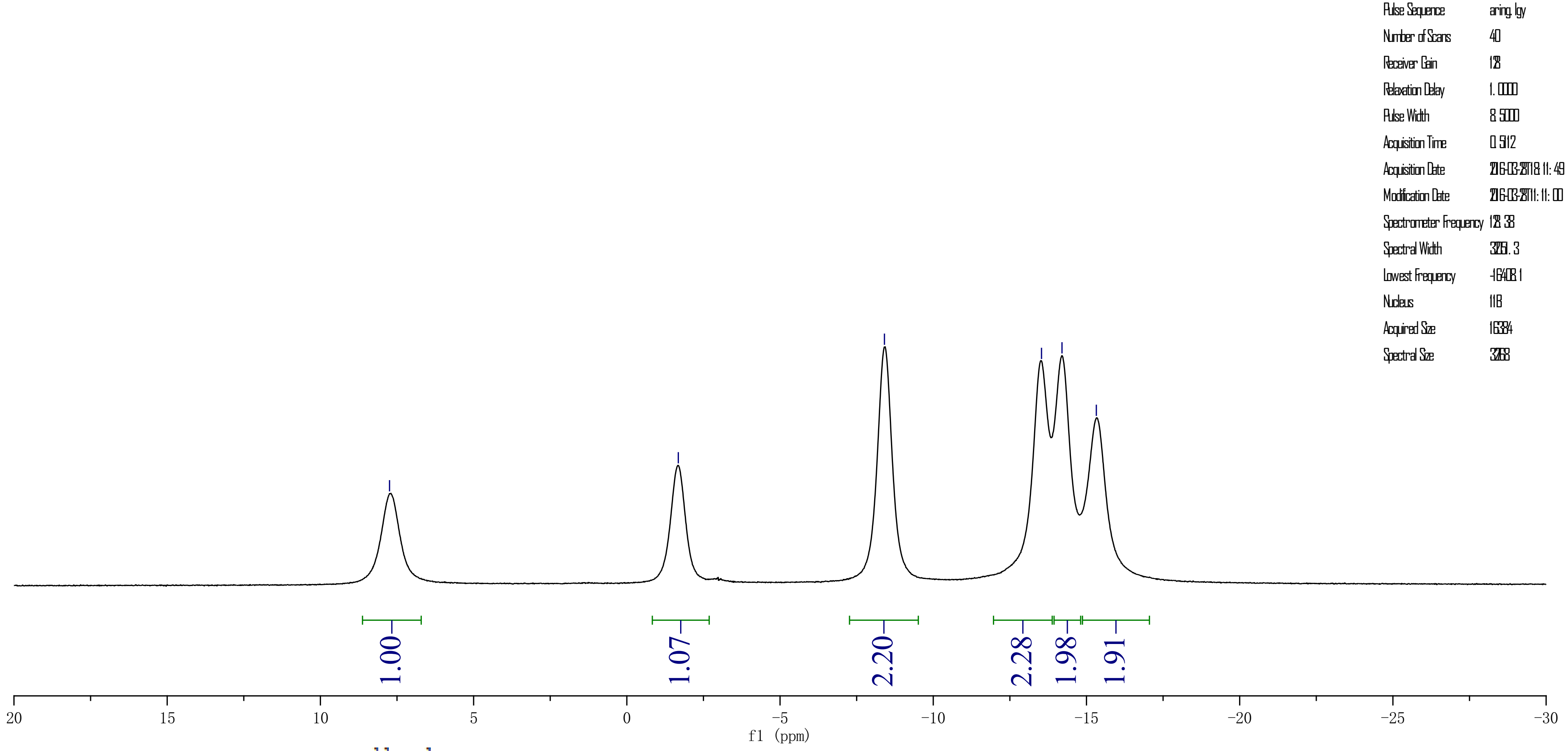

Parameter	Value
Data File Name	E:/boralation/boralatiom/9(2MePh_-CB/b-crf3-1g/fid
Title	9(2MePh_-CB
Comment	
Origin	UXNMR, Bruker Analytische Messtechnik GmbH
Owner	root
Site	
Spectrometer	drx400
Author	
Solvent	CDCl3
Temperature	300.0
Pulse Sequence	aring.lgy
Number of Scans	40
Receiver Gain	128
Relaxation Delay	1.0000
Pulse Width	8.5000
Acquisition Time	0.5112
Acquisition Date	2016-03-28T18:11:49
Modification Date	2016-03-28T11:11:00
Spectrometer Frequency	128.38
Spectral Width	32051.3
Lowest Frequency	-16408.1
Nucleus	11B
Acquired Size	16384
Spectral Size	32768

Supplementary Figure 23. $^{11}B\{^1H\}$ NMR Spectrum of **1f**.

crf-4-41-B-coupling-CDCl3

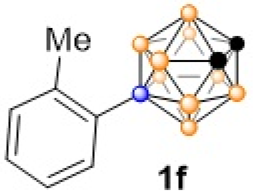

1f

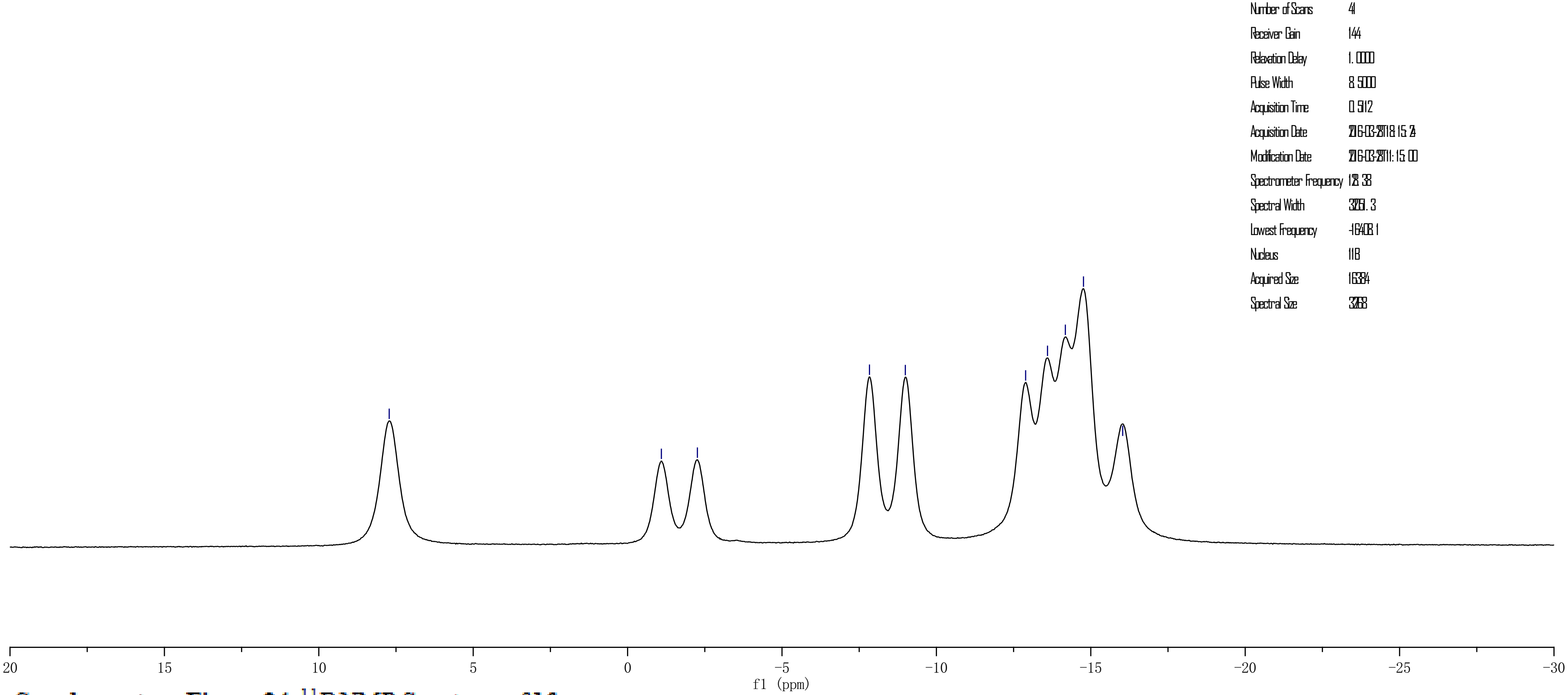

Parameter	Value
Data File Name	E:/boralation/boralatiom/9(2MePh)-CB/b-crf3-41g-coupling/fid
Title	9(2MePh)-CB
Comment	
Origin	UXNMR, Bruker Analytische Messtechnik GmbH
Owner	root
Site	
Spectrometer	drx400
Author	
Solvent	DMSO
Temperature	300.0
Pulse Sequence	aring.lgy
Number of Scans	41
Receiver Gain	144
Relaxation Delay	1.0000
Pulse Width	8.5000
Acquisition Time	0.5112
Acquisition Date	2016-03-28T18:15:24
Modification Date	2016-03-28T11:15:00
Spectrometer Frequency	128.38
Spectral Width	32051.3
Lowest Frequency	-16408.1
Nucleus	11B
Acquired Size	16384
Spectral Size	32768

Supplementary Figure 24. ^{11}B NMR Spectrum of **1f**.

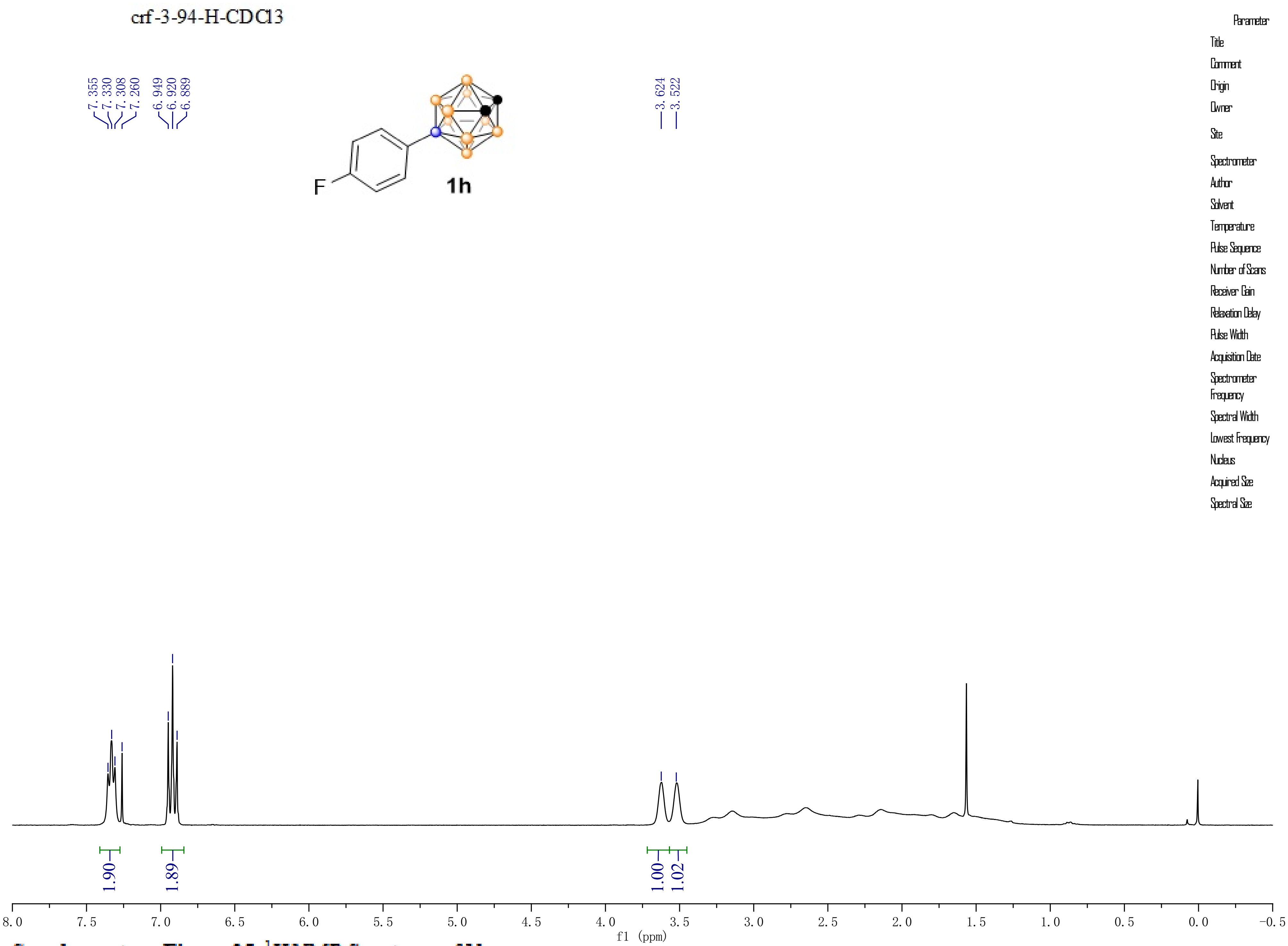

Parameter	Value
Title	crf-3-1h-H-0408
Comment	STANDARD 1H OBSERVE
Origin	Varian
Owner	
Site	
Spectrometer	mercury
Author	omc
Solvent	CDCl3
Temperature	21.0
Pulse Sequence	s2pul
Number of Scans	8
Receiver Gain	28
Relaxation Delay	1.0000
Pulse Width	0.0000
Acquisition Date	2016-04-08T12:55:05
Spectrometer Frequency	300.03
Spectral Width	5494.5
Lowest Frequency	-709.9
Nucleus	1H
Acquired Size	10989
Spectral Size	32768

Supplementary Figure 25. ^{1}H NMR Spectrum of **1h**.

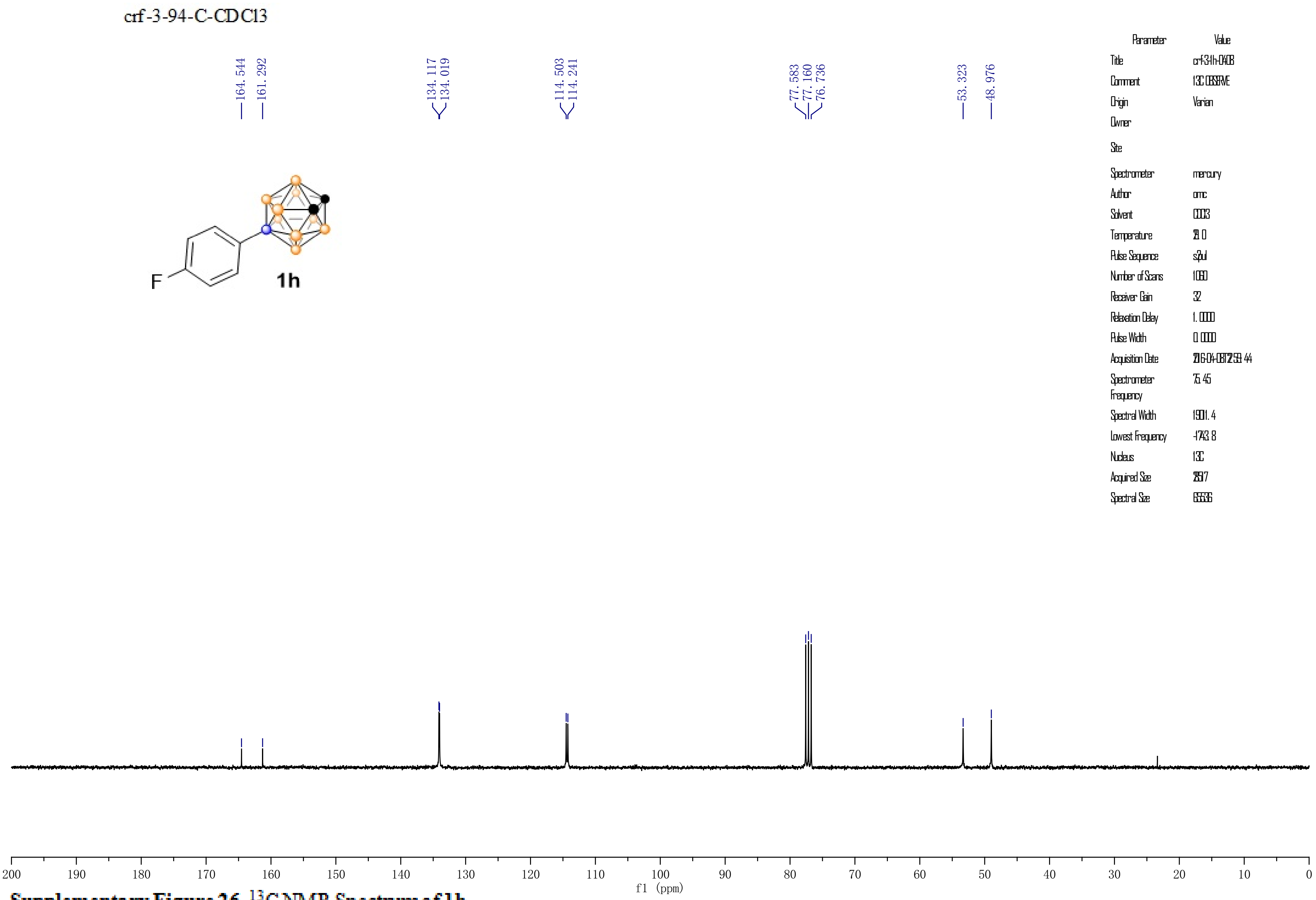

Supplementary Figure 26. ^{13}C NMR Spectrum of **1h**.

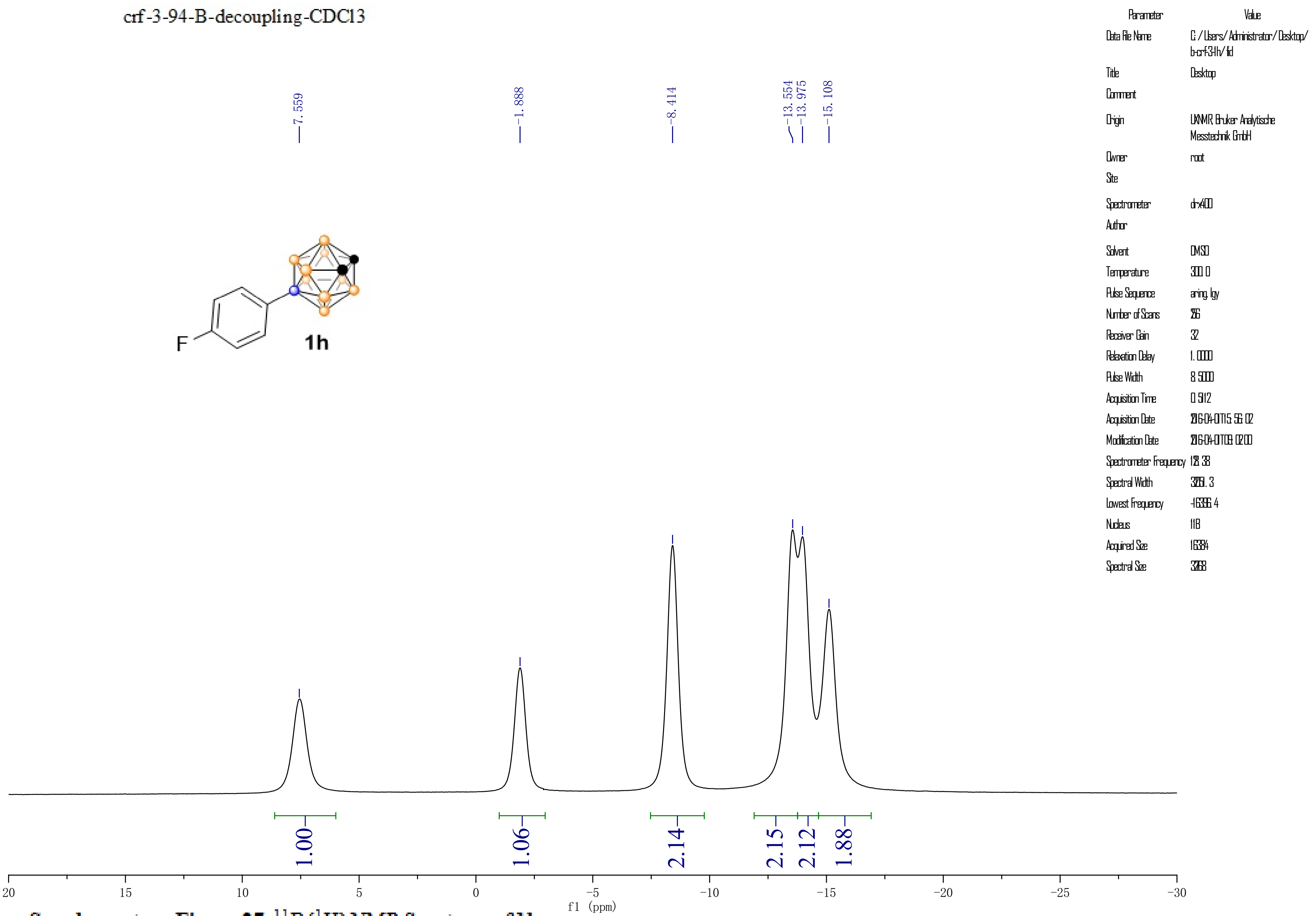

Supplementary Figure 27. $^{11}B\{^{1}H\}$ NMR Spectrum of **1h**.

crf-3-94-B-coupling-CDCl3

Parameter	Value
Data File Name	C:/Users/Administrator/Desktop/b-crf-3-1h-coupling (1)/fid
Title	Desktop
Comment	
Origin	UXNMR, Bruker Analytische Messtechnik GmbH
Owner	root
Site	
Spectrometer	drx400
Author	
Solvent	DMSO
Temperature	300.0
Pulse Sequence	aring.lgy
Number of Scans	256
Receiver Gain	32
Relaxation Delay	1.0000
Pulse Width	8.5000
Acquisition Time	0.5112
Acquisition Date	2016-04-01T16:08:10
Modification Date	2016-04-01T09:14:00
Spectrometer Frequency	128.38
Spectral Width	32051.3
Lowest Frequency	-16394.6
Nucleus	11B
Acquired Size	16384
Spectral Size	32768

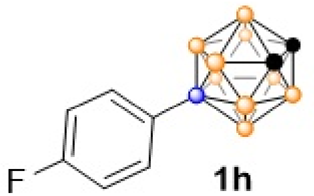

1h

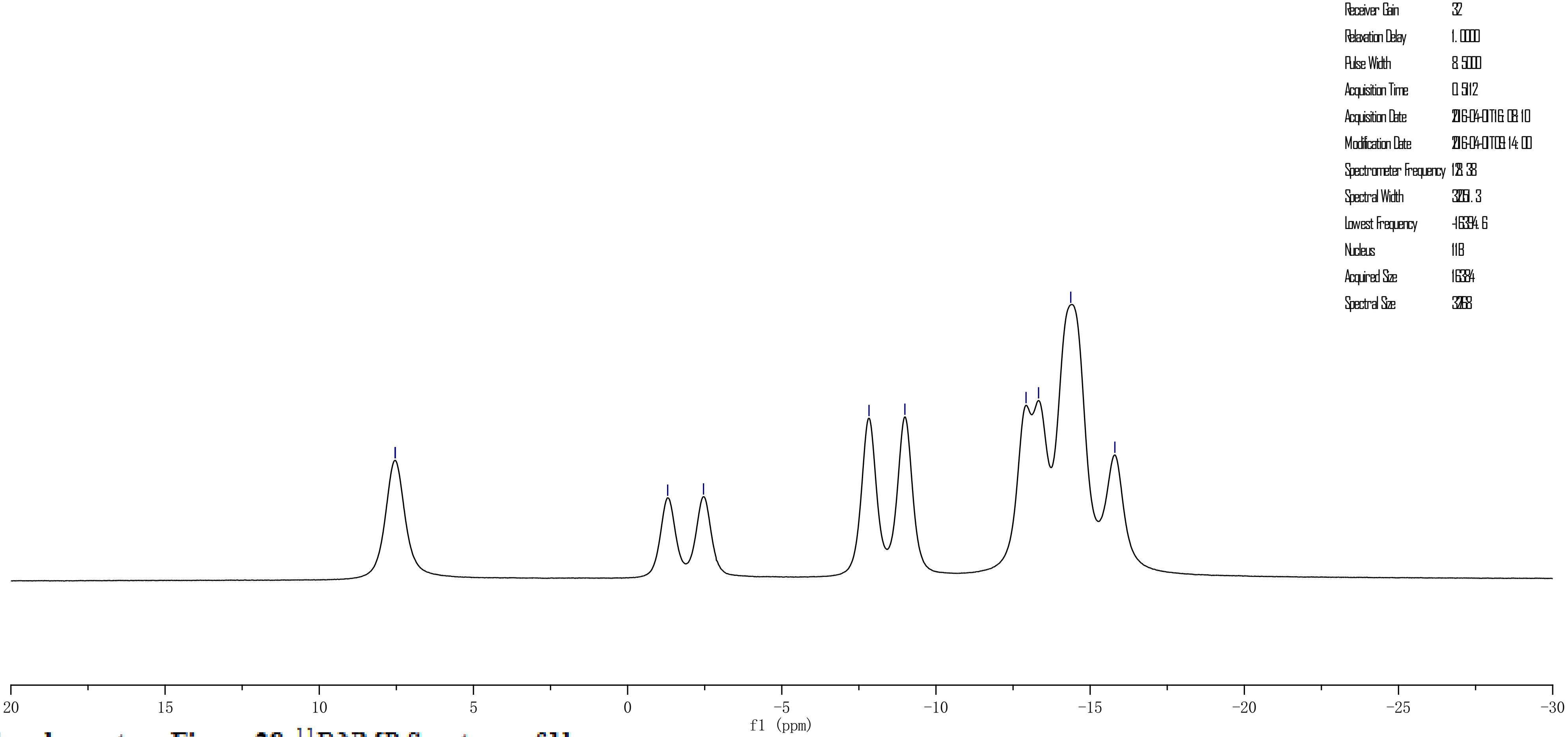

Supplementary Figure 28. ^{11}B NMR Spectrum of **1h**.

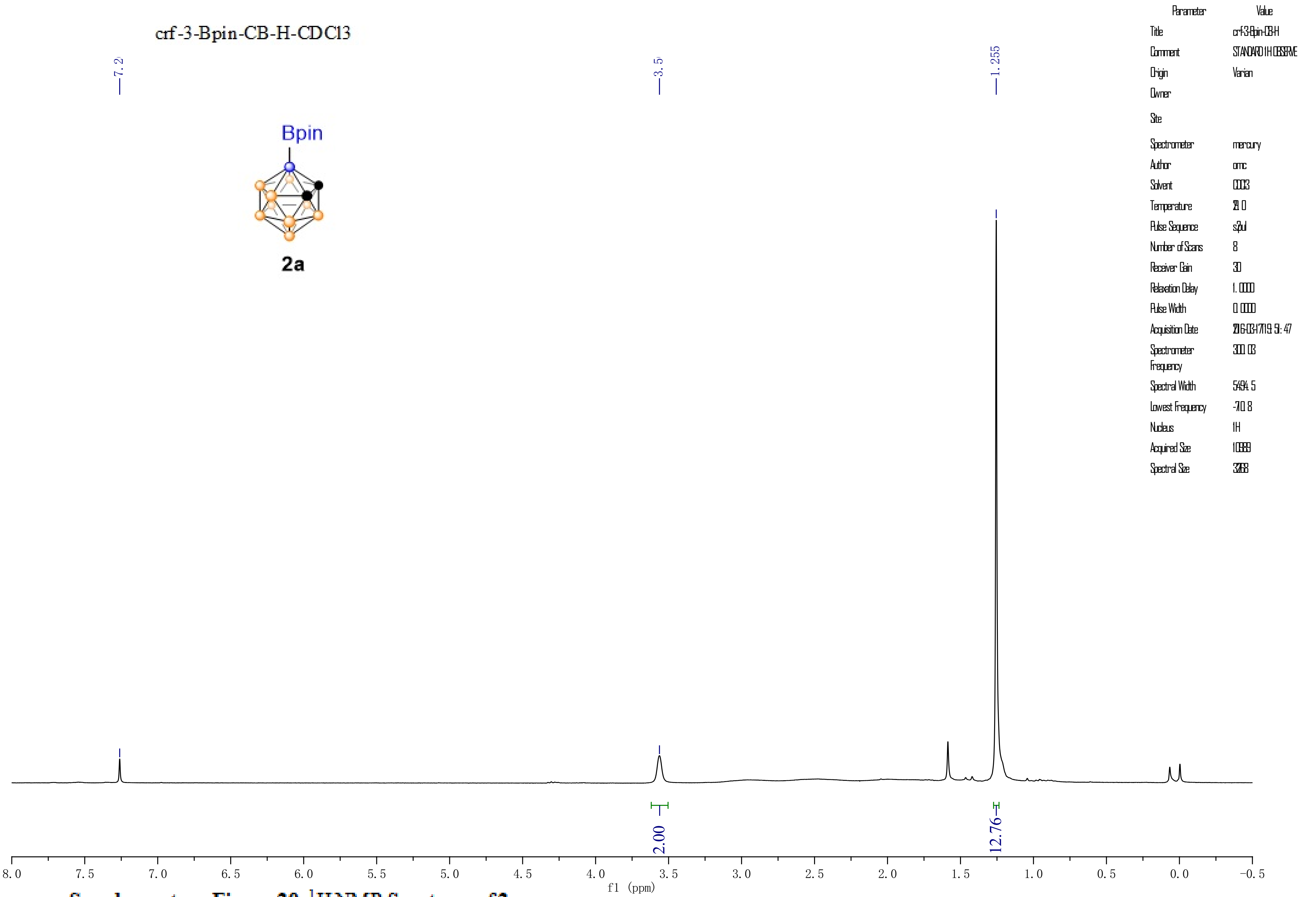

Supplementary Figure 29. ^{1}H NMR Spectrum of **2a**.

crf-3-Bpin-CB-C-CDCl3

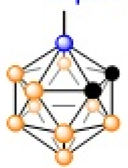

2a

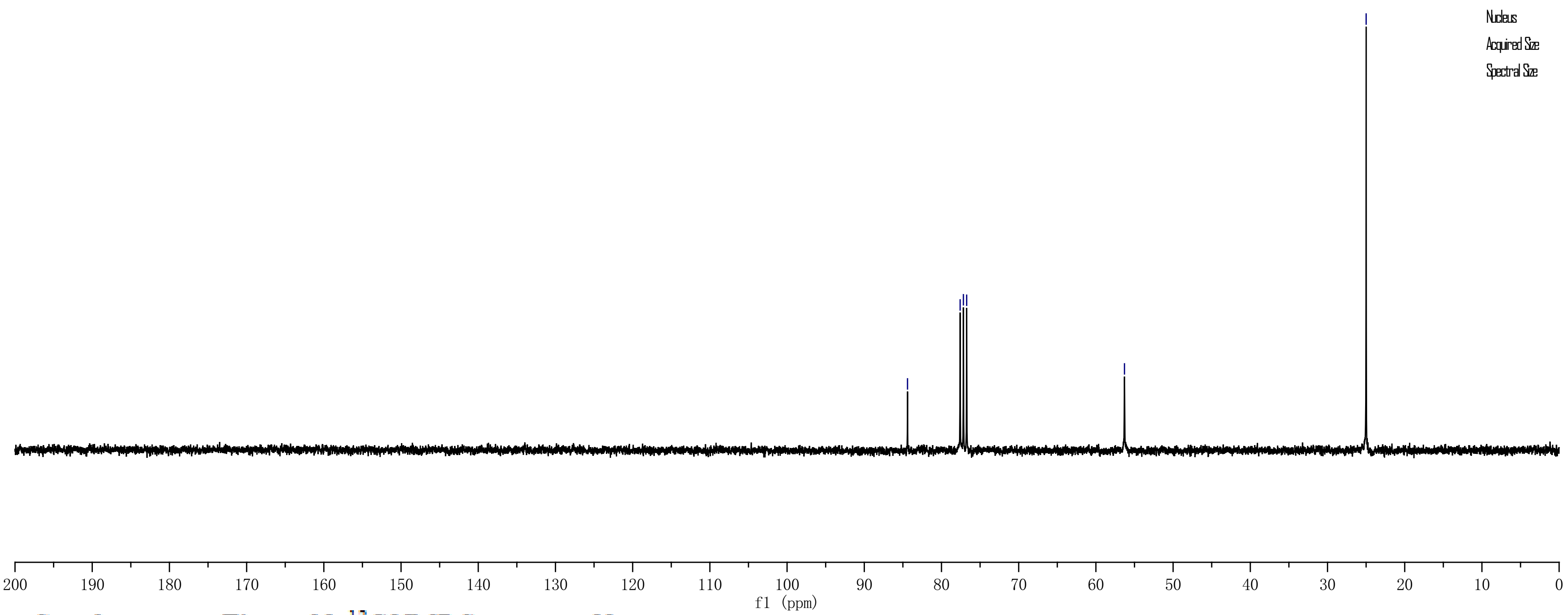

Parameter	Value
Title	crf-3-Bpin-CB-C
Comment	13C OBSERVE
Origin	Varian
Owner	
Site	
Spectrometer	mercury
Author	omc
Solvent	CDCl3
Temperature	[illegible]
Pulse Sequence	s2pul
Number of Scans	272
Receiver Gain	34
Relaxation Delay	1.0000
Pulse Width	0.0000
Acquisition Date	2016-03-17T19:58:15
Spectrometer Frequency	75.45
Spectral Width	19011.4
Lowest Frequency	-1735.0
Nucleus	13C
Acquired Size	28517
Spectral Size	65536

Supplementary Figure 30. ^{13}C NMR Spectrum of **2a**.

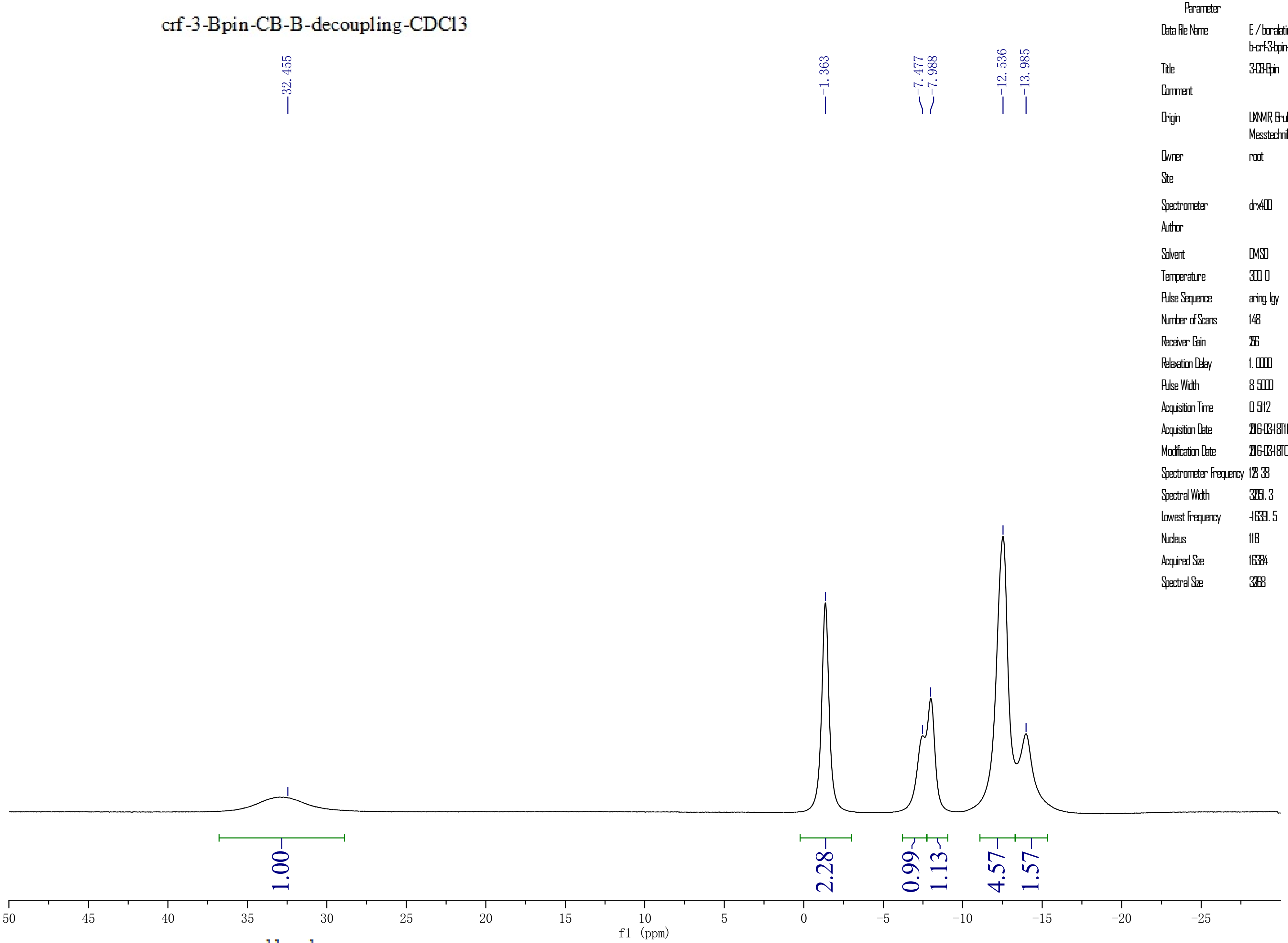

Parameter	Value
Data File Name	E:/boralation/boralatiom/3-CB-Bpin/b-crf-3-bpin-cb/fid
Title	3-CB-Bpin
Comment	
Origin	UXNMR, Bruker Analytische Messtechnik GmbH
Owner	root
Site	
Spectrometer	drx400
Author	
Solvent	DMSO
Temperature	300.0
Pulse Sequence	aring.lgy
Number of Scans	148
Receiver Gain	256
Relaxation Delay	1.0000
Pulse Width	8.5000
Acquisition Time	0.5112
Acquisition Date	2016-03-18T16:02:19
Modification Date	2016-03-18T09:02:00
Spectrometer Frequency	128.38
Spectral Width	32051.3
Lowest Frequency	-16391.5
Nucleus	11B
Acquired Size	16384
Spectral Size	32768

Supplementary Figure 31. $^{11}B\{^{1}H\}$ NMR Spectrum of **2a**.

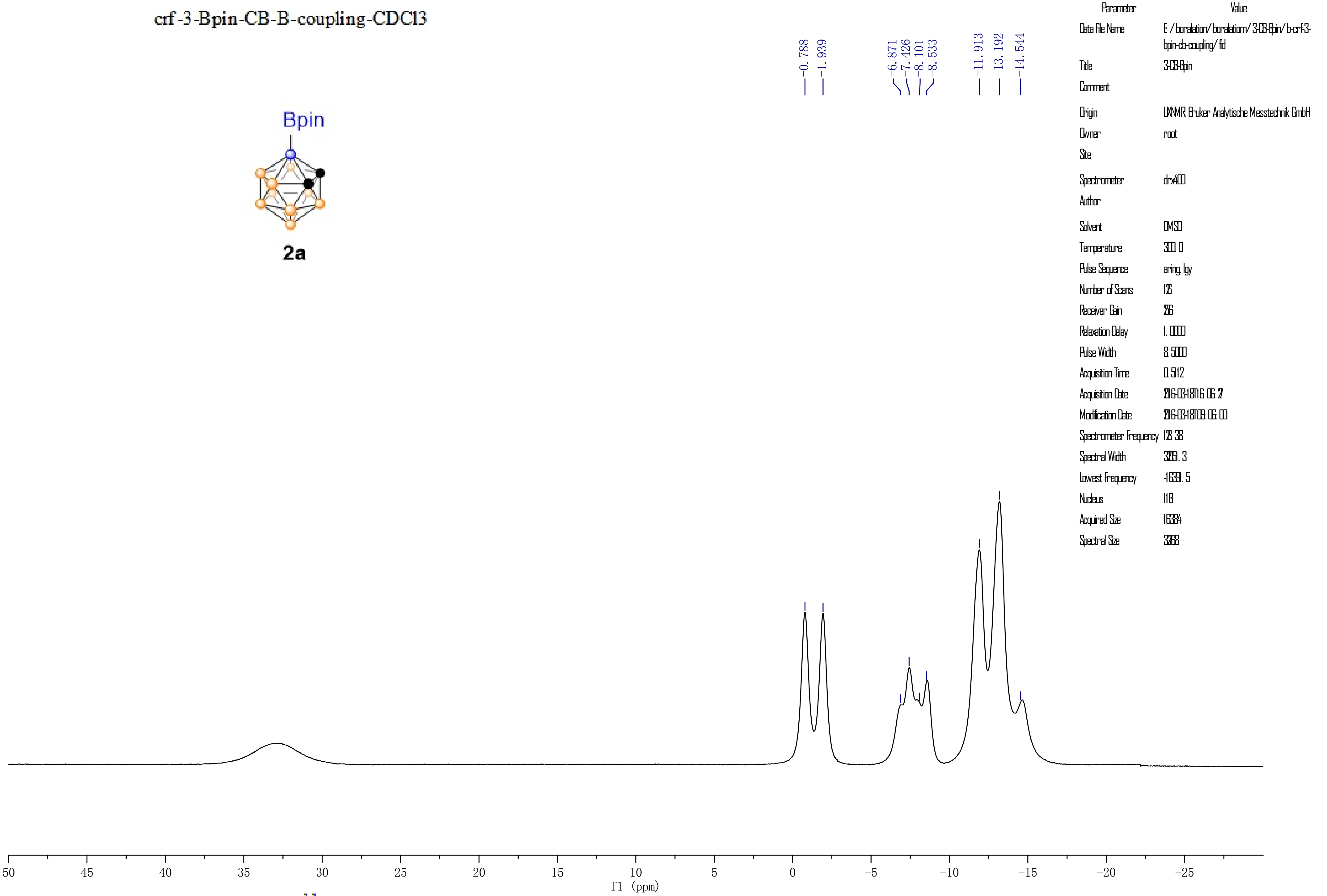

Supplementary Figure 32. ^{11}B **NMR Spectrum of 2a.**

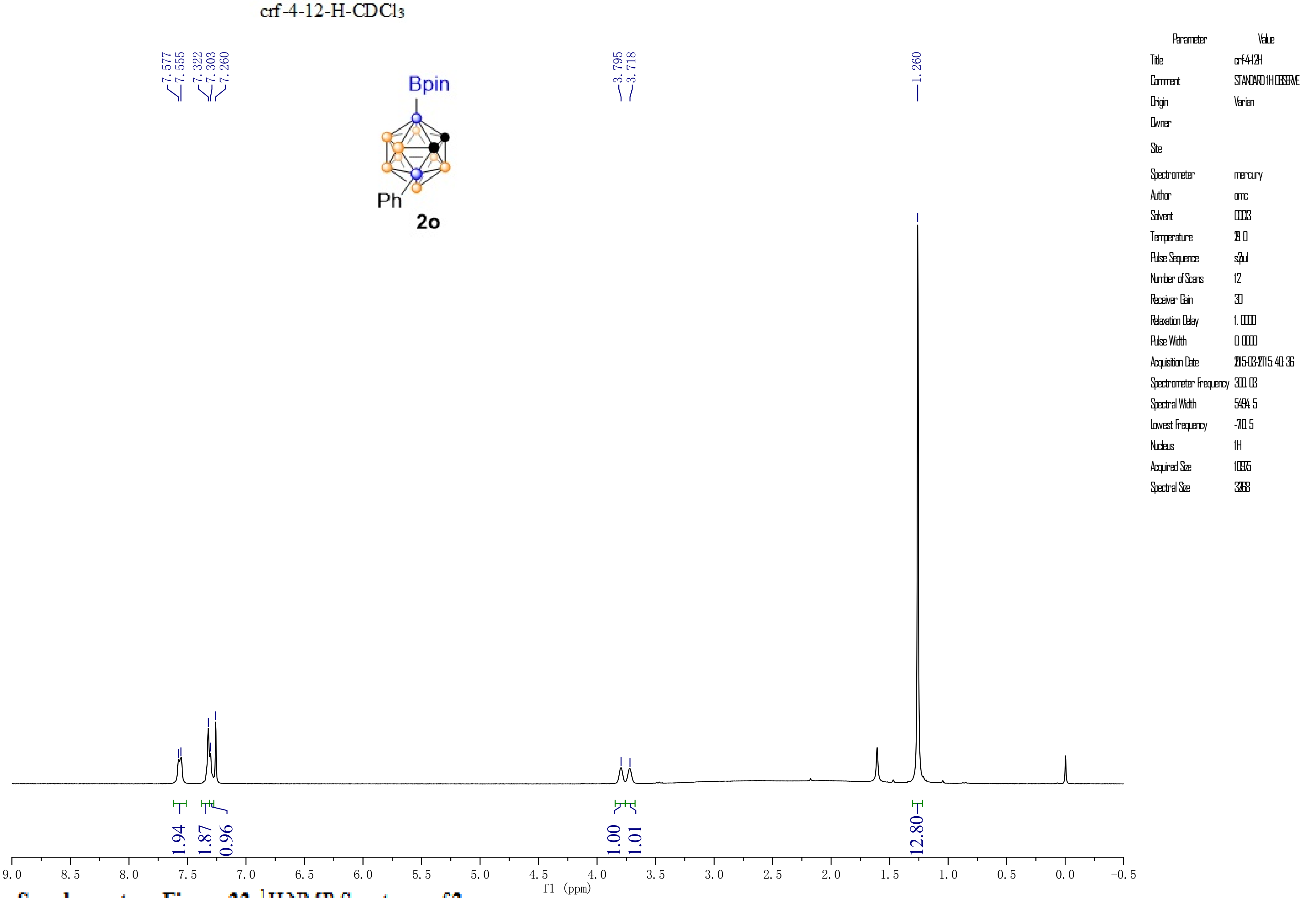

Supplementary Figure 33. ^{1}H NMR Spectrum of **2o**.

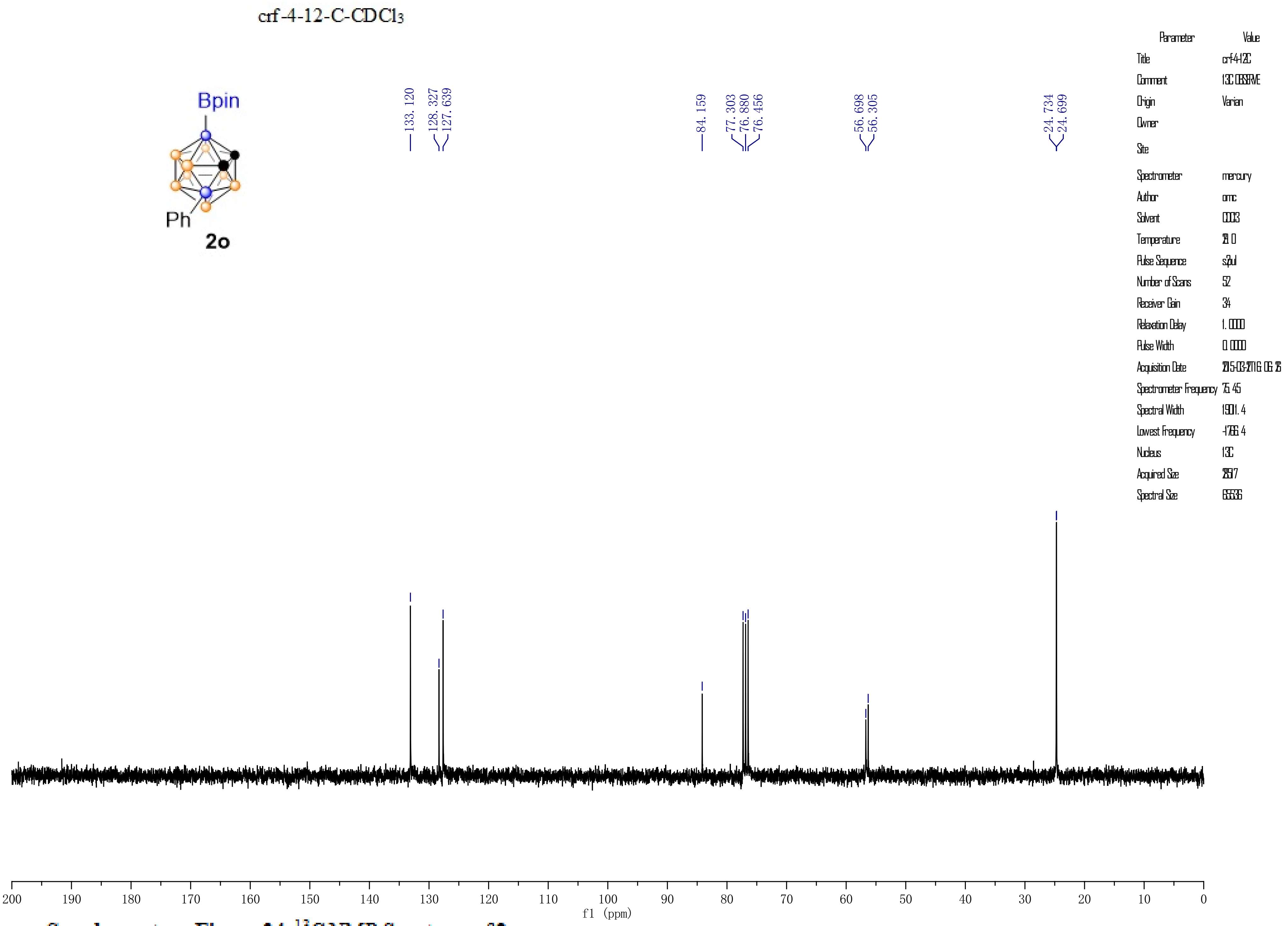

Supplementary Figure 34. ^{13}C NMR Spectrum of **2o**.

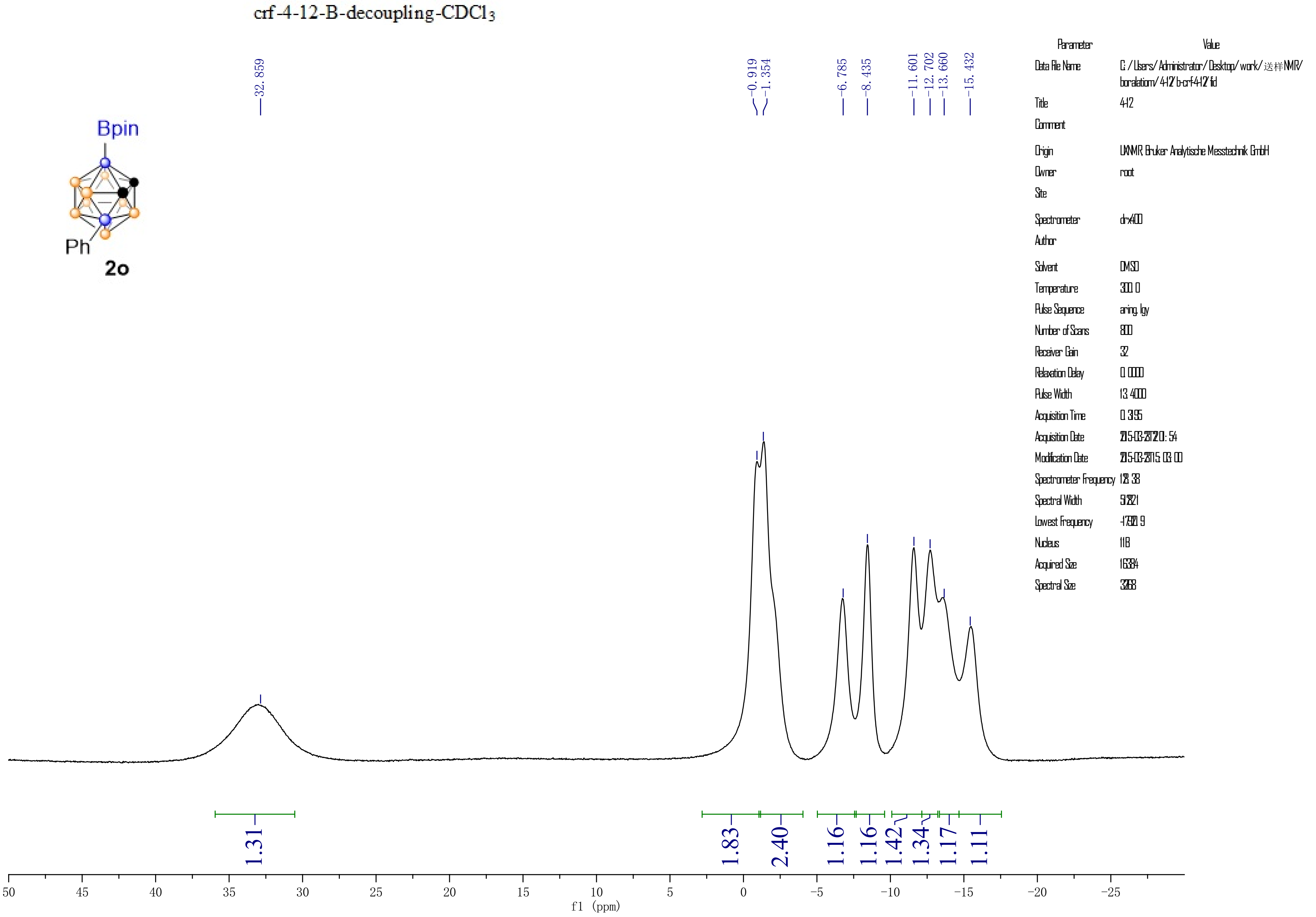

Supplementary Figure 35. $^{11}B\{^{1}H\}$ NMR Spectrum of **2o**.

crf-4-12-B-coupling-$CDCl_3$

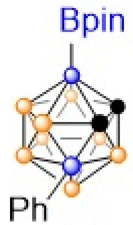

2o

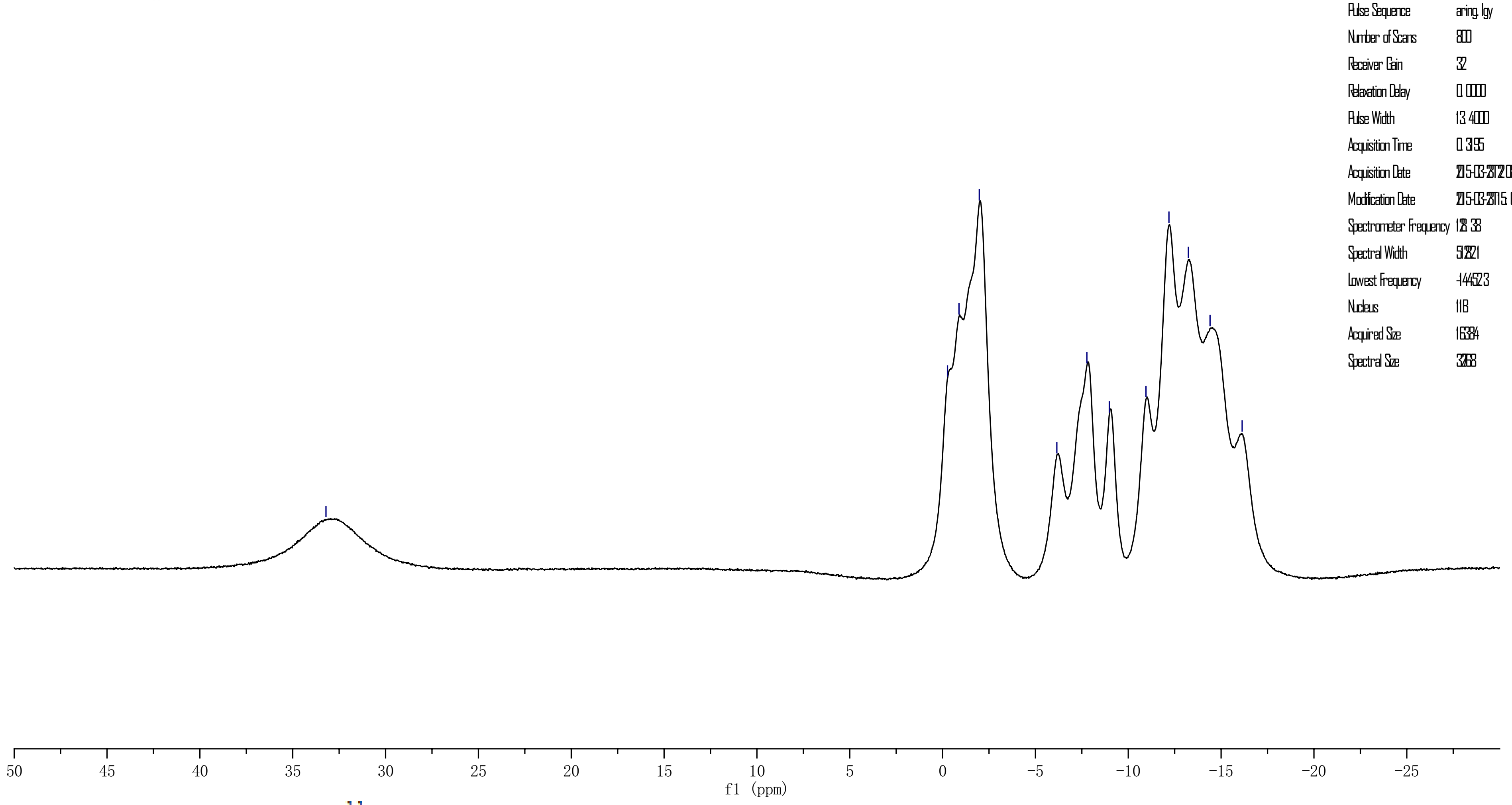

Parameter	Value
Data File Name	C:/Users/Administrator/Desktop/work/送样NMR/boralatiom/4-12/b-crf-4-12coupling/fid
Title	4-12
Comment	
Origin	UXNMR, Bruker Analytische Messtechnik GmbH
Owner	root
Site	
Spectrometer	drx400
Author	
Solvent	DMSO
Temperature	300.0
Pulse Sequence	aring.lgy
Number of Scans	800
Receiver Gain	32
Relaxation Delay	0.0000
Pulse Width	13.4000
Acquisition Time	0.3195
Acquisition Date	2015-03-28T12:08:[illegible]
Modification Date	2015-03-28T15:08:00
Spectrometer Frequency	128.38
Spectral Width	51282.1
Lowest Frequency	-14452.3
Nucleus	11B
Acquired Size	16384
Spectral Size	32768

Supplementary Figure 36. ^{11}B NMR Spectrum of **2o**.

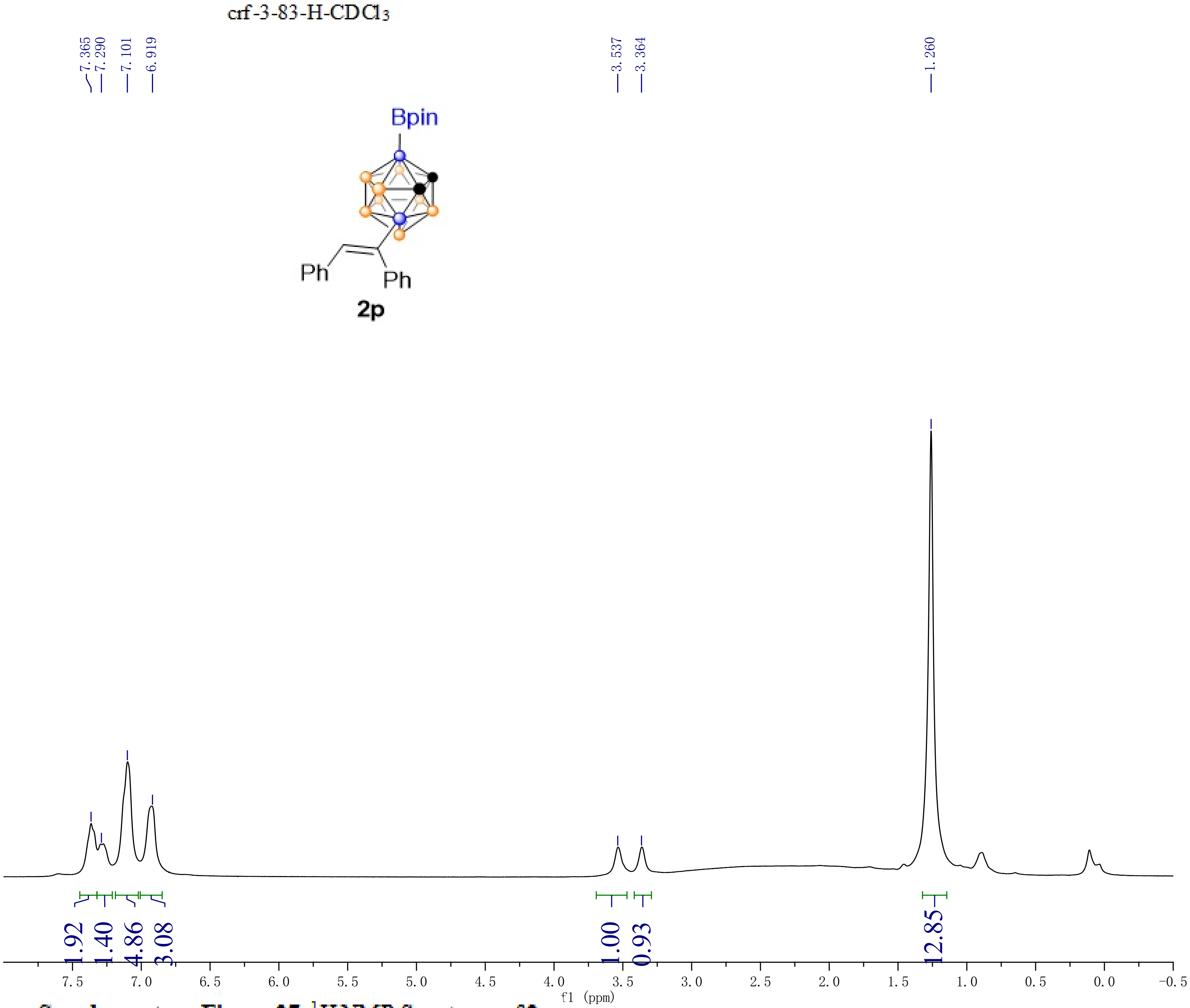

Parameter	Value
Title	crf-3-83-h-0207
Comment	STANDARD 1H OBSERVE
Origin	Varian
Owner	
Site	
Spectrometer	mercury
Author	omc
Solvent	CDCl3
Temperature	21.0
Pulse Sequence	s2pul
Number of Scans	4
Receiver Gain	12
Relaxation Delay	1.0000
Pulse Width	0.0000
Acquisition Date	2015-02-07T09:59:11
Spectrometer Frequency	300.03
Spectral Width	5494.5
Lowest Frequency	-707.9
Nucleus	1H
Acquired Size	10975
Spectral Size	32768

Supplementary Figure 37. ^{1}H NMR Spectrum of **2p**.

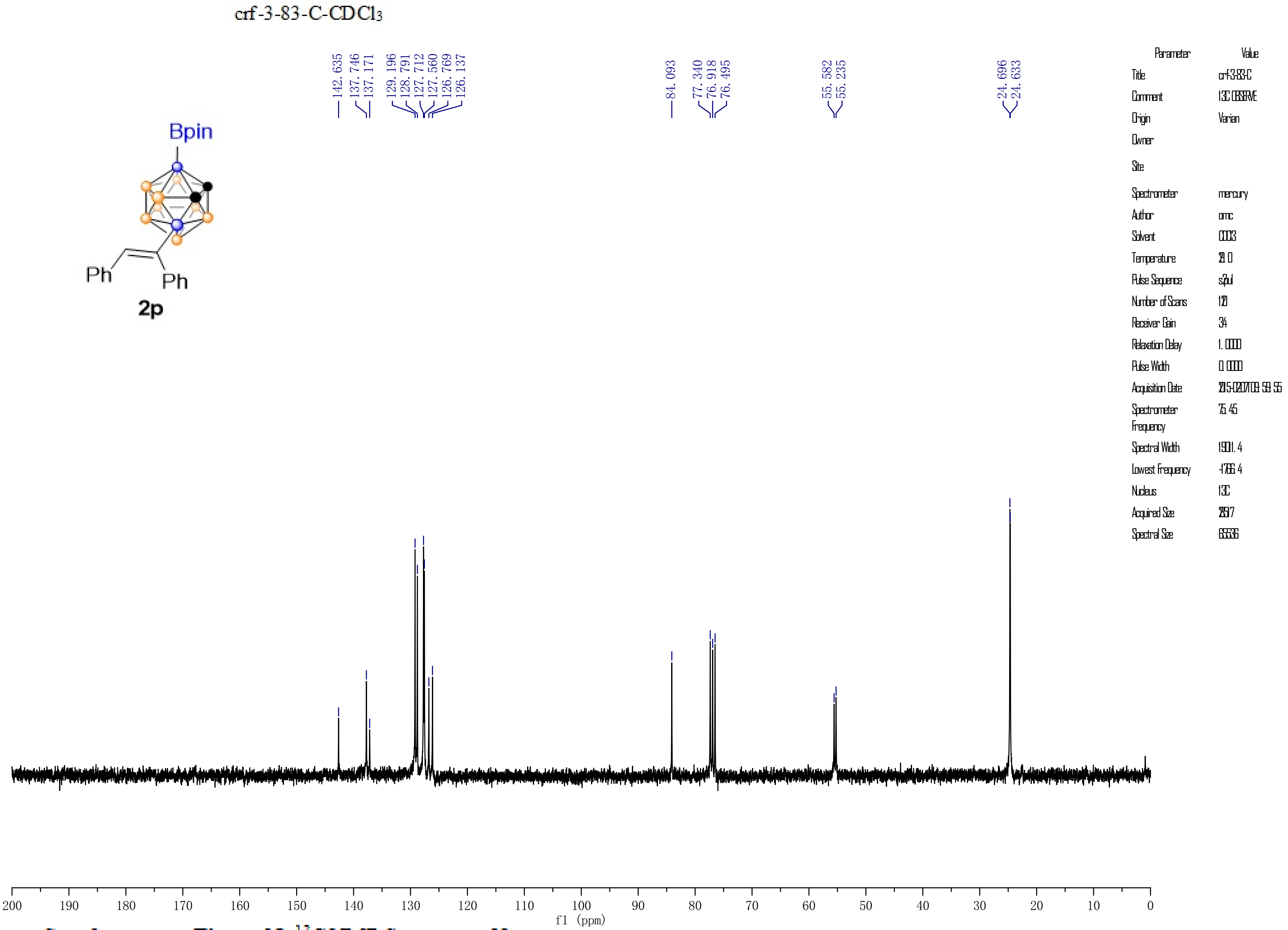

Supplementary Figure 38. ^{13}C NMR Spectrum of **2p**.

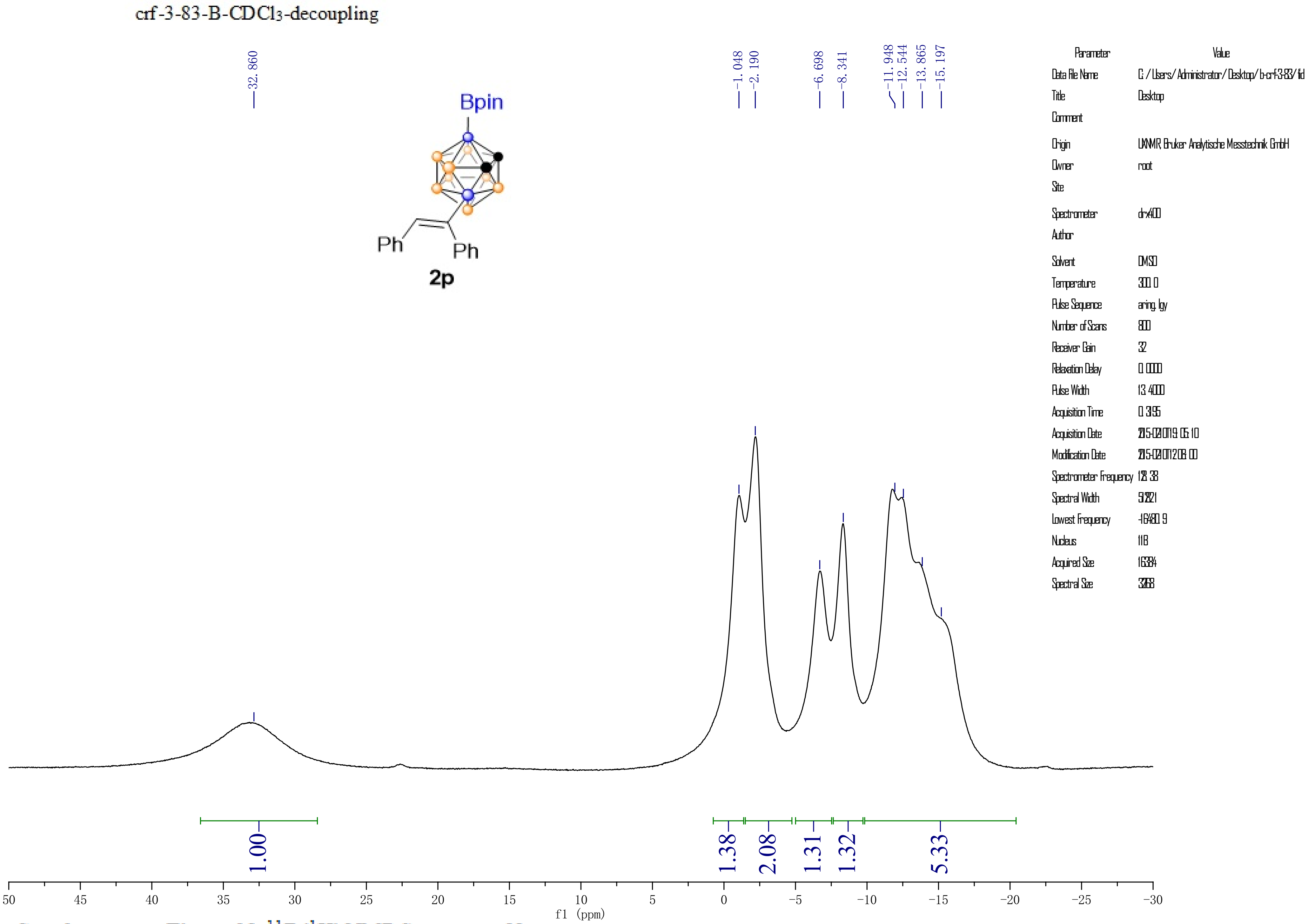

Supplementary Figure 39. $^{11}B\{^1H\}$ NMR Spectrum of **2p**.

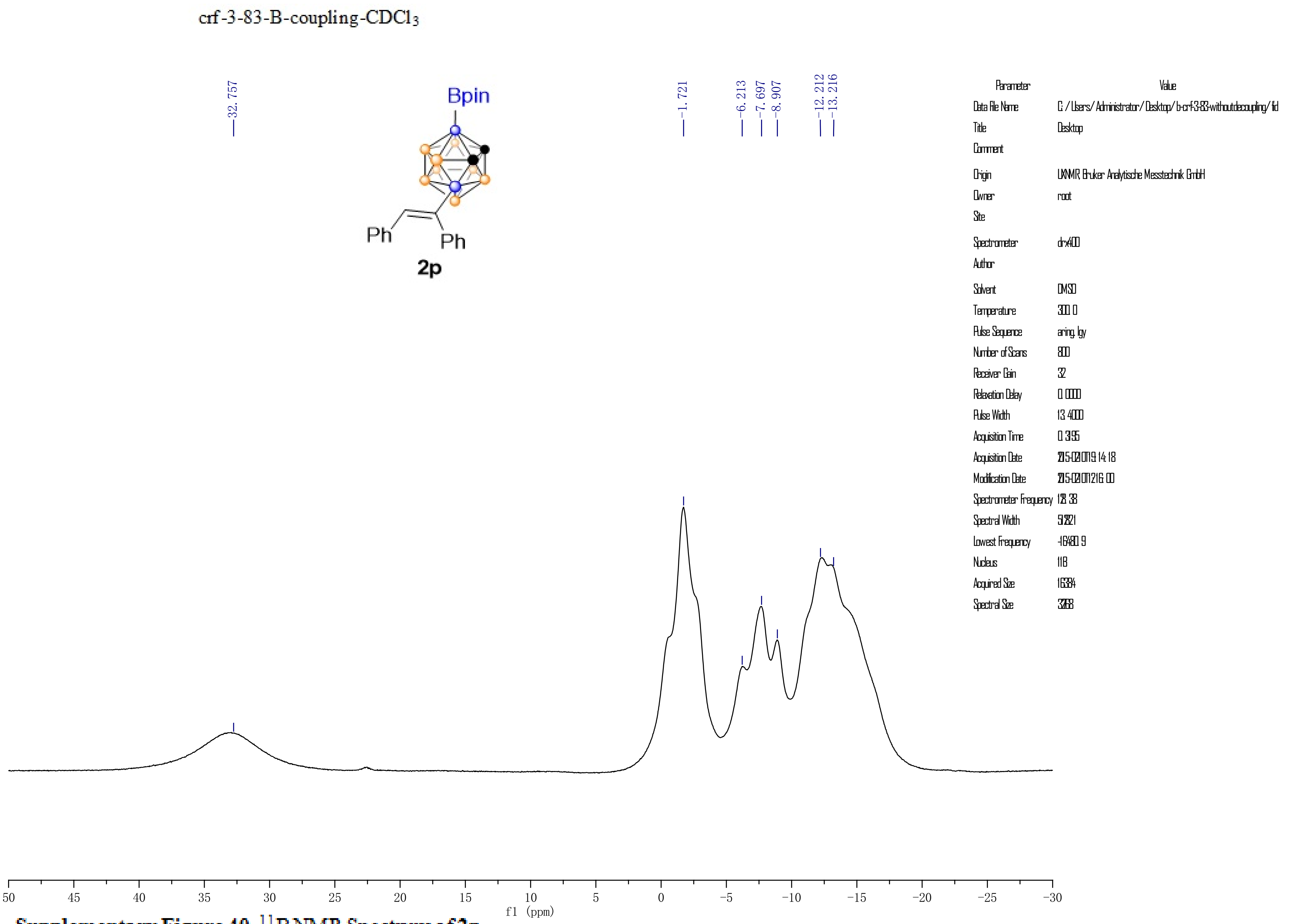

Supplementary Figure 40. ^{11}B NMR Spectrum of **2p**.

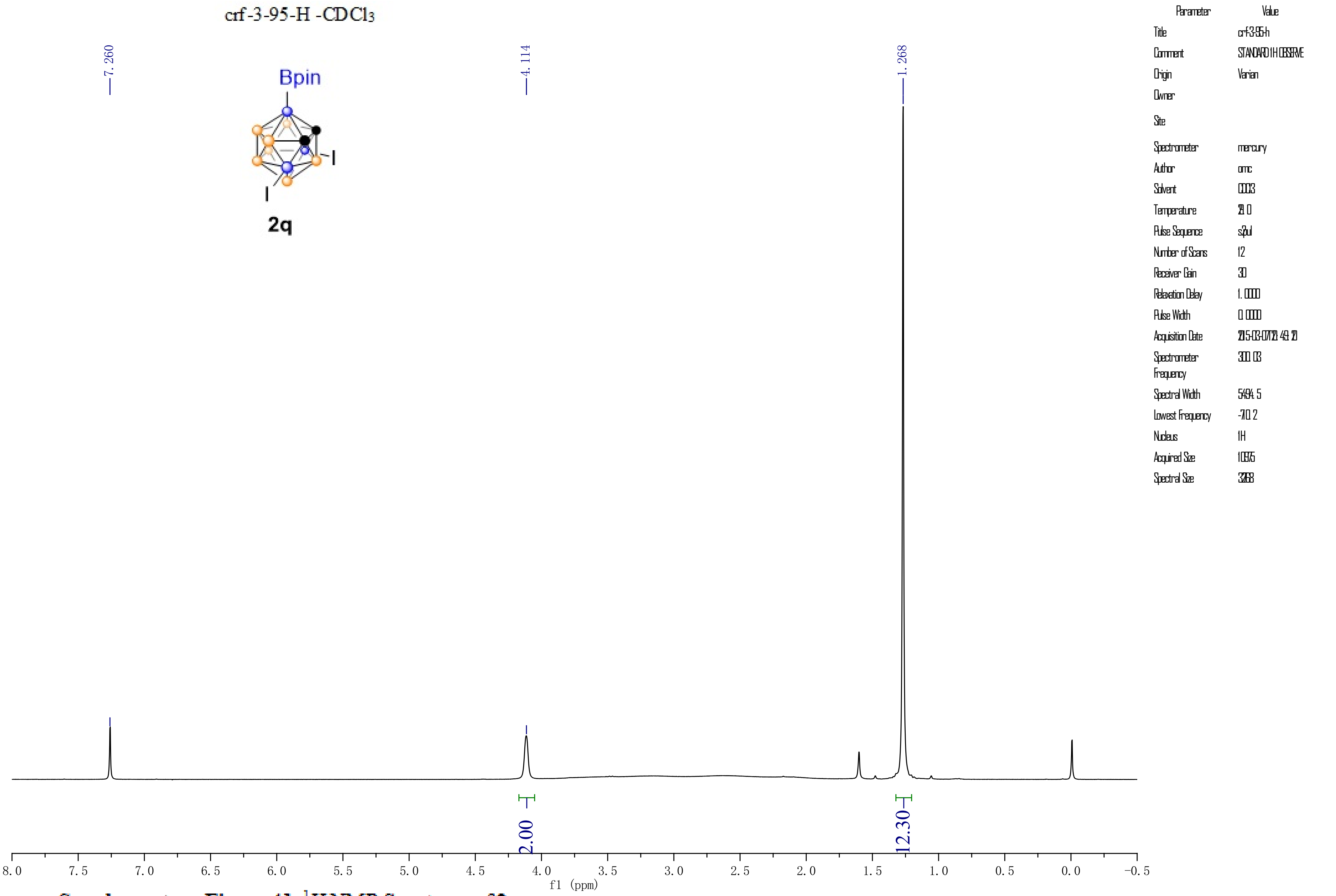

Supplementary Figure 41. ^{1}H NMR Spectrum of **2q**.

crf-3-95-C-$CDCl_3$

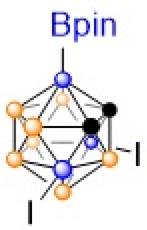

2q

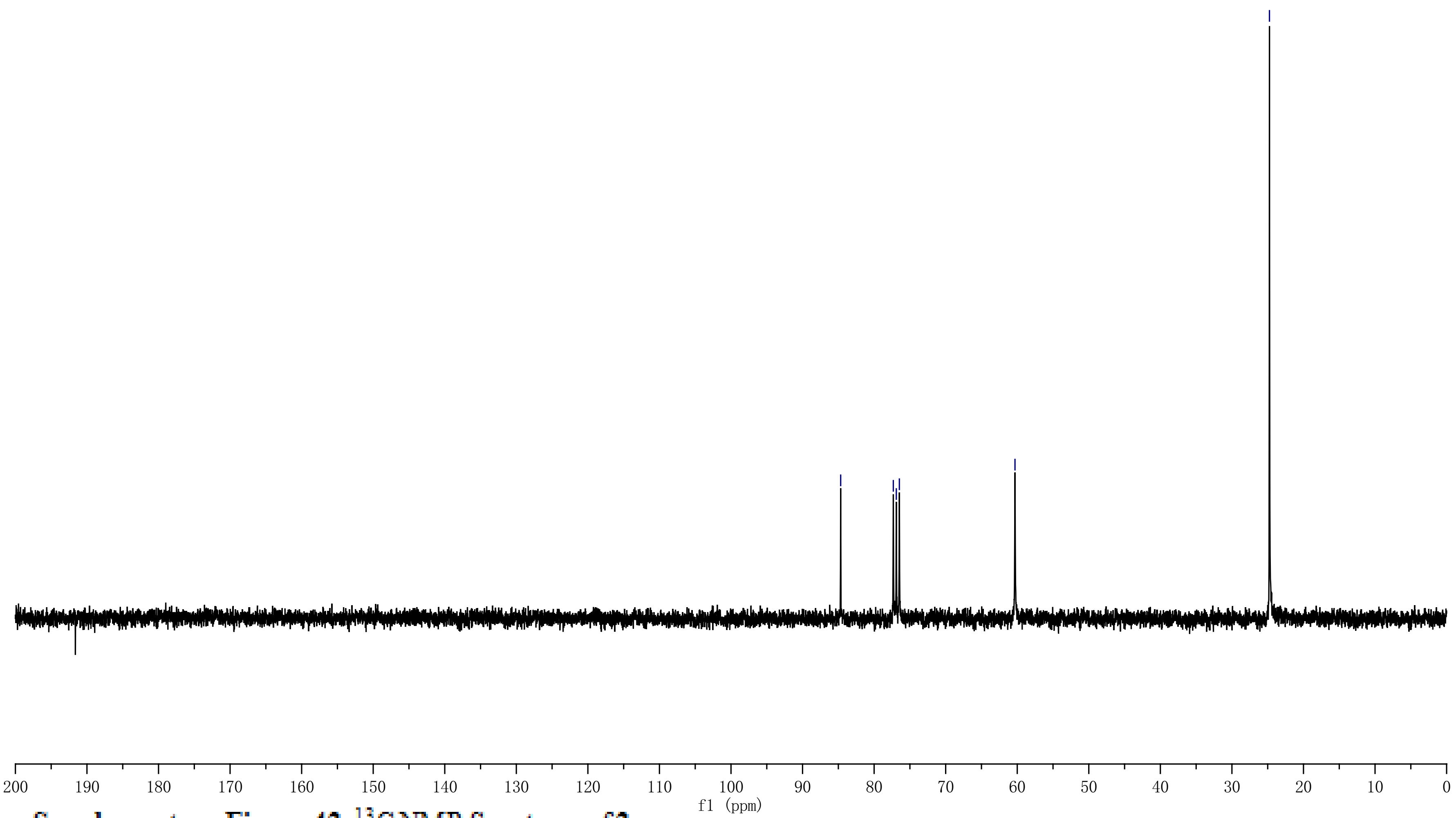

Parameter	Value
Title	crf-3-95-C
Comment	13C OBSERVE
Origin	Varian
Owner	
Site	
Spectrometer	mercury
Author	omc
Solvent	CDCl3
Temperature	21.0
Pulse Sequence	s2pul
Number of Scans	52
Receiver Gain	32
Relaxation Delay	1.0000
Pulse Width	0.0000
Acquisition Date	2015-03-07T21:32:52
Spectrometer Frequency	75.45
Spectral Width	19011.4
Lowest Frequency	-1766.4
Nucleus	13C
Acquired Size	28517
Spectral Size	65536

Supplementary Figure 42. ^{13}C NMR Spectrum of **2q**.

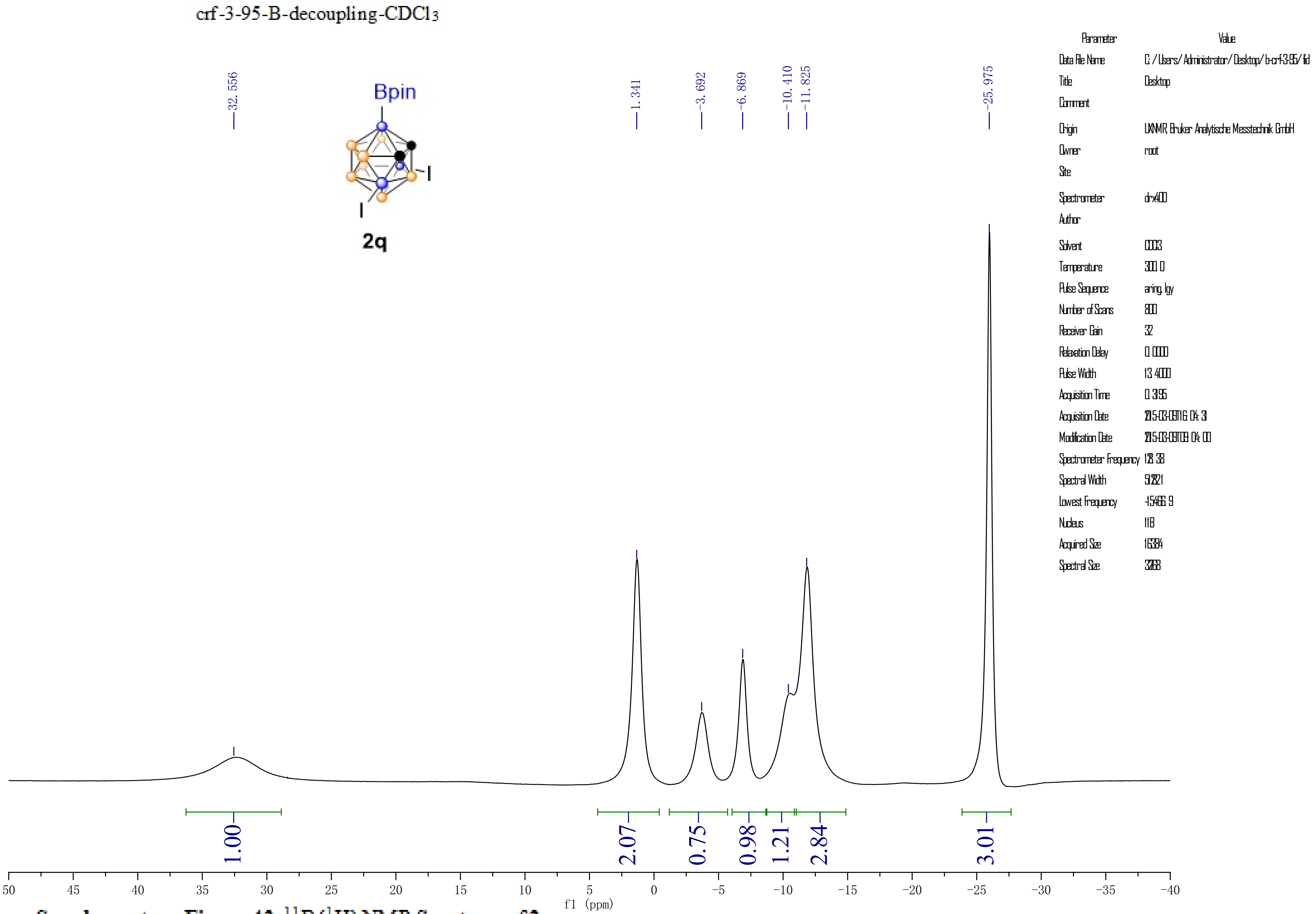

Supplementary Figure 43. $^{11}B\{^1H\}$ NMR Spectrum of **2q**.

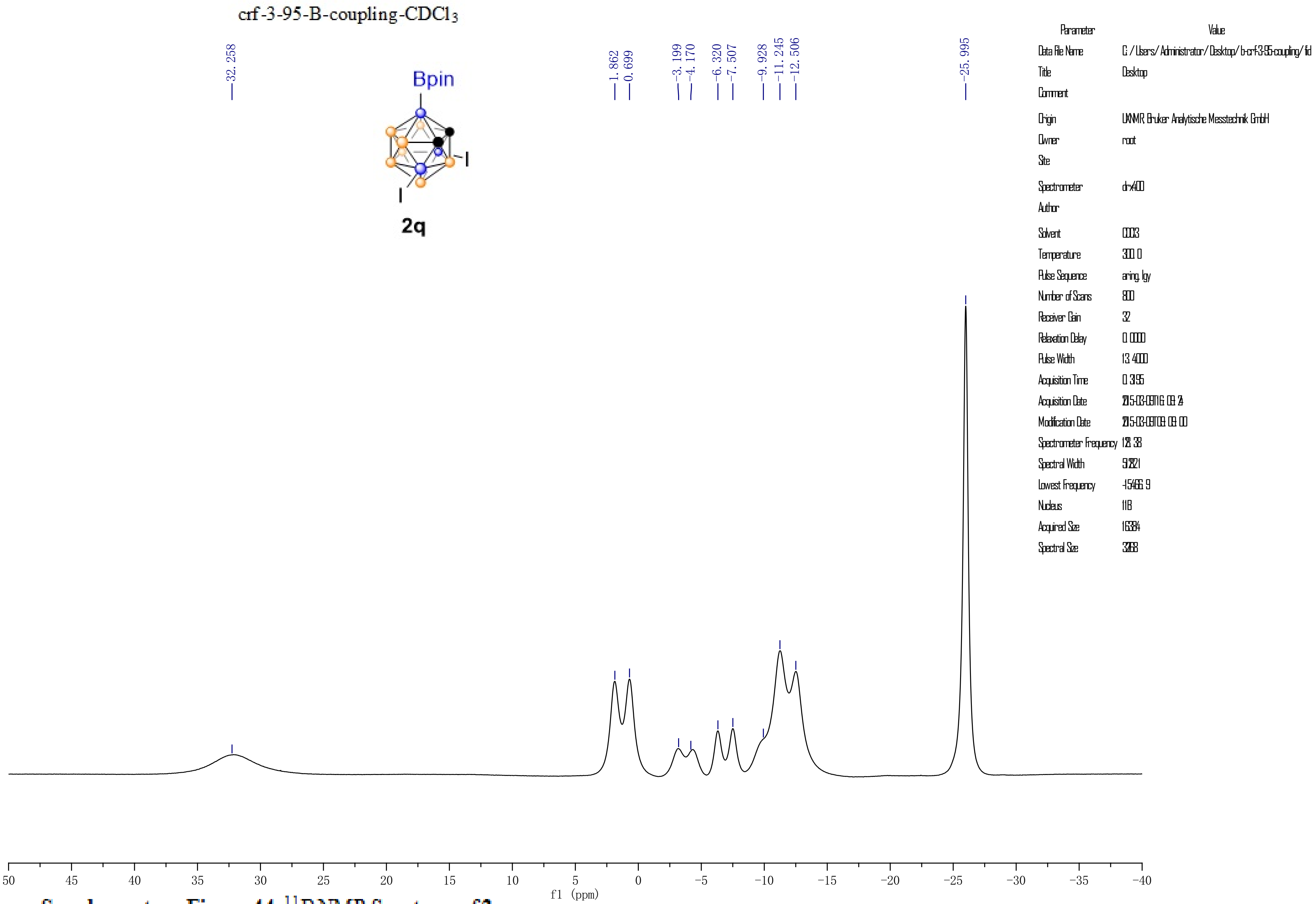

Supplementary Figure 44. ^{11}B NMR Spectrum of **2q**.

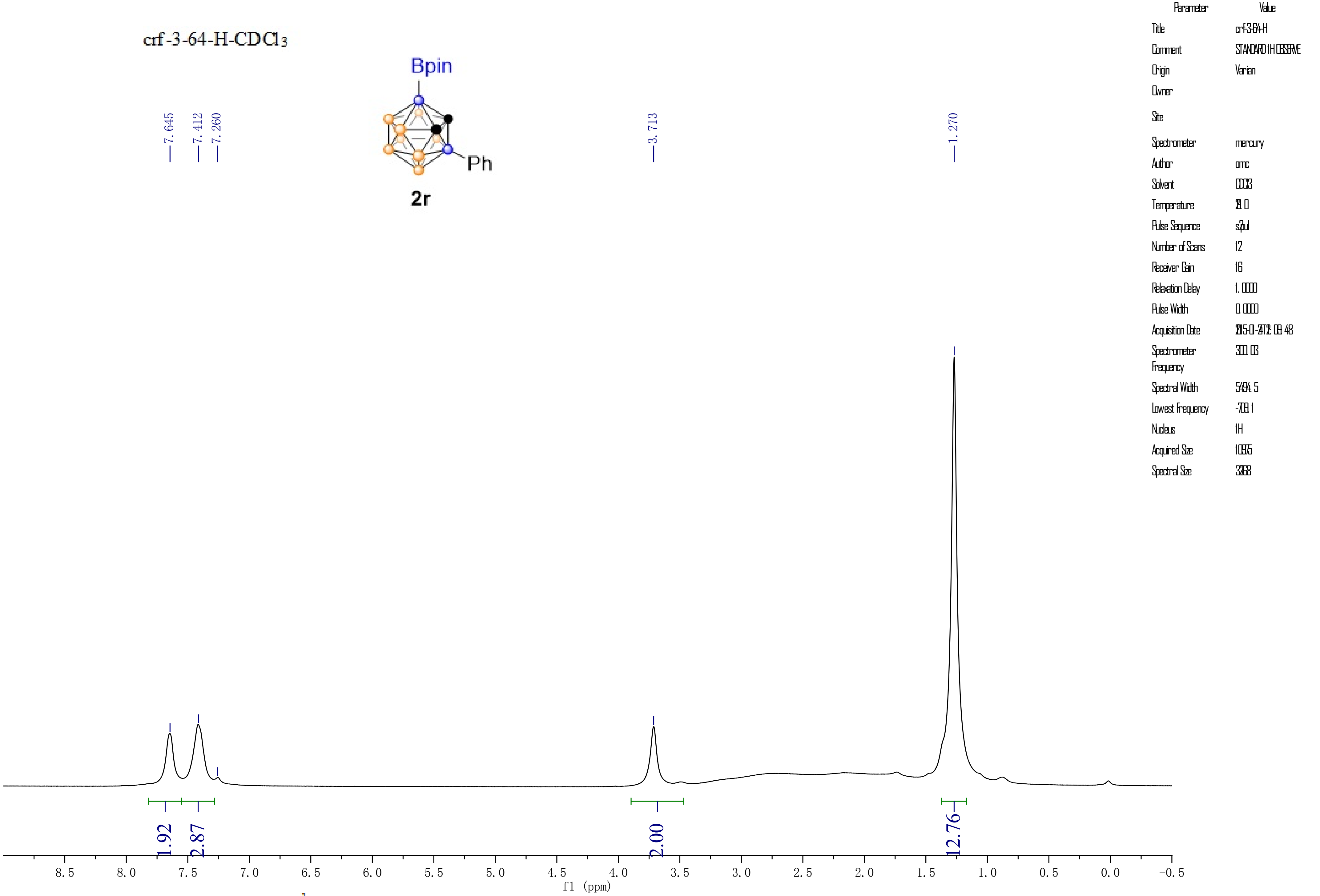

Supplementary Figure 45. ^{1}H NMR Spectrum of **2r**.

crf-3-64-C-CDCl3

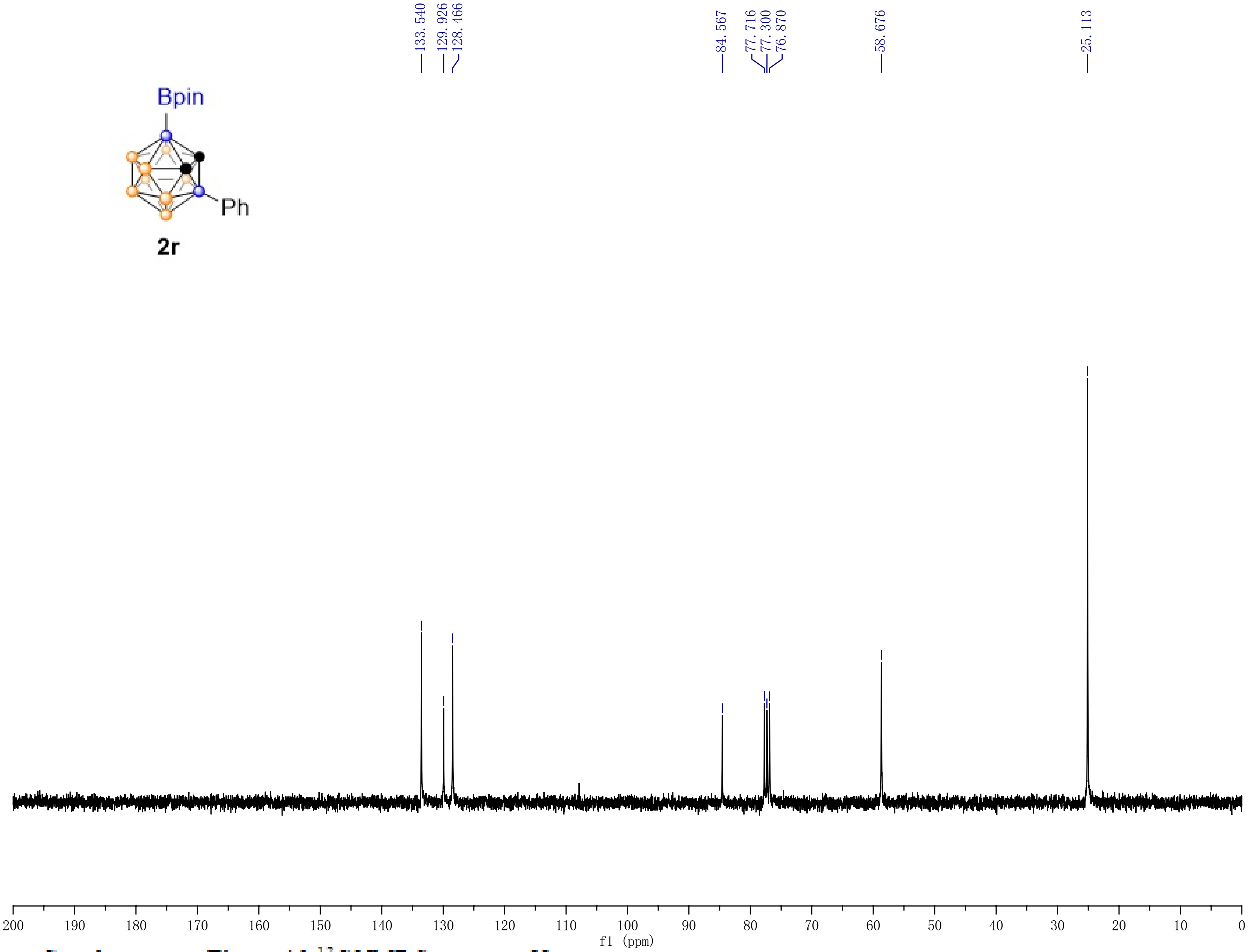

Parameter	Value
Title	crf-3-64-C
Comment	13C OBSERVE
Origin	Varian
Owner	
Site	
Spectrometer	mercury
Author	omc
Solvent	CDCl3
Temperature	21.0
Pulse Sequence	s2pul
Number of Scans	120
Receiver Gain	30
Relaxation Delay	1.0000
Pulse Width	0.0000
Acquisition Date	2015-01-24T12:10:50
Spectrometer Frequency	75.45
Spectral Width	18797.0
Lowest Frequency	-1627.8
Nucleus	13C
Acquired Size	28195
Spectral Size	65536

Supplementary Figure 46. ^{13}C NMR Spectrum of **2r**.

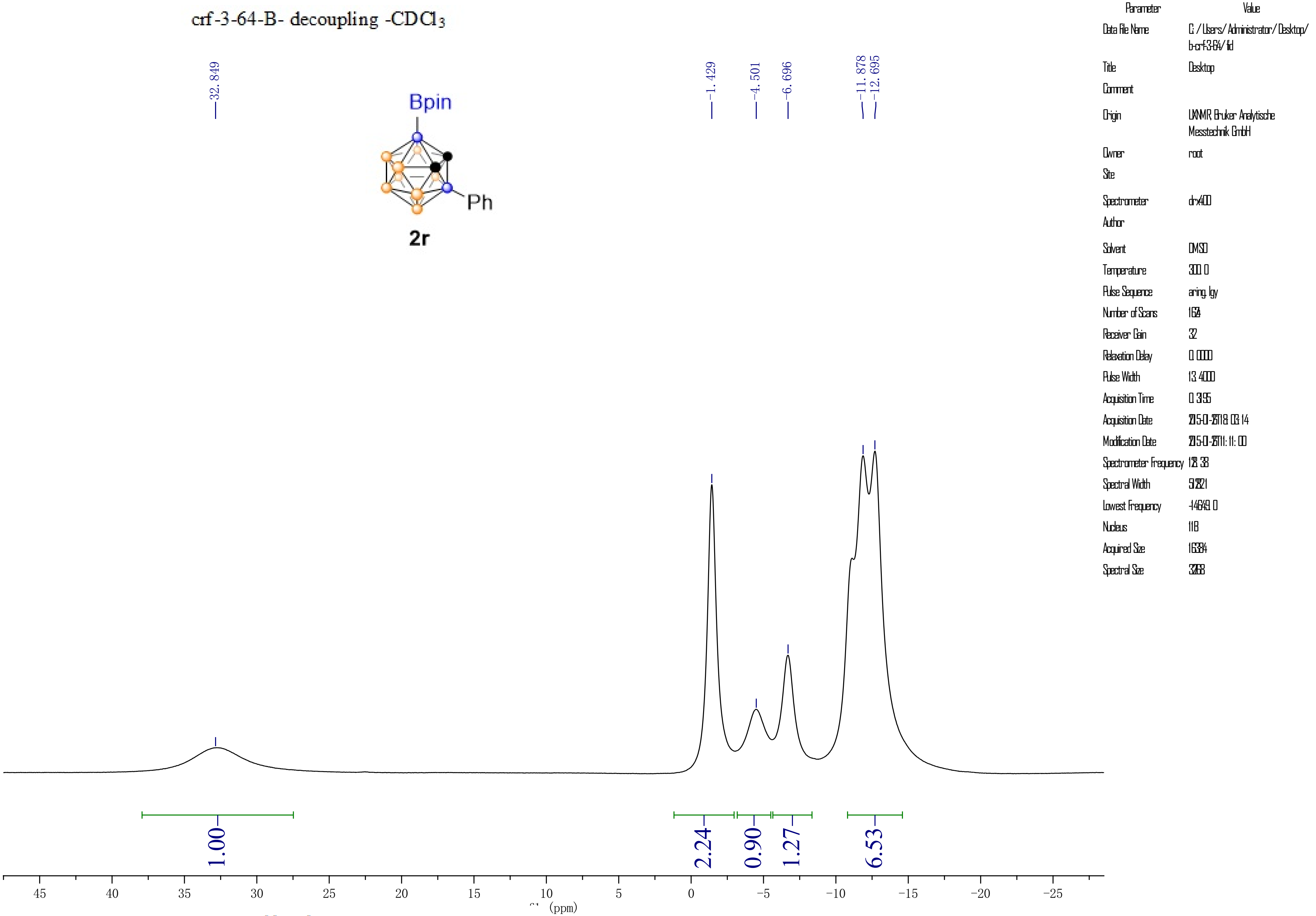

Supplementary Figure 47. $^{11}B\{^1H\}$ **NMR Spectrum of 2r.**

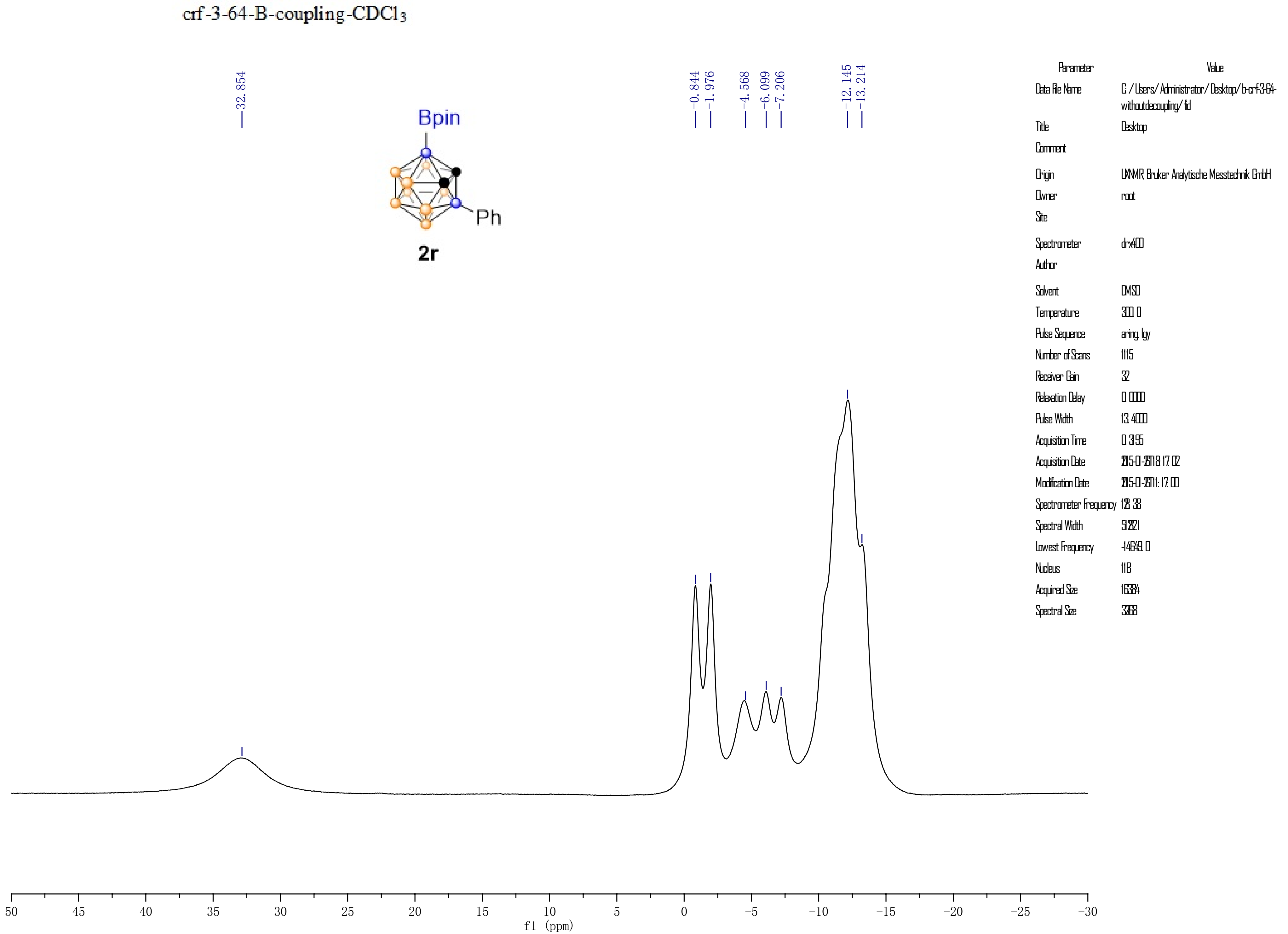

Supplementary Figure 48. ^{11}B NMR Spectrum of **2r**.

crf-CB-Bpin$_2$-H-CDCl$_3$

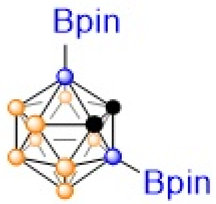

3a

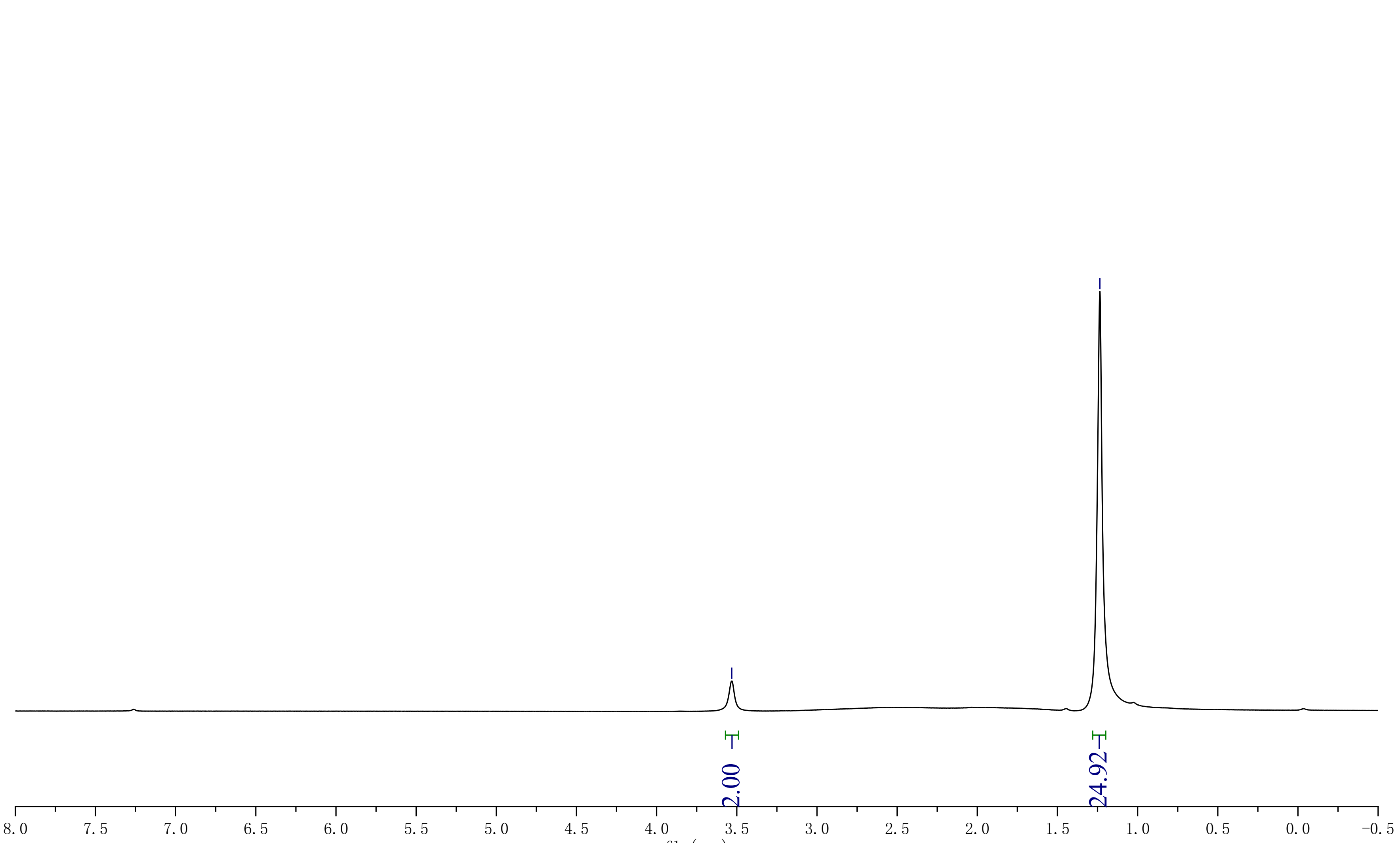

Parameter	Value
Title	crf-CB-Bpin2
Comment	STANDARD 1H OBSERVE
Origin	Varian
Owner	
Site	
Spectrometer	mercury
Author	omc
Solvent	CDCl3
Temperature	[illegible]
Pulse Sequence	s2pul
Number of Scans	[illegible]
Receiver Gain	10
Relaxation Delay	1.0000
Pulse Width	0.0000
Acquisition Date	2015-01-11T15:[illegible]
Spectrometer Frequency	300.03
Spectral Width	5494.5
Lowest Frequency	-707.4
Nucleus	1H
Acquired Size	10975
Spectral Size	32768

Supplementary Figure 49. ^{1}H NMR Spectrum of **3a**.

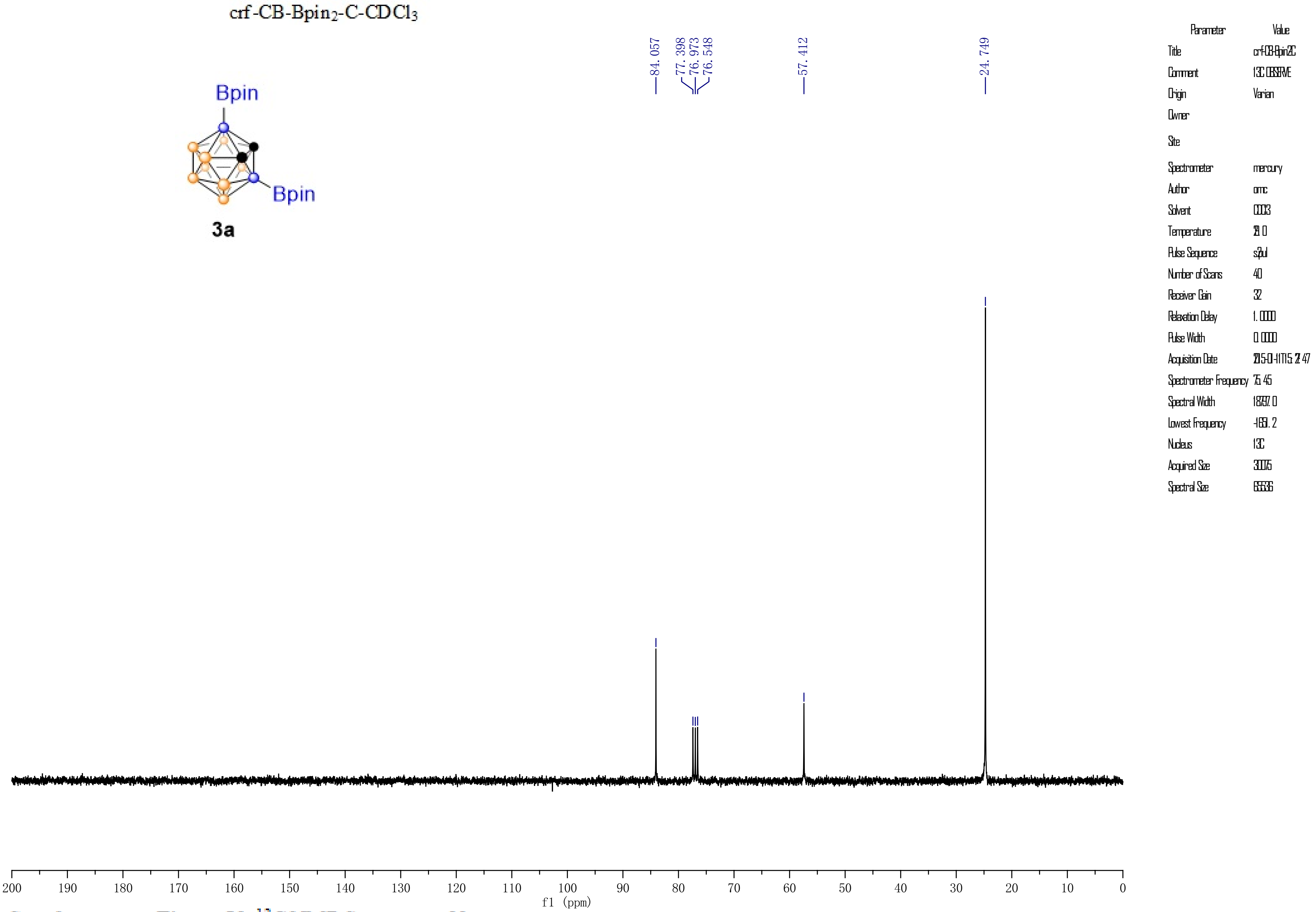

Supplementary Figure 50. ^{13}C NMR Spectrum of **3a**.

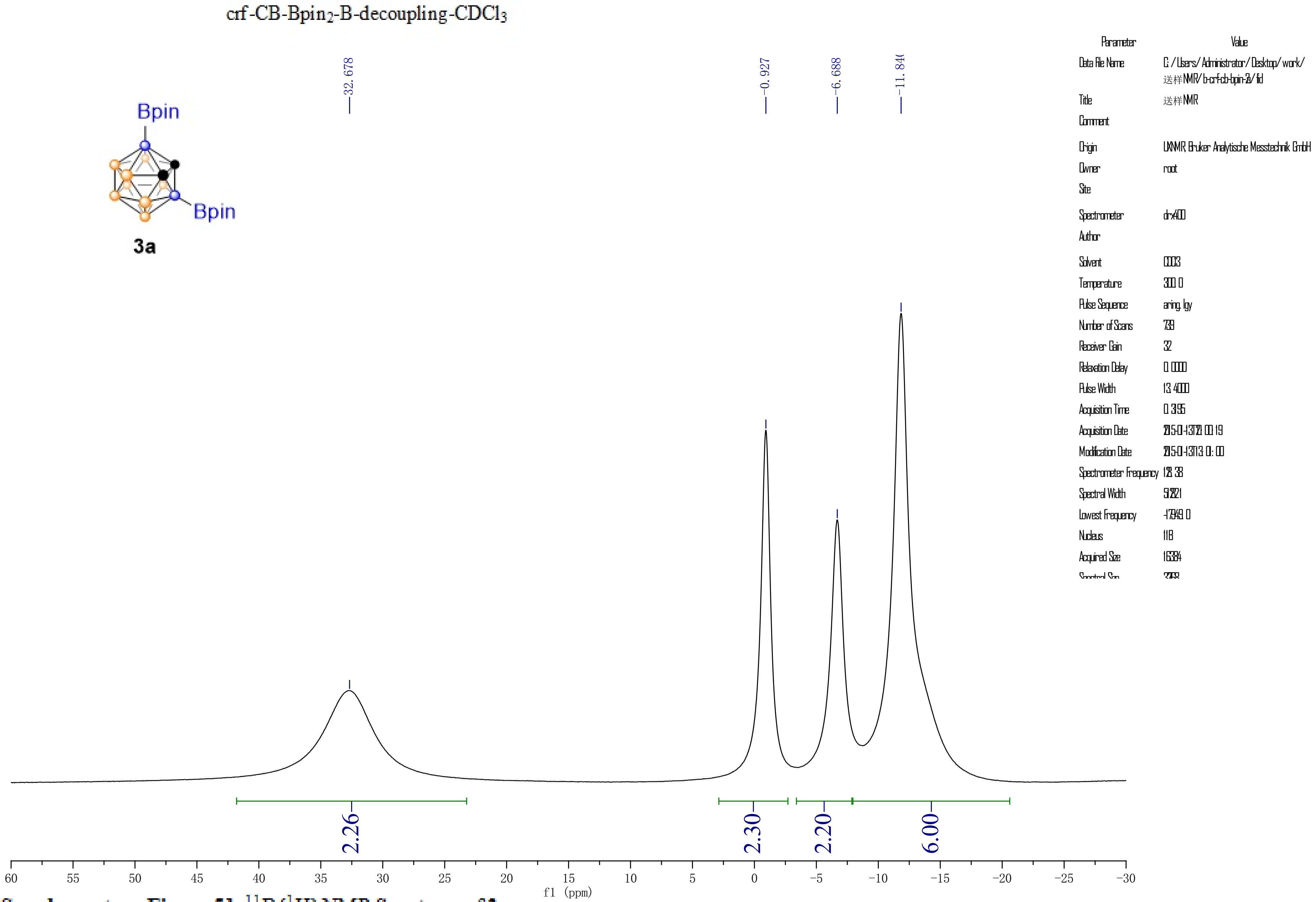

Supplementary Figure 51. $^{11}B\{^{1}H\}$ **NMR Spectrum of 3a.**

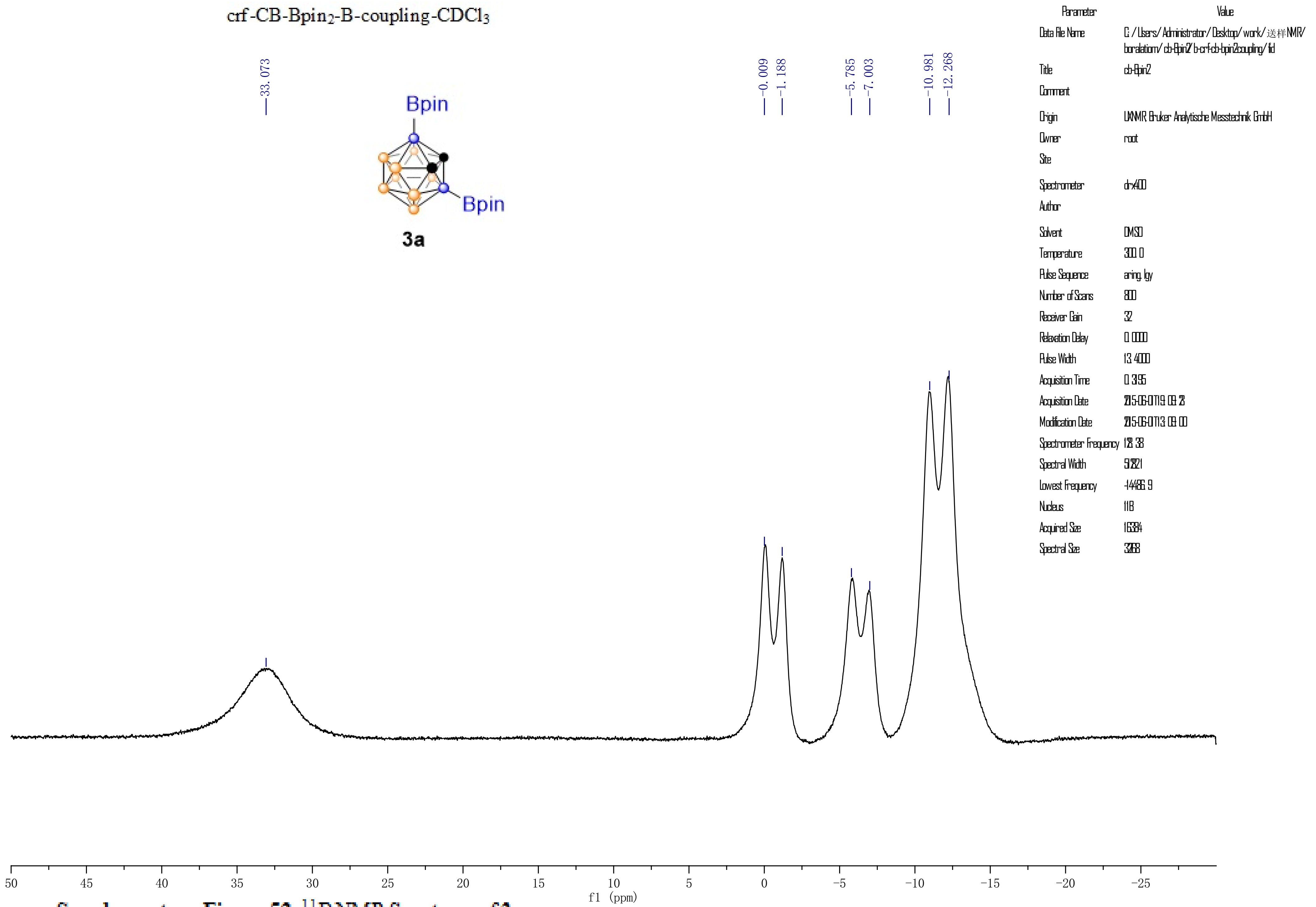

Supplementary Figure 52. ^{11}B NMR Spectrum of **3a**.

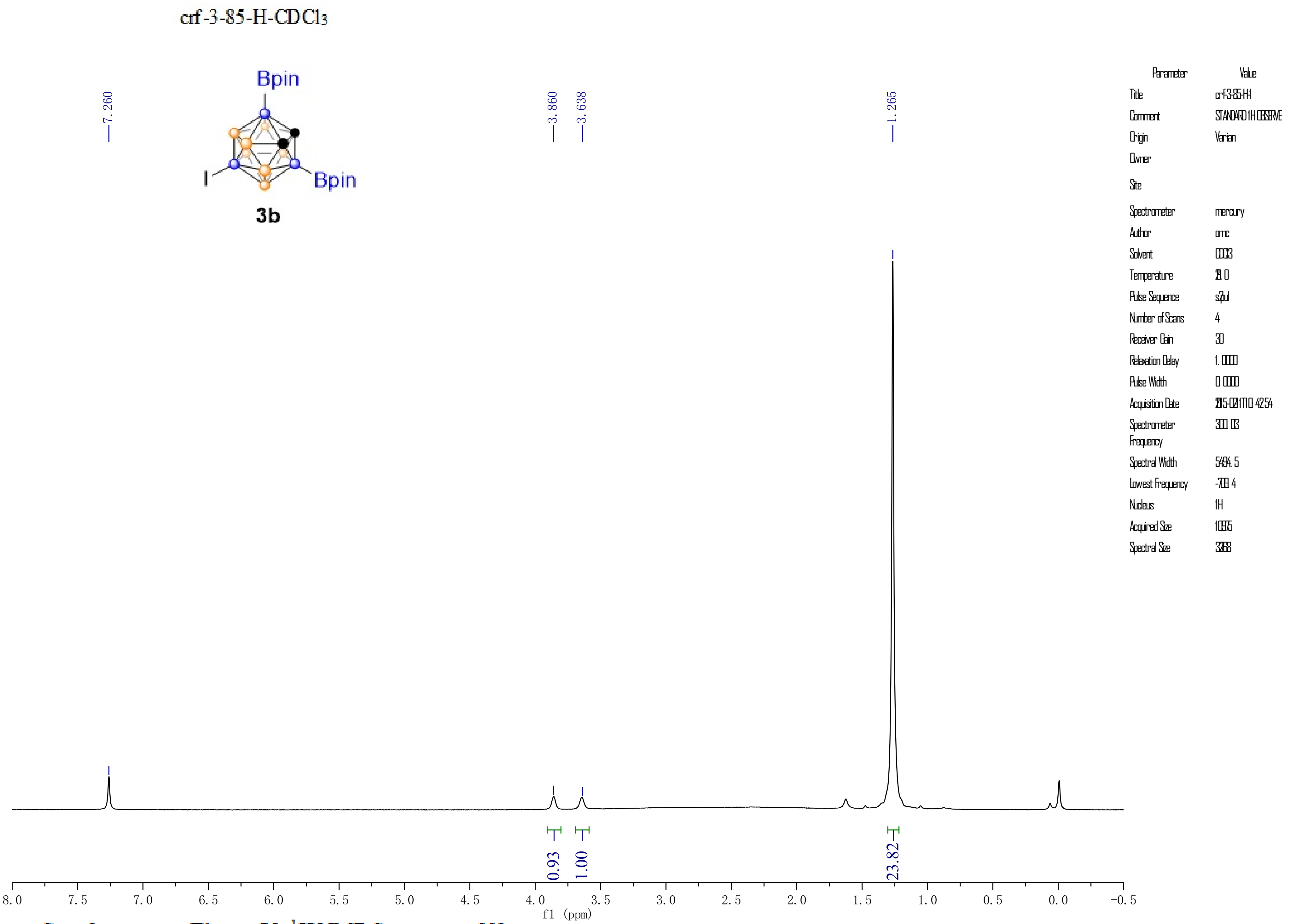

Supplementary Figure 53. ^{1}H NMR Spectrum of **3b**.

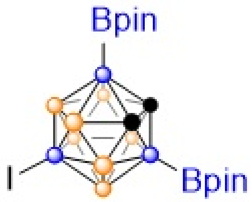

3b

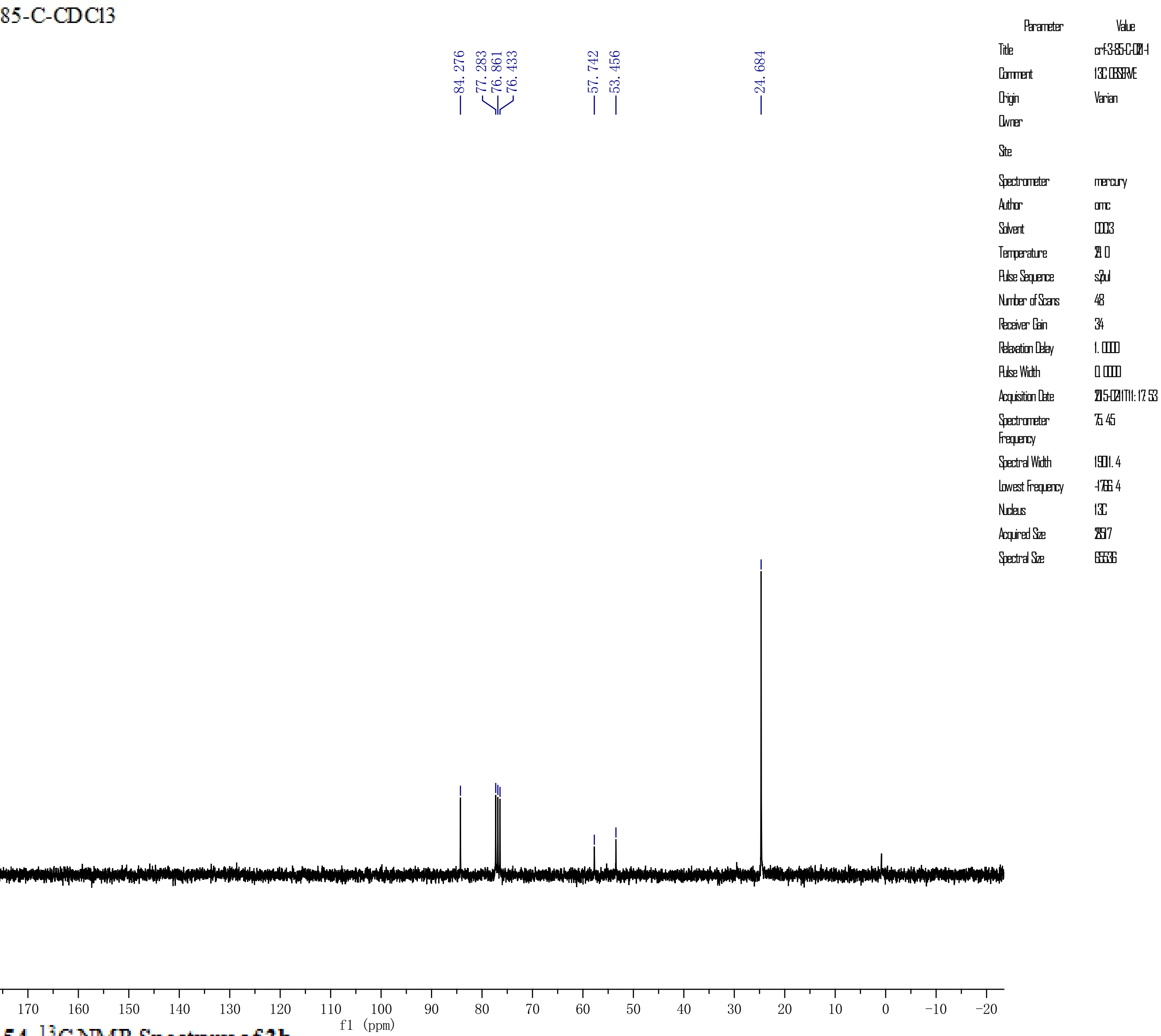

Supplementary Figure 54. ^{13}C NMR Spectrum of **3b**.

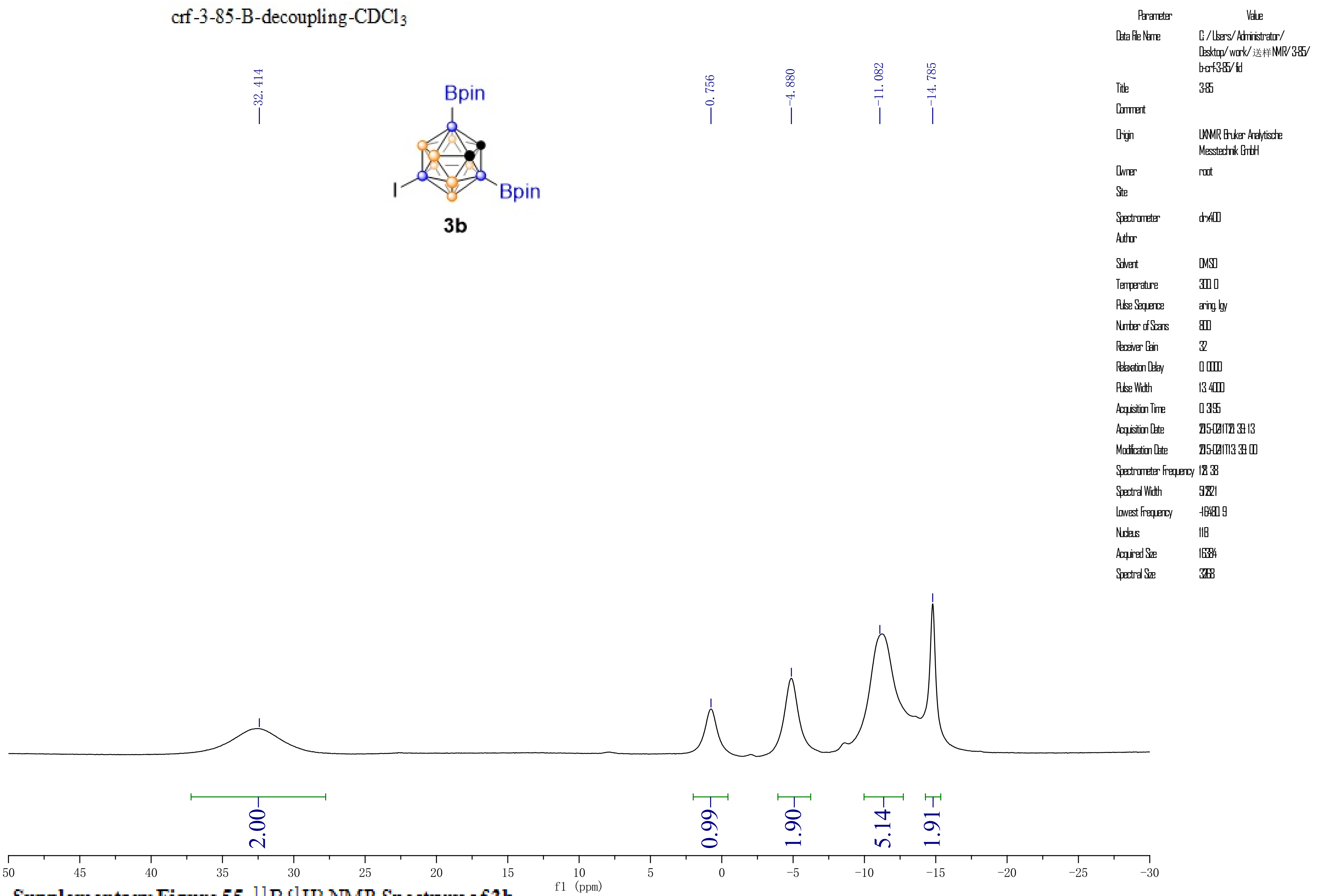

Supplementary Figure 55. $^{11}B\{^1H\}$ NMR Spectrum of **3b**.

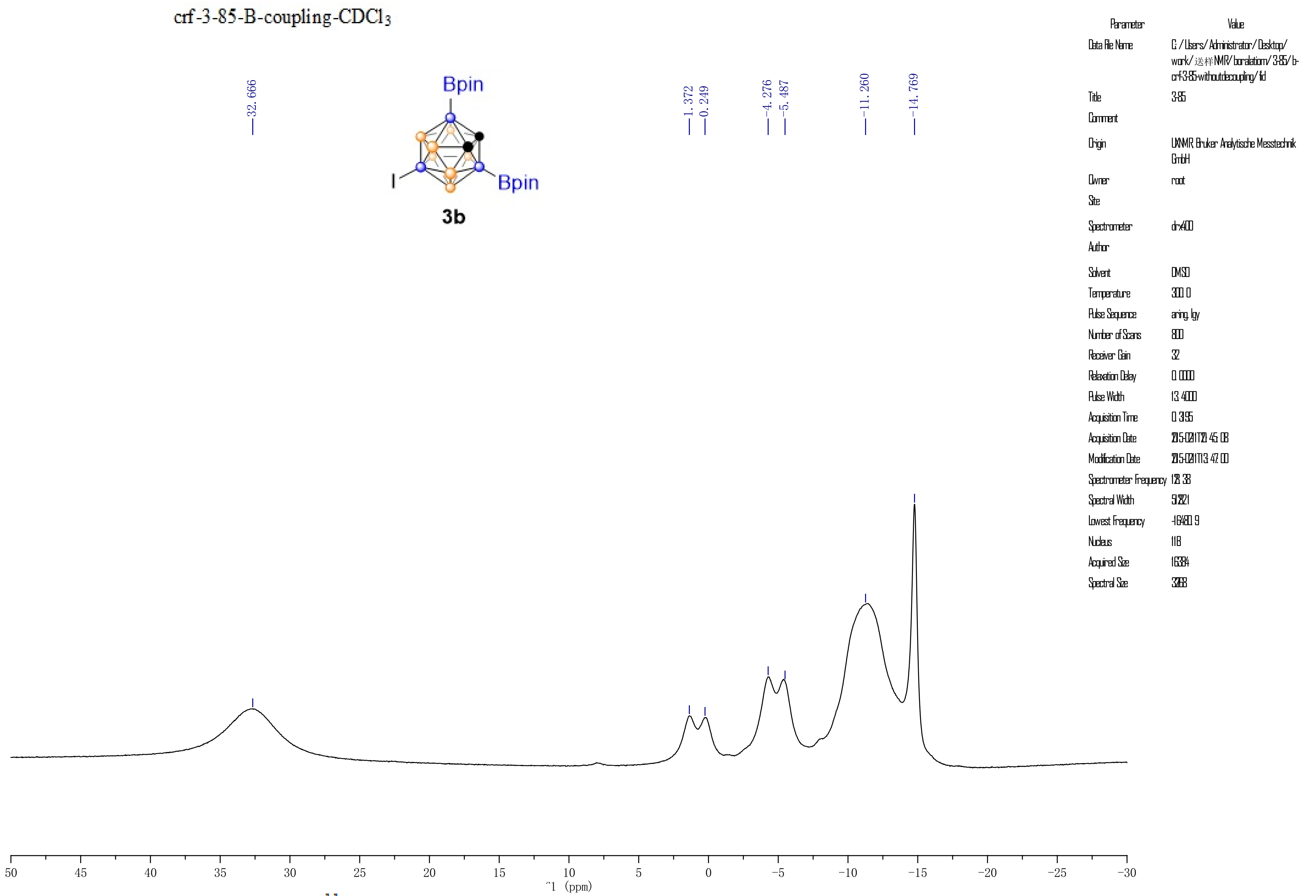

Supplementary Figure 56. ^{11}B NMR Spectrum of **3b**.

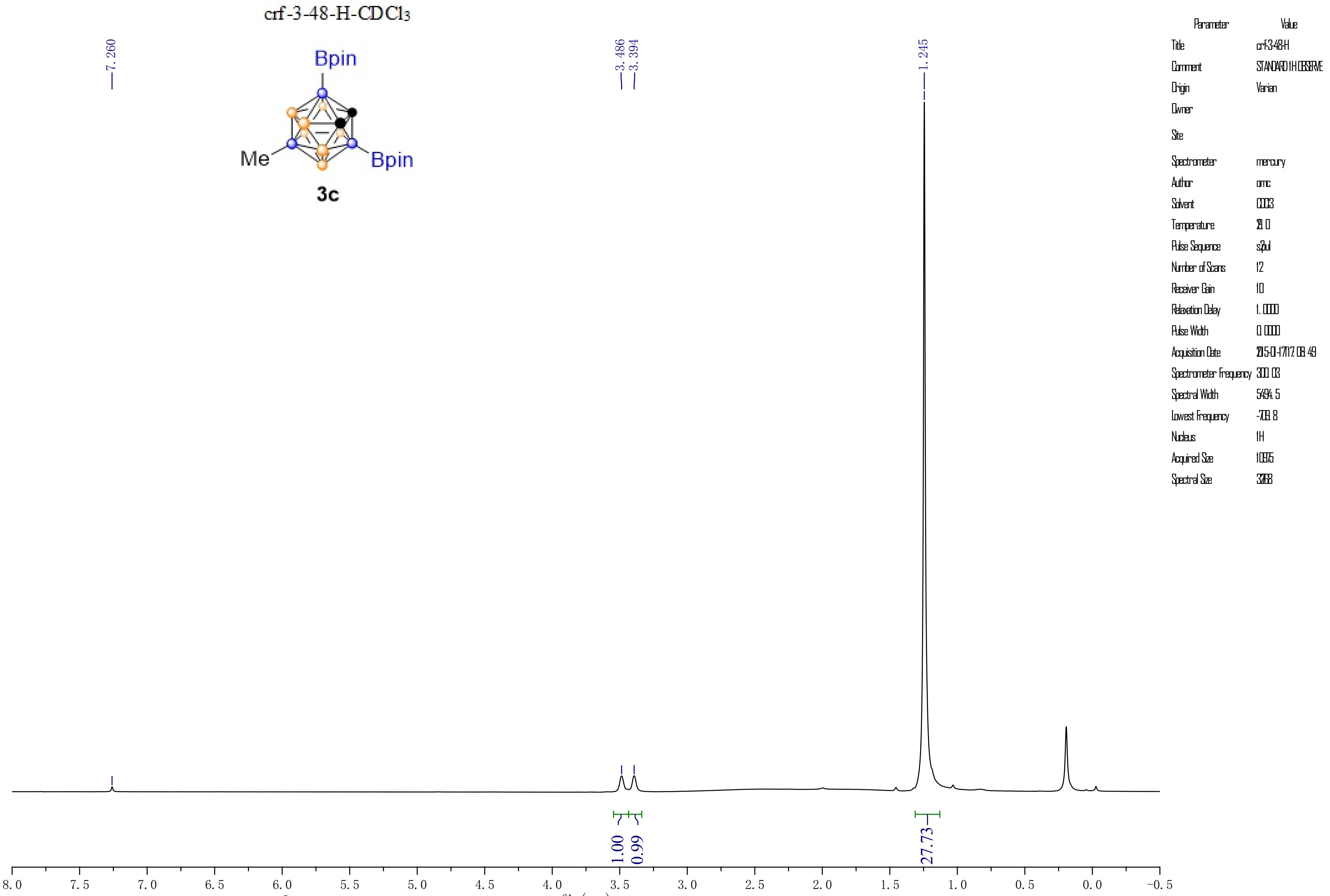

Supplementary Figure 57. ^{1}H NMR Spectrum of **3c**.

crf-3-48-C-$CDCl_3$

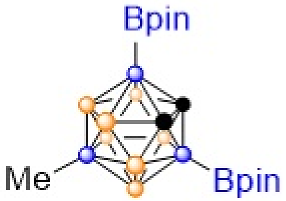

3c

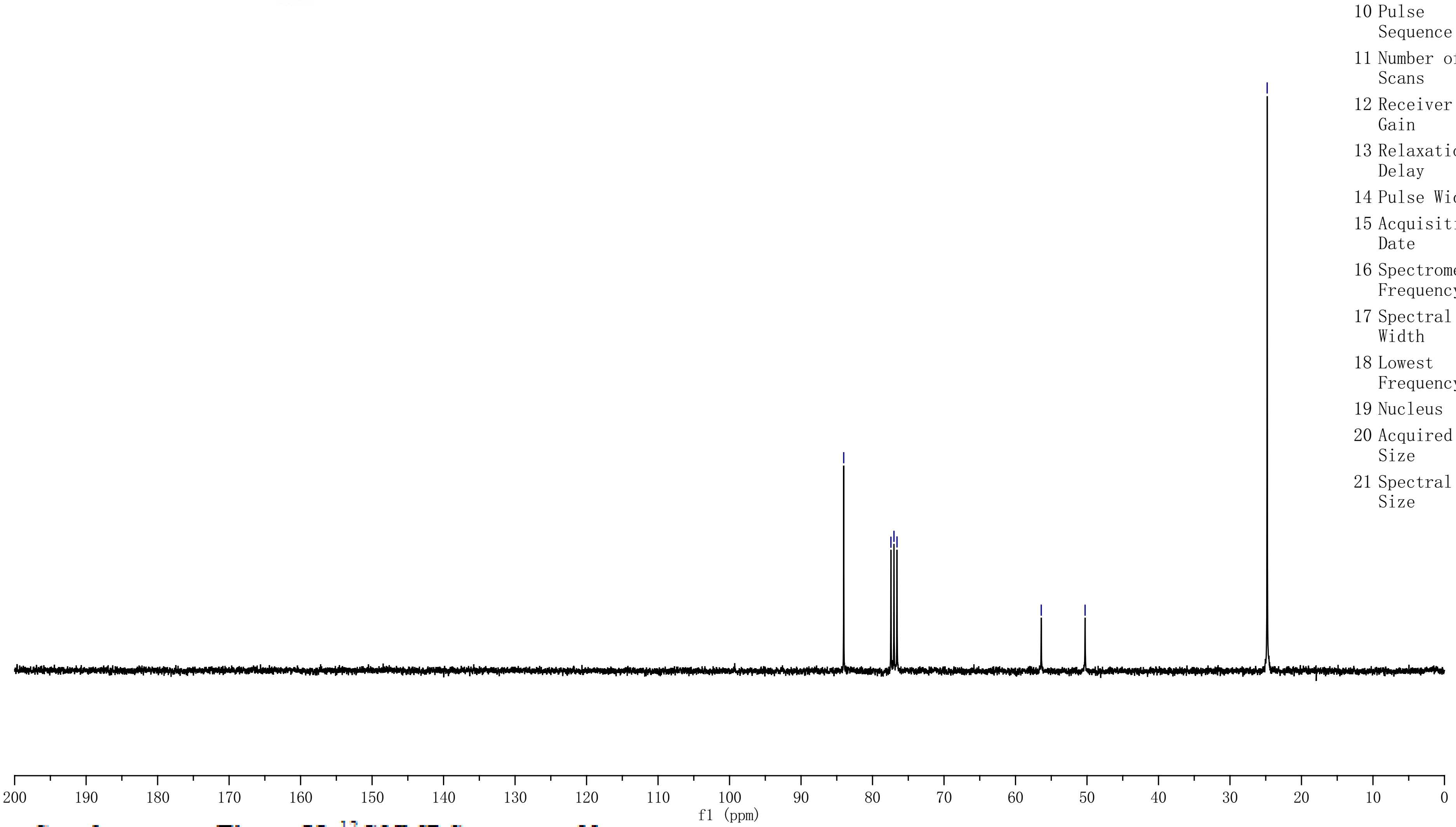

	Parameter	Value
1	Title	crf-3-48-C
2	Comment	13C OBSERVE
3	Origin	Varian
4	Owner	
5	Site	
6	Spectrometer	mercury
7	Author	omc
8	Solvent	CDCl3
9	Temperature	29.0
10	Pulse Sequence	s2pul
11	Number of Scans	124
12	Receiver Gain	34
13	Relaxation Delay	1.0000
14	Pulse Width	0.0000
15	Acquisition Date	2015-01-17T17:09:5
16	Spectrometer Frequency	75.45
17	Spectral Width	18797.0
18	Lowest Frequency	-1649.4
19	Nucleus	13C
20	Acquired Size	30075
21	Spectral Size	65536

Supplementary Figure 58. ^{13}C NMR Spectrum of **3c**.

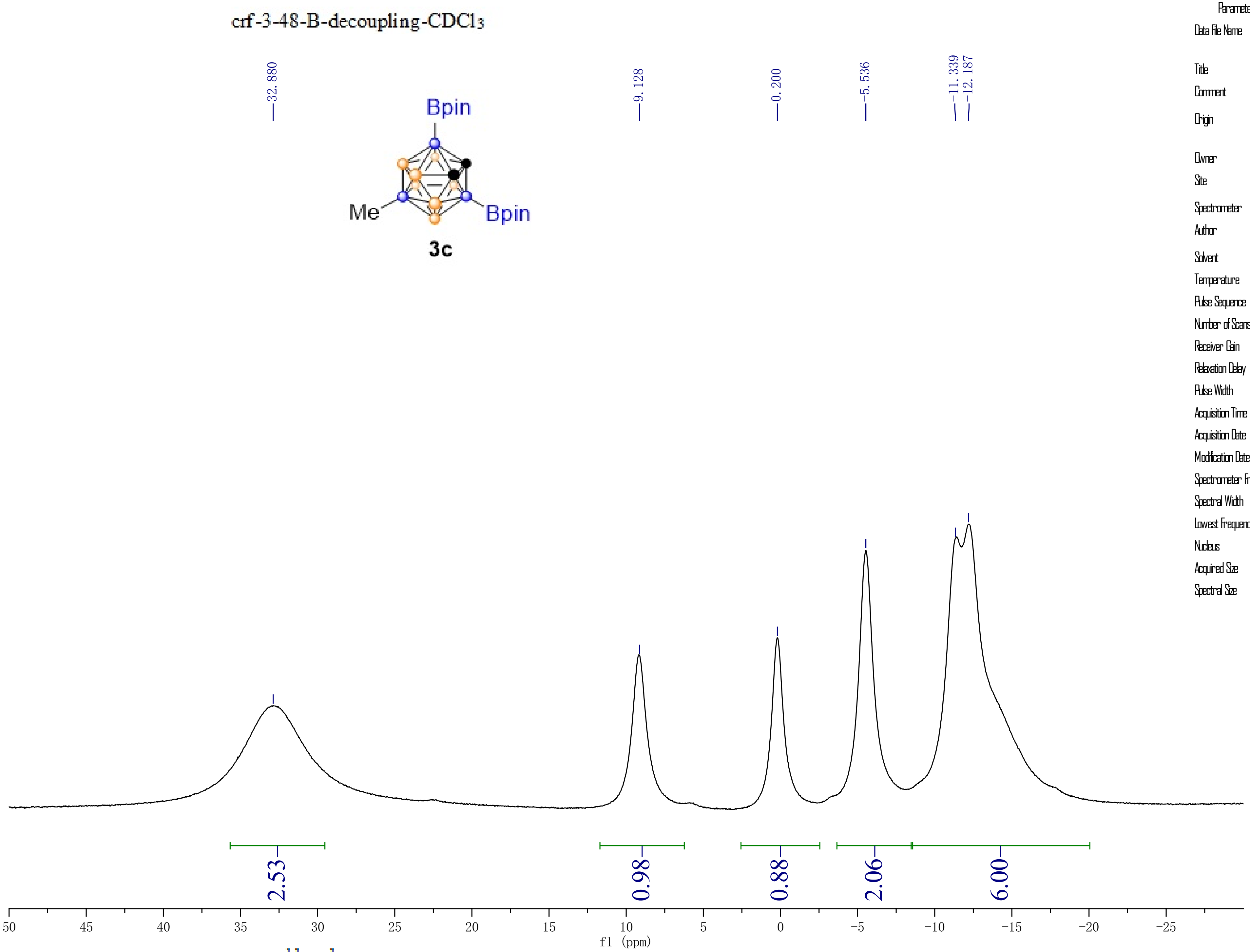

Parameter	Value
Data File Name	C:/Users/Administrator/Desktop/b-crf-3-48 (1)/fid
Title	Desktop
Comment	
Origin	UXNMR, Bruker Analytische Messtechnik GmbH
Owner	root
Site	
Spectrometer	drx400
Author	
Solvent	DMSO
Temperature	300.0
Pulse Sequence	aring.lgy
Number of Scans	364
Receiver Gain	32
Relaxation Delay	0.0000
Pulse Width	13.4000
Acquisition Time	0.3195
Acquisition Date	2015-01-21T19:00:31
Modification Date	2015-01-21T12:01:00
Spectrometer Frequency	128.38
Spectral Width	51282.1
Lowest Frequency	-17920.9
Nucleus	11B
Acquired Size	16384
Spectral Size	32768

Supplementary Figure 59. $^{11}B\{^{1}H\}$ NMR Spectrum of **3c**.

crf-3-48-B-coupling-$CDCl_3$

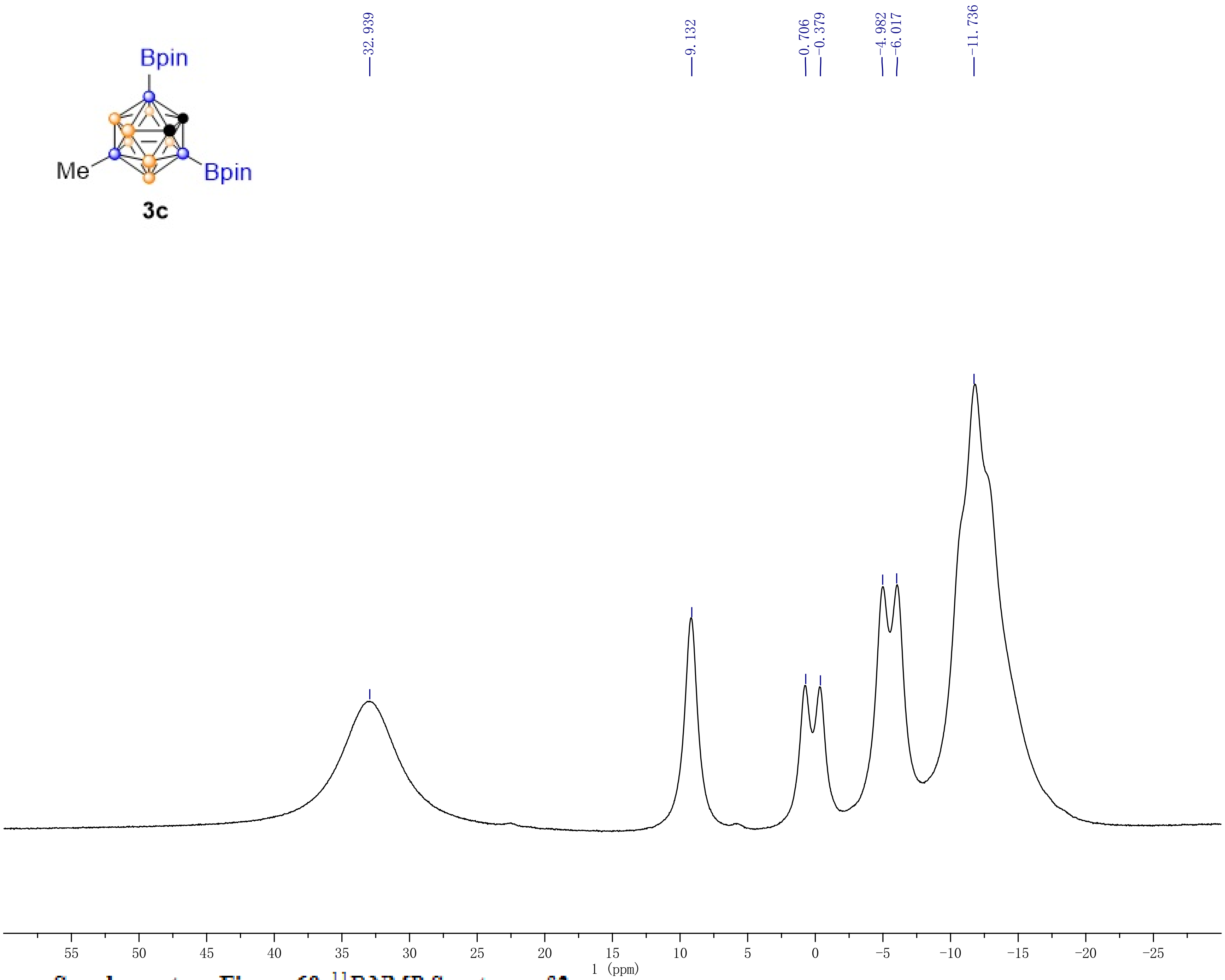

Parameter	Value
Data File Name	C:/Users/Administrator/Desktop/ b-crf-3-48-withoutdecoupling/fid
Title	Desktop
Comment	
Origin	UXNMR, Bruker Analytische Messtechnik GmbH
Owner	root
Site	
Spectrometer	drx400
Author	
Solvent	DMSO
Temperature	300.0
Pulse Sequence	aring.lgy
Number of Scans	540
Receiver Gain	32
Relaxation Delay	0.0000
Pulse Width	13.4000
Acquisition Time	0.3195
Acquisition Date	2015-01-27T22:34:37
Modification Date	2015-01-27T15:34:00
Spectrometer Frequency	128.38
Spectral Width	51282.1
Lowest Frequency	-16625.9
Nucleus	11B
Acquired Size	16384
Spectral Size	32768

Supplementary Figure 60. ^{11}B NMR Spectrum of **3c**.

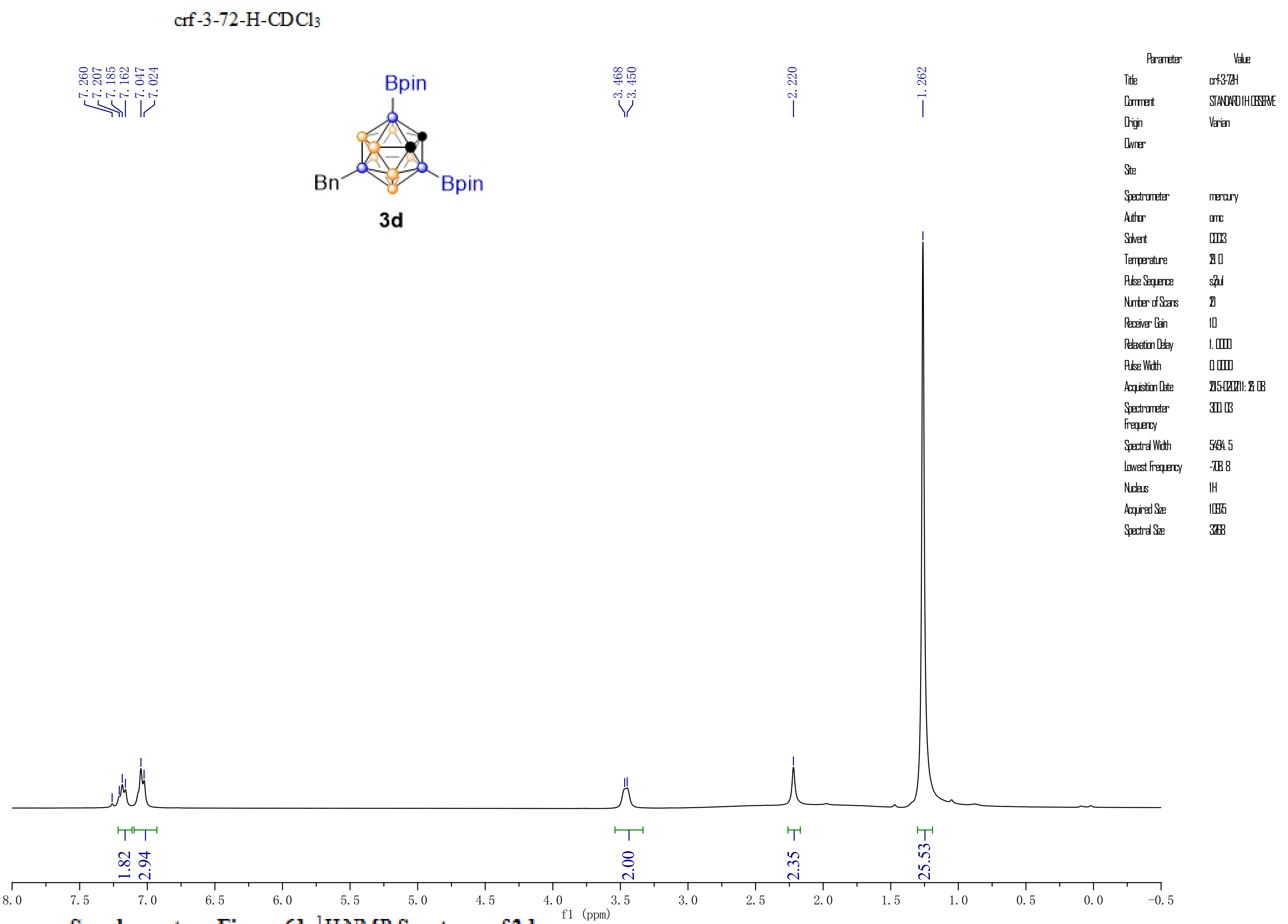

Supplementary Figure 61. ^{1}H NMR Spectrum of **3d**.

crf-3-72-C-CDCl3

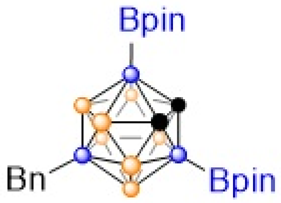

3d

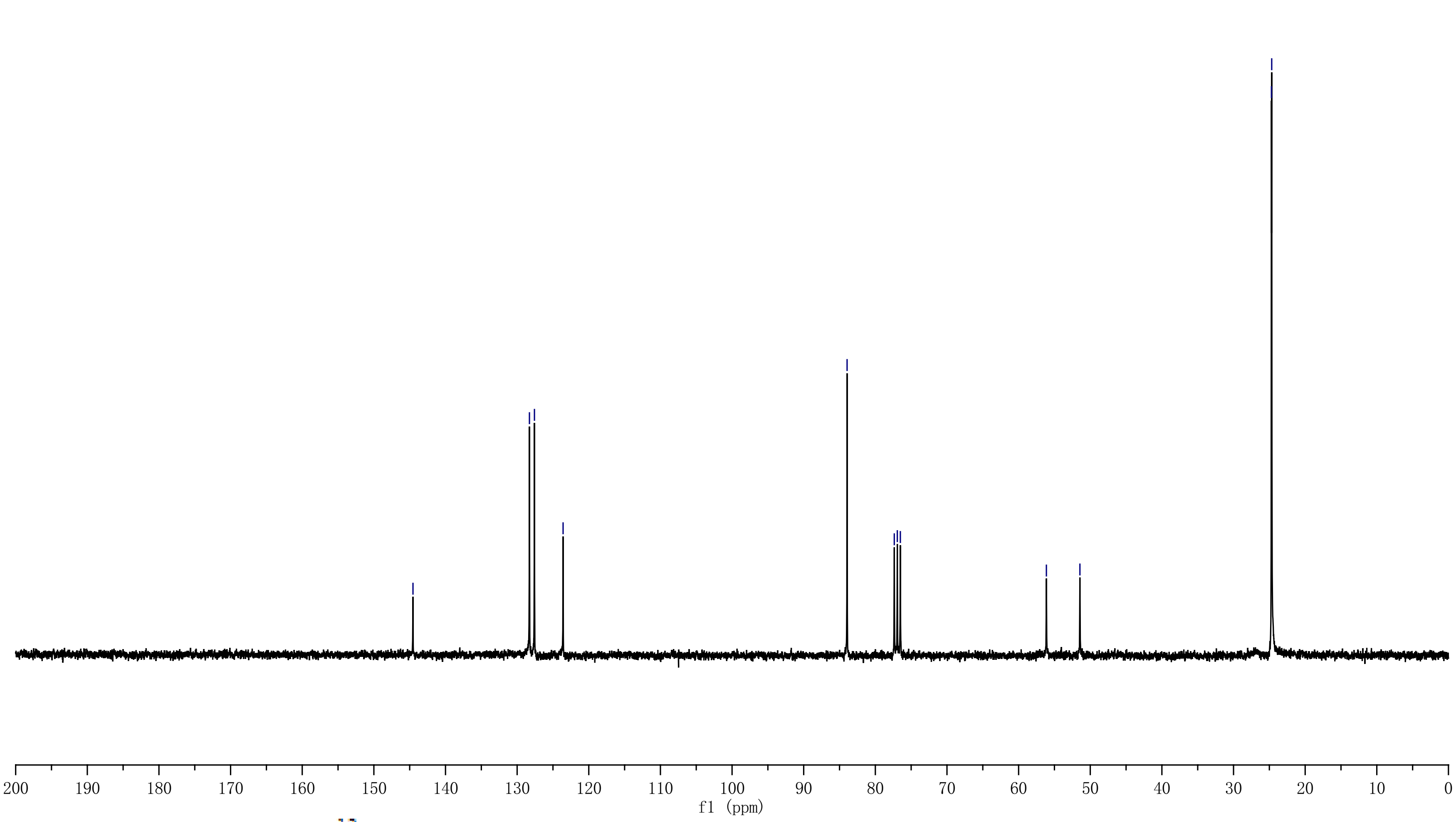

Parameter	Value
Title	[illegible]
Comment	13C OBSERVE
Origin	Varian
Owner	
Site	
Spectrometer	mercury
Author	omc
Solvent	[illegible]
Temperature	[illegible]
Pulse Sequence	s2pul
Number of Scans	132
Receiver Gain	34
Relaxation Delay	1.0000
Pulse Width	0.0000
Acquisition Date	[illegible]
Spectrometer Frequency	75.45
Spectral Width	18797.0
Lowest Frequency	-1659.2
Nucleus	13C
Acquired Size	[illegible]
Spectral Size	65536

Supplementary Figure 62. ^{13}C NMR Spectrum of **3d**.

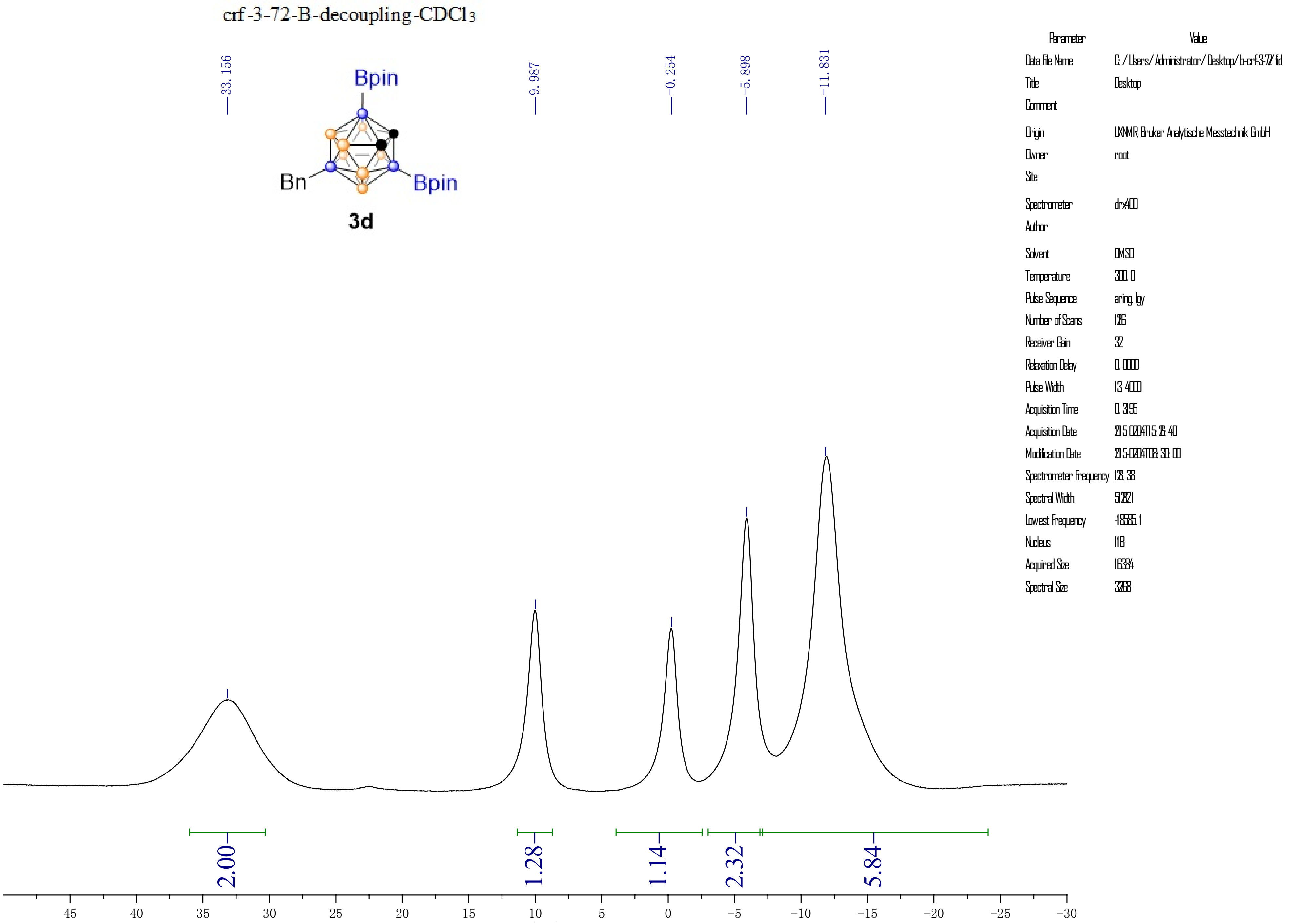

Supplementary Figure 63. $^{11}B\{^{1}H\}$ NMR Spectrum of **3d**.

crf-3-72-B-coupling-$CDCl_3$

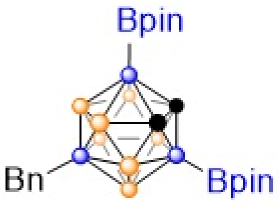

3d

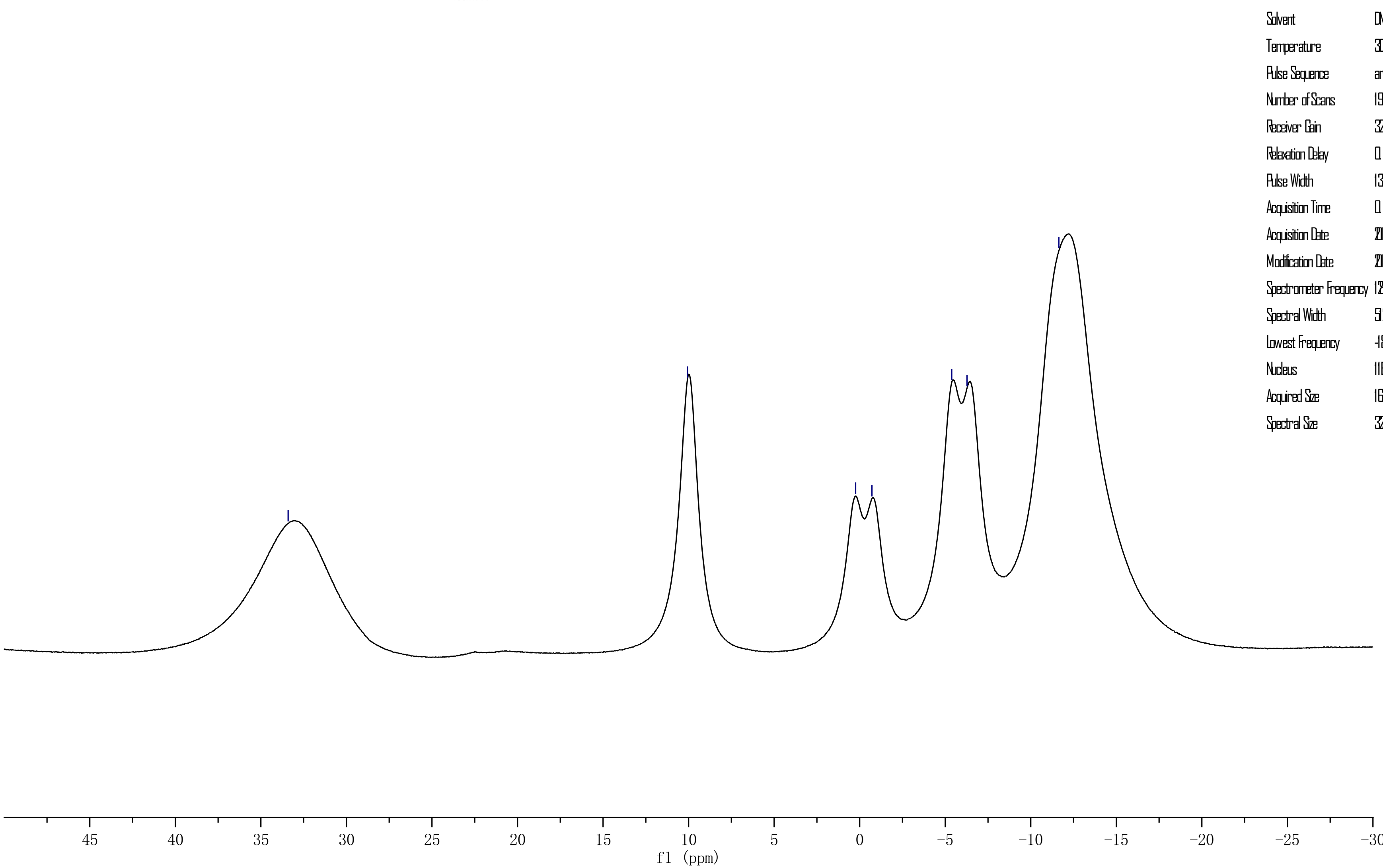

Parameter	Value
Data File Name	C:/Users/Administrator/Desktop/b-crf-3-72withoutdecoupling/fid
Title	Desktop
Comment	
Origin	UXNMR, Bruker Analytische Messtechnik GmbH
Owner	root
Site	
Spectrometer	drx400
Author	
Solvent	DMSO
Temperature	300.0
Pulse Sequence	aring.lgy
Number of Scans	1986
Receiver Gain	32
Relaxation Delay	0.0000
Pulse Width	13.4000
Acquisition Time	0.3195
Acquisition Date	2015-02-04T15:40:48
Modification Date	2015-02-04T08:40:00
Spectrometer Frequency	128.38
Spectral Width	51282.1
Lowest Frequency	-18585.1
Nucleus	11B
Acquired Size	16384
Spectral Size	32768

Supplementary Figure 64. ^{11}B NMR Spectrum of **3d**.

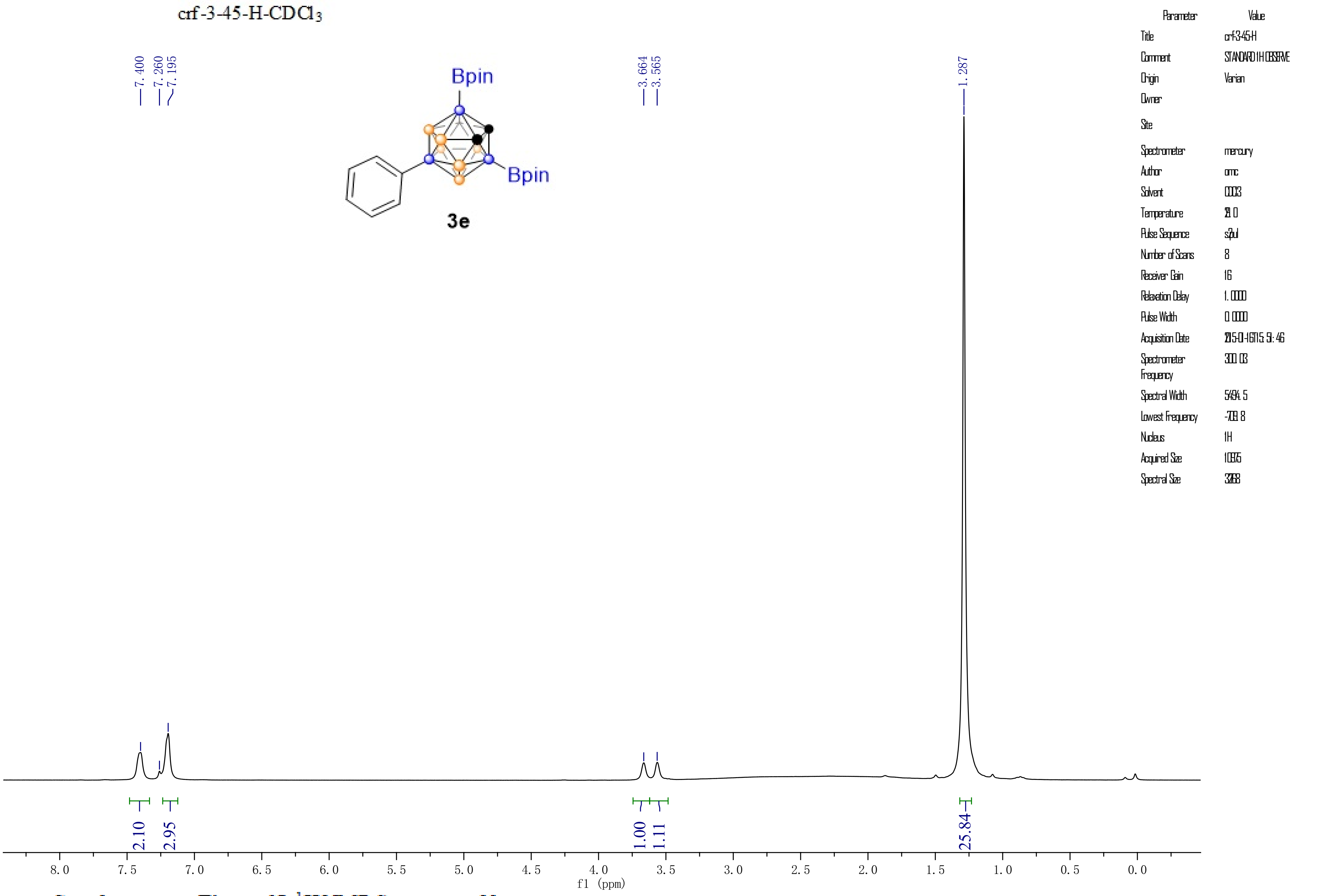

Supplementary Figure 65. ^{1}H NMR Spectrum of 3e.

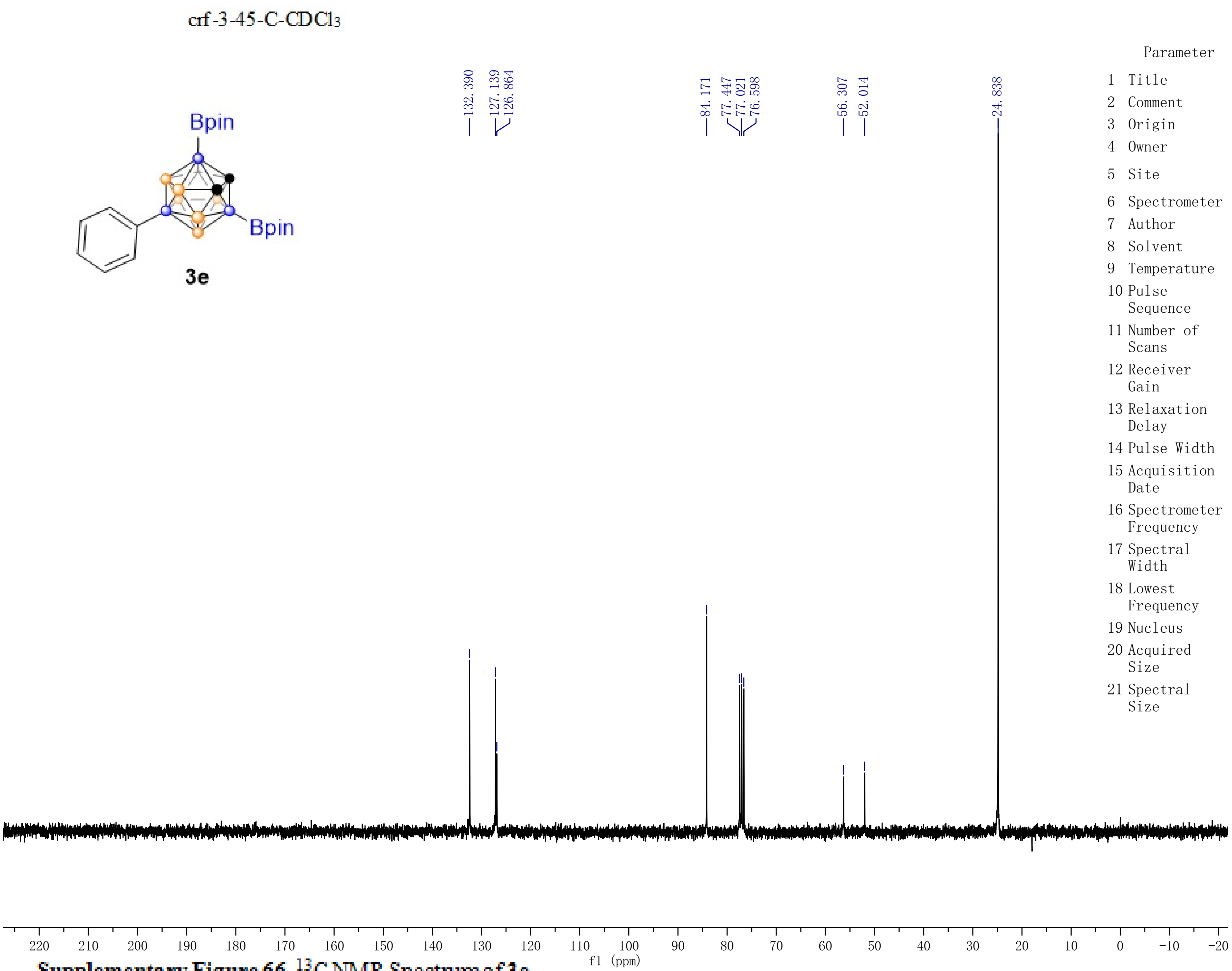

	Parameter	Value
1	Title	crf-3-45-C
2	Comment	13C OBSERVE
3	Origin	Varian
4	Owner	
5	Site	
6	Spectrometer	mercury
7	Author	omc
8	Solvent	CDCl3
9	Temperature	29.0
10	Pulse Sequence	s2pul
11	Number of Scans	148
12	Receiver Gain	34
13	Relaxation Delay	1.0000
14	Pulse Width	0.0000
15	Acquisition Date	2015-01-16T15:52:36
16	Spectrometer Frequency	75.45
17	Spectral Width	18797.0
18	Lowest Frequency	-1649.4
19	Nucleus	13C
20	Acquired Size	30075
21	Spectral Size	65536

Supplementary Figure 66. ^{13}C NMR Spectrum of **3e**.

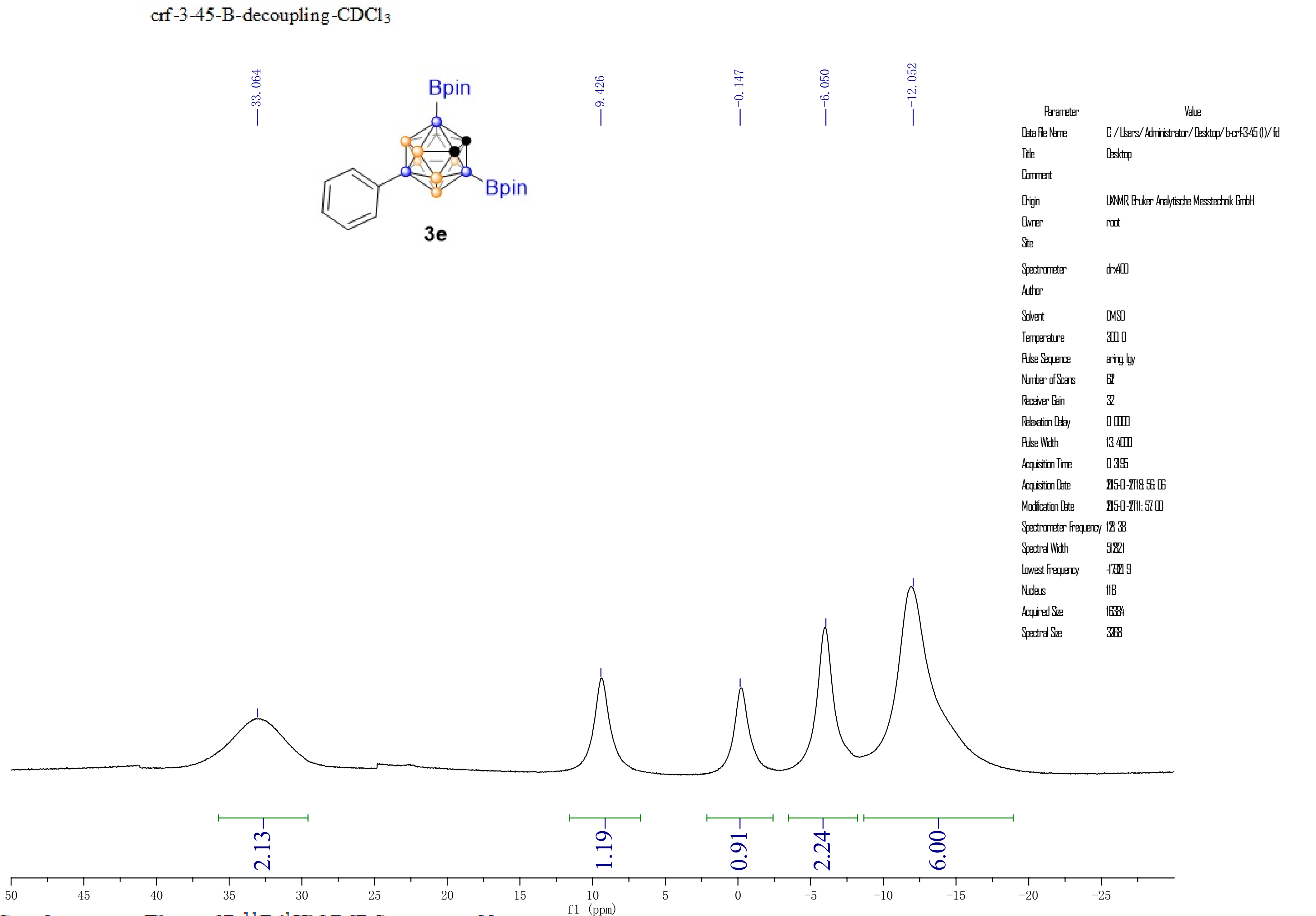

Supplementary Figure 67. $^{11}B\{^{1}H\}$ NMR Spectrum of **3e**.

crf-3-45-B-coupling-$CDCl_3$

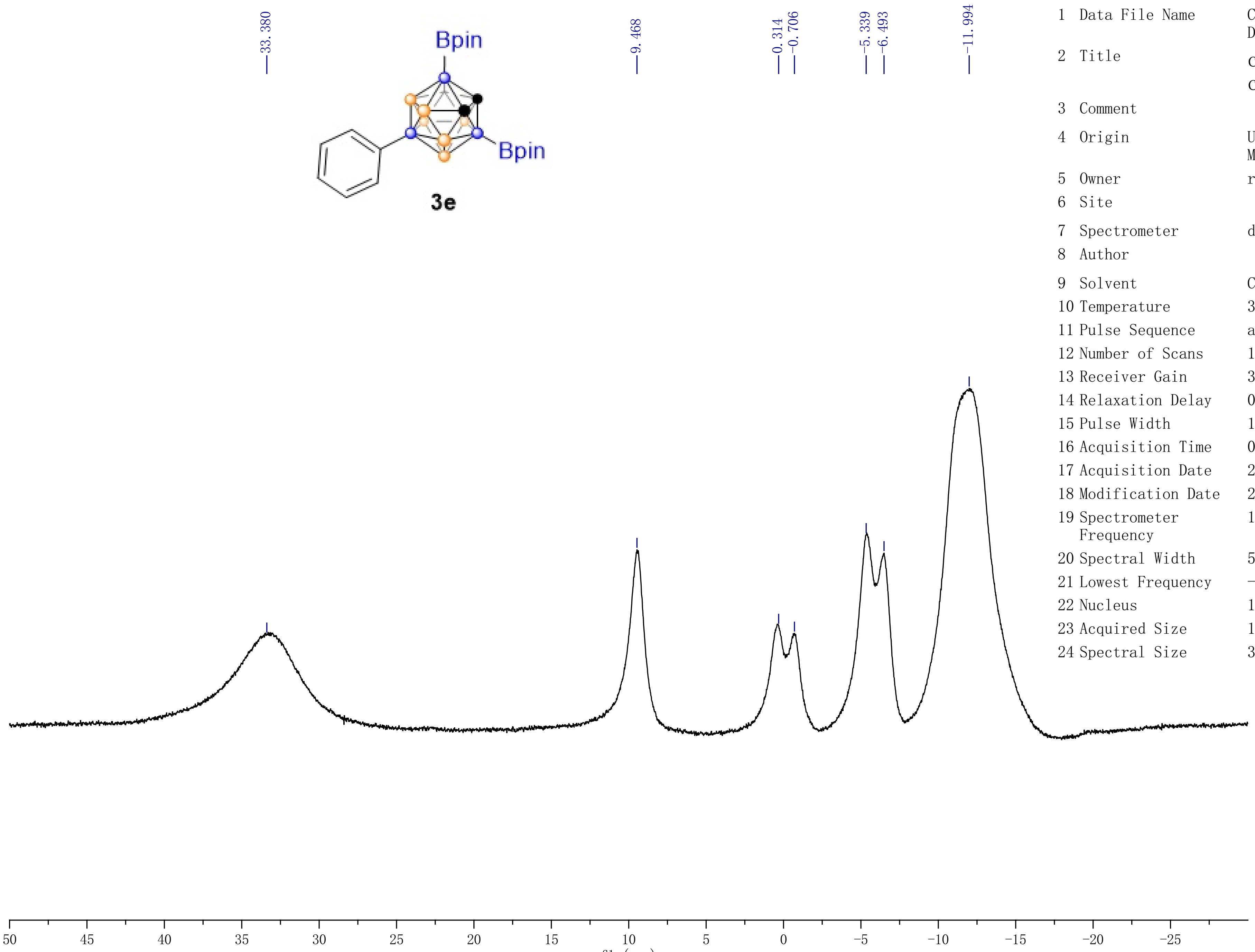

	Parameter	Value
1	Data File Name	C:/ Users/ Administrator/ Desktop/ b-crf-3-45/ fid
2	Title	crf-3-45-B-coupling-CDCl3
3	Comment	
4	Origin	UXNMR, Bruker Analytische Messtechnik GmbH
5	Owner	root
6	Site	
7	Spectrometer	drx400
8	Author	
9	Solvent	CDCl3
10	Temperature	300.0
11	Pulse Sequence	aring.lgy
12	Number of Scans	1600
13	Receiver Gain	32
14	Relaxation Delay	0.0000
15	Pulse Width	13.4000
16	Acquisition Time	0.3195
17	Acquisition Date	2015-03-09T15:57:45
18	Modification Date	2015-03-09T08:57:00
19	Spectrometer Frequency	128.38
20	Spectral Width	51282.1
21	Lowest Frequency	-15466.9
22	Nucleus	11B
23	Acquired Size	16384
24	Spectral Size	32768

Supplementary Figure 68. ^{11}B NMR Spectrum of **3e**.

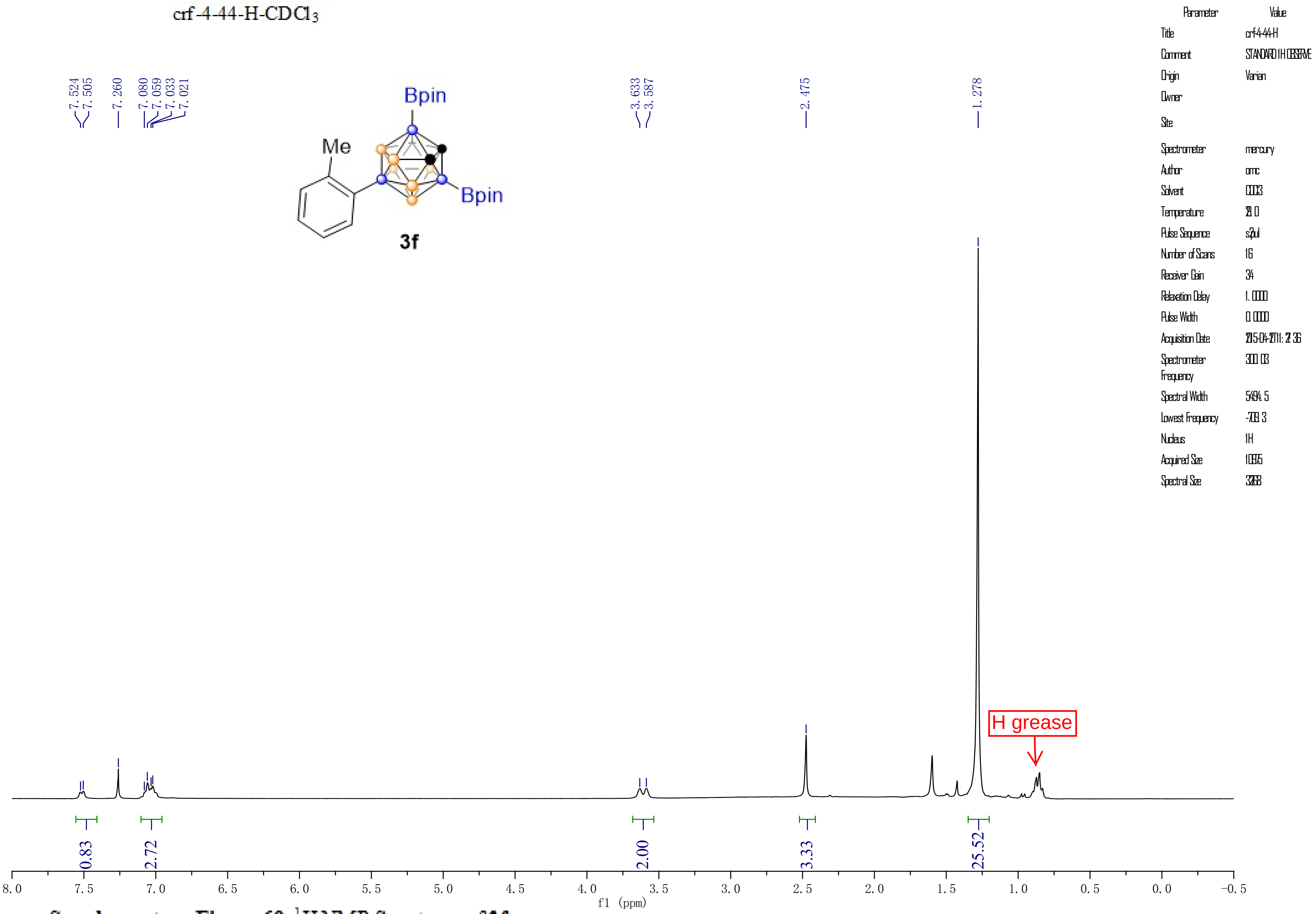

Supplementary Figure 69. ^{1}H NMR Spectrum of **3f**.

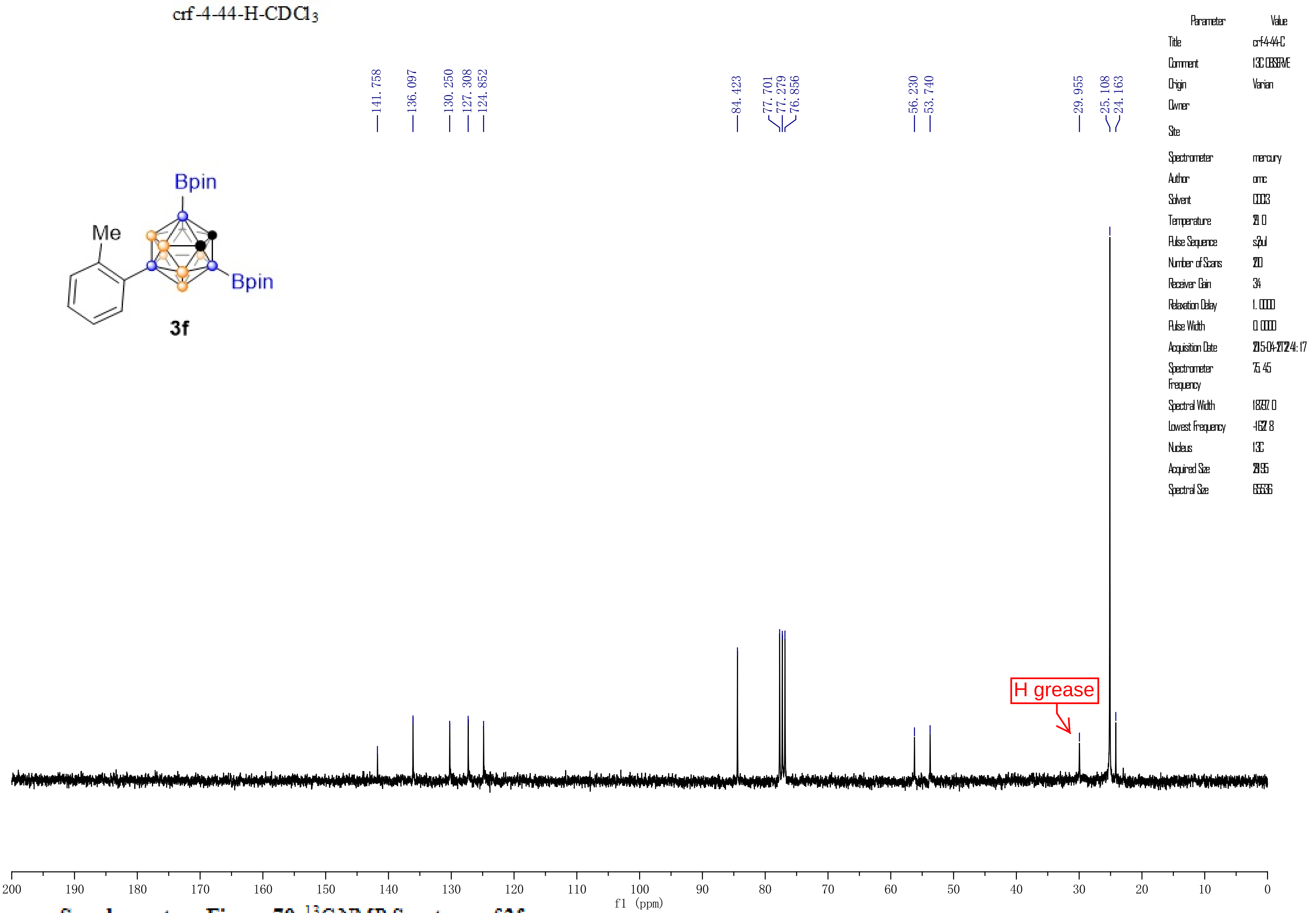

Supplementary Figure 70. ^{13}C NMR Spectrum of **3f**.

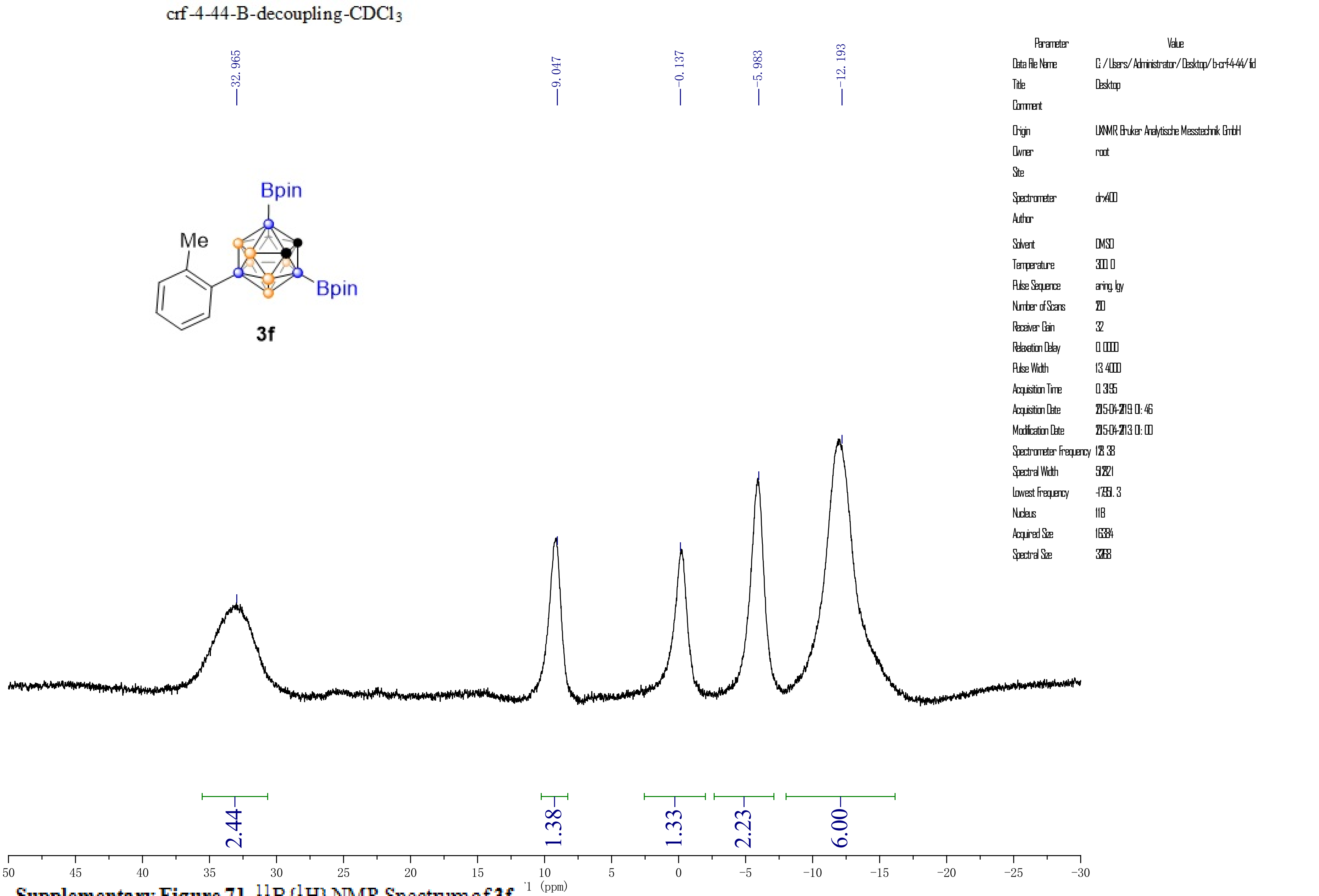

Supplementary Figure 71. $^{11}B\{^1H\}$ NMR Spectrum of **3f**.

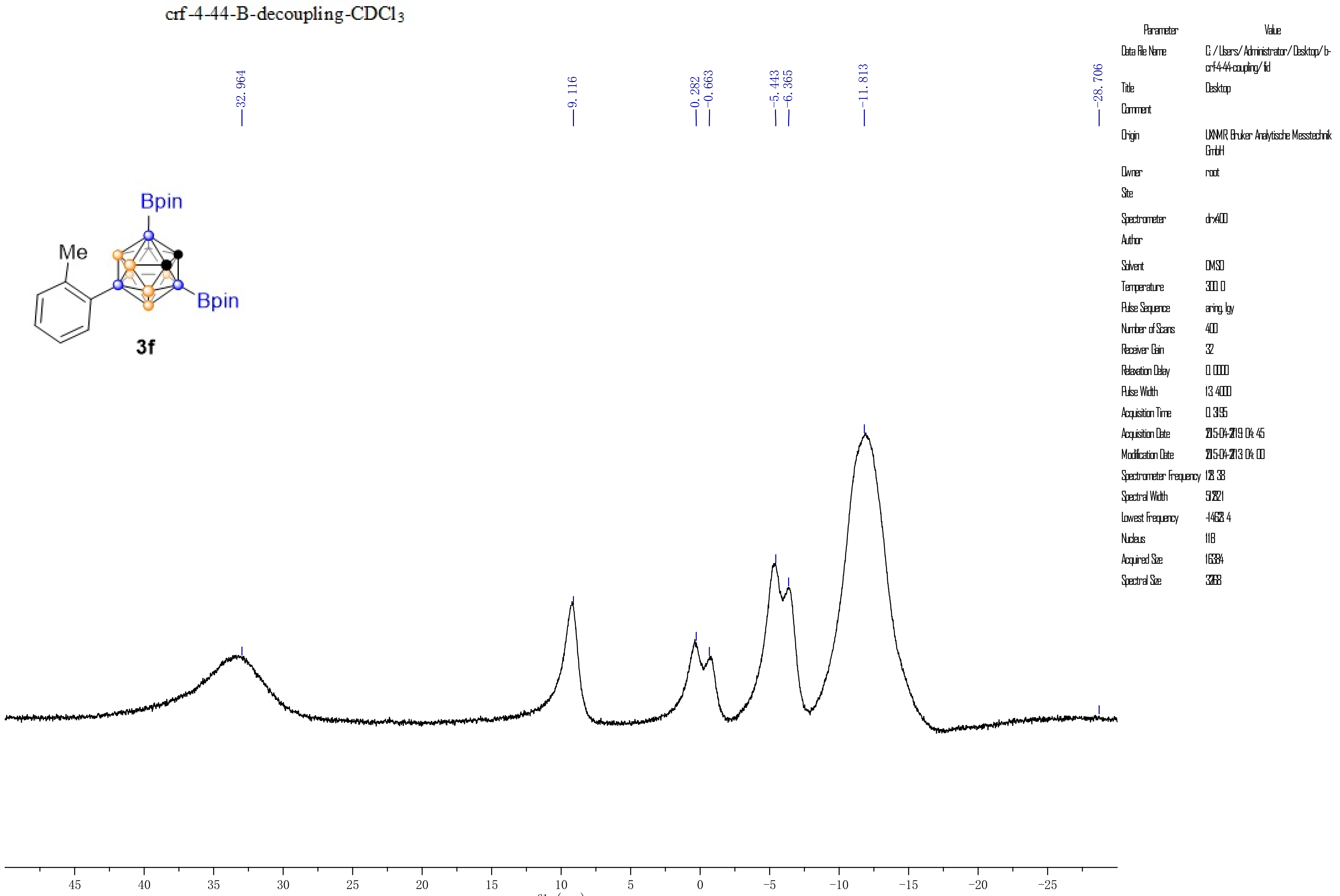

Supplementary Figure 72. ^{11}B NMR Spectrum of **3f**.

crf-3-90-H-$CDCl_3$

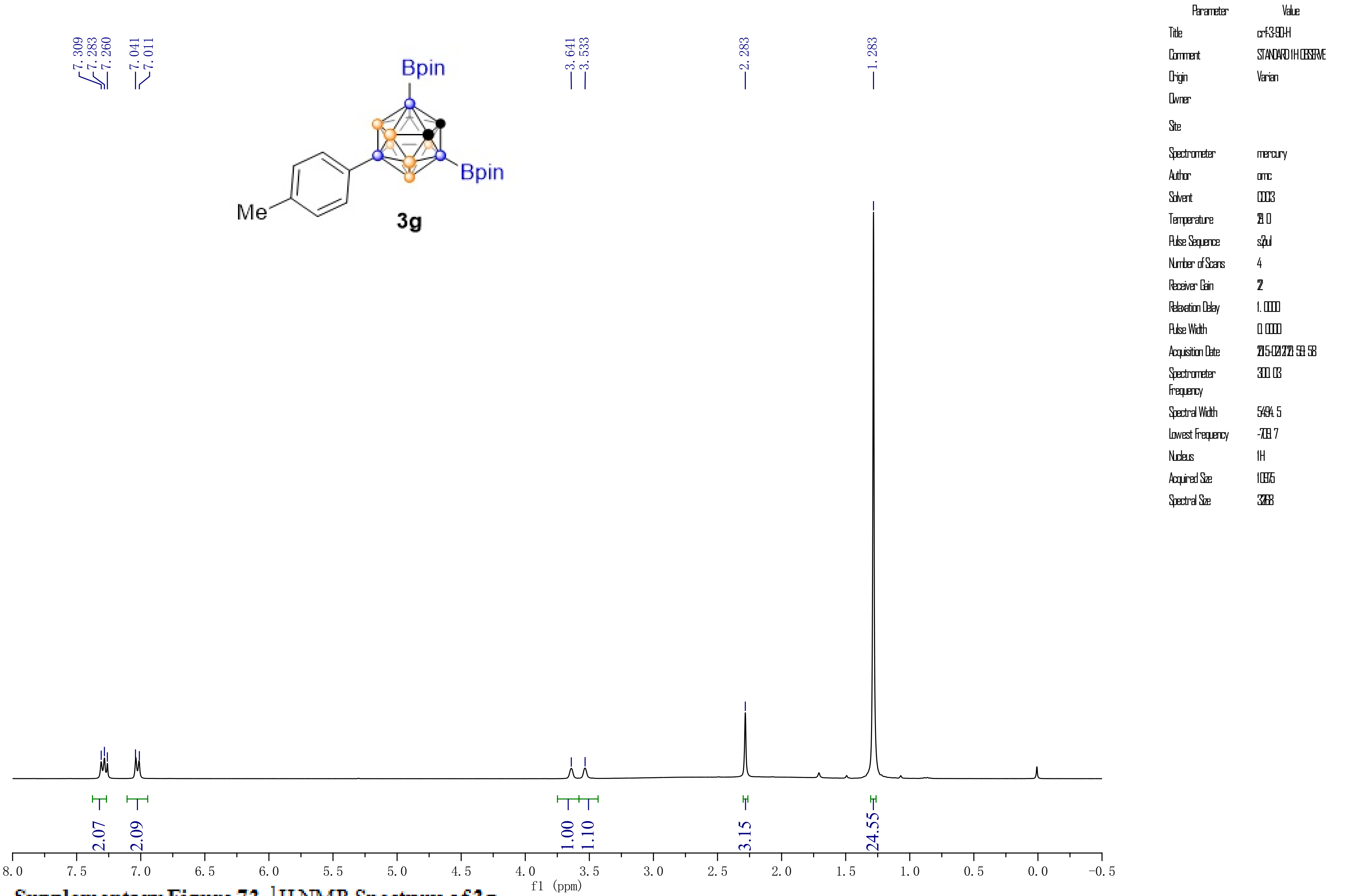

Supplementary Figure 73. ^{1}H NMR Spectrum of **3g**.

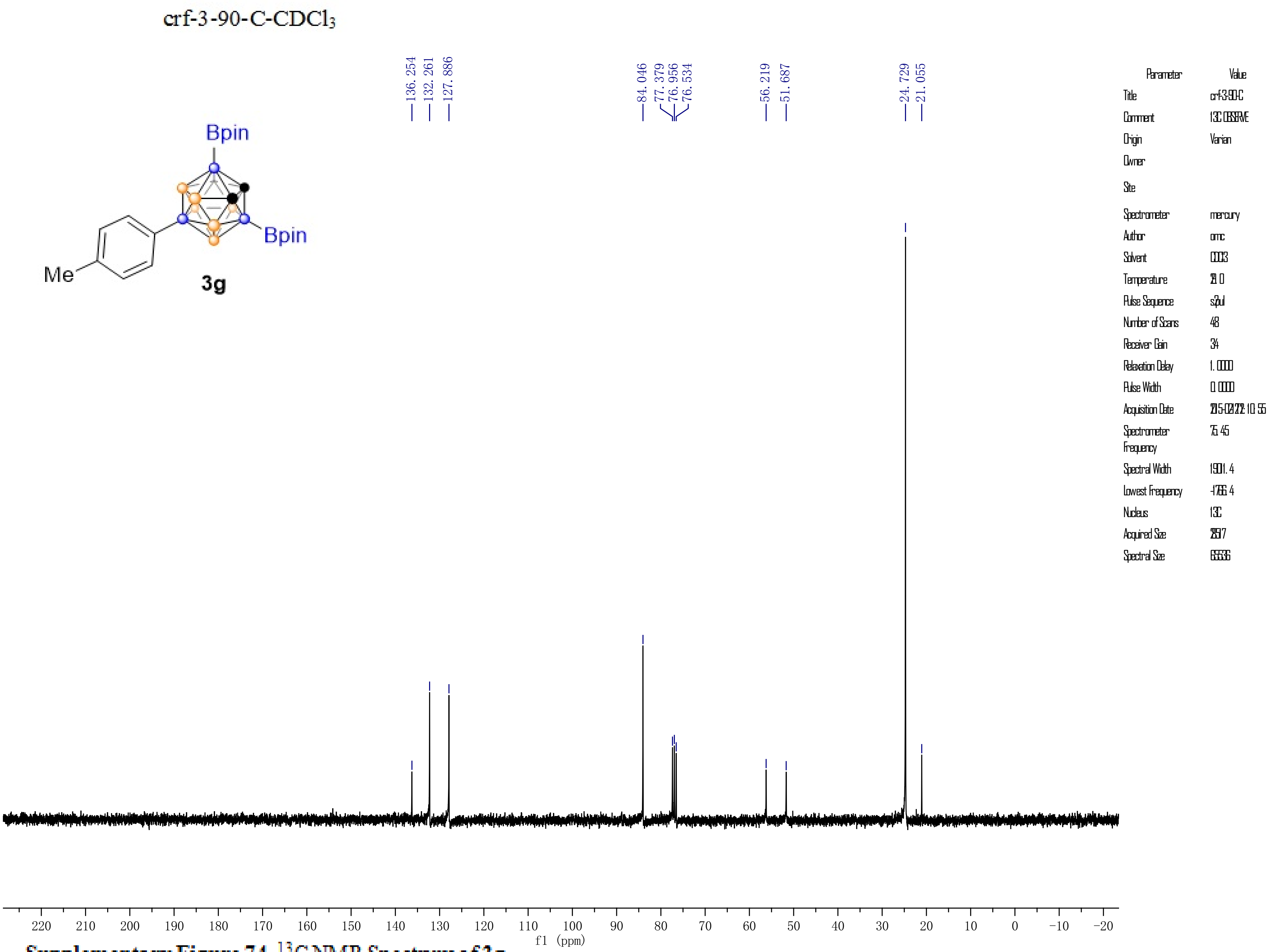

Supplementary Figure 74. ^{13}C NMR Spectrum of **3g**.

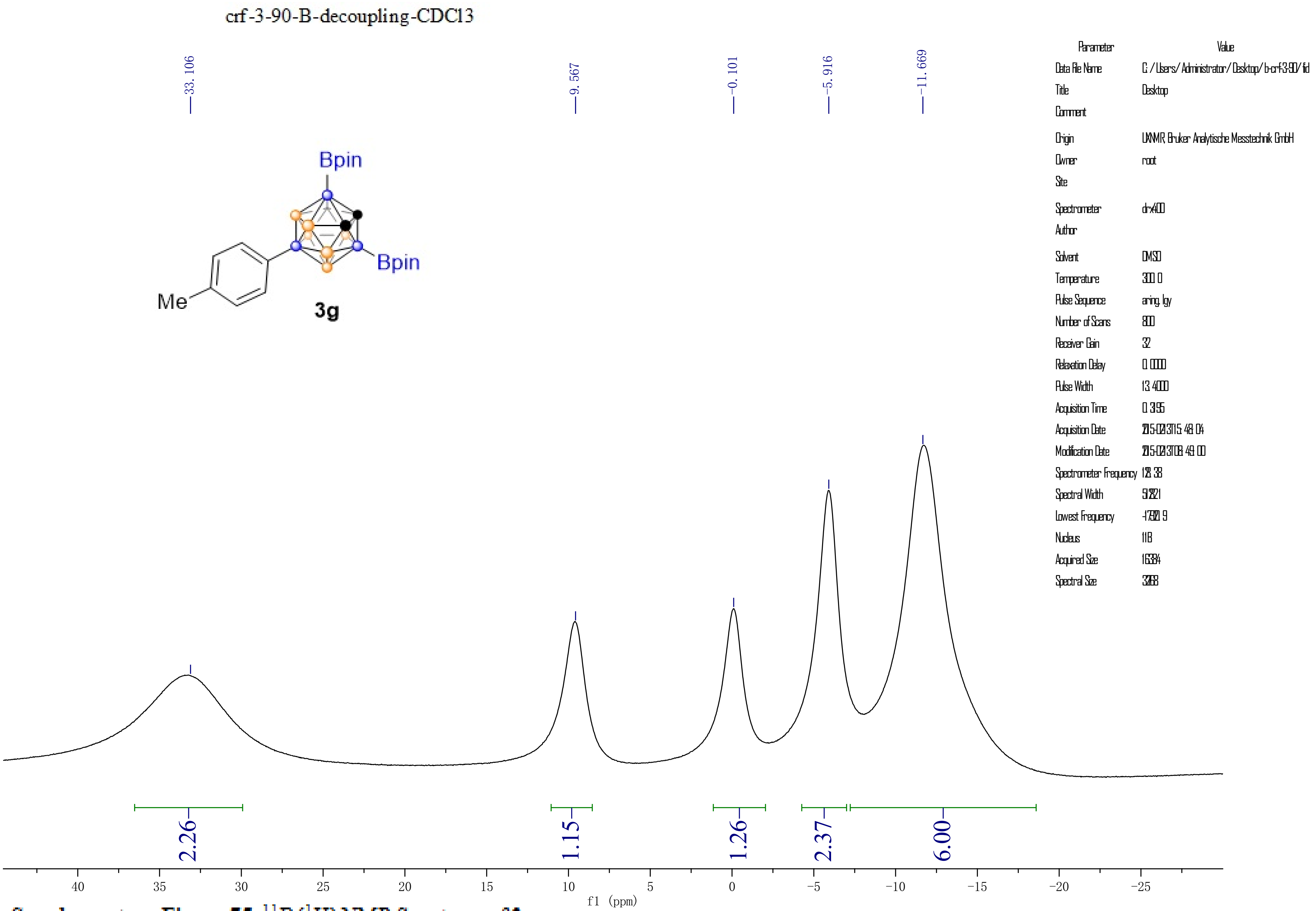

Supplementary Figure 75. $^{11}B\{^1H\}$ NMR Spectrum of **3g**.

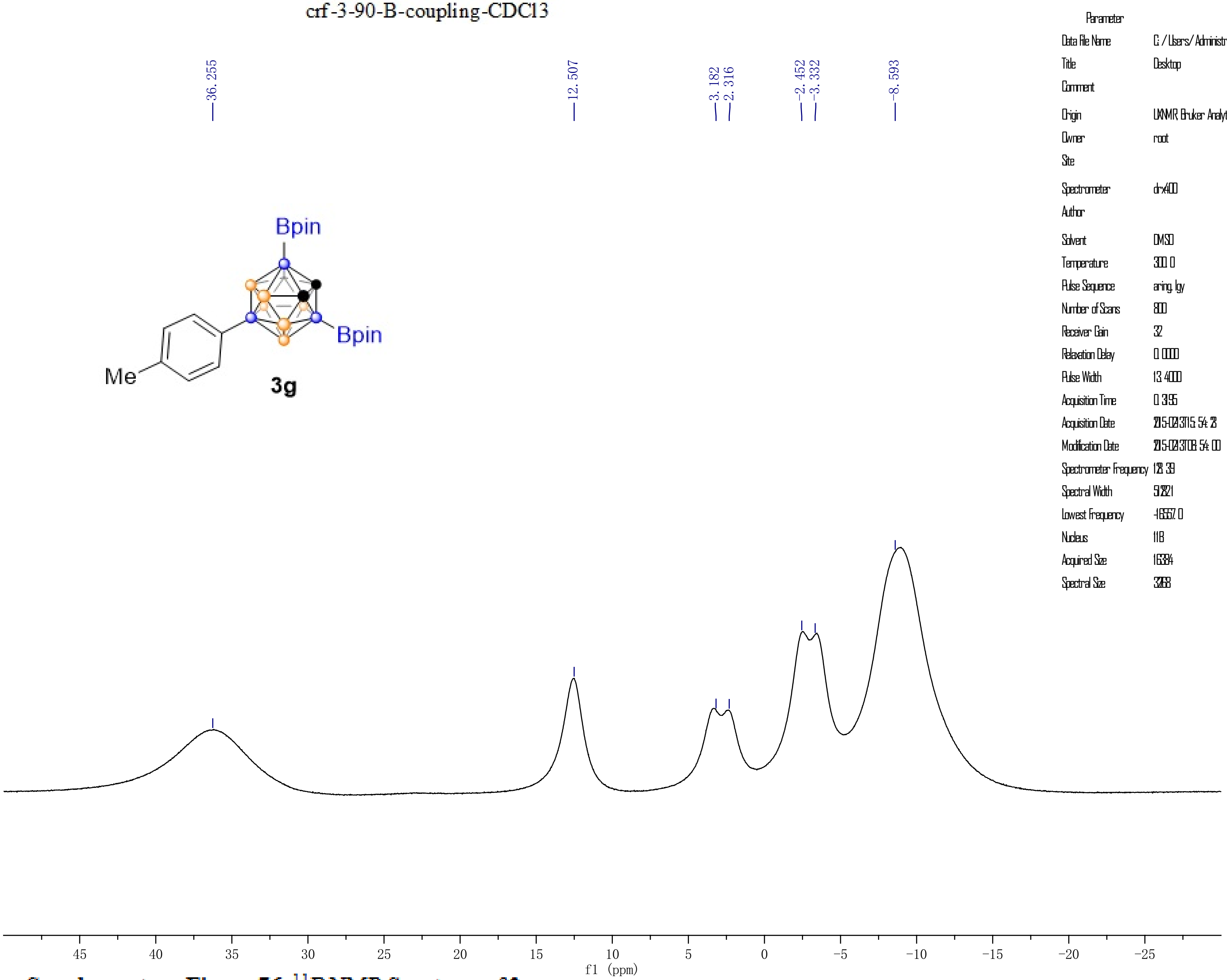

Parameter	Value
Data File Name	C:/Users/Administrator/Desktop/b-crf-3-90-withoutdecoupling/fid
Title	Desktop
Comment	
Origin	UXNMR, Bruker Analytische Messtechnik GmbH
Owner	root
Site	
Spectrometer	drx400
Author	
Solvent	DMSO
Temperature	300.0
Pulse Sequence	aring.lgy
Number of Scans	800
Receiver Gain	32
Relaxation Delay	0.0000
Pulse Width	13.4000
Acquisition Time	0.3195
Acquisition Date	2015-02-13T15:54:28
Modification Date	2015-02-13T08:54:00
Spectrometer Frequency	128.39
Spectral Width	51282.1
Lowest Frequency	-16557.0
Nucleus	11B
Acquired Size	16384
Spectral Size	32768

Supplementary Figure 76. ^{11}B NMR Spectrum of **3g**.

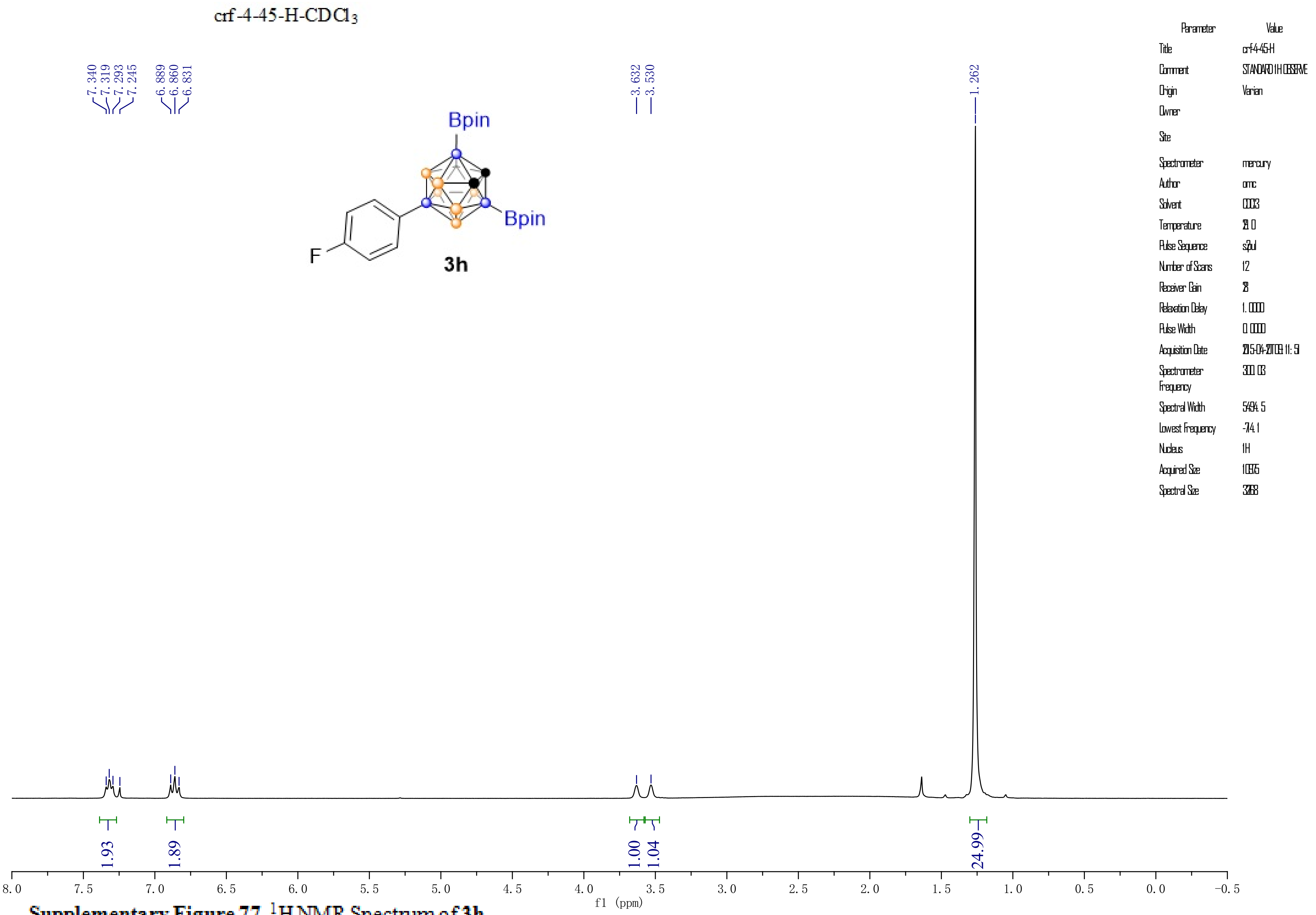

Supplementary Figure 77. ^{1}H NMR Spectrum of **3h**.

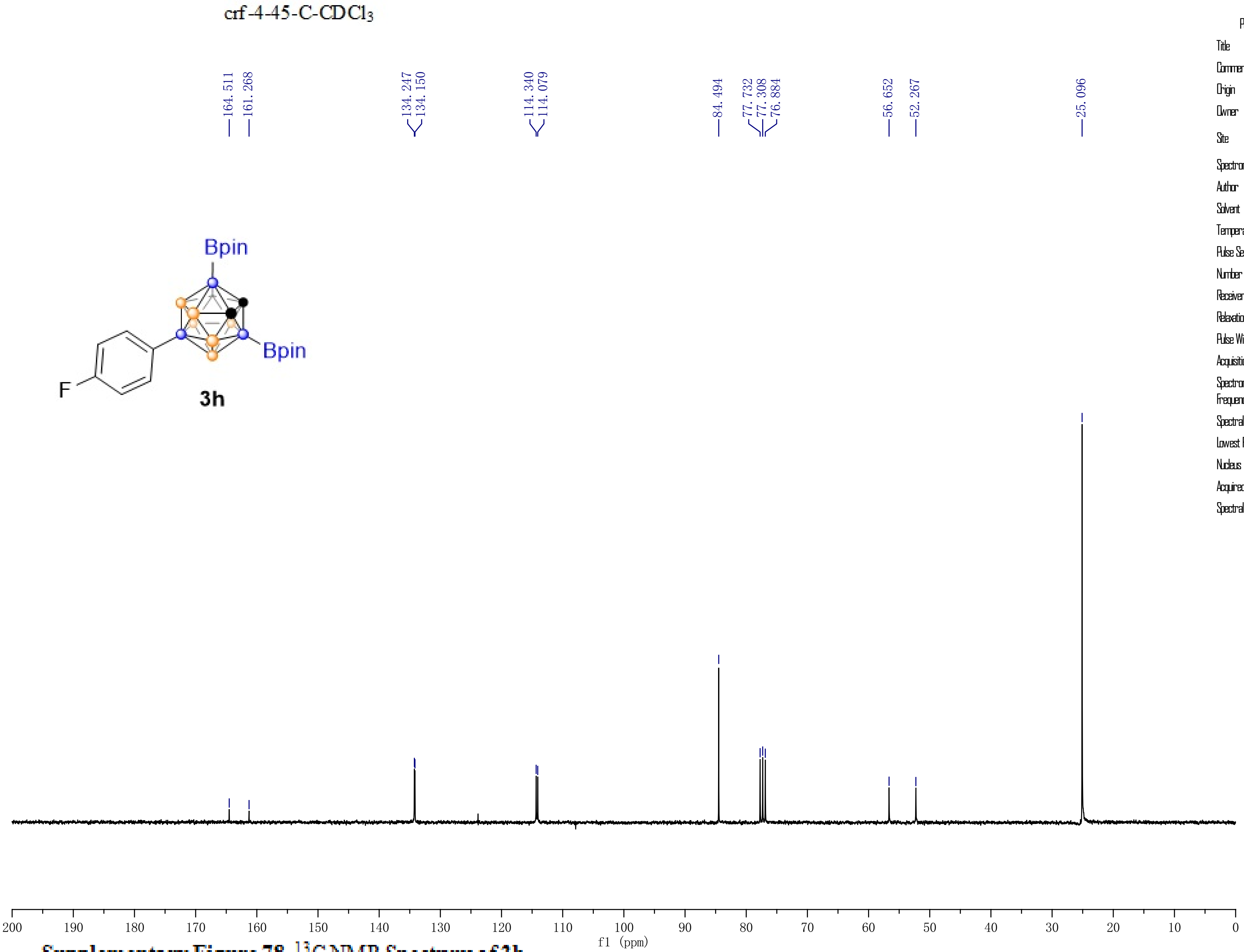

Parameter	Value
Title	crf-4-45-C-0420
Comment	13C OBSERVE
Origin	Varian
Owner	
Site	
Spectrometer	mercury
Author	omc
Solvent	CDCl3
Temperature	21.0
Pulse Sequence	s2pul
Number of Scans	200
Receiver Gain	34
Relaxation Delay	1.0000
Pulse Width	0.0000
Acquisition Date	2015-04-20T11:24:56
Spectrometer Frequency	75.45
Spectral Width	18797.0
Lowest Frequency	-1627.8
Nucleus	13C
Acquired Size	28195
Spectral Size	65536

Supplementary Figure 78. ^{13}C NMR Spectrum of **3h**.

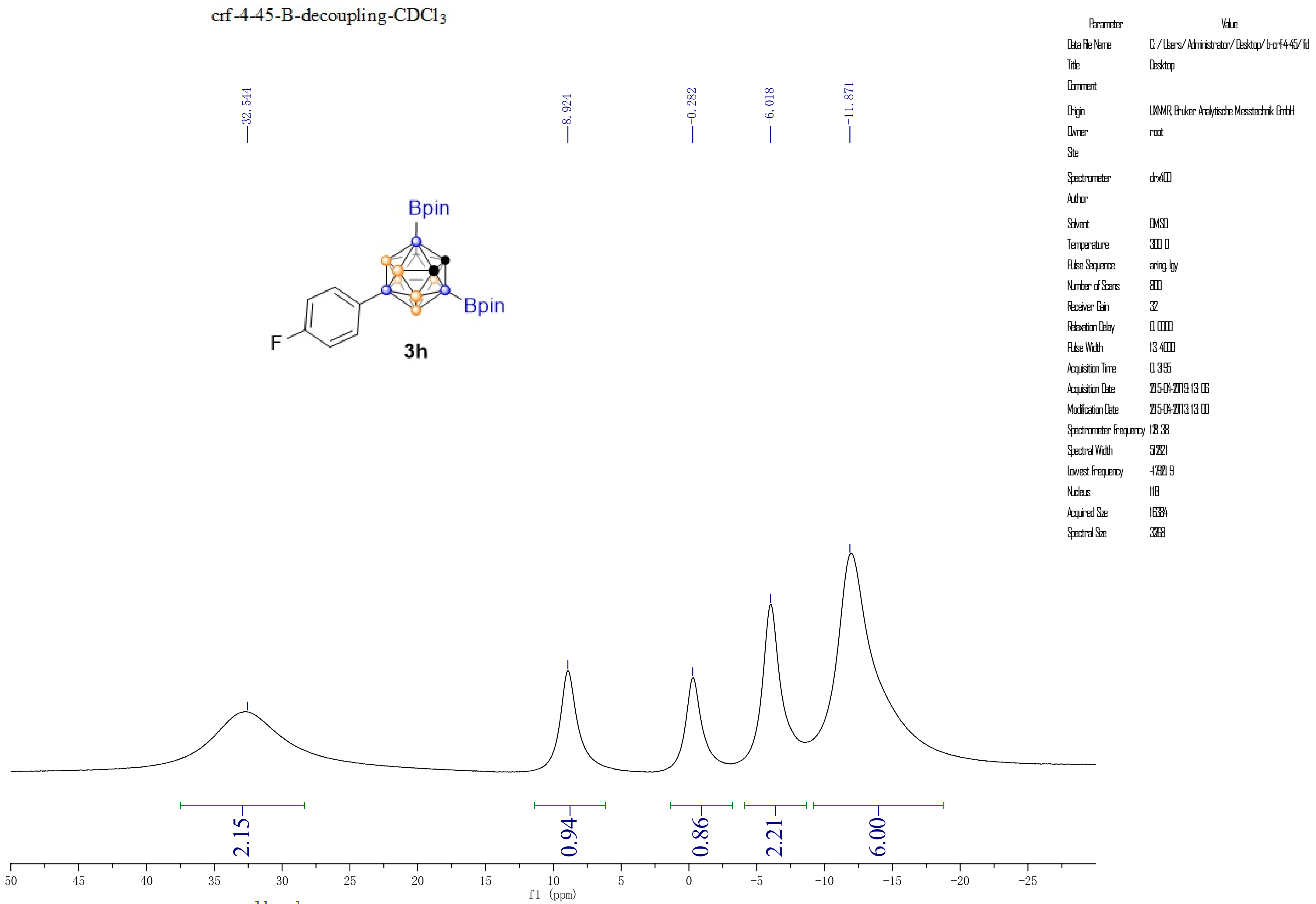

Supplementary Figure 79. $^{11}B\{^1H\}$ NMR Spectrum of **3h**.

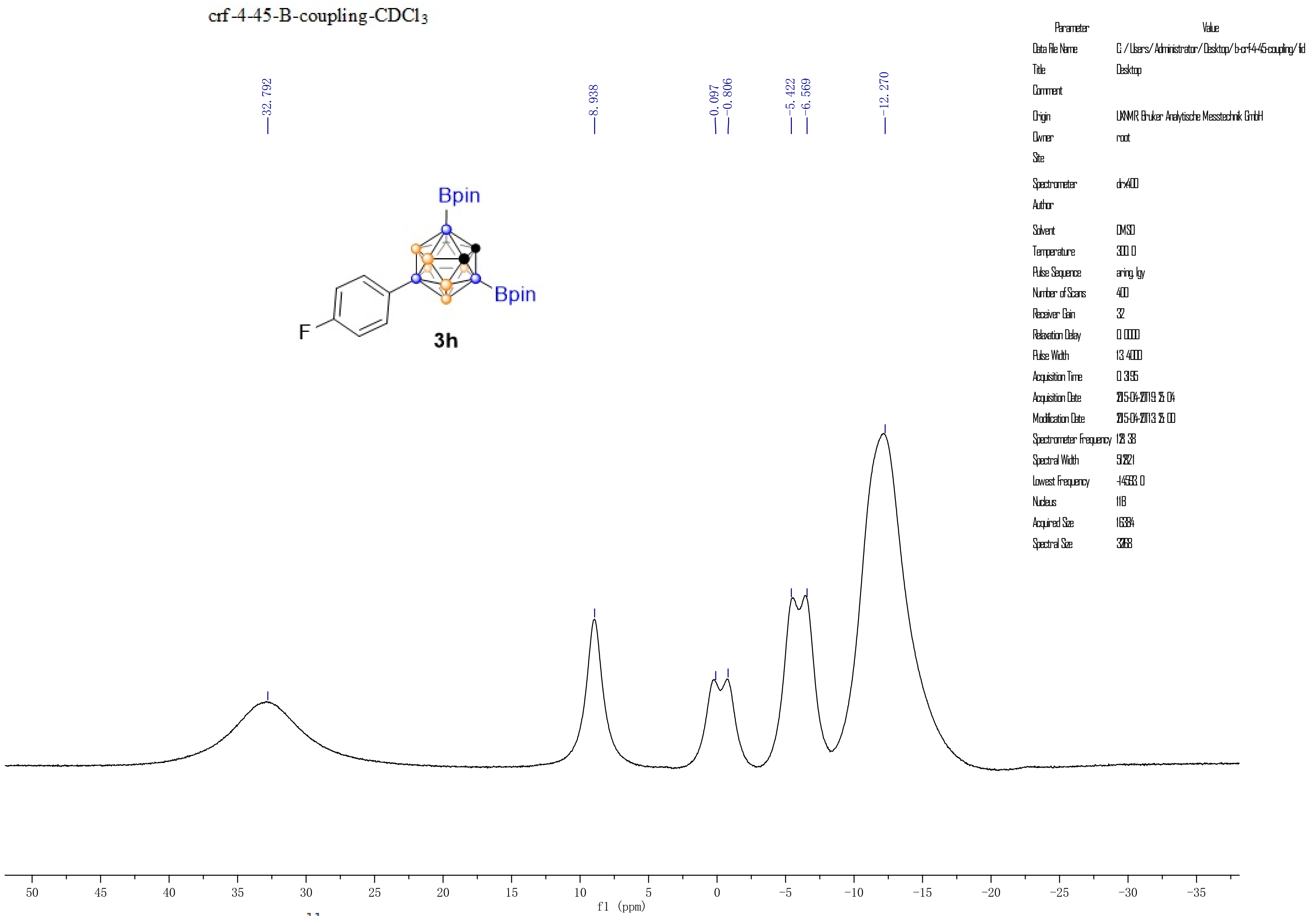

Supplementary Figure 80. ^{11}B NMR Spectrum of **3h**.

crf-4-45-F-$CDCl_3$

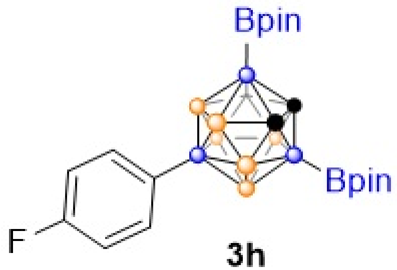

3h

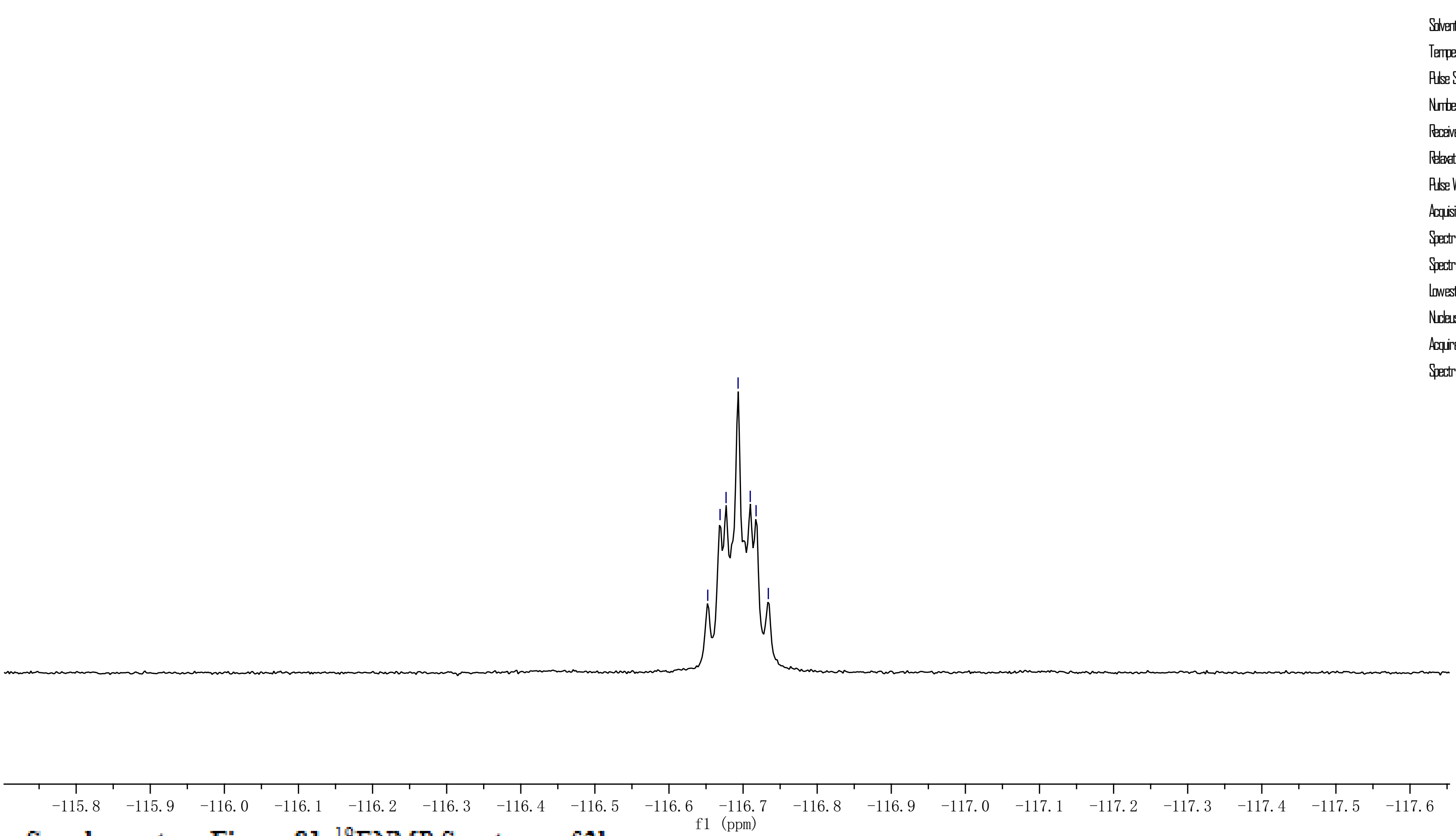

Parameter	Value
Title	[illegible]-CRF-4-45_FLUORINE_01
Comment	[illegible]-CRF-4-45
Origin	Varian
Owner	
Site	
Spectrometer	vnmrs
Author	
Solvent	cdcl3
Temperature	25.0
Pulse Sequence	s2pul
Number of Scans	16
Receiver Gain	56
Relaxation Delay	1.0000
Pulse Width	0.0000
Acquisition Date	[illegible]
Spectrometer Frequency	376.06
Spectral Width	89285.7
Lowest Frequency	[illegible]
Nucleus	19F
Acquired Size	65536
Spectral Size	131072

Supplementary Figure 81. ^{19}F NMR Spectrum of **3h**.

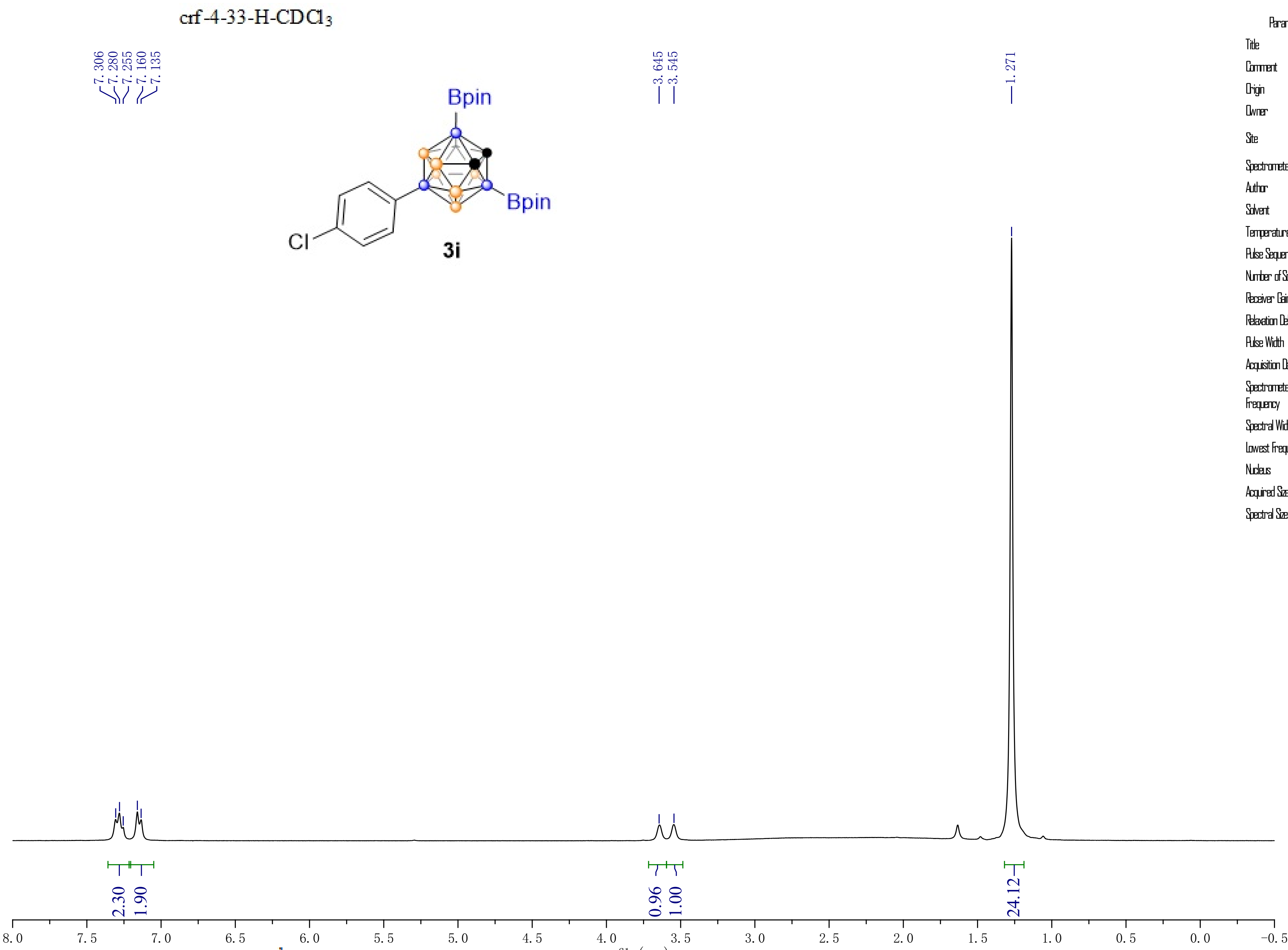

Parameter	Value
Title	crf4-33
Comment	STANDARD 1H OBSERVE
Origin	Varian
Owner	
Site	
Spectrometer	mercury
Author	omc
Solvent	CDCl3
Temperature	28.0
Pulse Sequence	s2pul
Number of Scans	8
Receiver Gain	28
Relaxation Delay	1.0000
Pulse Width	0.0000
Acquisition Date	2015-04-10T09:56:28
Spectrometer Frequency	300.03
Spectral Width	5494.5
Lowest Frequency	-711.1
Nucleus	1H
Acquired Size	10975
Spectral Size	32768

Supplementary Figure 82. ^{1}H NMR Spectrum of **3i**.

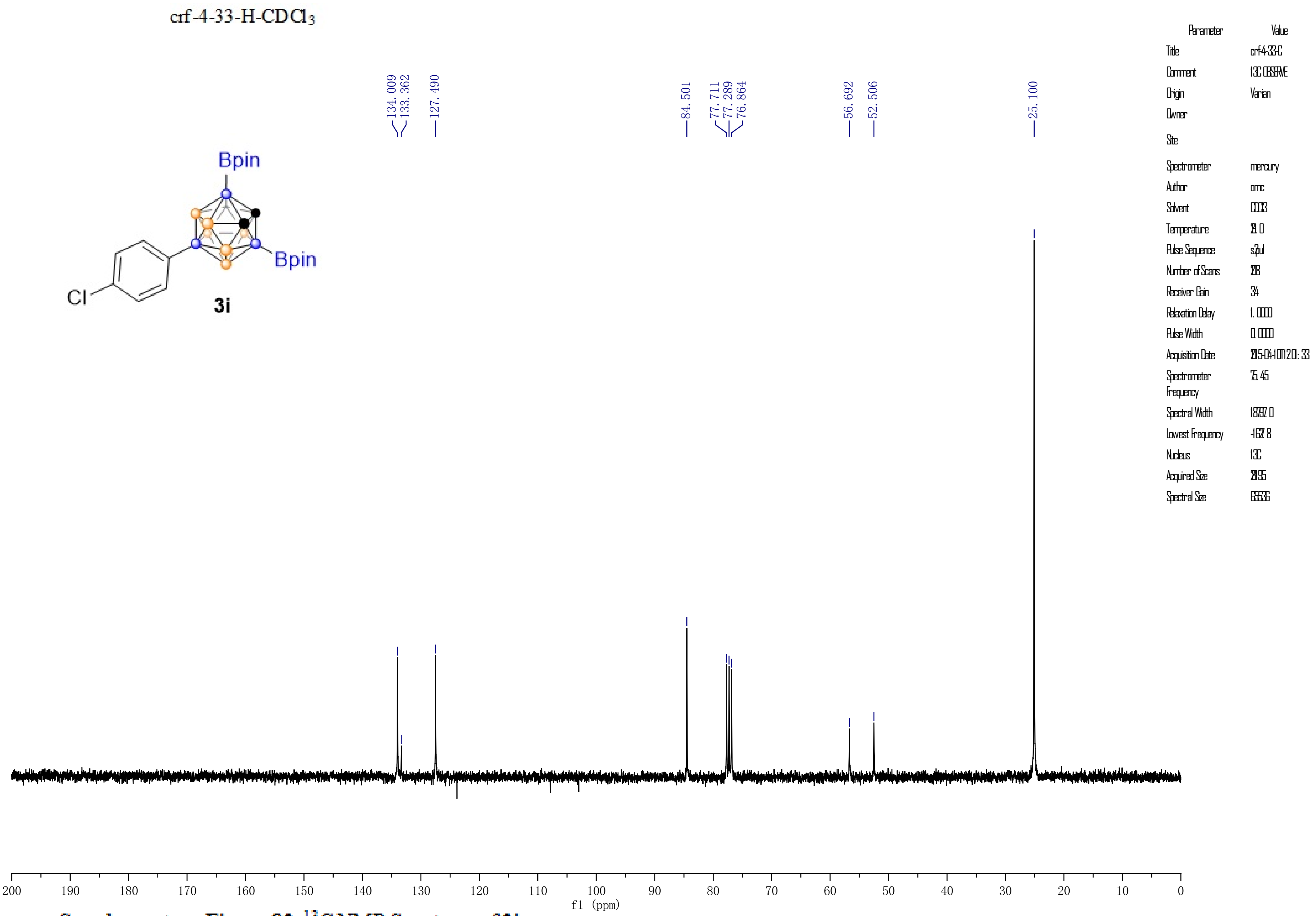

Supplementary Figure 83. ^{13}C NMR Spectrum of **3i**.

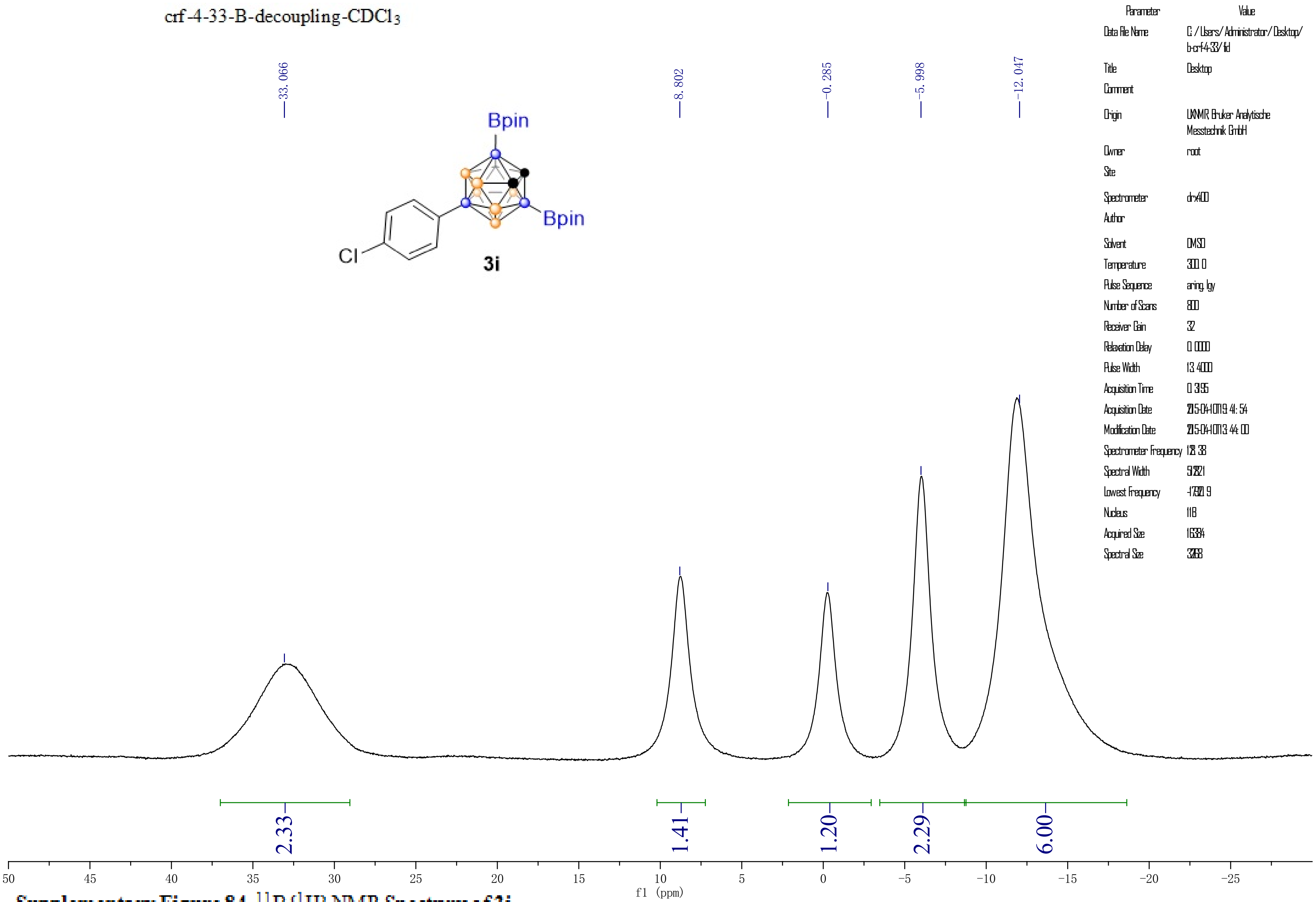

Supplementary Figure 84. $^{11}B\{^{1}H\}$ NMR Spectrum of **3i**.

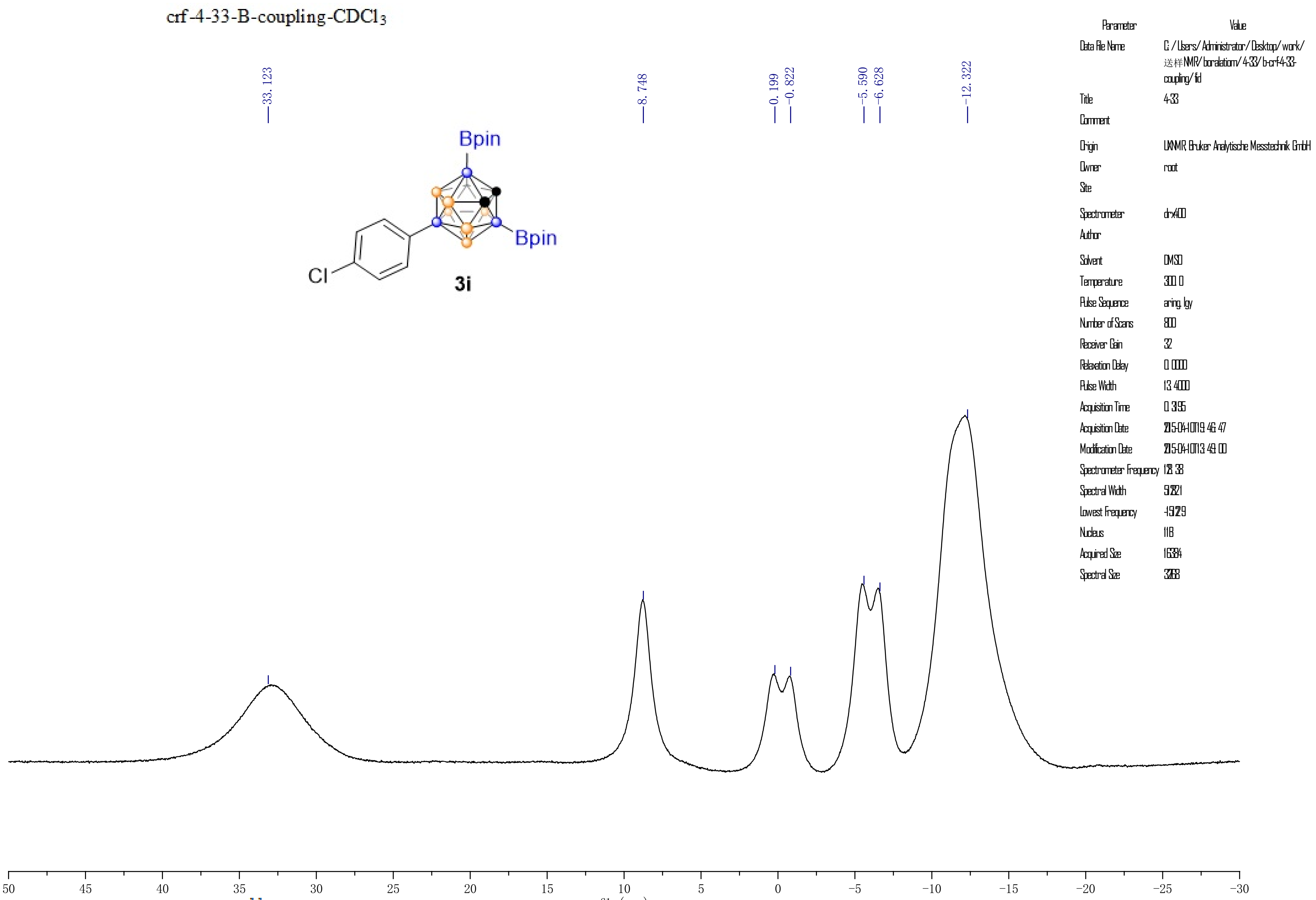

Supplementary Figure 85. ^{11}B NMR Spectrum of **3i**.

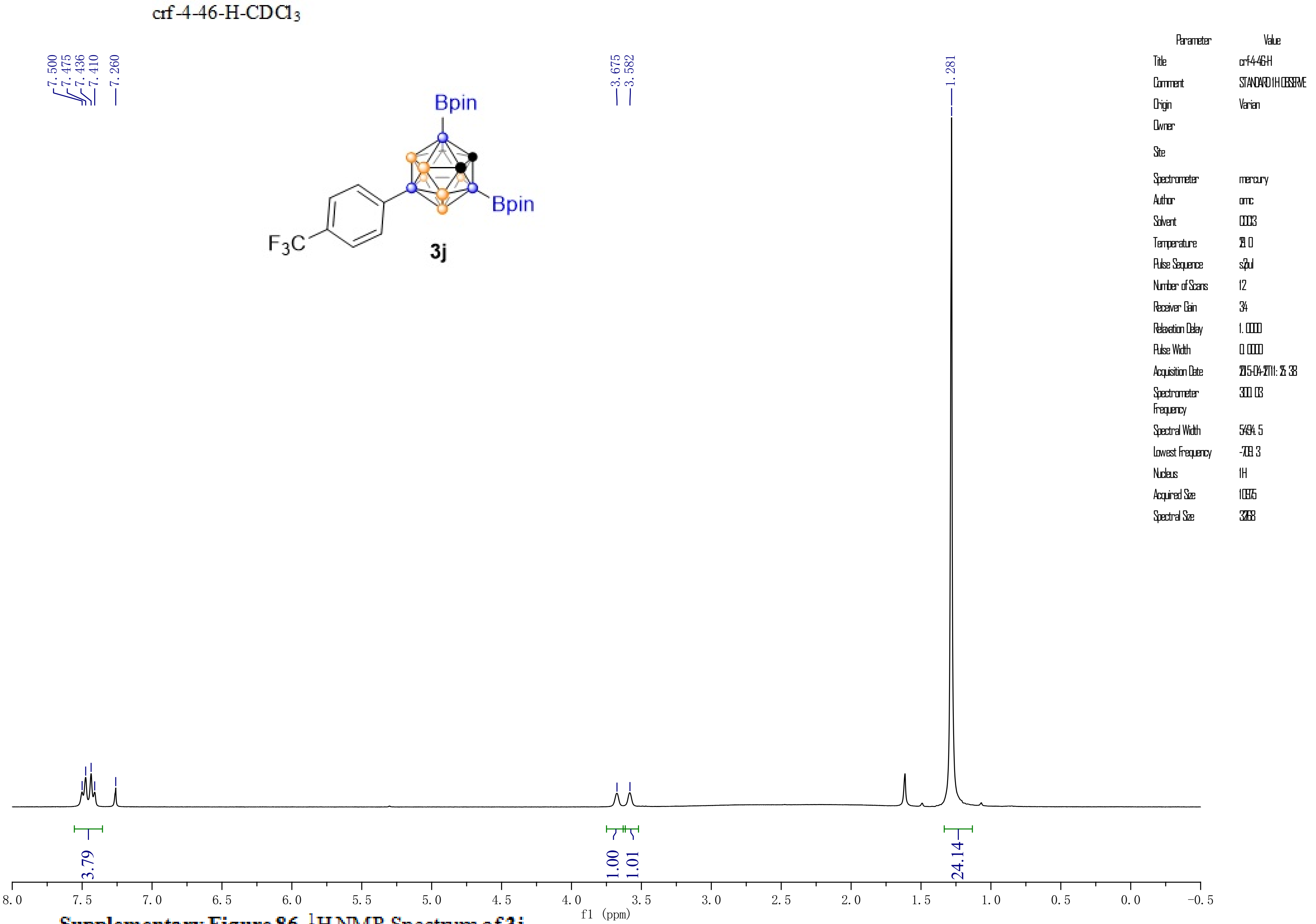

Supplementary Figure 86. ^{1}H NMR Spectrum of **3j**.

crf-4-46-C-CDCl$_3$

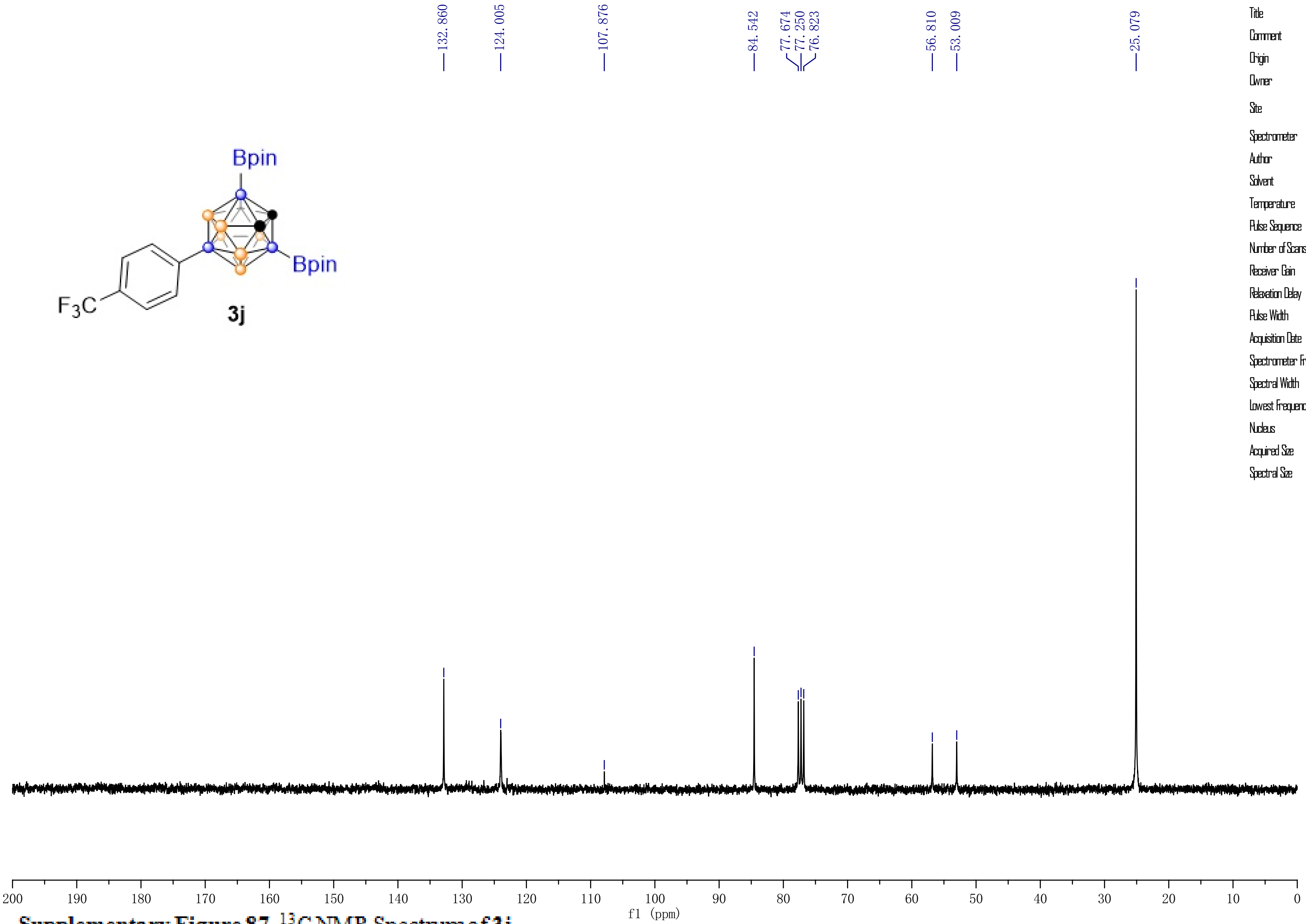

3j

Parameter	Value
Title	crf-4-46-C
Comment	13C OBSERVE
Origin	Varian
Owner	
Site	
Spectrometer	mercury
Author	omc
Solvent	CDCl3
Temperature	21.0
Pulse Sequence	s2pul
Number of Scans	4083
Receiver Gain	34
Relaxation Delay	1.0000
Pulse Width	0.0000
Acquisition Date	2015-04-27T12:22:34
Spectrometer Frequency	75.45
Spectral Width	18797.0
Lowest Frequency	-1627.8
Nucleus	13C
Acquired Size	28195
Spectral Size	65536

Supplementary Figure 87. ^{13}C NMR Spectrum of **3j**.

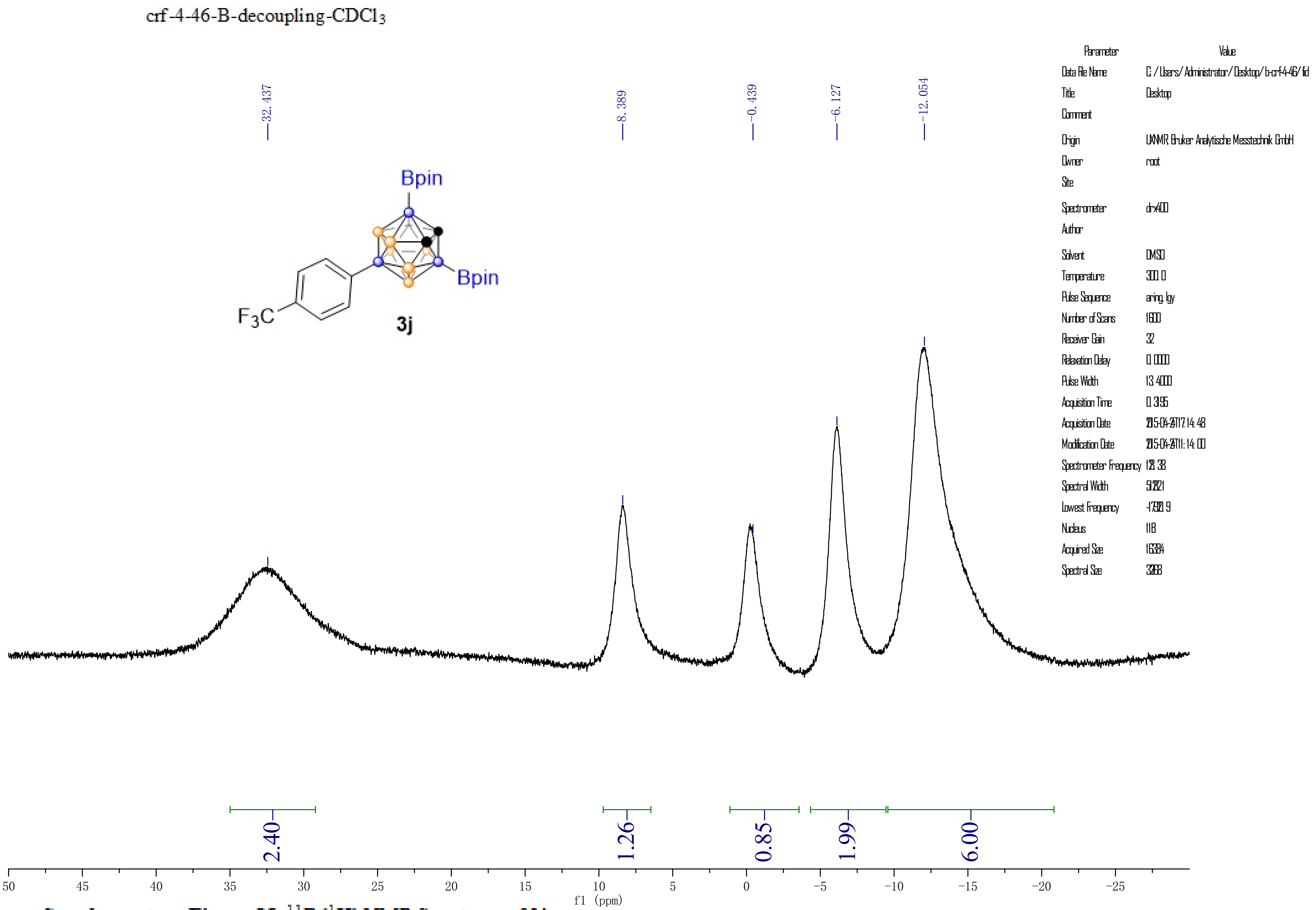

Supplementary Figure 88. $^{11}B\{^{1}H\}$ NMR Spectrum of **3j**.

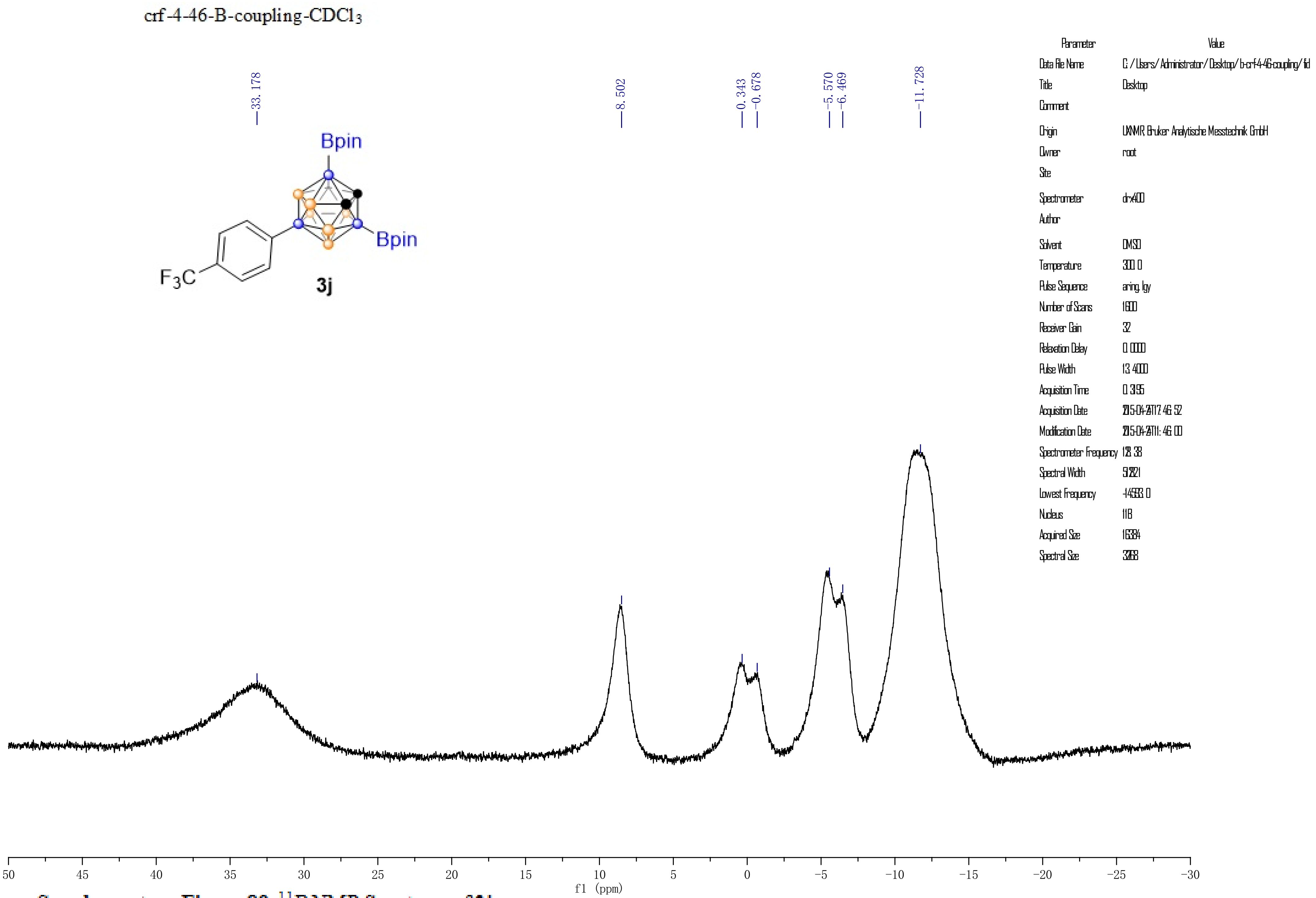

Supplementary Figure 89. ^{11}B NMR Spectrum of **3j**.

crf-4-46-F-$CDCl_3$

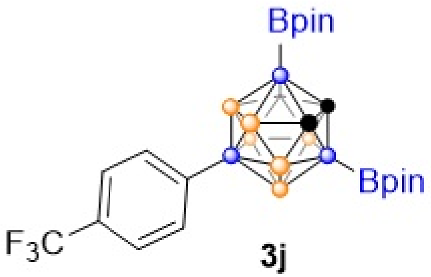

3j

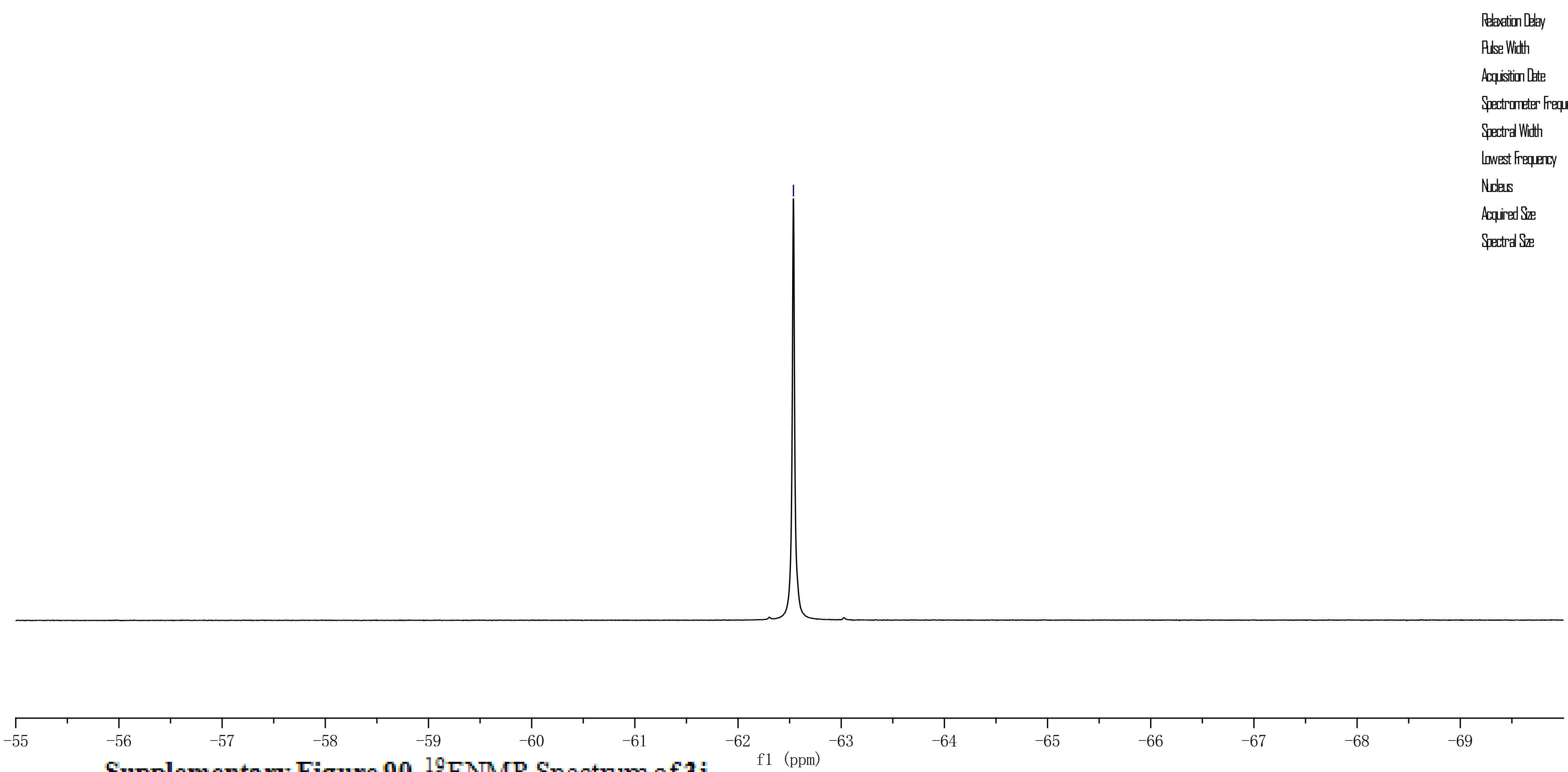

Parameter	Value
Title	2013153-crf-4-46_FLUORINE_01
Comment	2013153-crf-4-46
Origin	Varian
Owner	
Site	
Spectrometer	vnmrs
Author	
Solvent	cdcl3
Temperature	25.0
Pulse Sequence	s2pul
Number of Scans	16
Receiver Gain	56
Relaxation Delay	1.0000
Pulse Width	0.0000
Acquisition Date	2016-03-04T21:08:19
Spectrometer Frequency	376.06
Spectral Width	89285.7
Lowest Frequency	-76610.4
Nucleus	19F
Acquired Size	65536
Spectral Size	131072

Supplementary Figure 90. ^{19}F NMR Spectrum of **3j**.

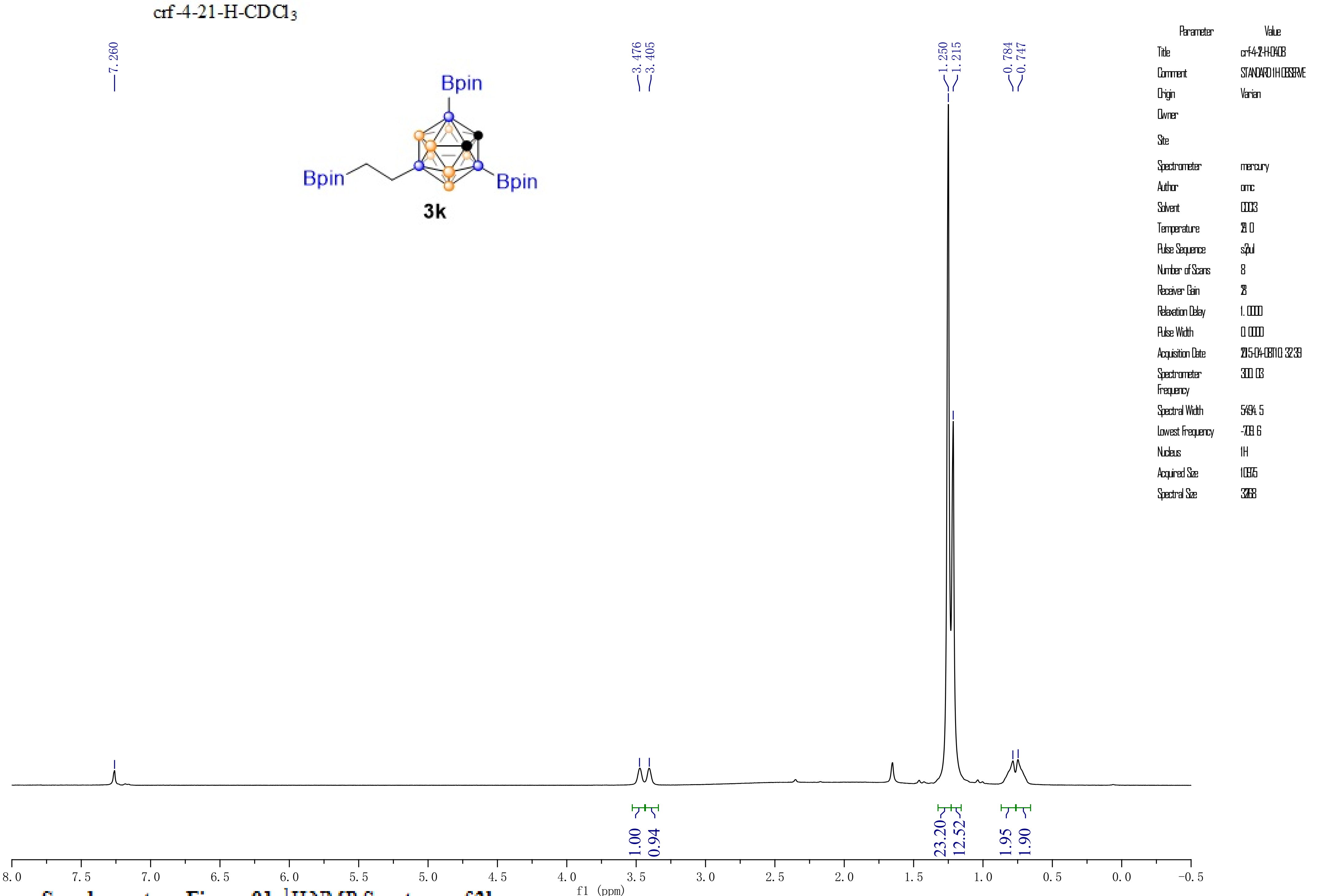

Supplementary Figure 91. ^{1}H NMR Spectrum of **3k**.

crf-4-21-H-CDCl3

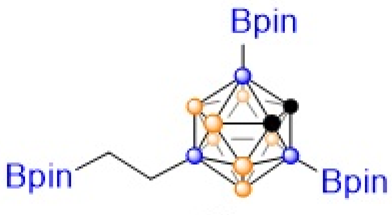

3k

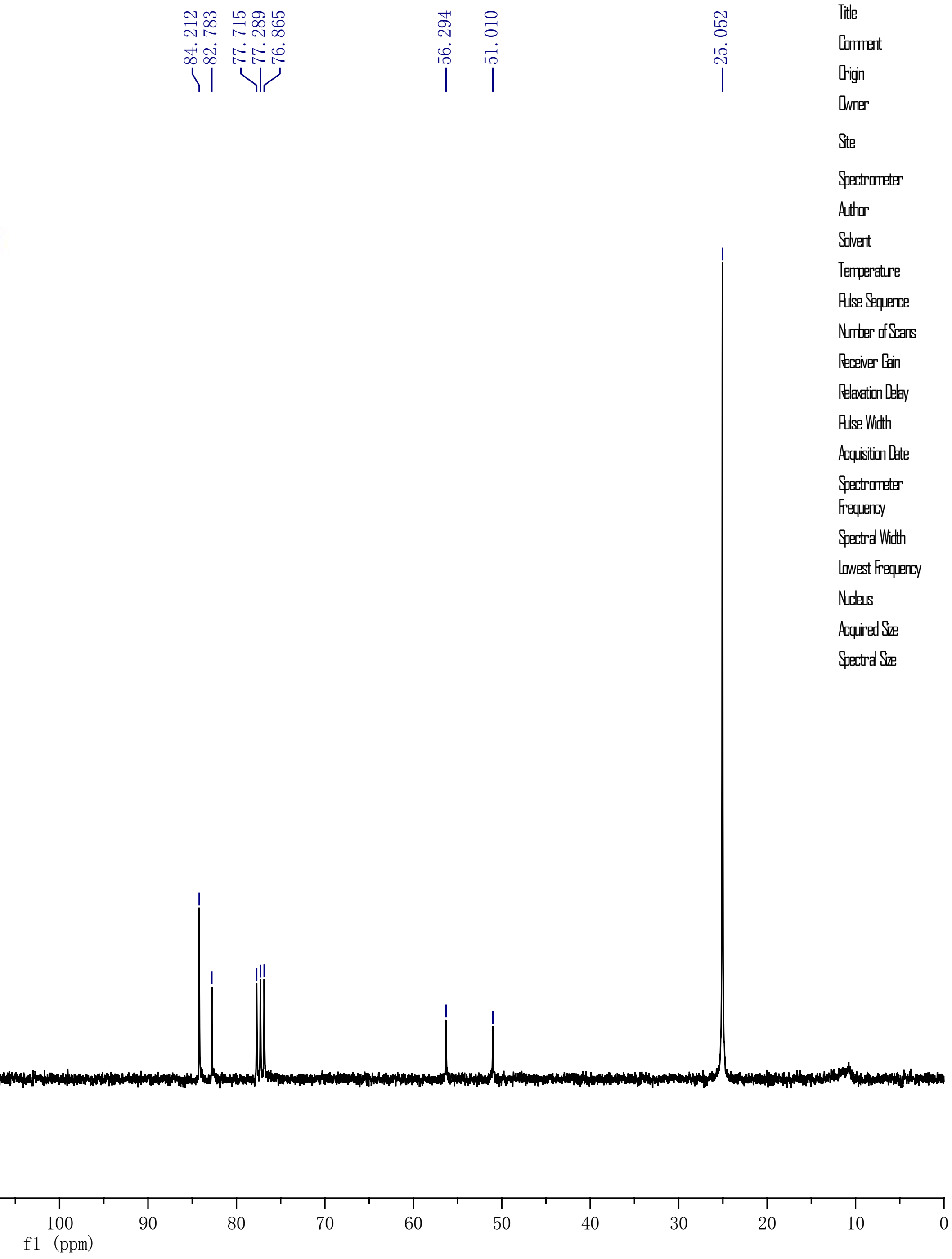

Parameter	Value
Title	crf-4-21-C-0408
Comment	13C OBSERVE
Origin	Varian
Owner	
Site	
Spectrometer	mercury
Author	omc
Solvent	CDCl3
Temperature	[illegible]
Pulse Sequence	s2pul
Number of Scans	128
Receiver Gain	34
Relaxation Delay	1.0000
Pulse Width	0.0000
Acquisition Date	2015-04-08T11:[illegible]
Spectrometer Frequency	75.45
Spectral Width	18797.0
Lowest Frequency	-1627.8
Nucleus	13C
Acquired Size	[illegible]
Spectral Size	65536

Supplementary Figure 92. ^{13}C NMR Spectrum of **3k**.

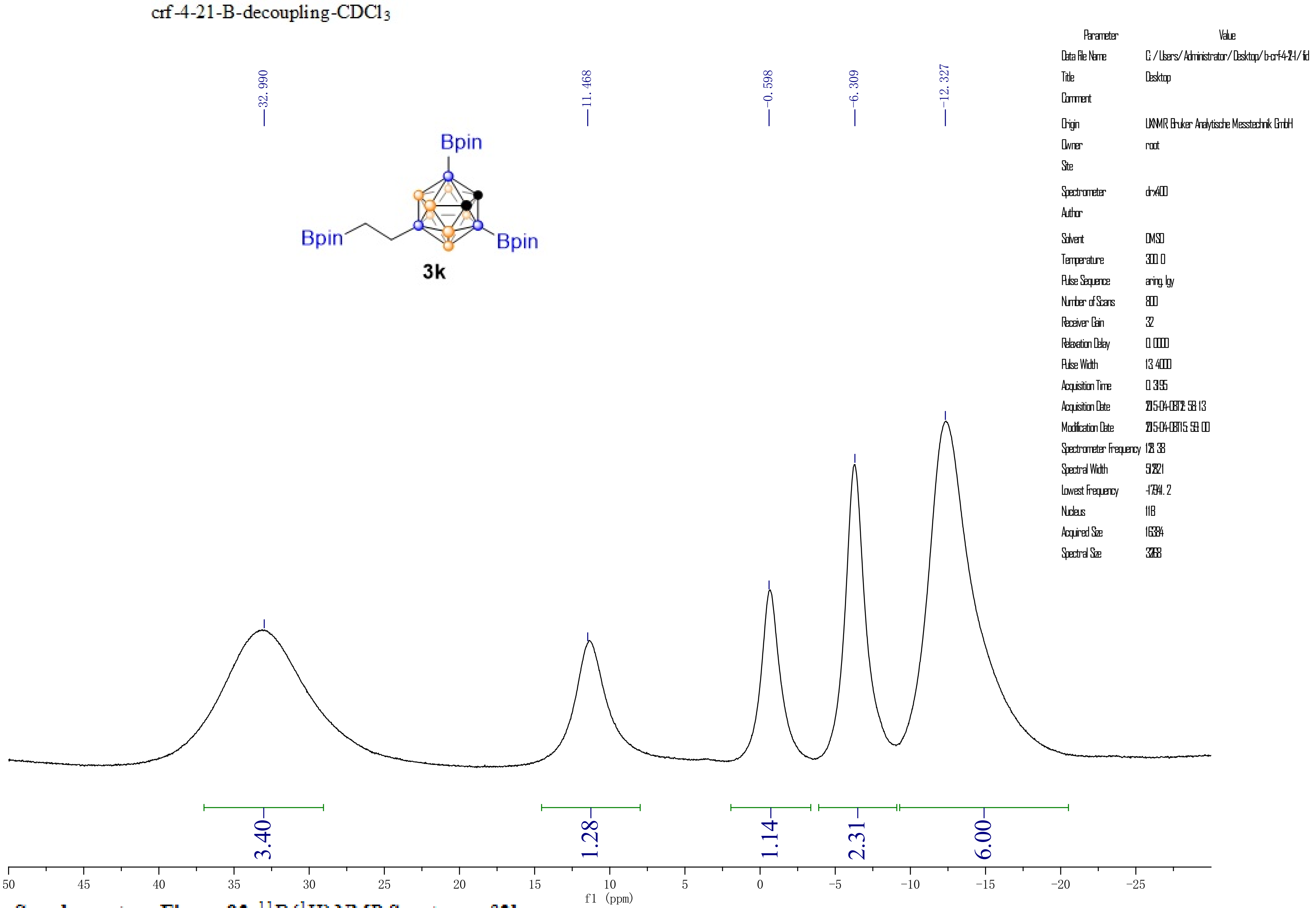

Supplementary Figure 93. $^{11}B\{^{1}H\}$ NMR Spectrum of **3k**.

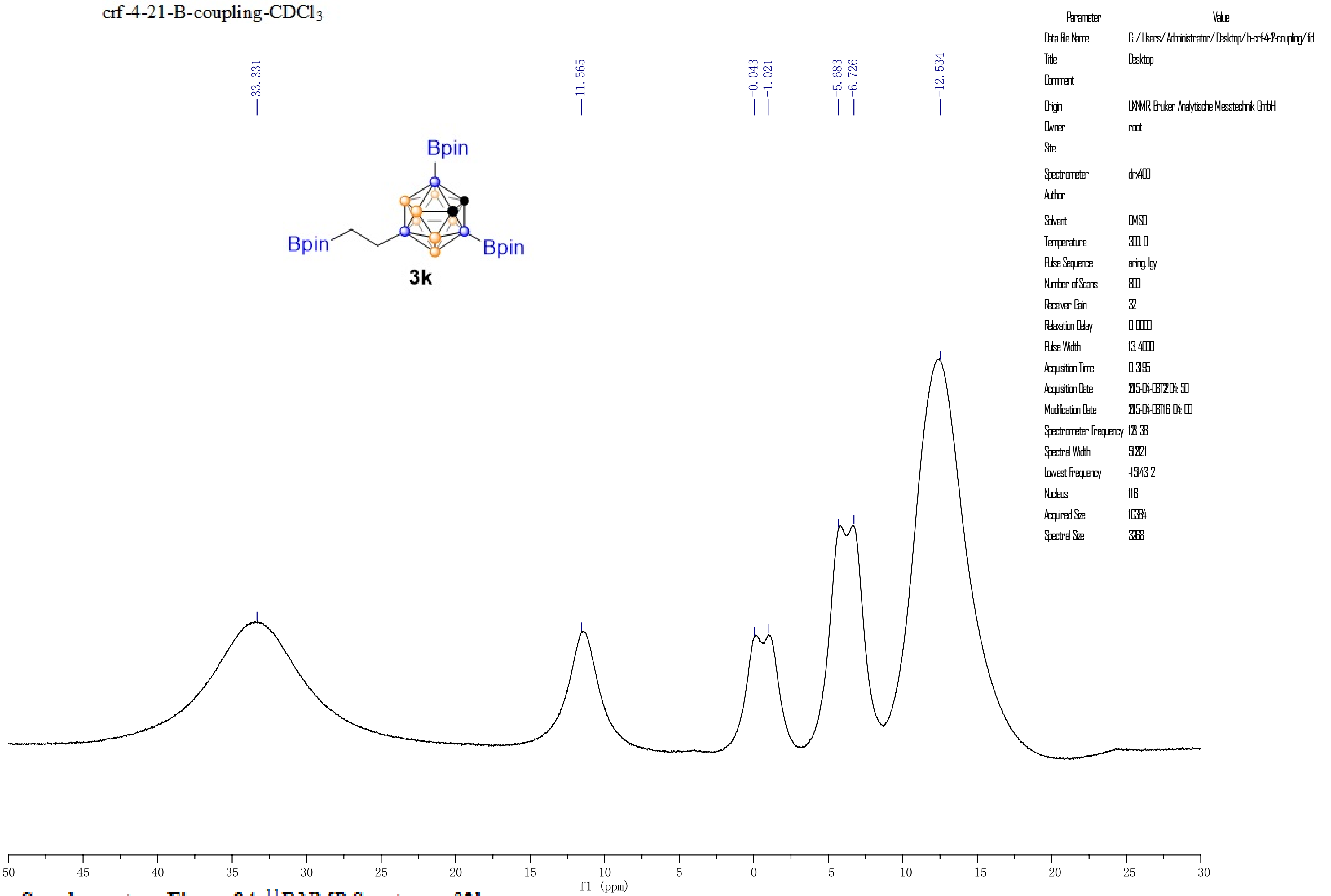

Supplementary Figure 94. ^{11}B NMR Spectrum of **3k**.

crf-3-76-H-$CDCl_3$

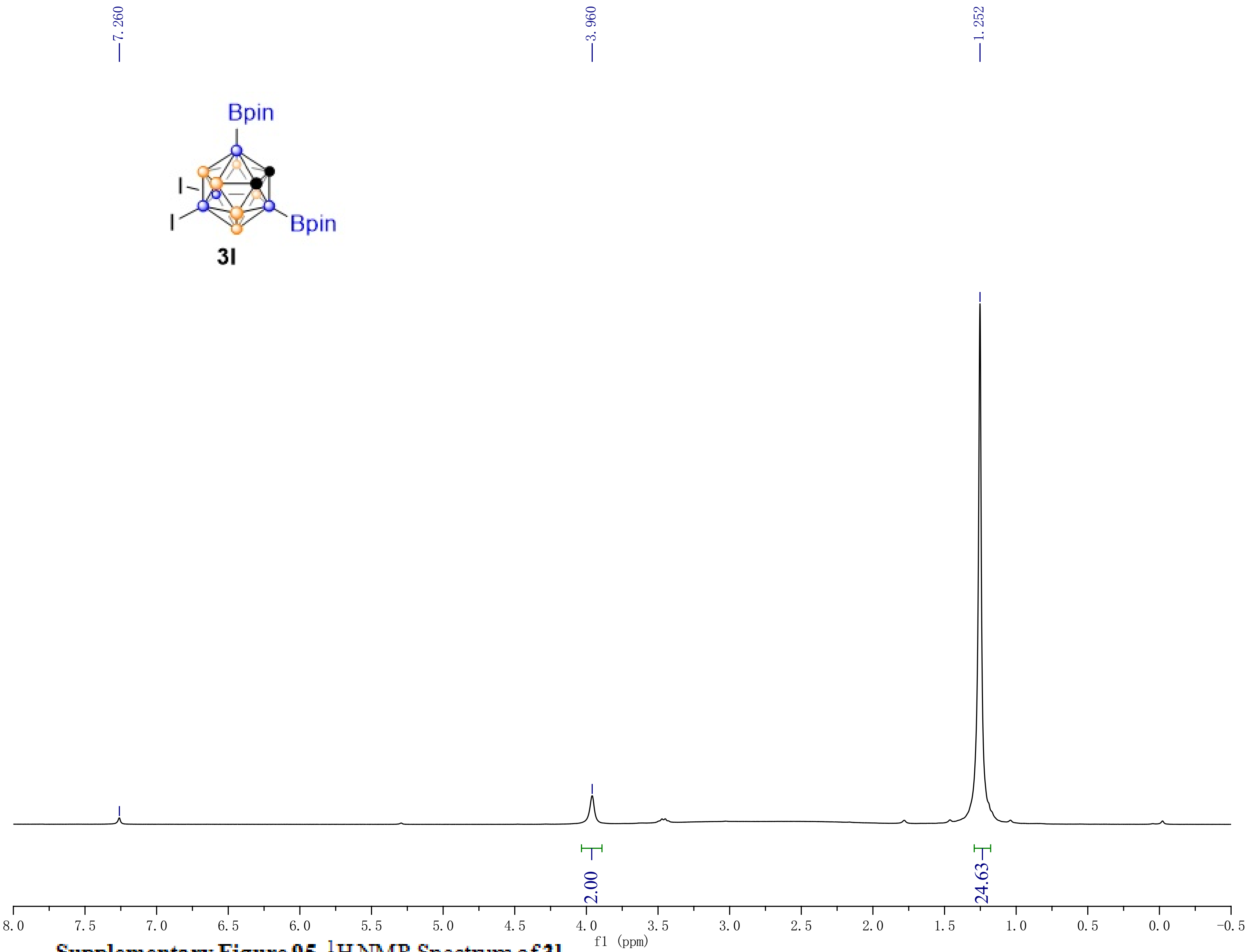

Parameter	Value
Title	crf-3-76-H
Comment	STANDARD 1H OBSERVE
Origin	Varian
Owner	
Site	
Spectrometer	mercury
Author	omc
Solvent	CDCl3
Temperature	[illegible]
Pulse Sequence	s2pul
Number of Scans	8
Receiver Gain	[illegible]
Relaxation Delay	1.0000
Pulse Width	0.0000
Acquisition Date	[illegible]
Spectrometer Frequency	[illegible]
Spectral Width	5494.5
Lowest Frequency	[illegible]
Nucleus	1H
Acquired Size	[illegible]
Spectral Size	[illegible]

Supplementary Figure 95. ^{1}H NMR Spectrum of **3l**.

crf-3-76-C-$CDCl_3$

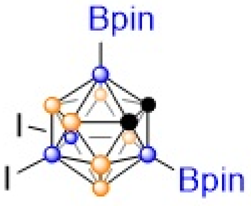

3l

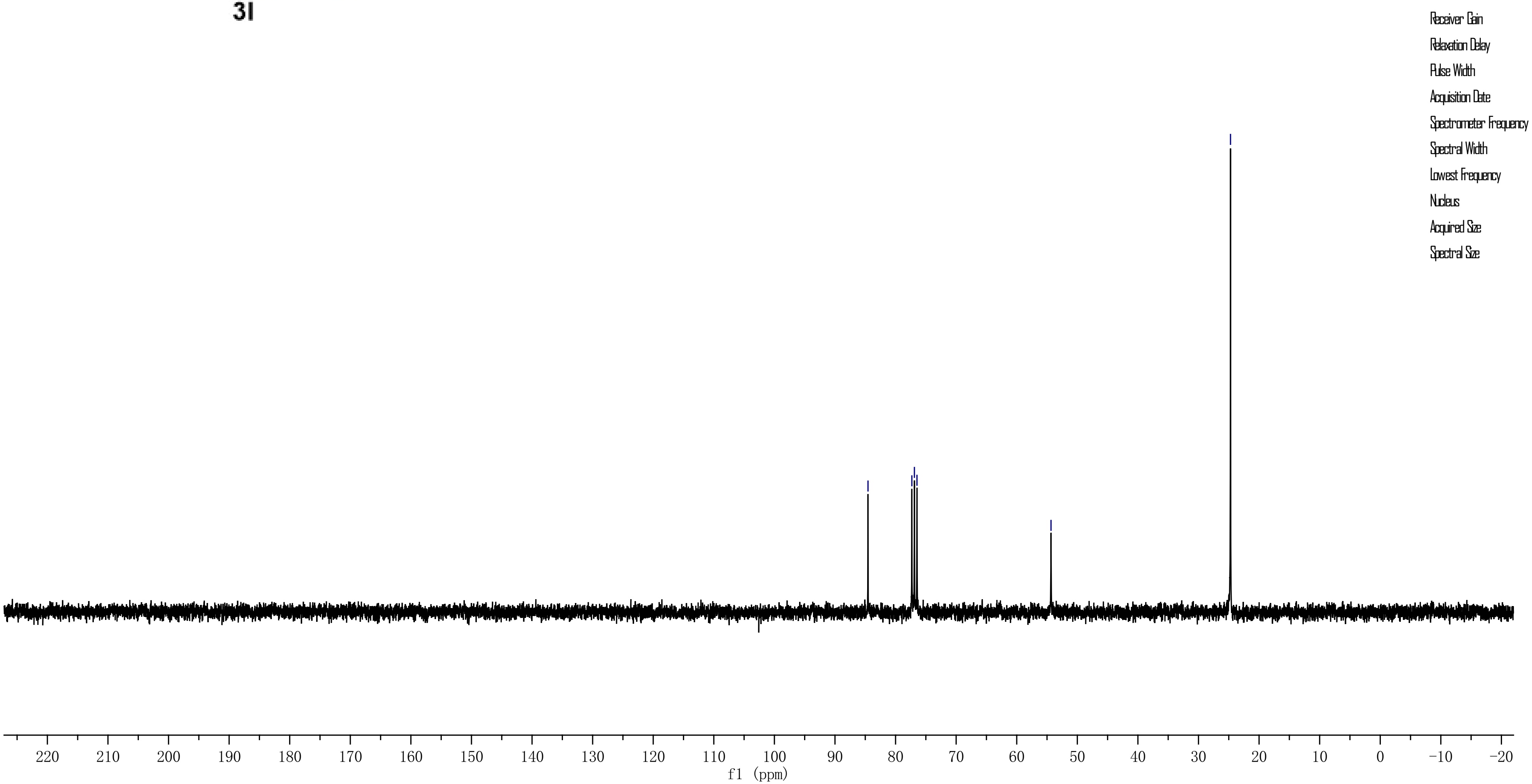

Parameter	Value
Title	crf-3-76-C
Comment	13C OBSERVE
Origin	Varian
Owner	
Site	
Spectrometer	mercury
Author	omc
Solvent	CDCl3
Temperature	21.0
Pulse Sequence	s2pul
Number of Scans	40
Receiver Gain	34
Relaxation Delay	1.0000
Pulse Width	0.0000
Acquisition Date	[illegible]
Spectrometer Frequency	75.45
Spectral Width	18797.0
Lowest Frequency	[illegible]
Nucleus	13C
Acquired Size	[illegible]
Spectral Size	65536

Supplementary Figure 96. ^{13}C NMR Spectrum of **3l**.

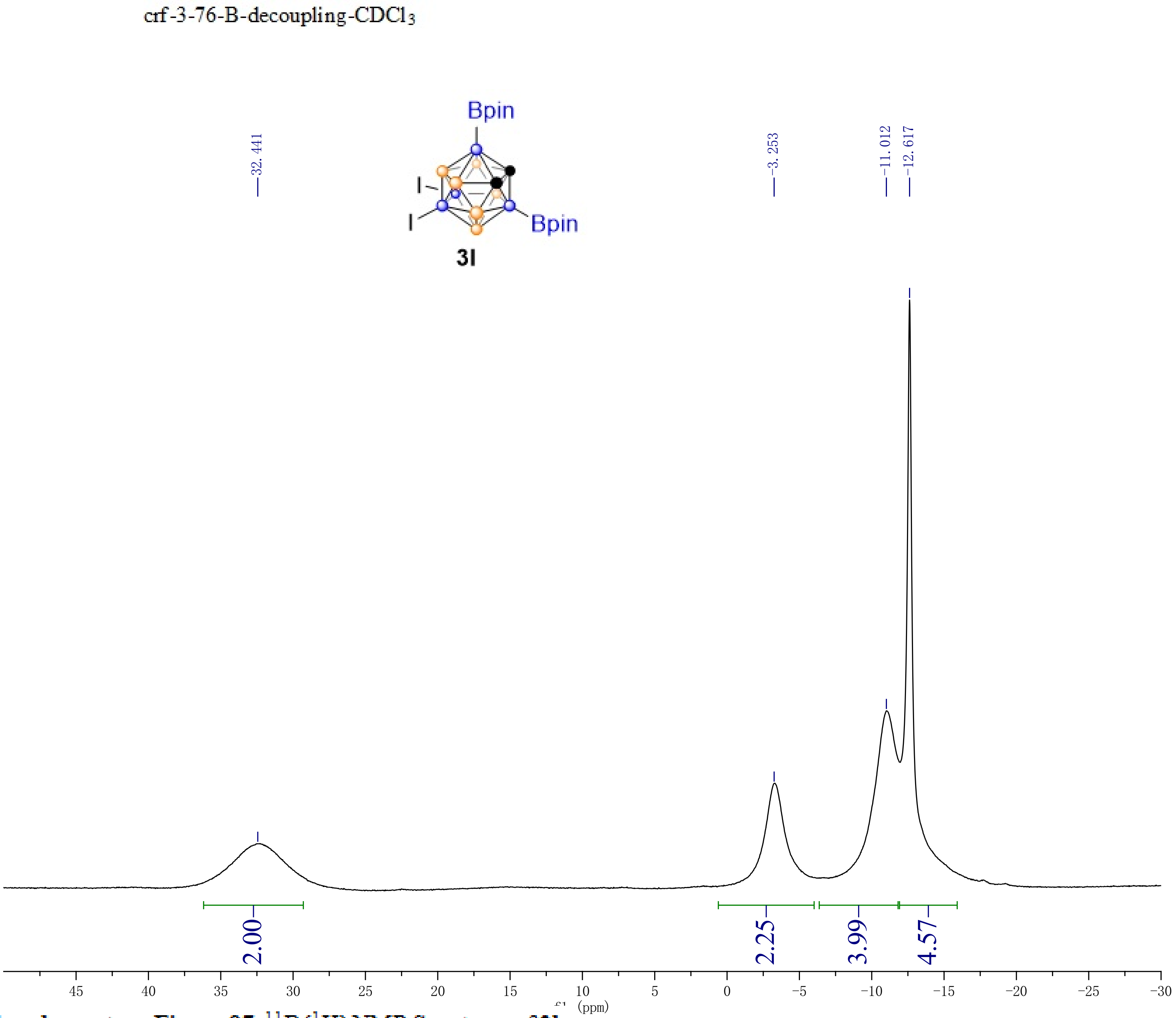

Parameter	Value
Data File Name	C:/Users/Administrator/Desktop/b-crf-3-76/fid
Title	Desktop
Comment	
Origin	UXNMR, Bruker Analytische Messtechnik GmbH
Owner	root
Site	
Spectrometer	drx400
Author	
Solvent	DMSO
Temperature	300.0
Pulse Sequence	aring.lgy
Number of Scans	435
Receiver Gain	32
Relaxation Delay	0.0000
Pulse Width	13.4000
Acquisition Time	0.3195
Acquisition Date	2015-02-04T15:59:59
Modification Date	2015-02-04T09:00:00
Spectrometer Frequency	128.38
Spectral Width	51282.1
Lowest Frequency	-18585.1
Nucleus	11B
Acquired Size	16384
Spectral Size	32768

Supplementary Figure 97. $^{11}B\{^{1}H\}$ **NMR Spectrum of 3l.**

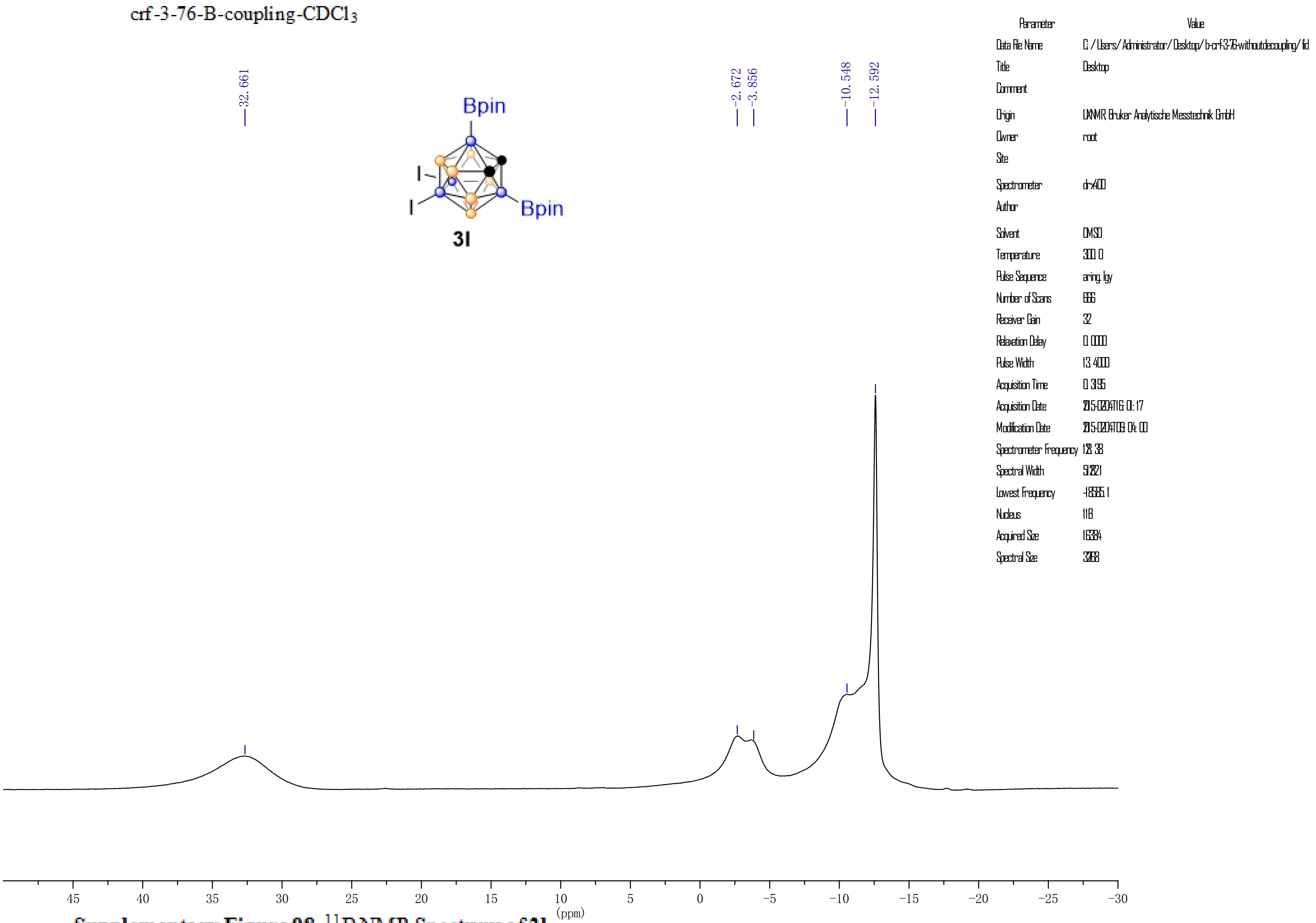

Supplementary Figure 98. ^{11}B NMR Spectrum of **3l**.

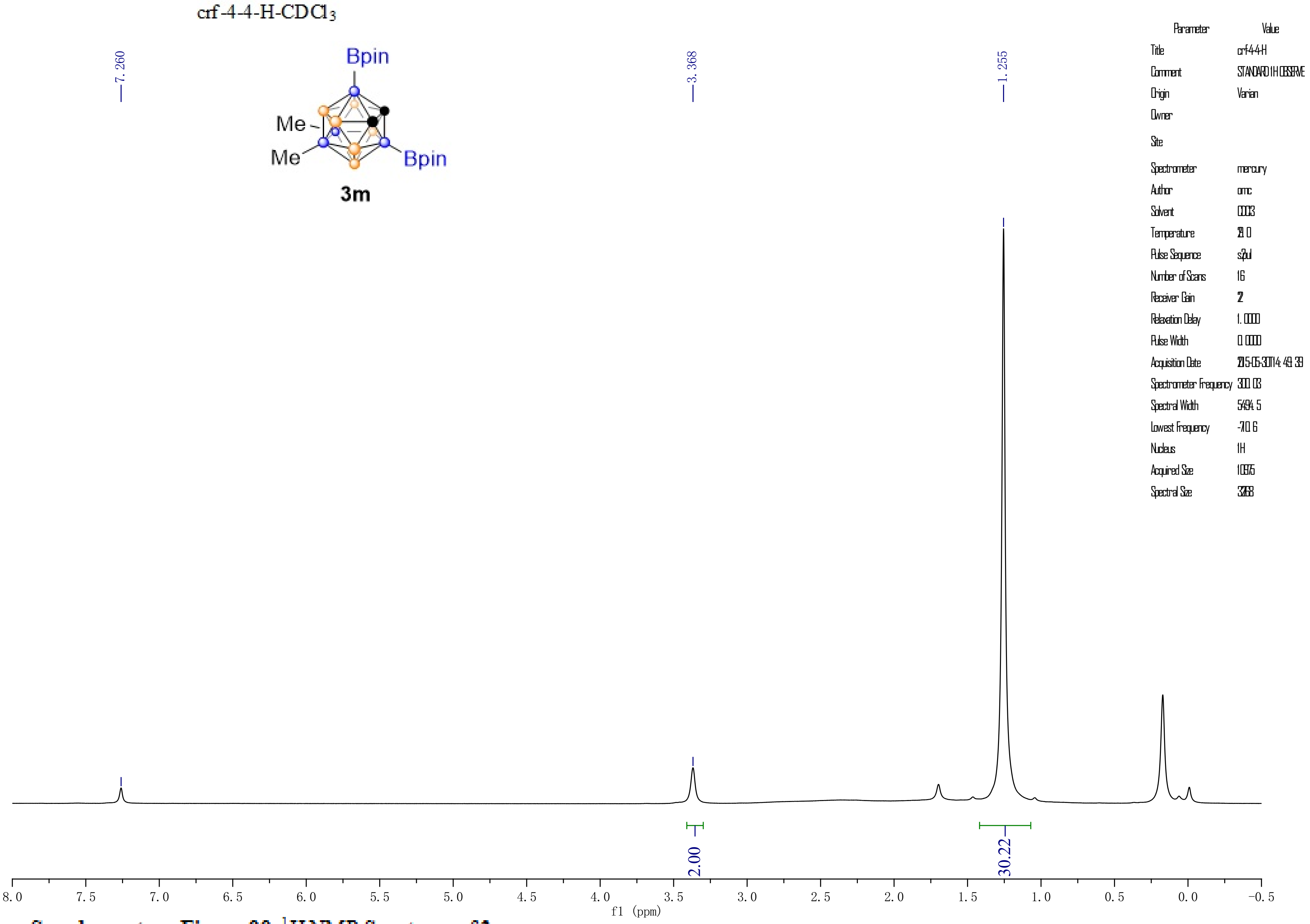

Supplementary Figure 99. ^{1}H NMR Spectrum of **3m**.

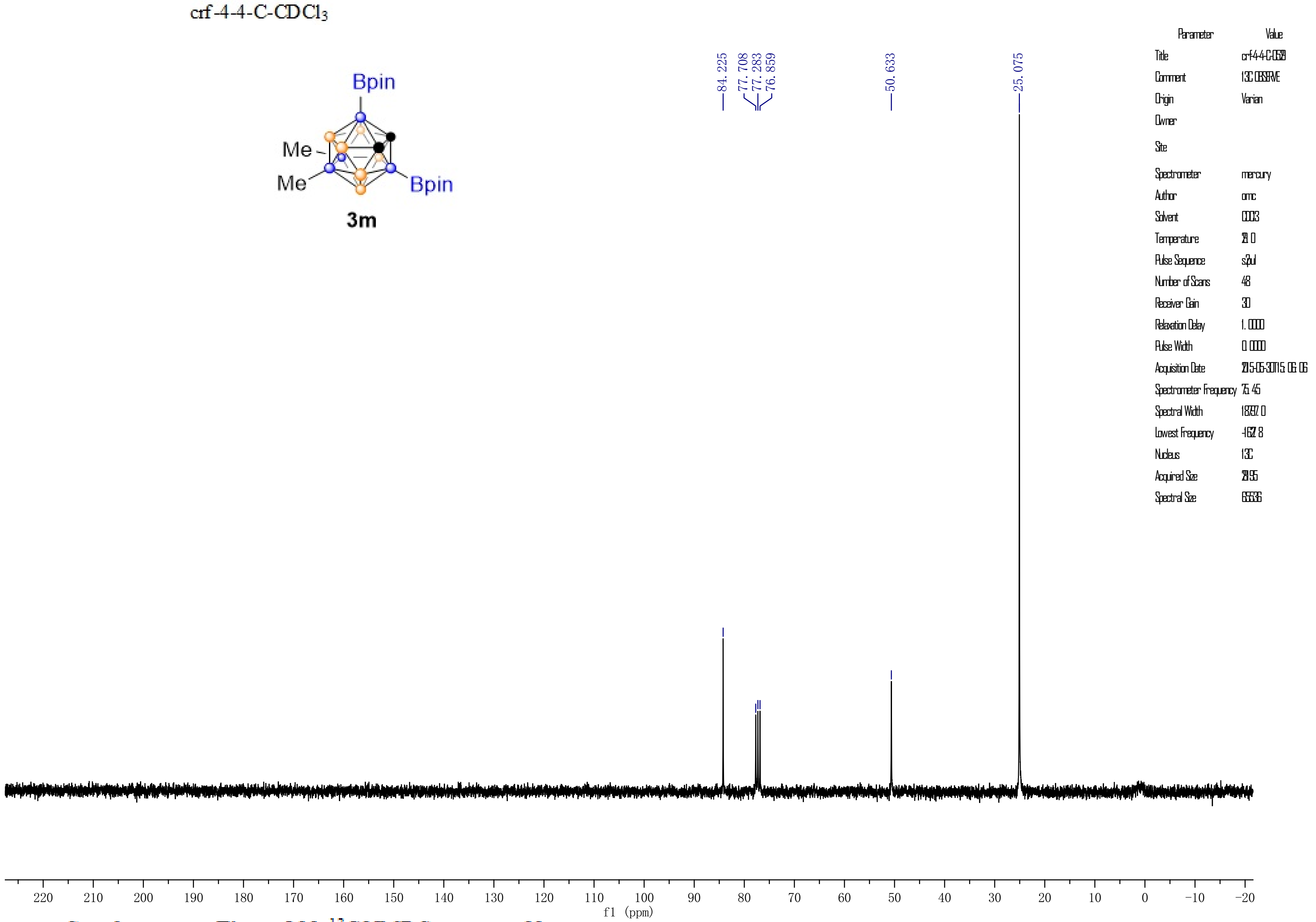

Supplementary Figure 100. ^{13}C NMR Spectrum of **3m**.

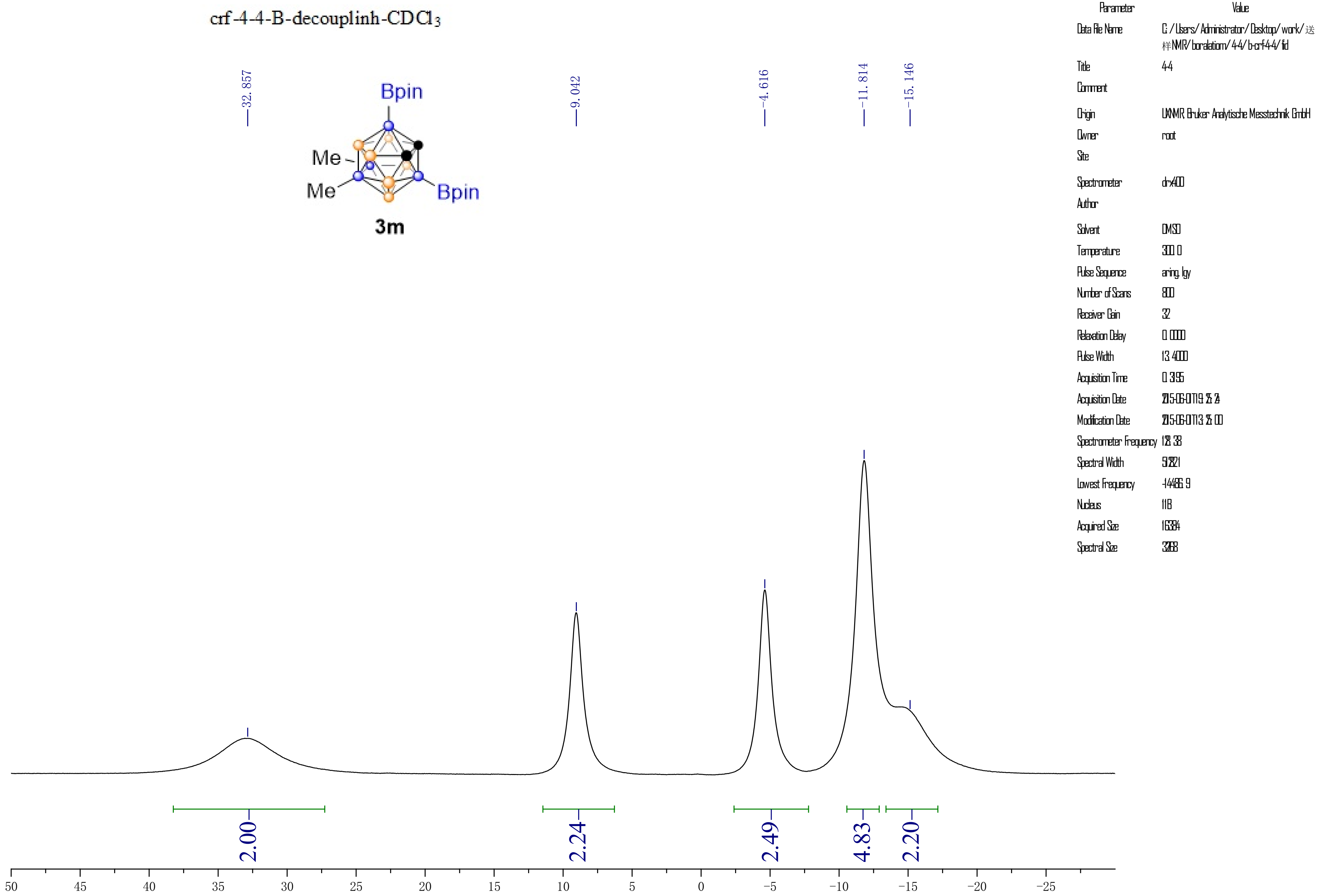

Supplementary Figure 101. $^{11}B\{^{1}H\}$ NMR Spectrum of **3m**.

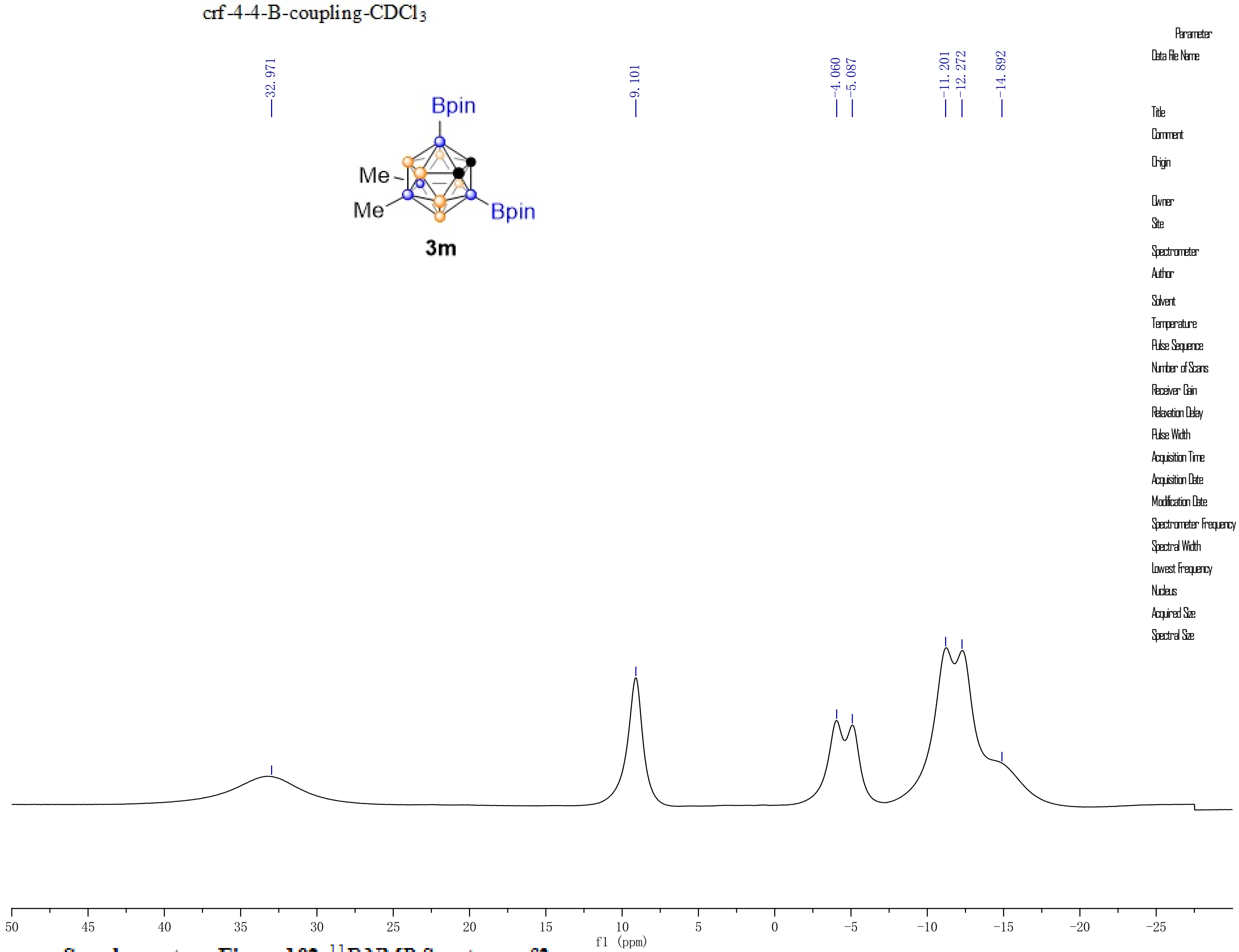

Parameter	Value
Data File Name	C:/Users/Administrator/Desktop/work/送样NMR/boralatiom/4-4/b-crf-4-4-coupling/fid
Title	4-4
Comment	
Origin	UXNMR, Bruker Analytische Messtechnik GmbH
Owner	root
Site	
Spectrometer	drx400
Author	
Solvent	DMSO
Temperature	300.0
Pulse Sequence	aring.lgy
Number of Scans	800
Receiver Gain	32
Relaxation Delay	0.0000
Pulse Width	13.4000
Acquisition Time	0.3195
Acquisition Date	2015-06-01T19:18:49
Modification Date	2015-06-01T13:18:00
Spectrometer Frequency	128.38
Spectral Width	51282.1
Lowest Frequency	-14486.9
Nucleus	11B
Acquired Size	16384
Spectral Size	32768

Supplementary Figure102. ^{11}B NMR Spectrum of **3m**.

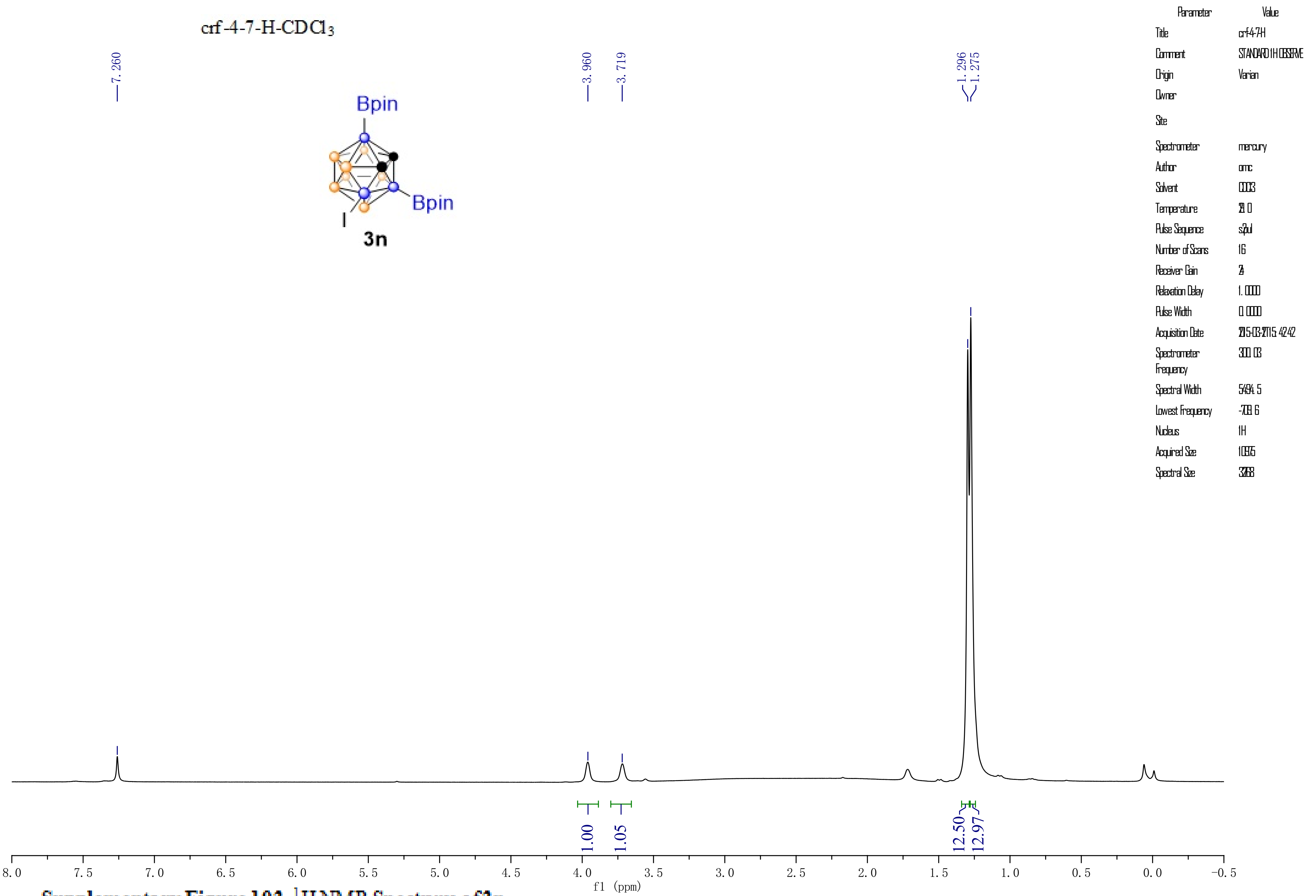

Supplementary Figure 103. ¹H NMR Spectrum of **3n**.

crf-4-7-C-CDCl3

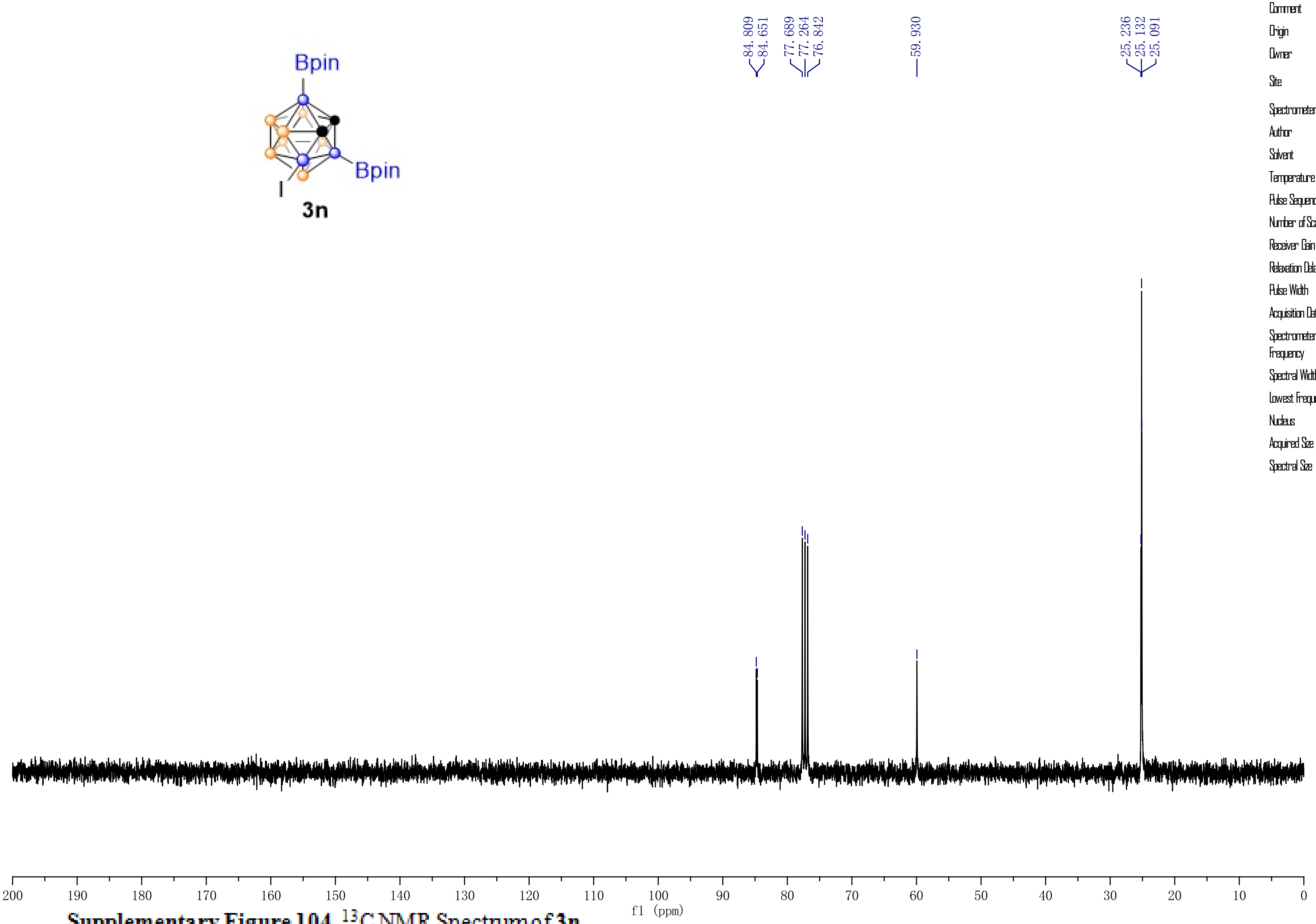

3n

Parameter	Value
Title	crf-4-7C-0409
Comment	13C OBSERVE
Origin	Varian
Owner	
Site	
Spectrometer	mercury
Author	omc
Solvent	CDCl3
Temperature	21.0
Pulse Sequence	s2pul
Number of Scans	112
Receiver Gain	34
Relaxation Delay	1.0000
Pulse Width	0.0000
Acquisition Date	2015-04-09T17:55:24
Spectrometer Frequency	75.45
Spectral Width	18797.0
Lowest Frequency	-1652.8
Nucleus	13C
Acquired Size	28955
Spectral Size	65536

Supplementary Figure 104. ^{13}C NMR Spectrum of **3n**.

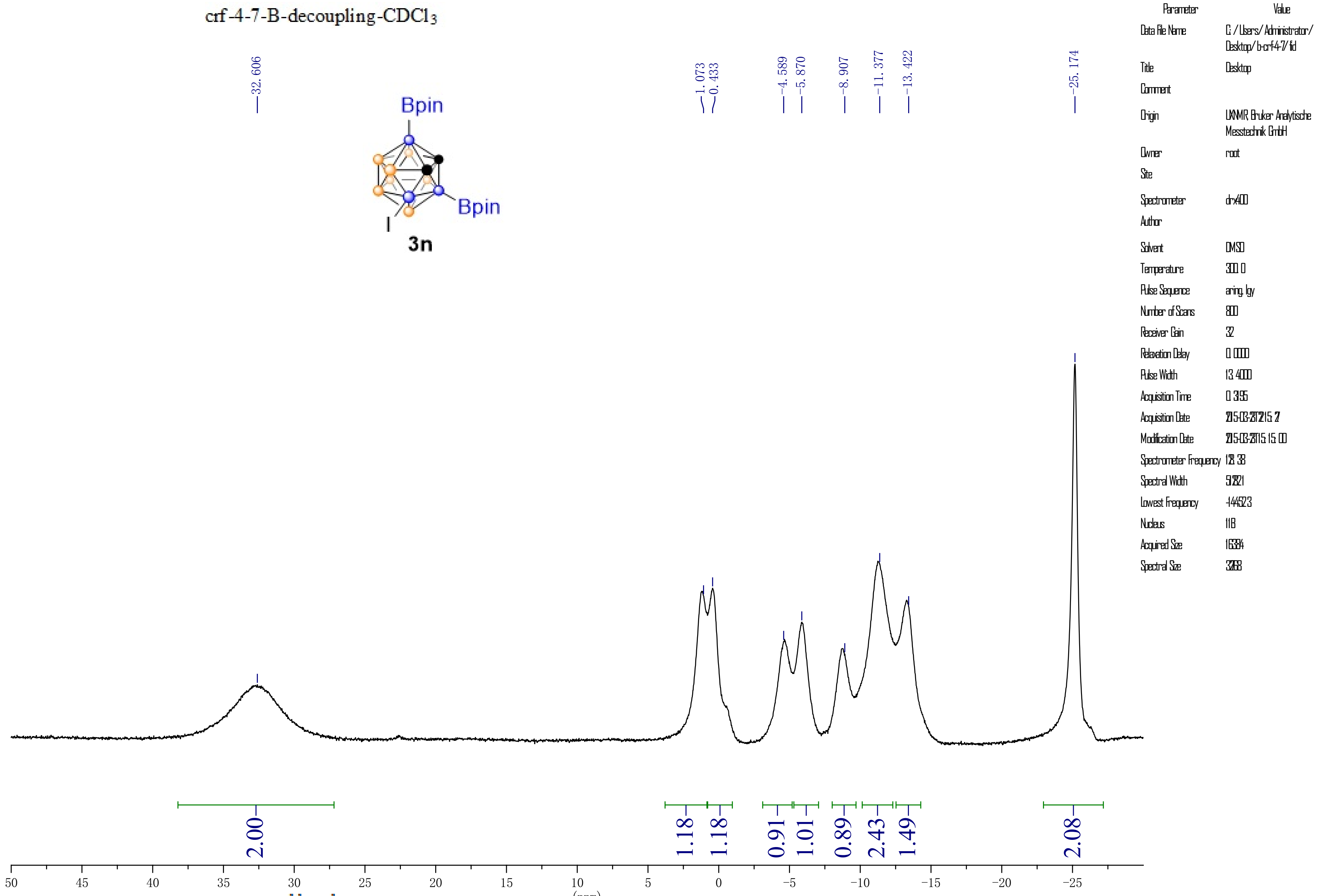

Supplementary Figure 105. $^{11}B\{^{1}H\}$ NMR Spectrum of **3n**.

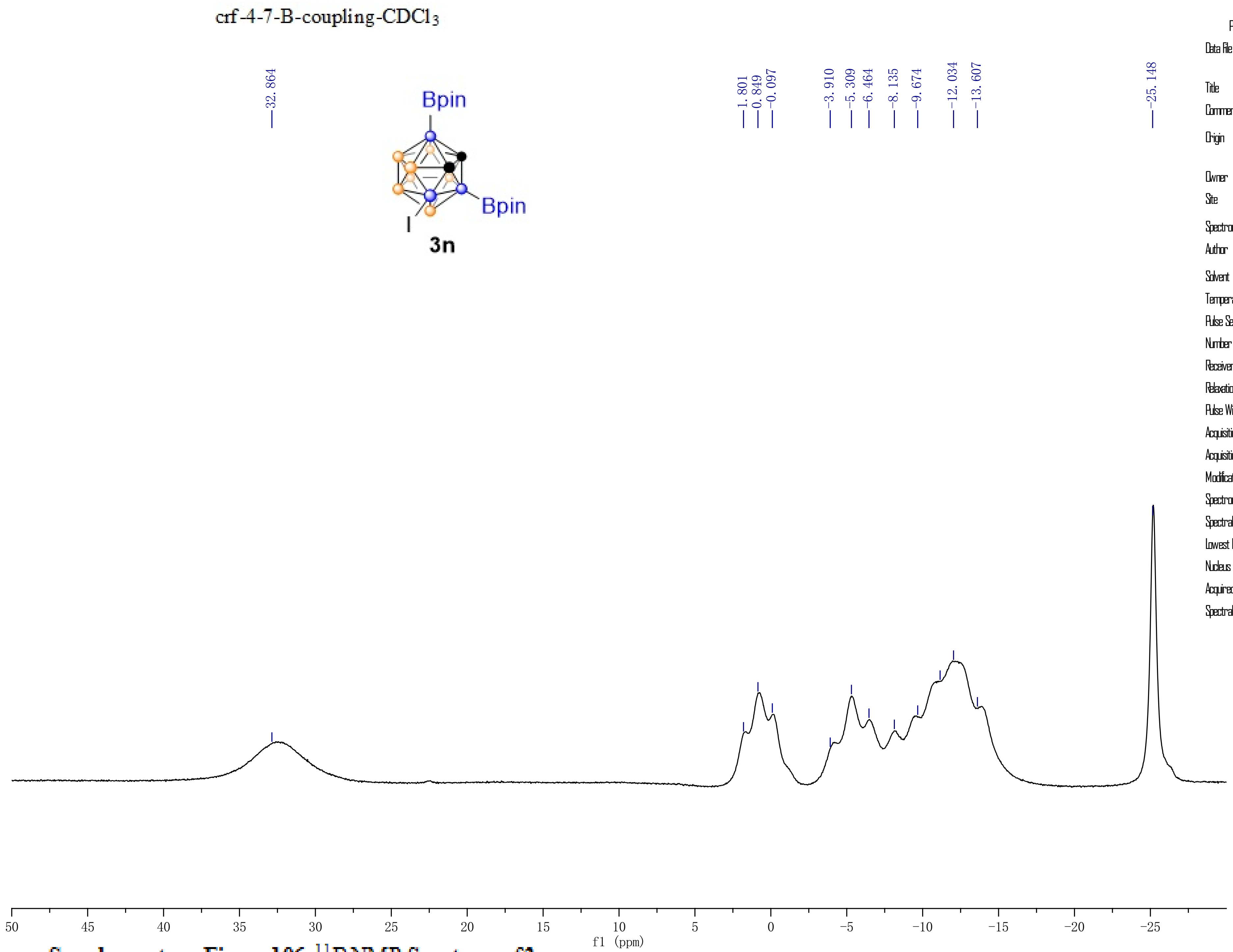

Parameter	Value
Data File Name	C:/Users/Administrator/Desktop/b-crf-4-7-coupling/fid
Title	Desktop
Comment	
Origin	UXNMR, Bruker Analytische Messtechnik GmbH
Owner	root
Site	
Spectrometer	drx400
Author	
Solvent	DMSO
Temperature	300.0
Pulse Sequence	aring.lgy
Number of Scans	1600
Receiver Gain	32
Relaxation Delay	0.0000
Pulse Width	13.4000
Acquisition Time	0.3195
Acquisition Date	2015-03-28T[illegible]
Modification Date	2015-03-28T15:[illegible]
Spectrometer Frequency	128.38
Spectral Width	51282.1
Lowest Frequency	-14452.3
Nucleus	11B
Acquired Size	16384
Spectral Size	32768

Supplementary Figure 106. ^{11}B NMR Spectrum of **3n**.

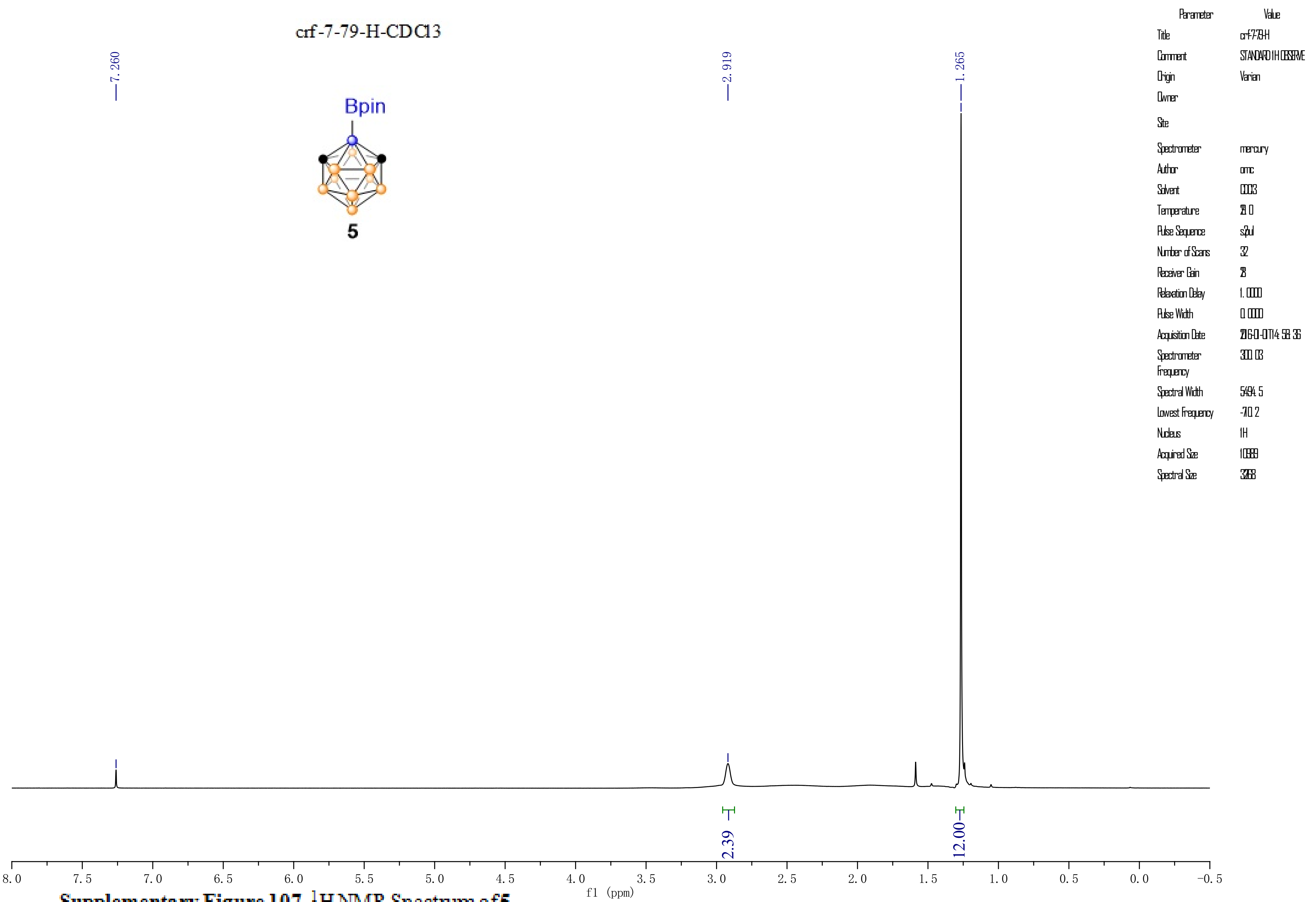

Supplementary Figure 107. ^{1}H NMR Spectrum of **5**.

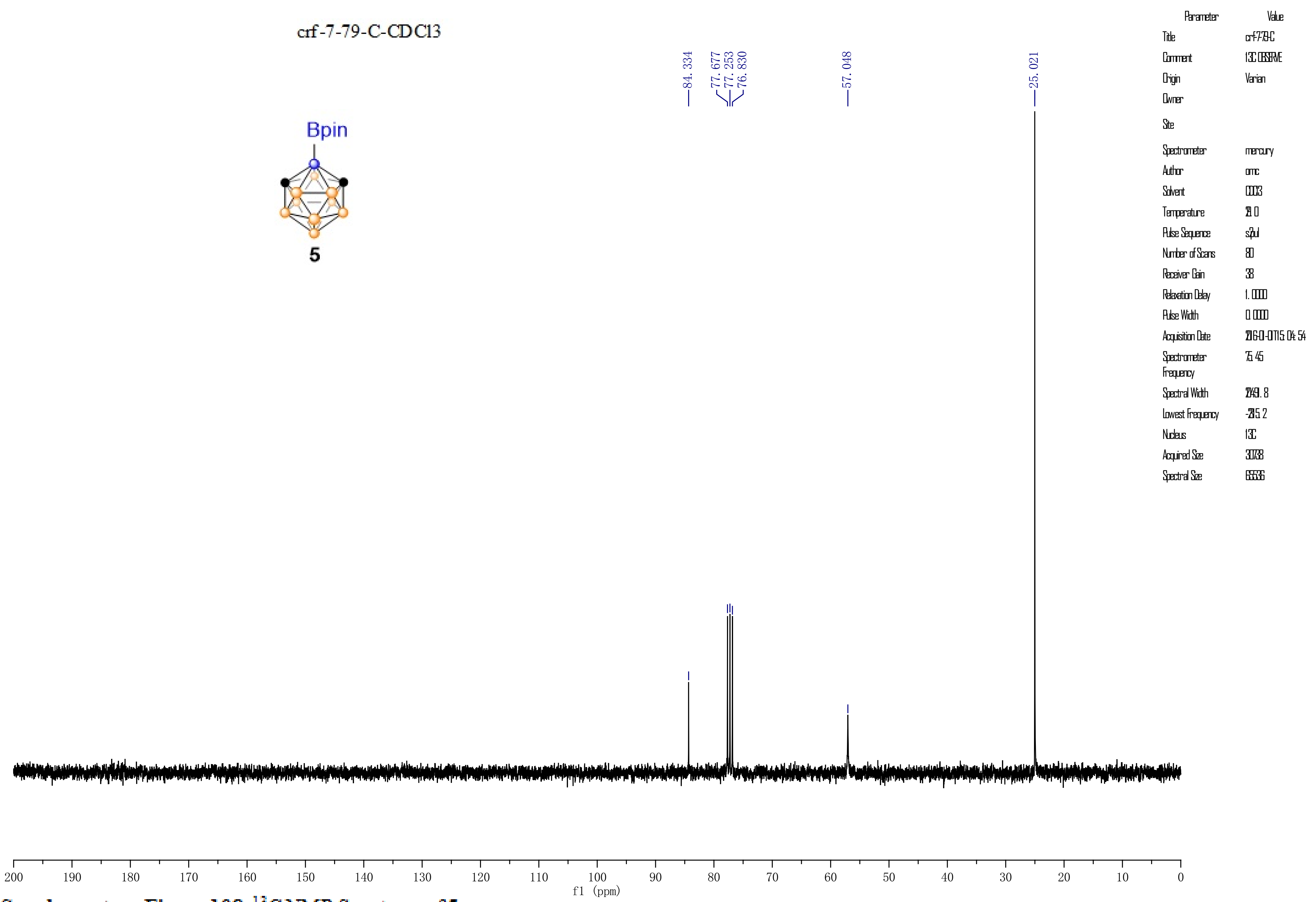

Supplementary Figure 108. ^{13}C NMR Spectrum of **5**.

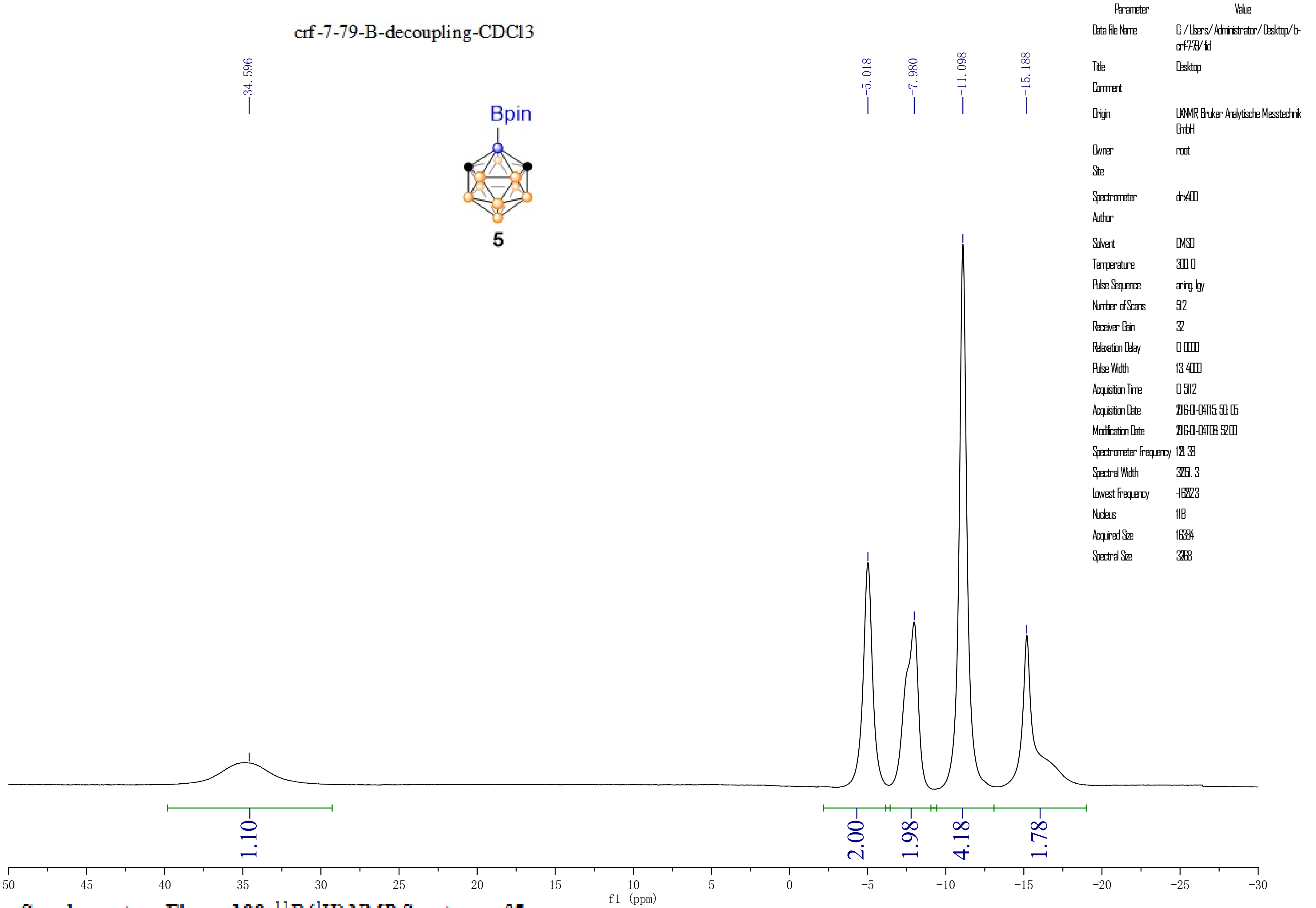

Supplementary Figure 109. $^{11}B\{^{1}H\}$ NMR Spectrum of **5**.

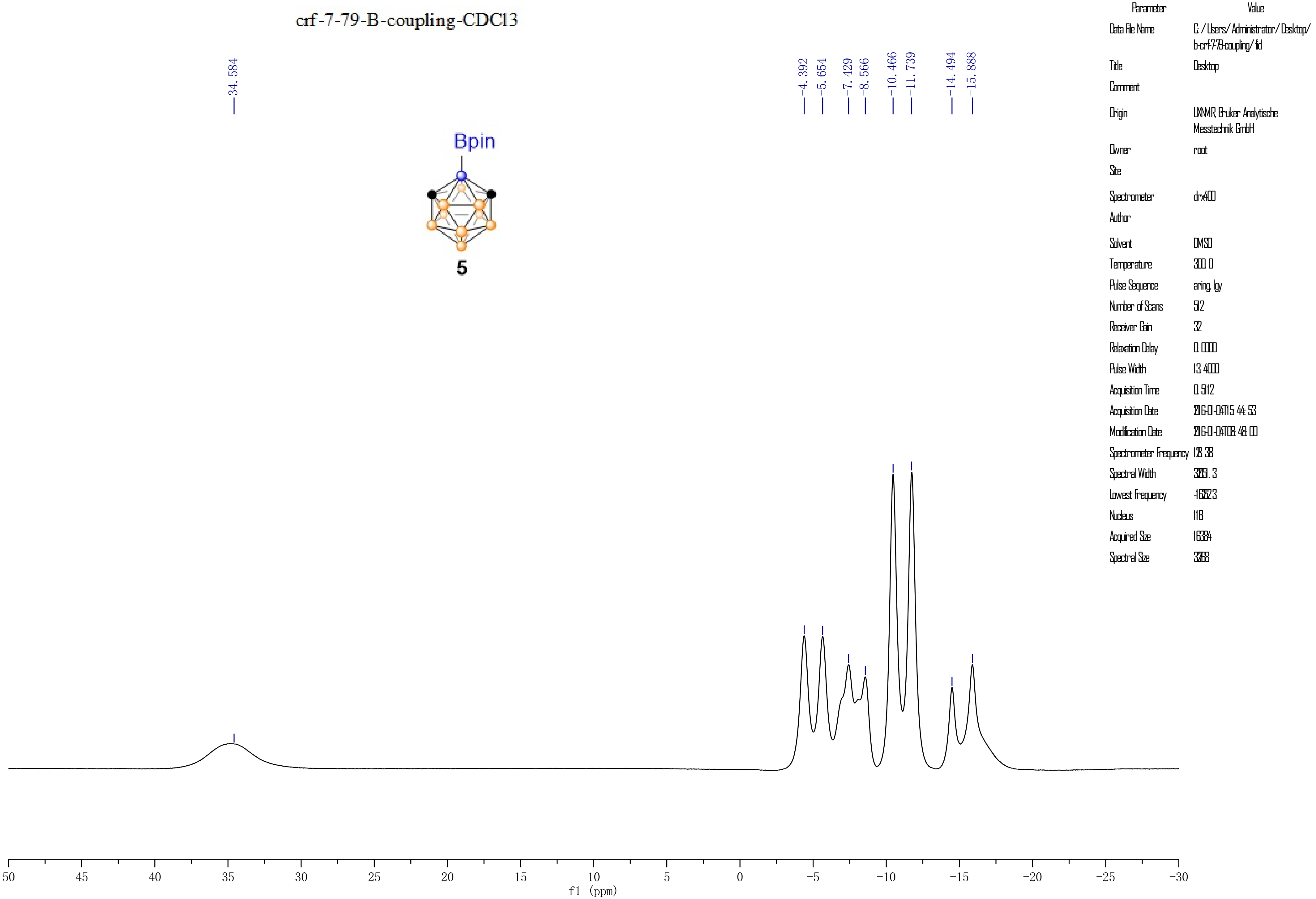

Supplementary Figure 110. ^{11}B NMR Spectrum of **5**.

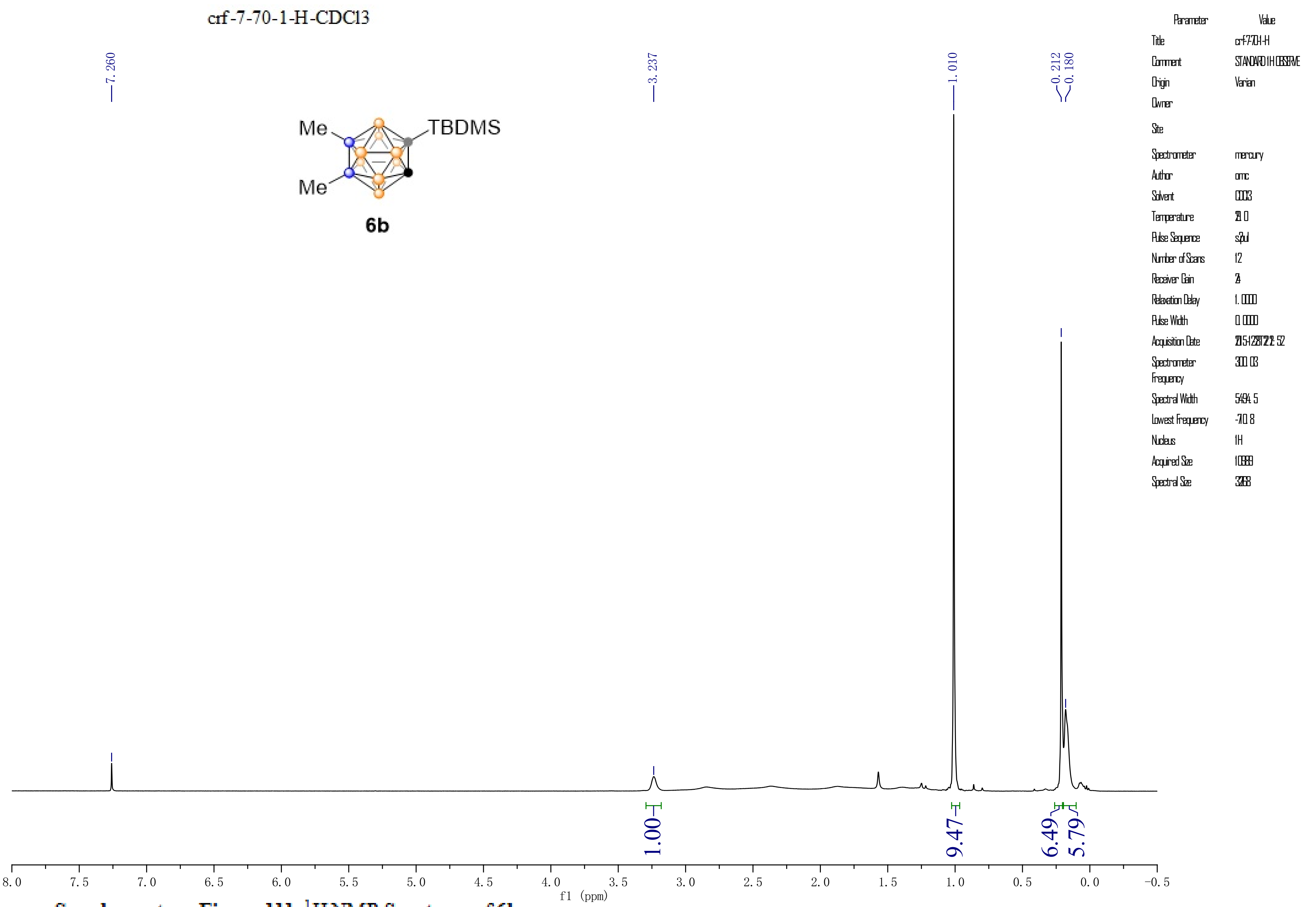

Supplementary Figure 111. ^{1}H NMR Spectrum of **6b**.

crf-7-70-1-C-CDCl3

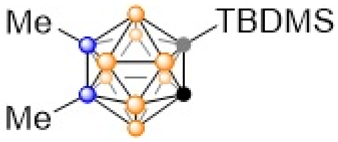

6b

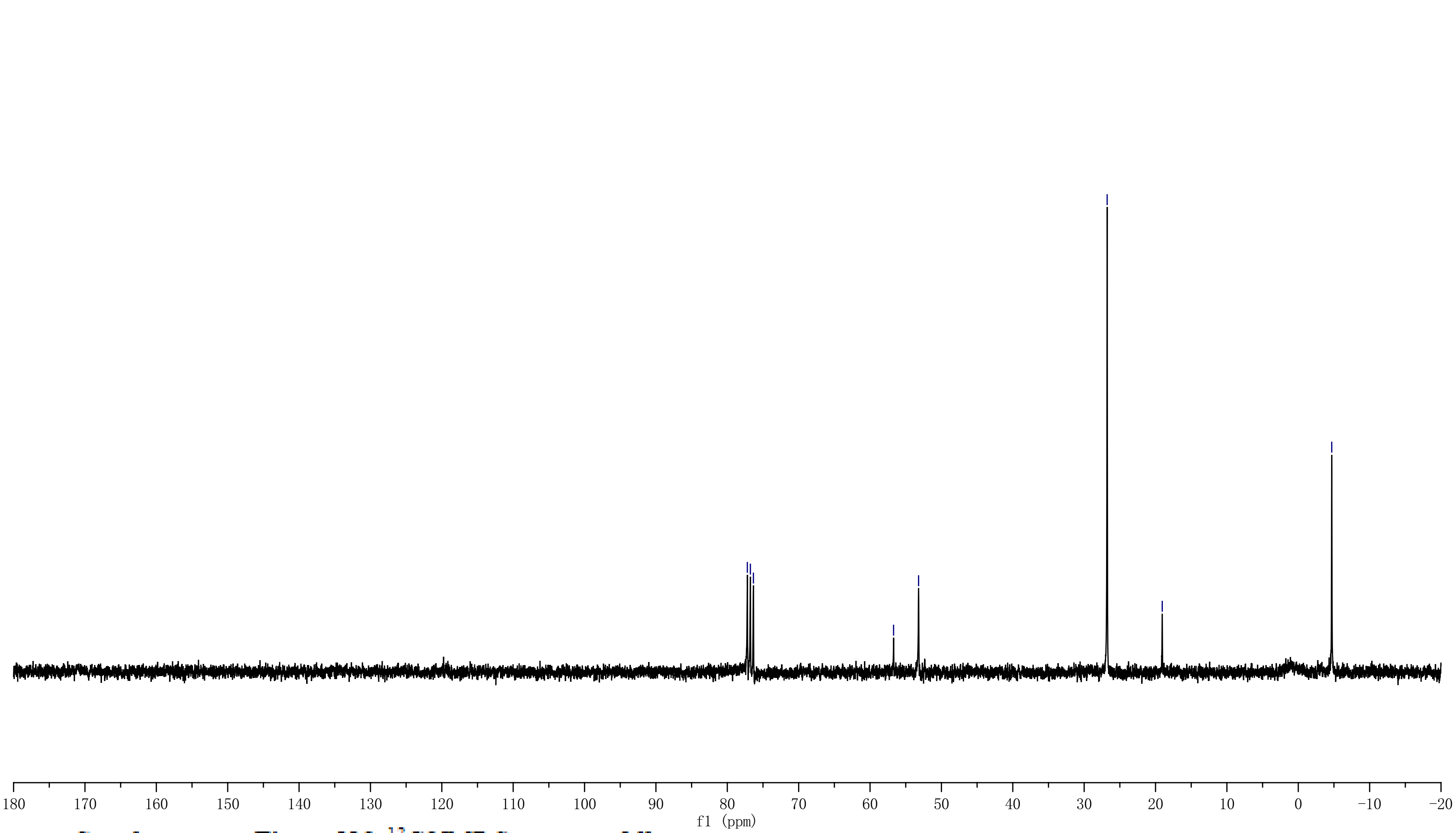

Parameter	Value
Title	crf-7-70-1-C
Comment	13C OBSERVE
Origin	Varian
Owner	
Site	
Spectrometer	mercury
Author	omc
Solvent	CDCl3
Temperature	[illegible]
Pulse Sequence	s2pul
Number of Scans	132
Receiver Gain	38
Relaxation Delay	1.0000
Pulse Width	0.0000
Acquisition Date	[illegible]
Spectrometer Frequency	75.45
Spectral Width	[illegible]
Lowest Frequency	[illegible]
Nucleus	13C
Acquired Size	30738
Spectral Size	65536

Supplementary Figure 112. ^{13}C NMR Spectrum of **6b**.

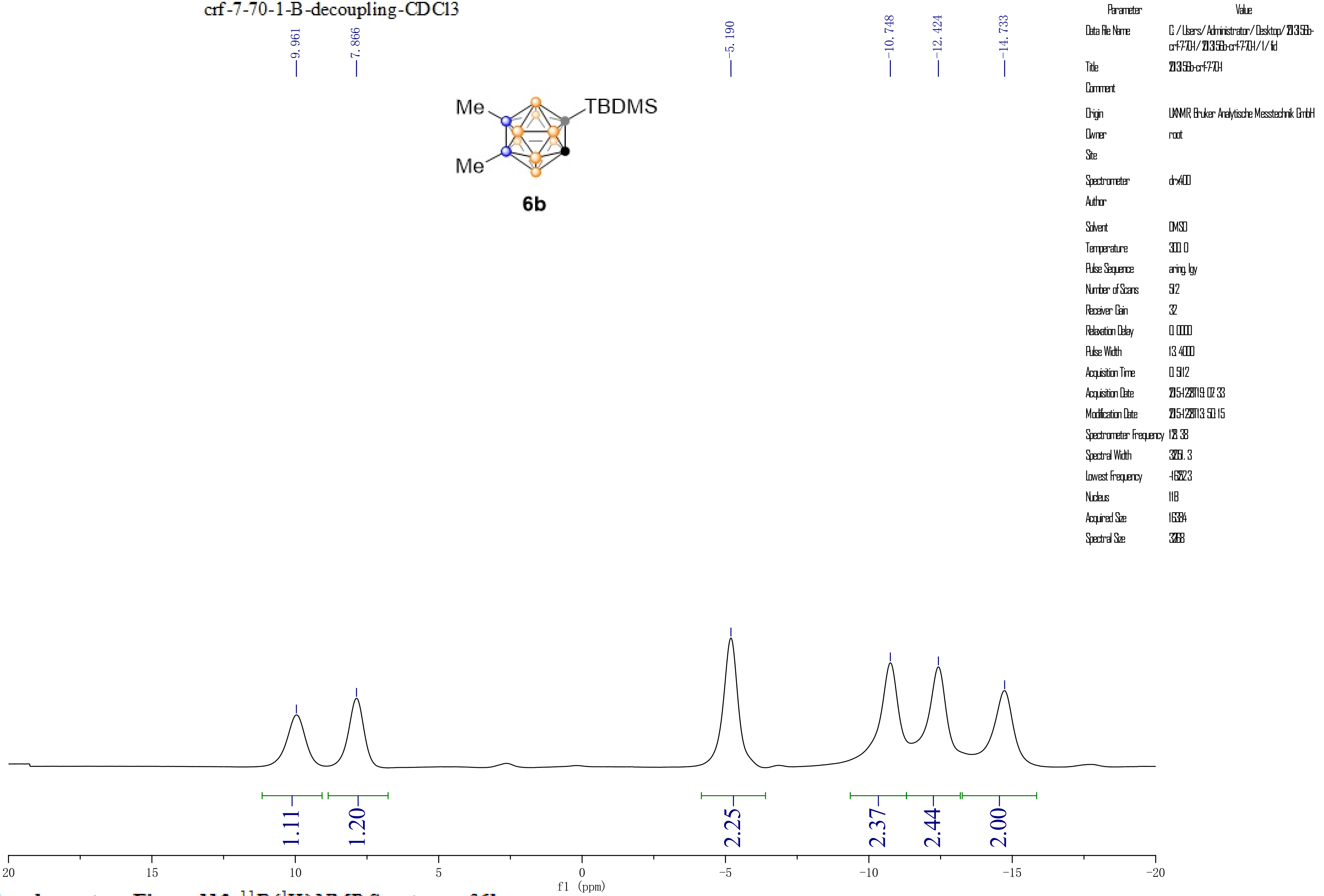

Supplementary Figure 113. $^{11}B\{^1H\}$ NMR Spectrum of **6b**.

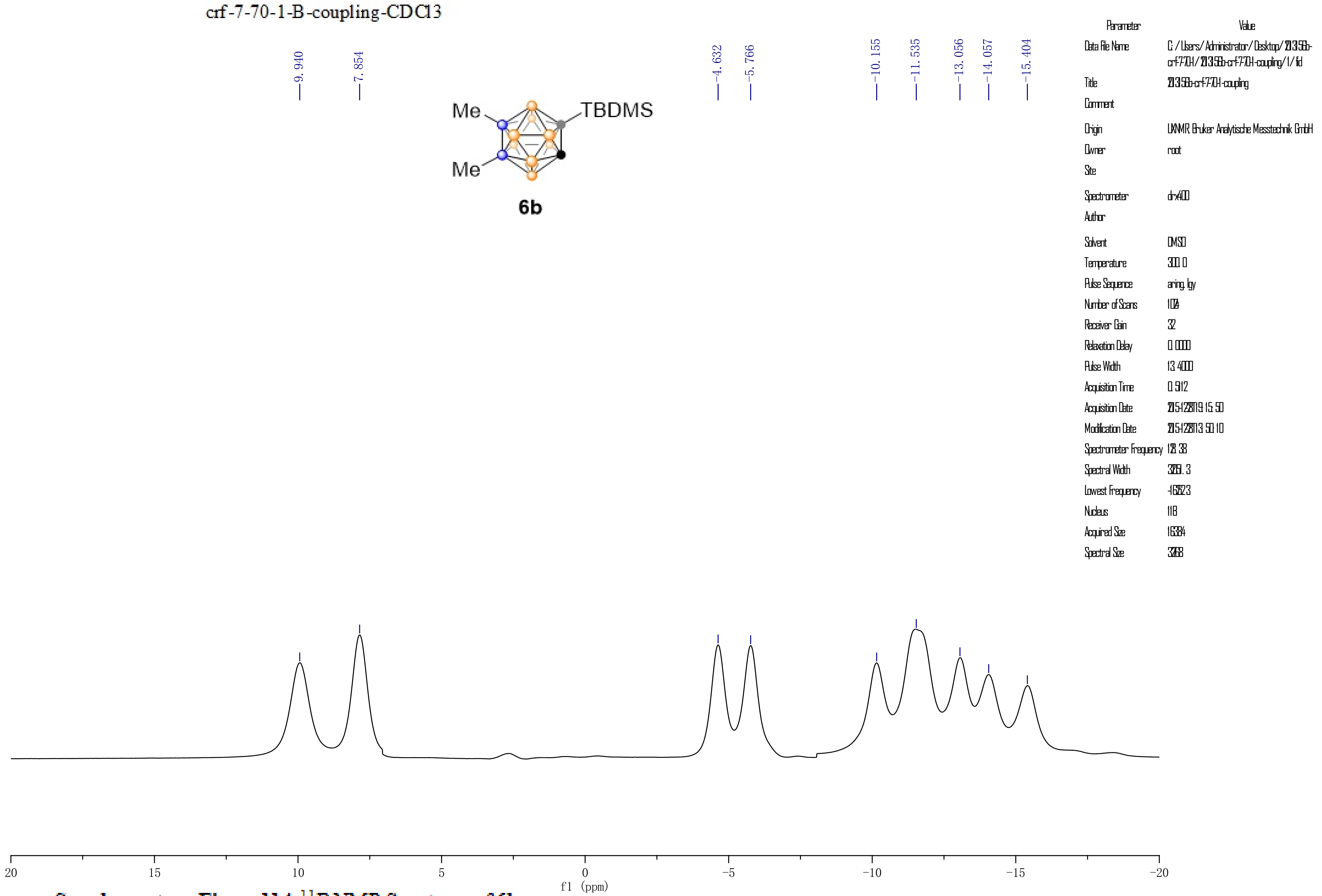

Supplementary Figure 114. ^{11}B NMR Spectrum of **6b**.

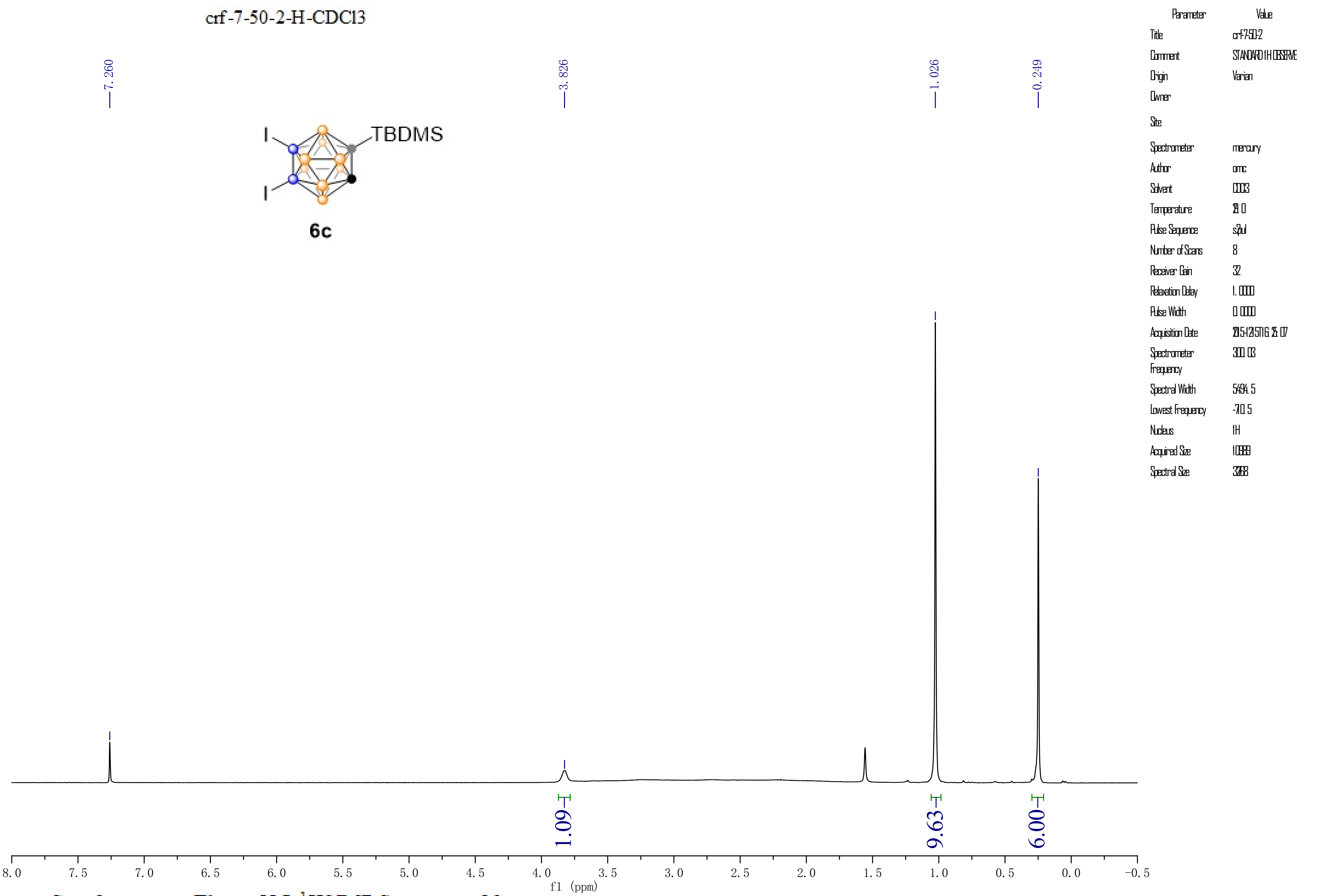

Supplementary Figure 115. ^{1}H NMR Spectrum of **6c**.

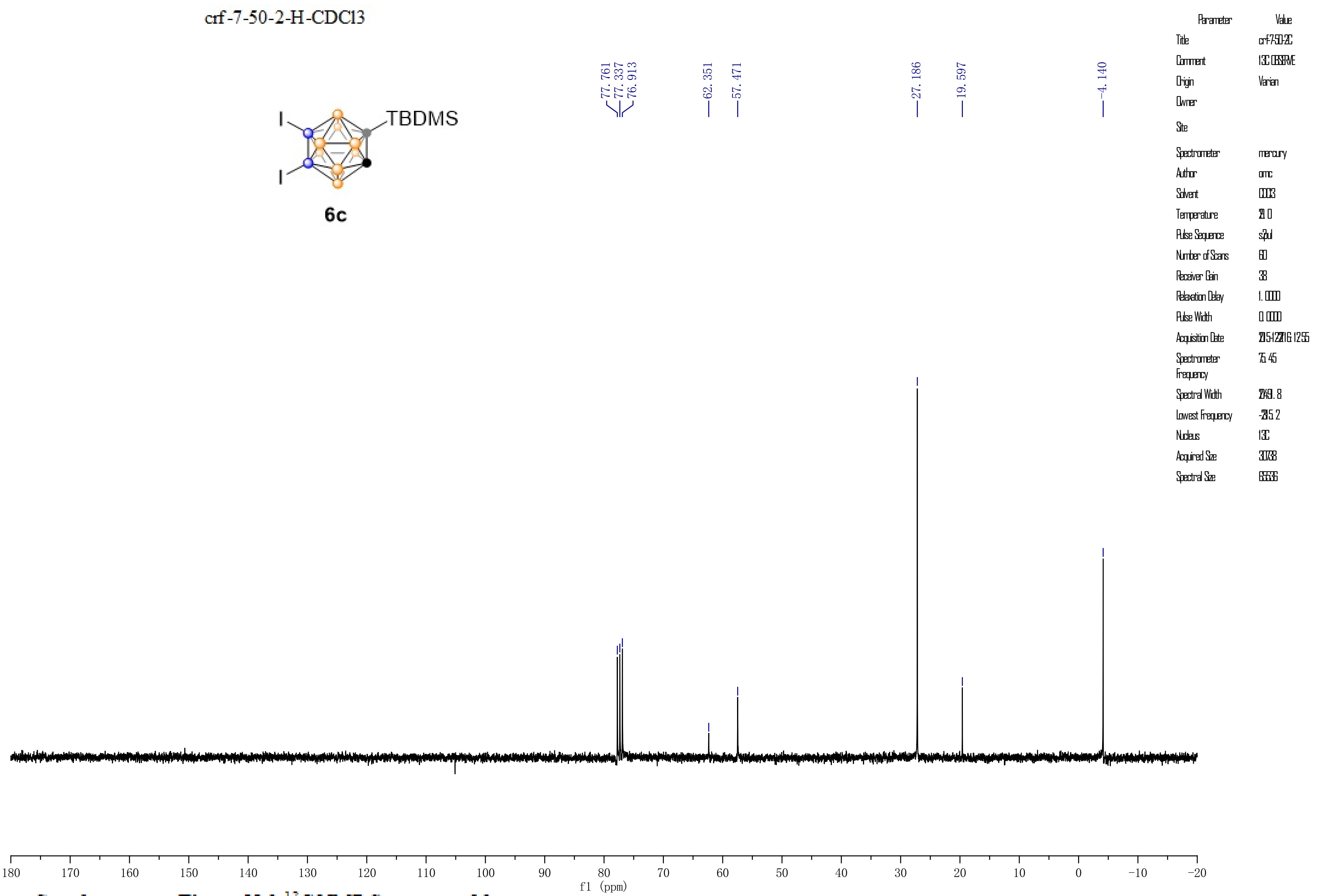

Supplementary Figure 116. ^{13}C NMR Spectrum of **6c**.

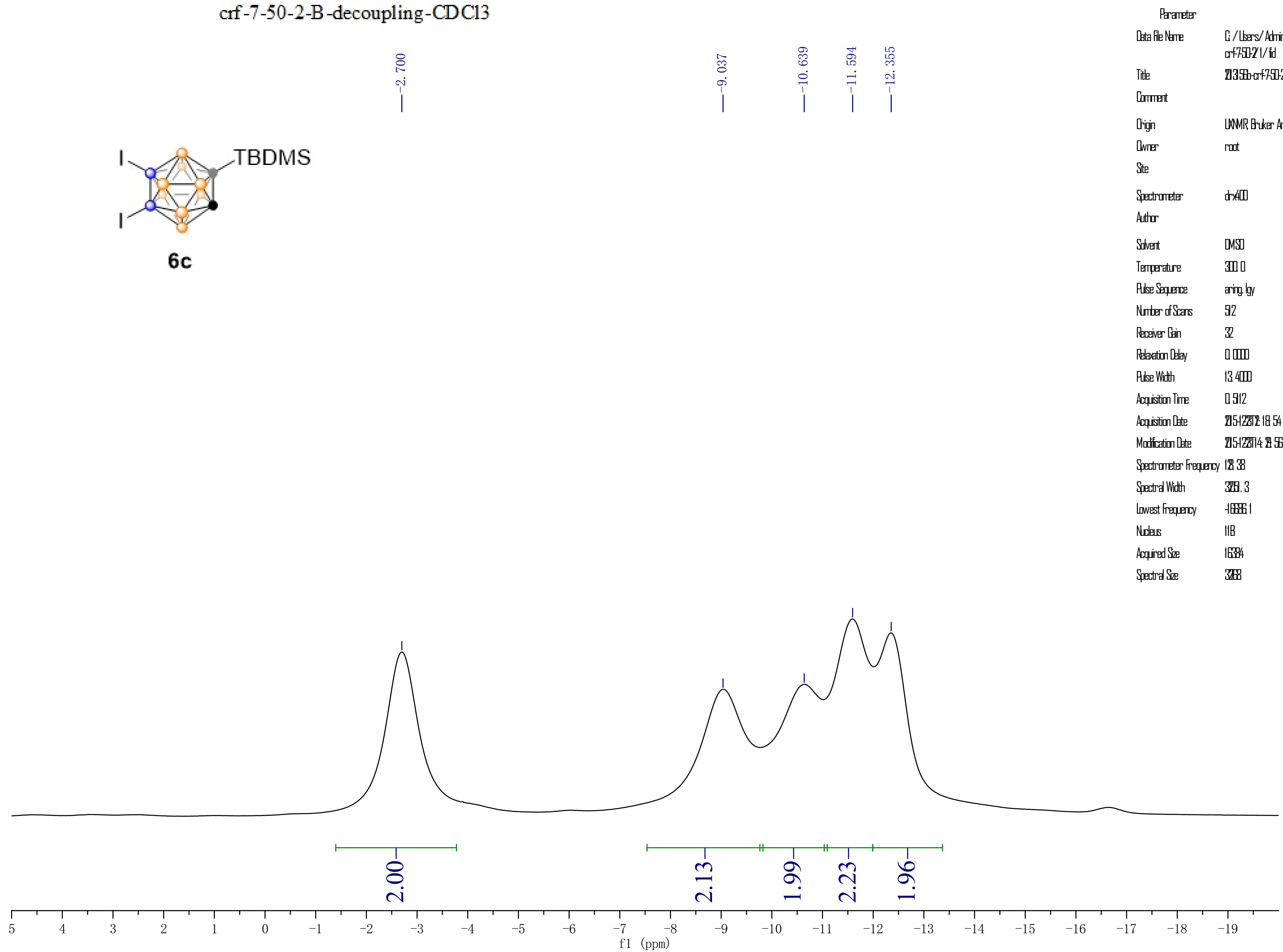

Parameter	Value
Data File Name	C:/Users/Administrator/Desktop/[illegible]-crf-7-50-2/1/fid
Title	[illegible]-crf-7-50-2
Comment	
Origin	UXNMR, Bruker Analytische Messtechnik GmbH
Owner	root
Site	
Spectrometer	drx400
Author	
Solvent	DMSO
Temperature	300.0
Pulse Sequence	zgig.lgy
Number of Scans	512
Receiver Gain	32
Relaxation Delay	0.0000
Pulse Width	13.4000
Acquisition Time	0.5112
Acquisition Date	2015-12-23T12:18:54
Modification Date	2015-12-23T14:28:56
Spectrometer Frequency	128.38
Spectral Width	32051.3
Lowest Frequency	-16686.1
Nucleus	11B
Acquired Size	16384
Spectral Size	32768

Supplementary Figure 117. $^{11}B\{^{1}H\}$ NMR Spectrum of **6c**.

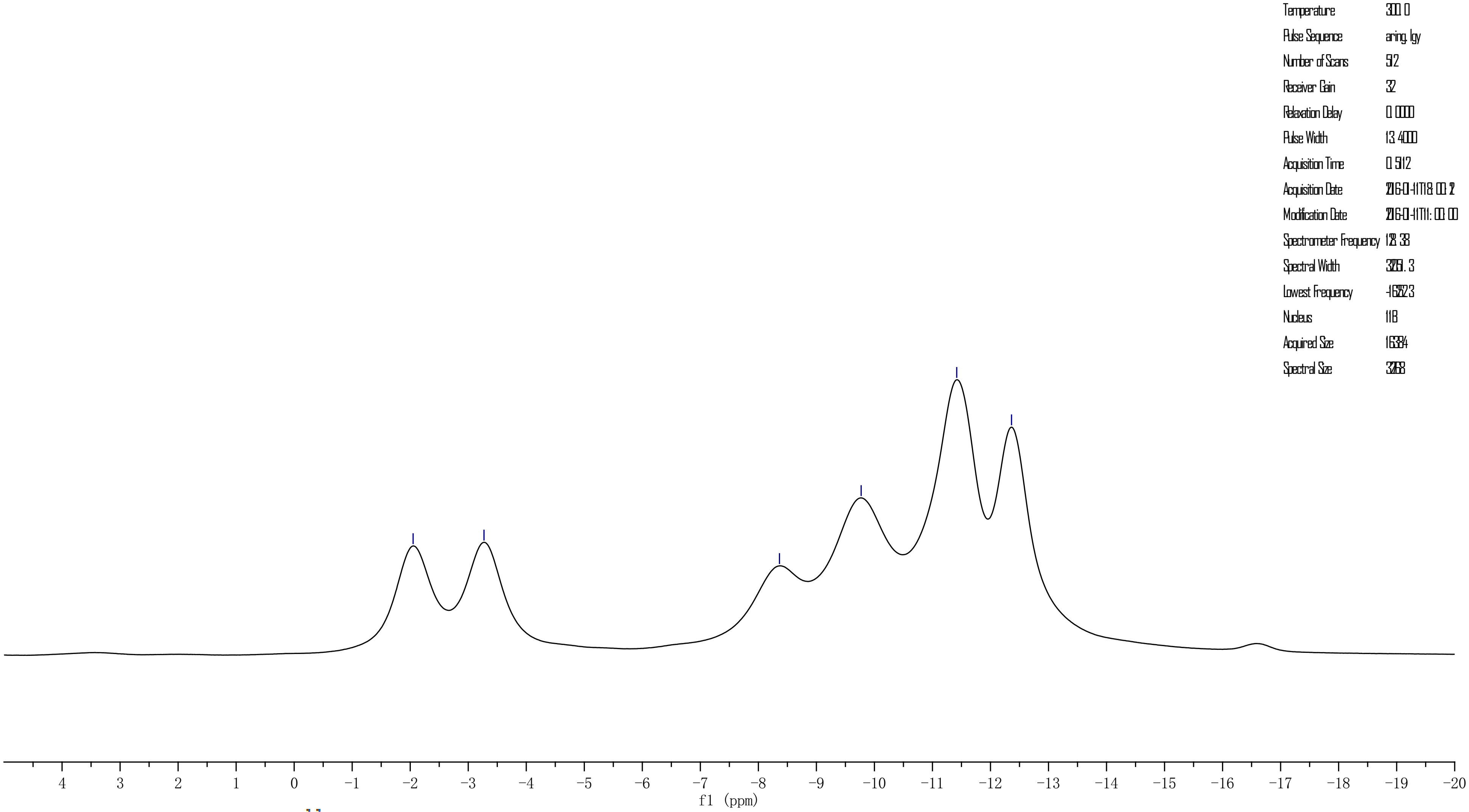

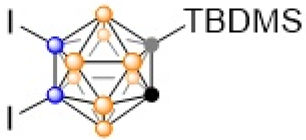

6c

Parameter	Value
Data File Name	C:/Users/Administrator/Desktop/work/送样NMR/boralatiom/7-50-2/b-crf-7-50-2coupling/fid
Title	7-50-2
Comment	
Origin	UXNMR, Bruker Analytische Messtechnik GmbH
Owner	root
Site	
Spectrometer	drx400
Author	
Solvent	DMSO
Temperature	300.0
Pulse Sequence	aring.lgy
Number of Scans	512
Receiver Gain	32
Relaxation Delay	0.0000
Pulse Width	13.4000
Acquisition Time	0.5112
Acquisition Date	2016-01-11T18:00:12
Modification Date	2016-01-11T11:00:00
Spectrometer Frequency	128.38
Spectral Width	32051.3
Lowest Frequency	-16252.3
Nucleus	11B
Acquired Size	16384
Spectral Size	32768

Supplementary Figure118. ^{11}B NMR Spectrum of **6c**.

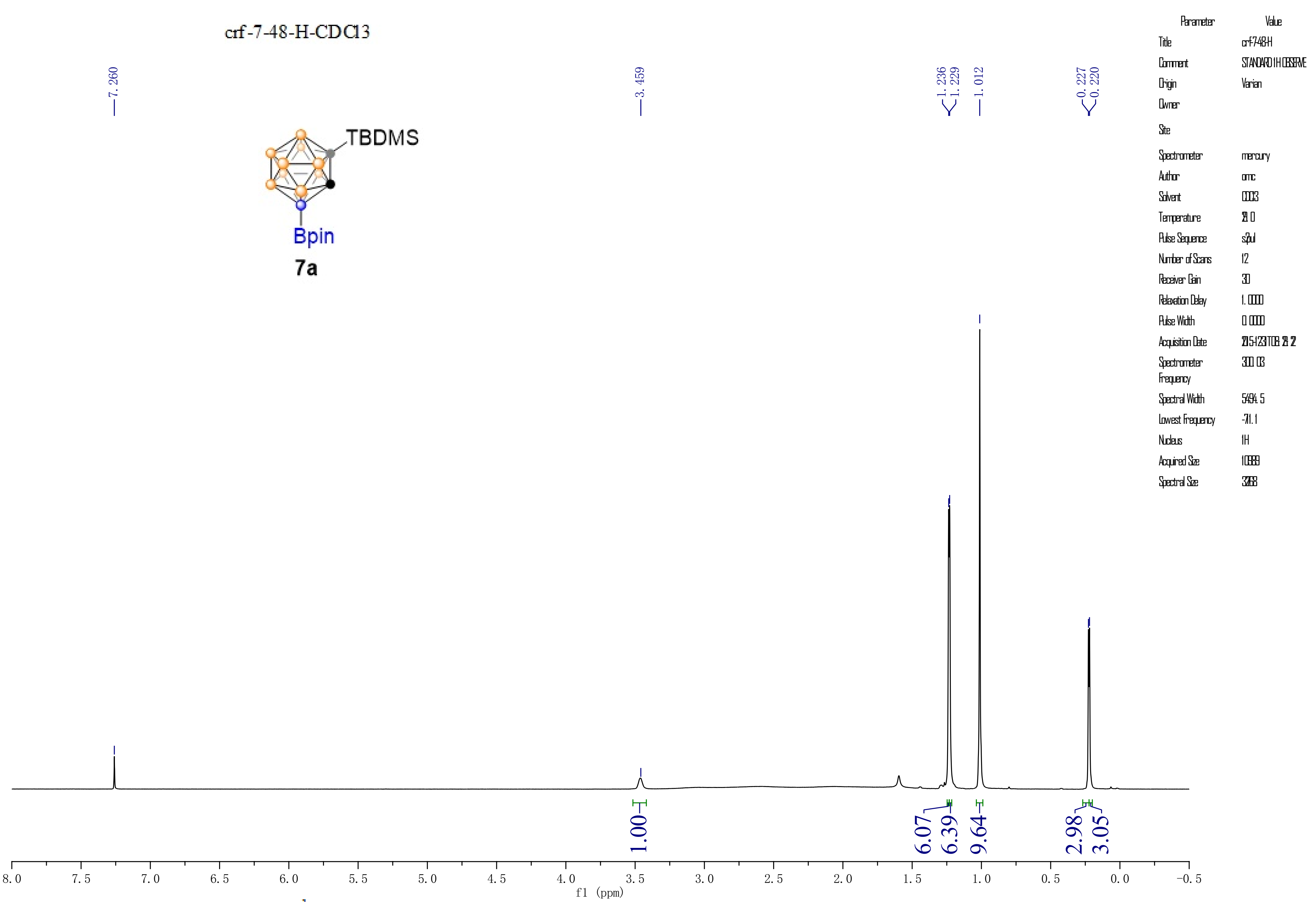

Supplementary Figure 119. ^{1}H NMR Spectrum of **7a**.

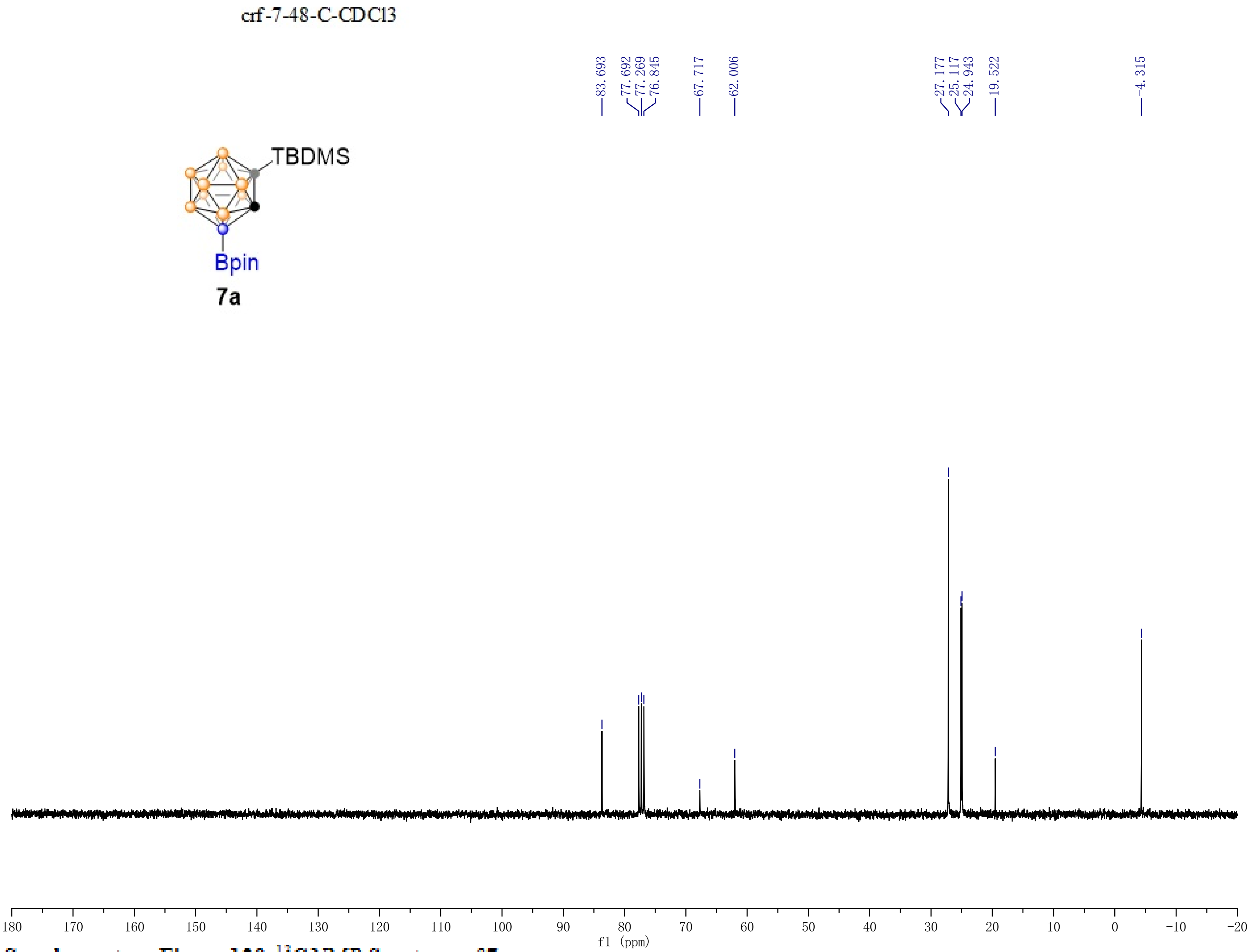

Parameter	Value
Title	crf-7-48-C
Comment	13C OBSERVE
Origin	Varian
Owner	
Site	
Spectrometer	mercury
Author	omc
Solvent	CDCl3
Temperature	[illegible]
Pulse Sequence	s2pul
Number of Scans	[illegible]
Receiver Gain	38
Relaxation Delay	1.0000
Pulse Width	[illegible]
Acquisition Date	[illegible]
Spectrometer Frequency	75.45
Spectral Width	[illegible]
Lowest Frequency	[illegible]
Nucleus	13C
Acquired Size	[illegible]
Spectral Size	65536

Supplementary Figure 120. ^{13}C NMR Spectrum of **7a**.

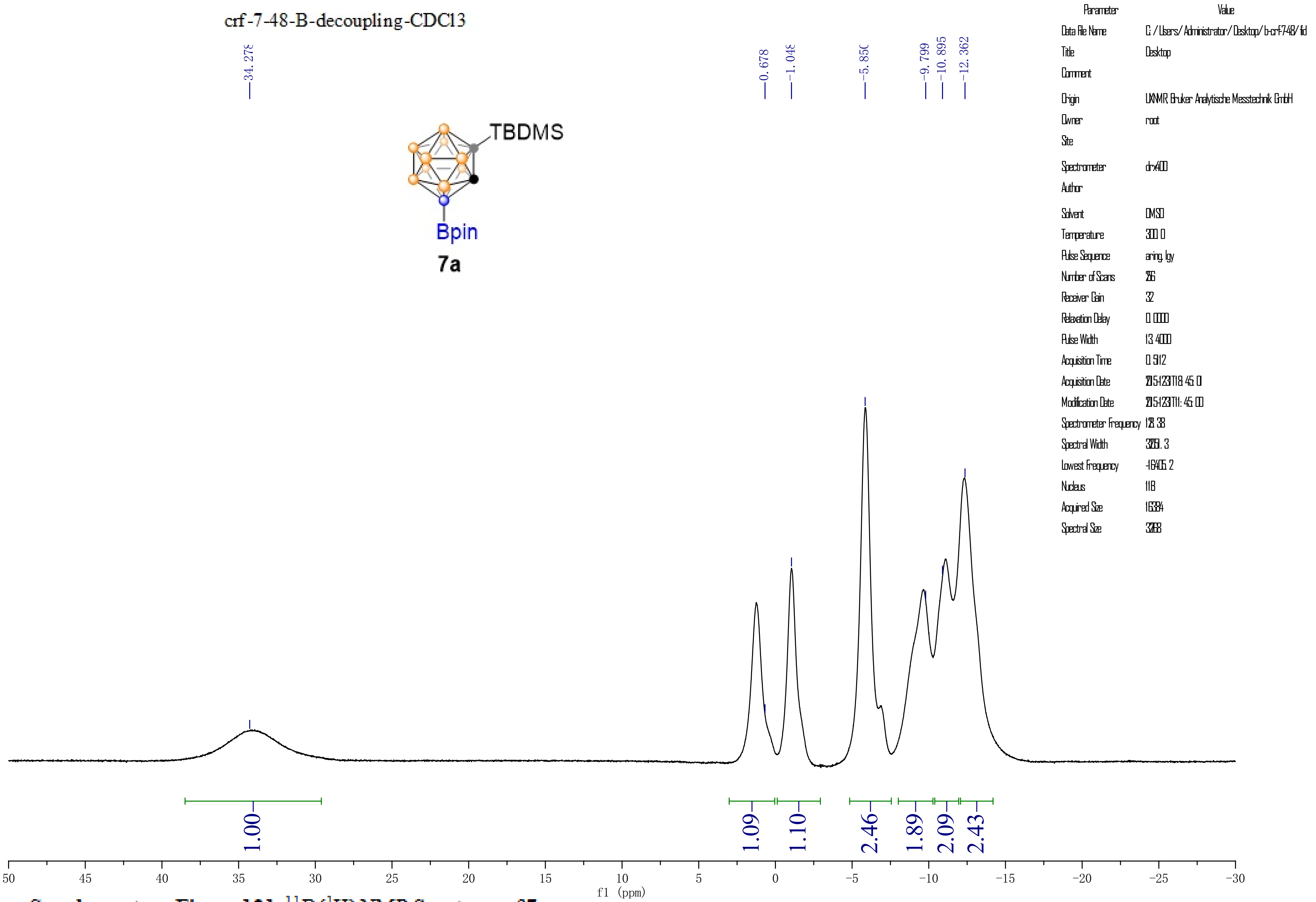

Supplementary Figure 121. $^{11}B\{^{1}H\}$ **NMR Spectrum of 7a.**

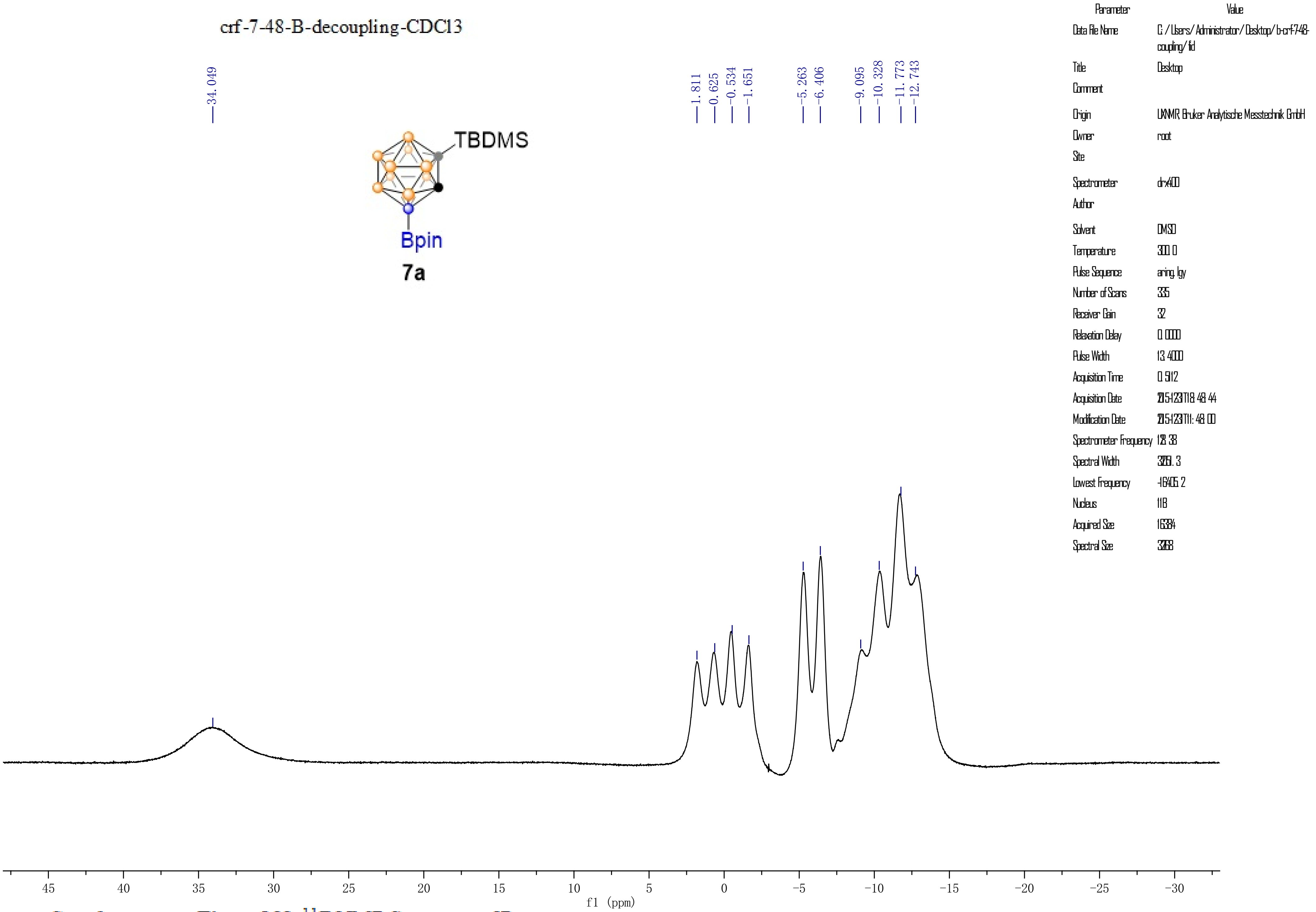

Supplementary Figure122. ^{11}B NMR Spectrum of **7a**.

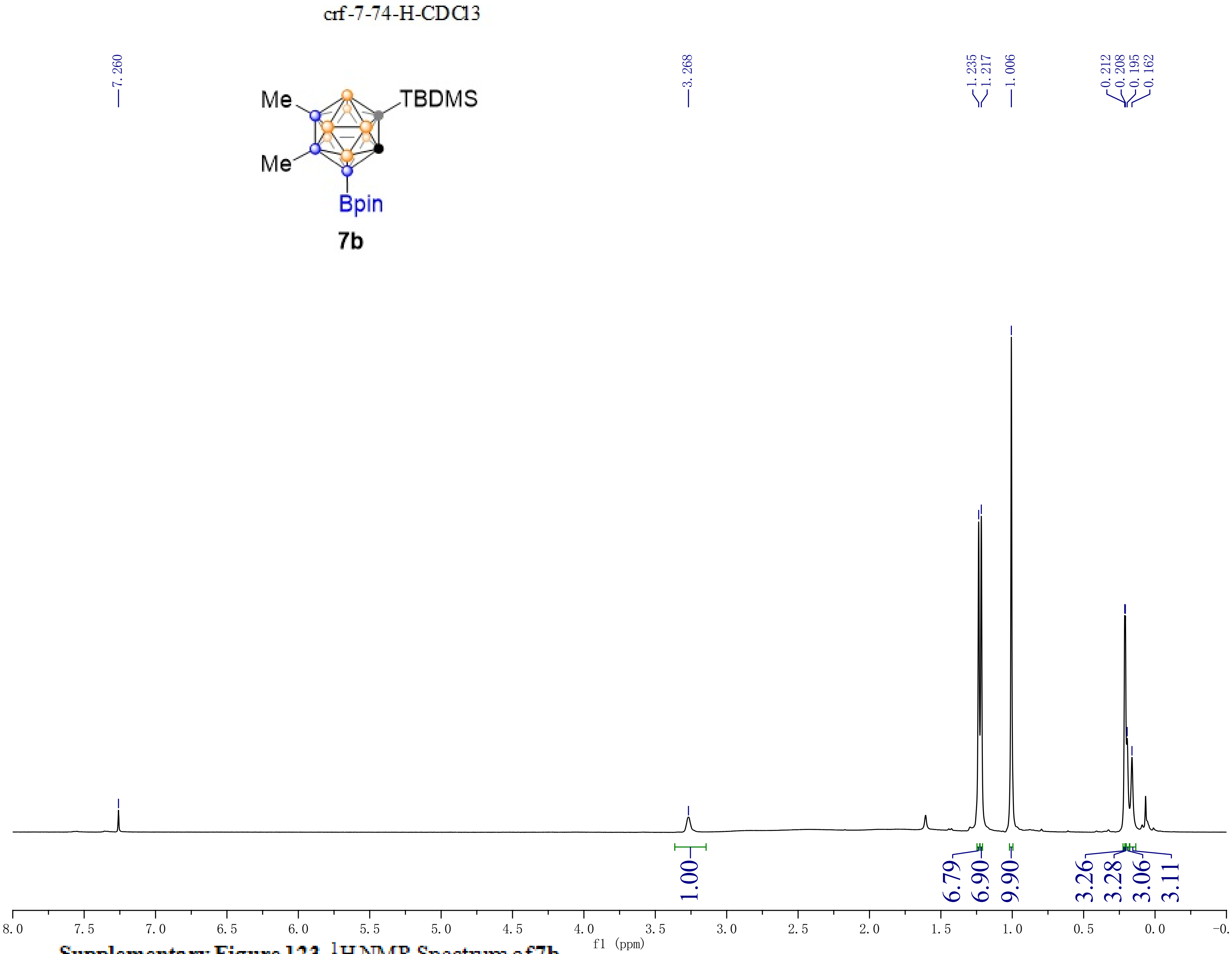

Parameter	Value
Title	crf-7-74-H
Comment	STANDARD 1H OBSERVE
Origin	Varian
Owner	
Site	
Spectrometer	mercury
Author	omc
Solvent	CDCl3
Temperature	21.0
Pulse Sequence	s2pul
Number of Scans	12
Receiver Gain	24
Relaxation Delay	1.0000
Pulse Width	0.0000
Acquisition Date	2015-12-31T09:33:45
Spectrometer Frequency	300.03
Spectral Width	5494.5
Lowest Frequency	-711.1
Nucleus	1H
Acquired Size	10989
Spectral Size	32768

Supplementary Figure 123. ^{1}H NMR Spectrum of **7b**.

crf-7-74-C-CDCl3

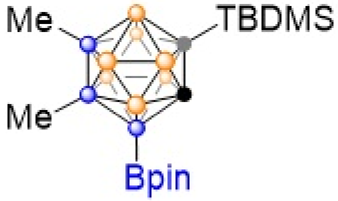

7b

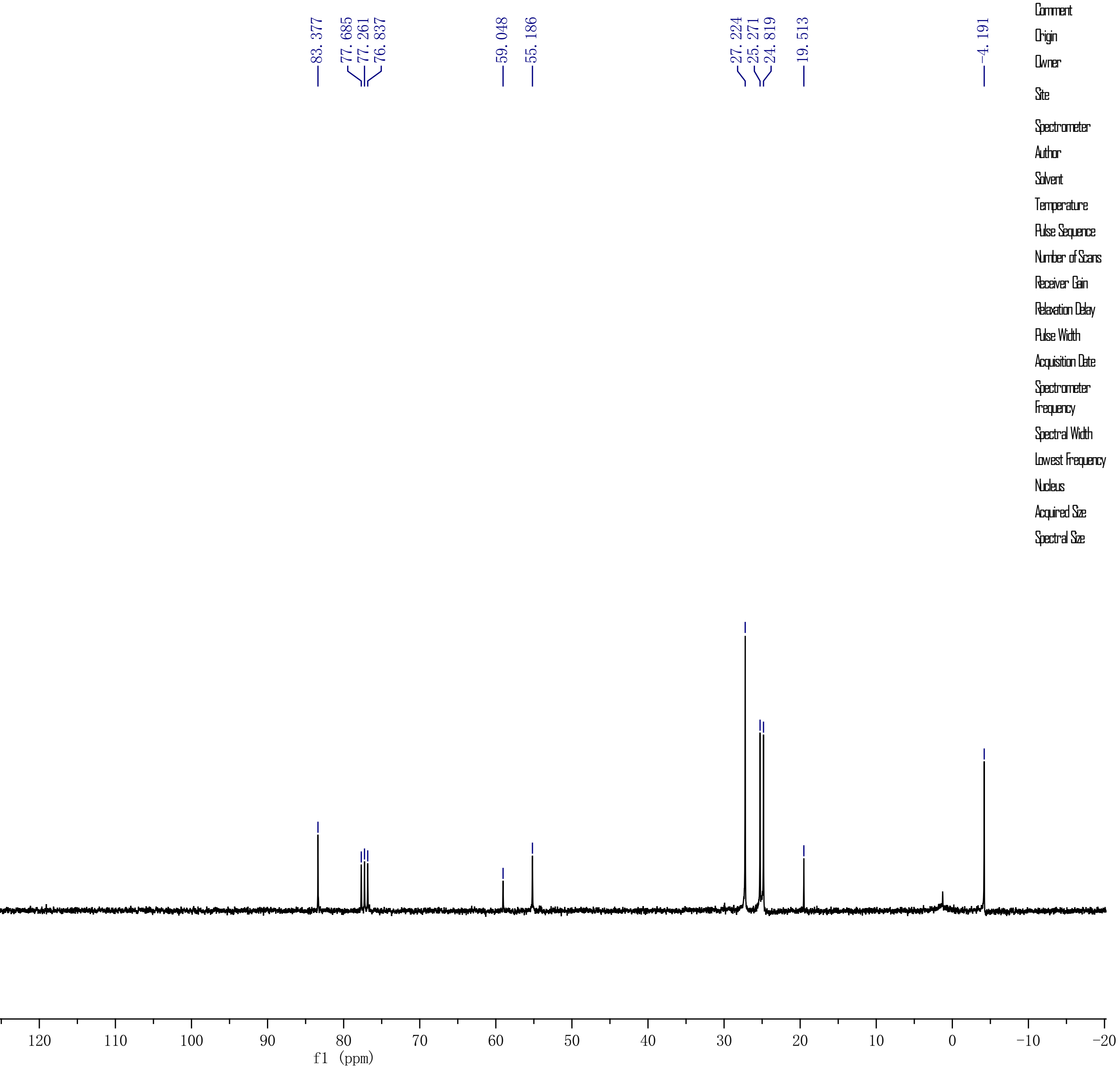

Parameter	Value
Title	crf-7-74-C
Comment	13C OBSERVE
Origin	Varian
Owner	
Site	
Spectrometer	mercury
Author	omc
Solvent	CDCl3
Temperature	21.0
Pulse Sequence	s2pul
Number of Scans	84
Receiver Gain	38
Relaxation Delay	1.0000
Pulse Width	0.0000
Acquisition Date	2015-12-31T09:53:43
Spectrometer Frequency	75.45
Spectral Width	20491.8
Lowest Frequency	-2815.2
Nucleus	13C
Acquired Size	30738
Spectral Size	65536

Supplementary Figure 124. ^{13}C NMR Spectrum of **7b**.

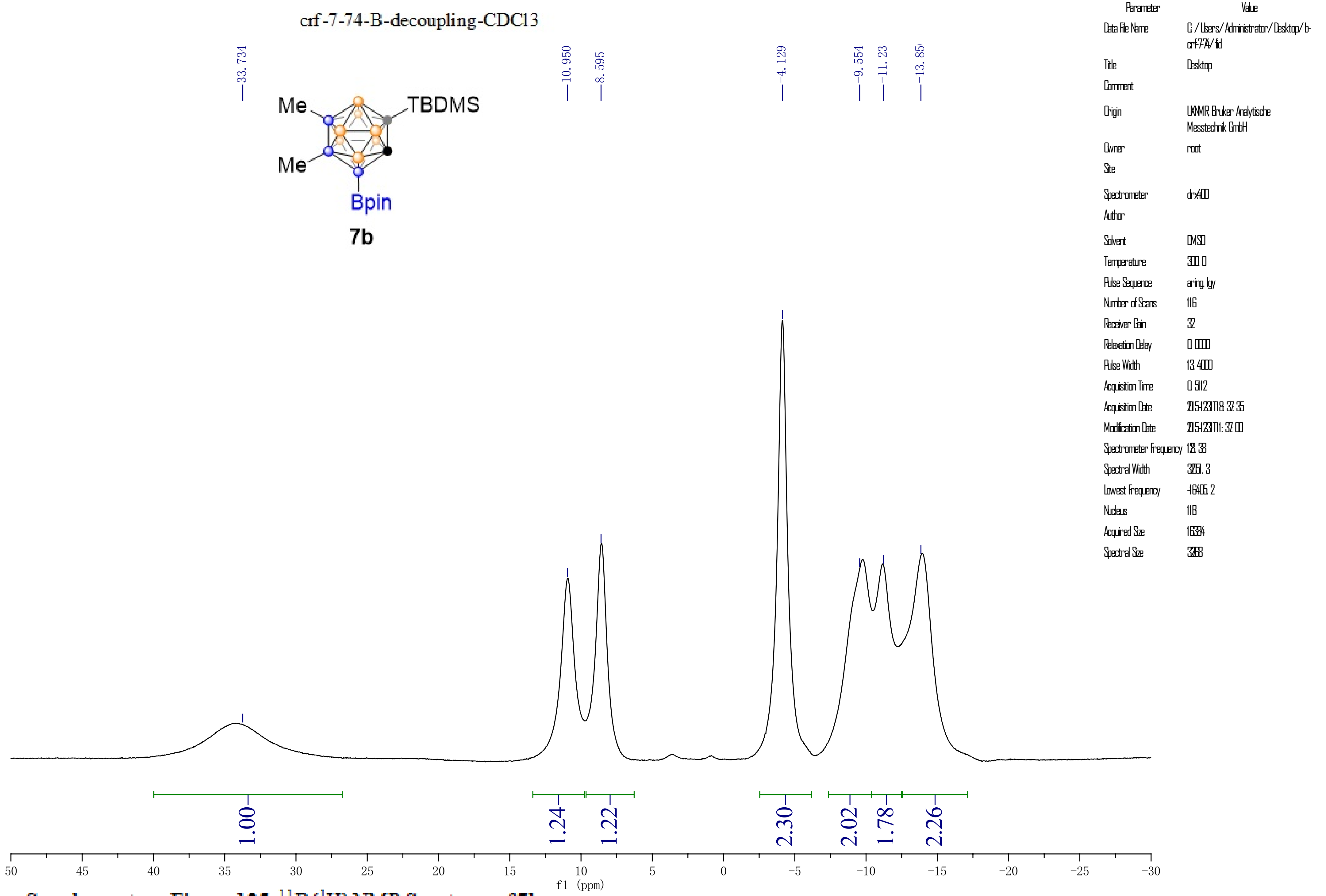

Supplementary Figure 125. $^{11}B\{^{1}H\}$ NMR Spectrum of **7b**.

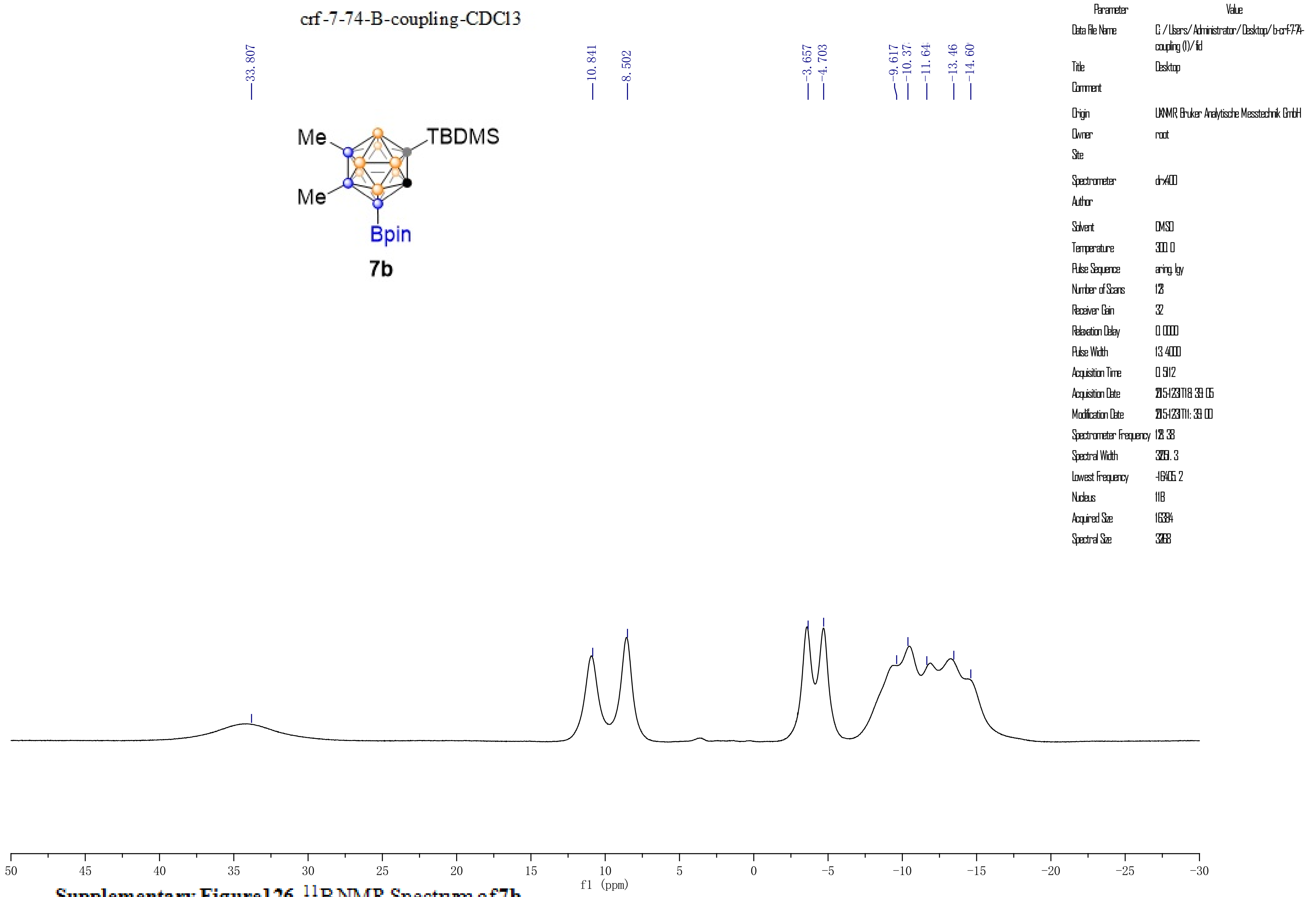

Supplementary Figure126. ^{11}B NMR Spectrum of **7b**.

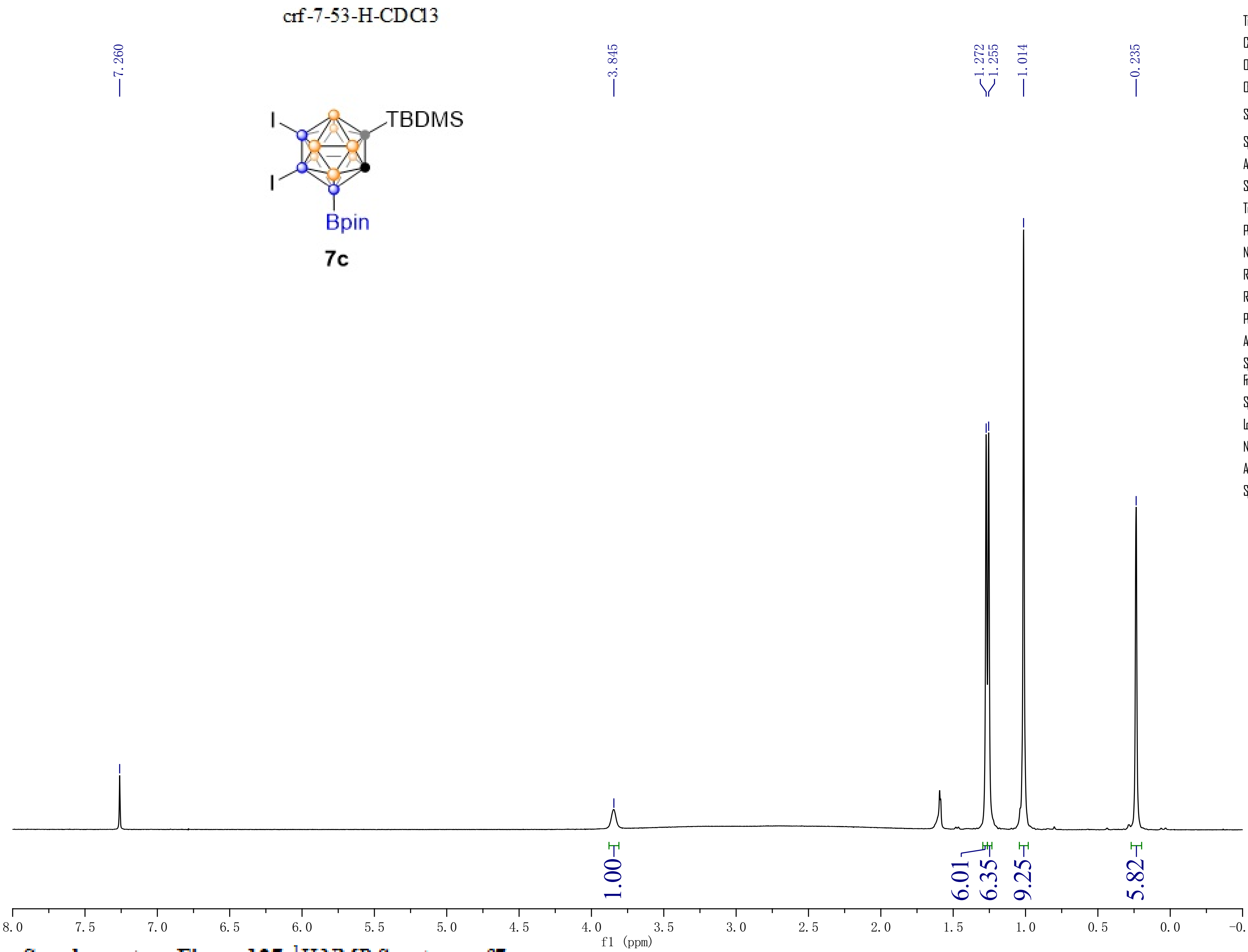

Parameter	Value
Title	crf-7-53-H-0107
Comment	STANDARD 1H OBSERVE
Origin	Varian
Owner	
Site	
Spectrometer	mercury
Author	omc
Solvent	CDCl3
Temperature	21.0
Pulse Sequence	s2pul
Number of Scans	8
Receiver Gain	32
Relaxation Delay	1.0000
Pulse Width	0.0000
Acquisition Date	2016-01-07T11:04:04
Spectrometer Frequency	300.03
Spectral Width	5494.5
Lowest Frequency	-711.3
Nucleus	1H
Acquired Size	10989
Spectral Size	32768

Supplementary Figure 127. ^{1}H NMR Spectrum of **7c**.

crf-7-53-C-CDCl3

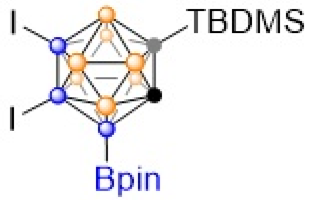

7c

Parameter	Value
Title	crf-7-53-C
Comment	13C OBSERVE
Origin	Varian
Owner	
Site	
Spectrometer	mercury
Author	omc
Solvent	CDCl3
Temperature	21.0
Pulse Sequence	s2pul
Number of Scans	164
Receiver Gain	38
Relaxation Delay	1.0000
Pulse Width	0.0000
Acquisition Date	2016-01-07T11:22:53
Spectrometer Frequency	75.45
Spectral Width	20491.8
Lowest Frequency	-2815.2
Nucleus	13C
Acquired Size	30738
Spectral Size	65536

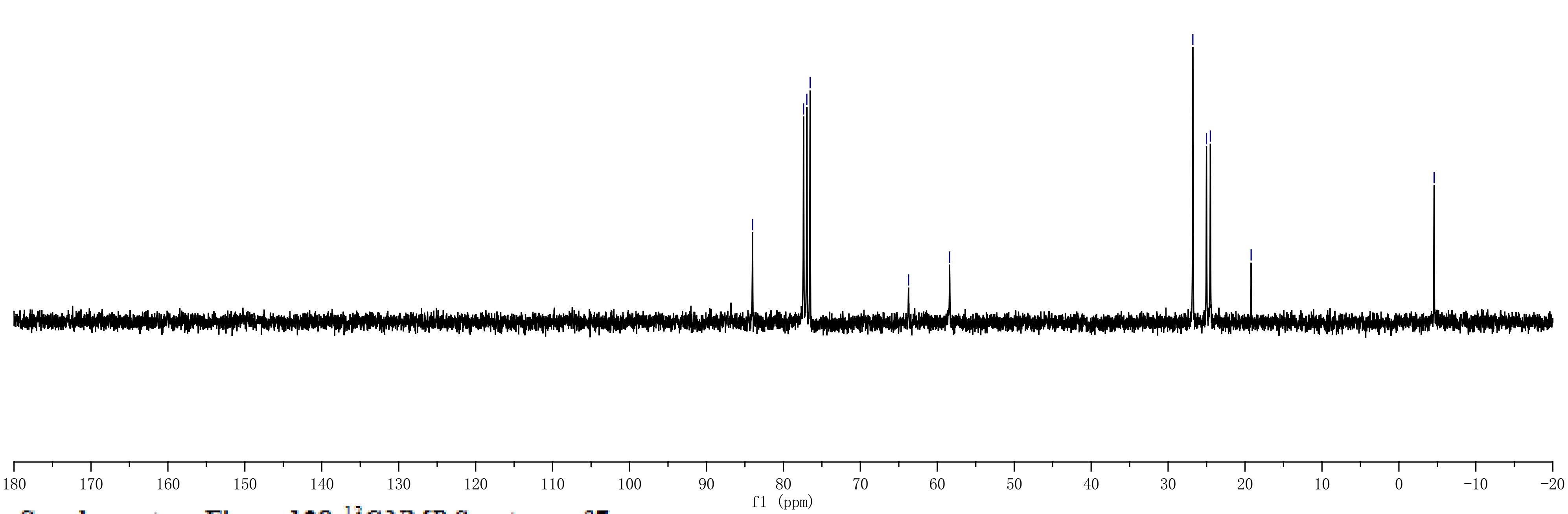

Supplementary Figure 128. ^{13}C NMR Spectrum of **7c**.

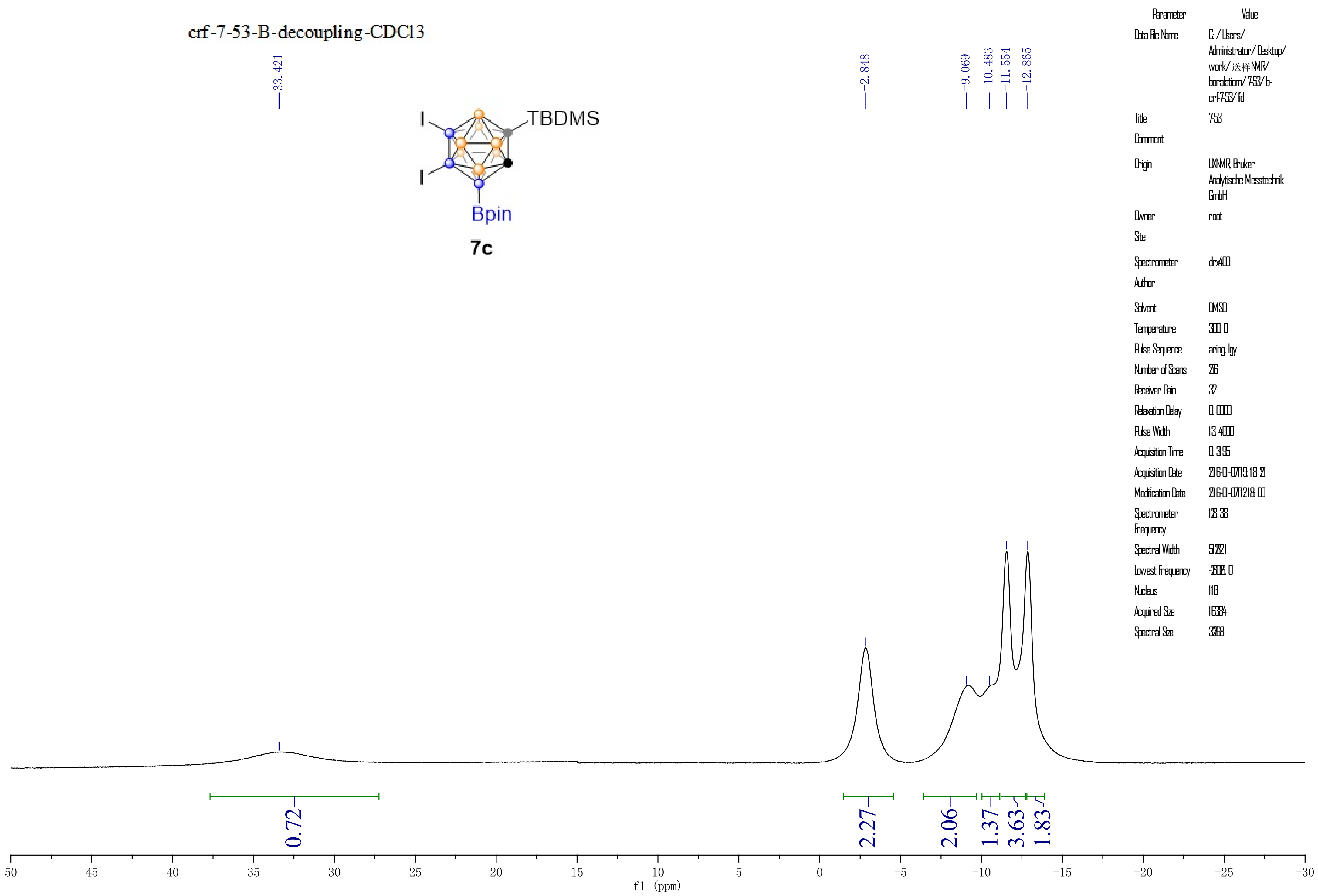

Supplementary Figure 129. $^{11}B\{^{1}H\}$ NMR Spectrum of **7c**.

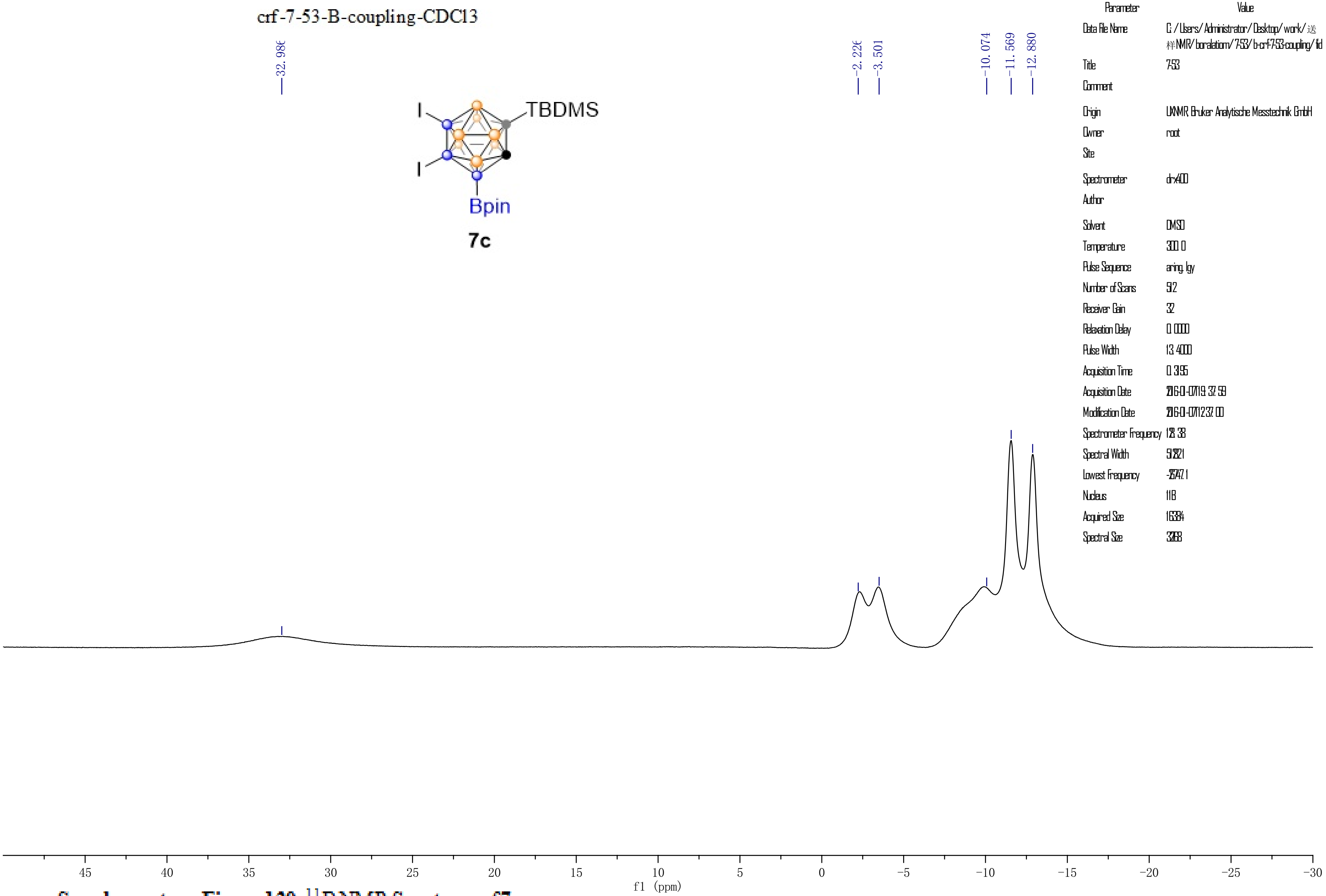

Supplementary Figure130. ^{11}B NMR Spectrum of **7c**.

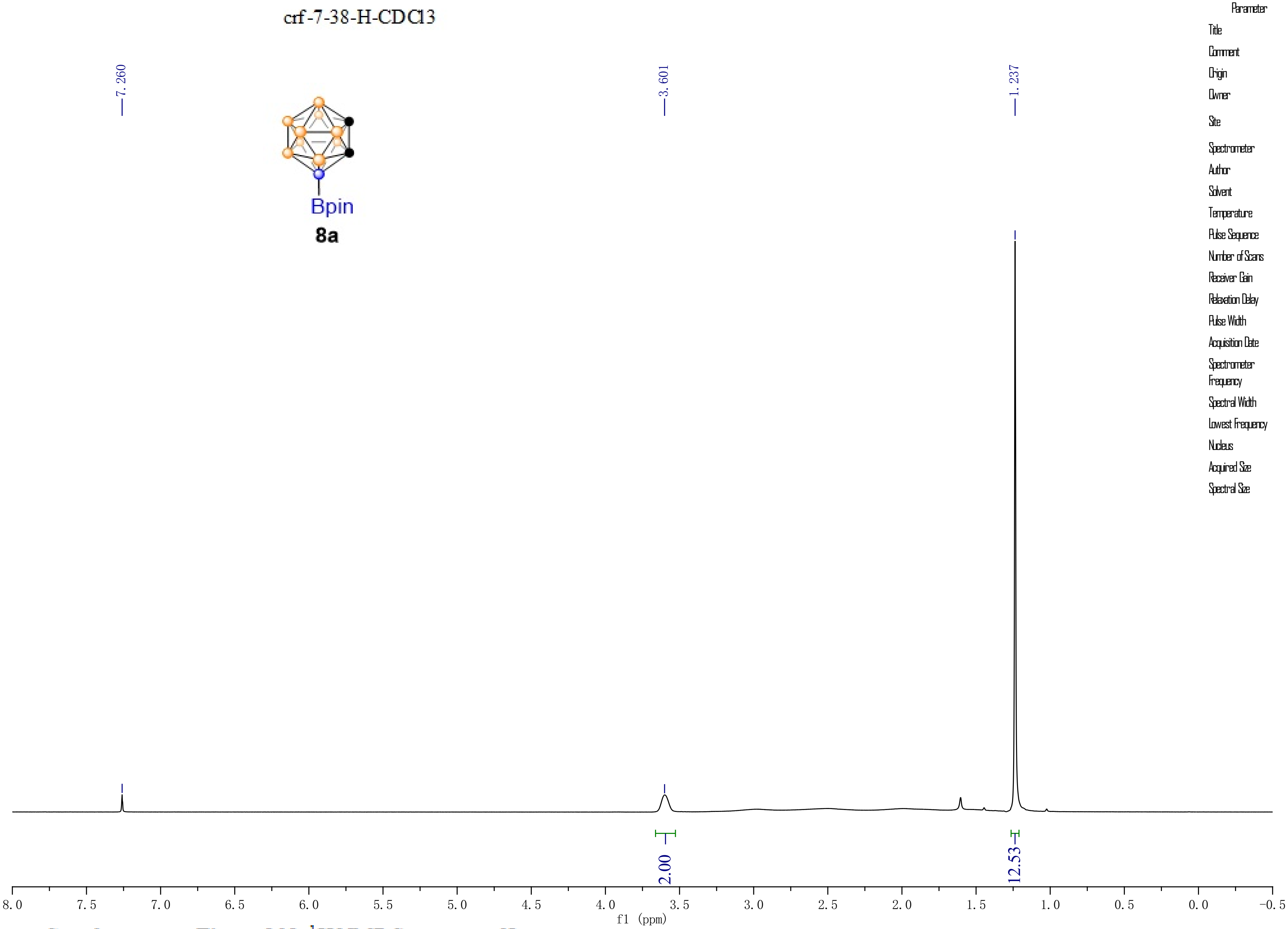

Parameter	Value
Title	crf-7-38-H24
Comment	STANDARD 1H OBSERVE
Origin	Varian
Owner	
Site	
Spectrometer	mercury
Author	omc
Solvent	CDCl3
Temperature	21.0
Pulse Sequence	s2pul
Number of Scans	16
Receiver Gain	30
Relaxation Delay	1.0000
Pulse Width	0.0000
Acquisition Date	2015-12-24T11:13:56
Spectrometer Frequency	300.03
Spectral Width	5494.5
Lowest Frequency	-710.5
Nucleus	1H
Acquired Size	10989
Spectral Size	32768

Supplementary Figure 131. ^{1}H NMR Spectrum of **8a**.

crf-7-38-C-CDCl3

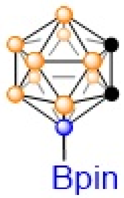

8a

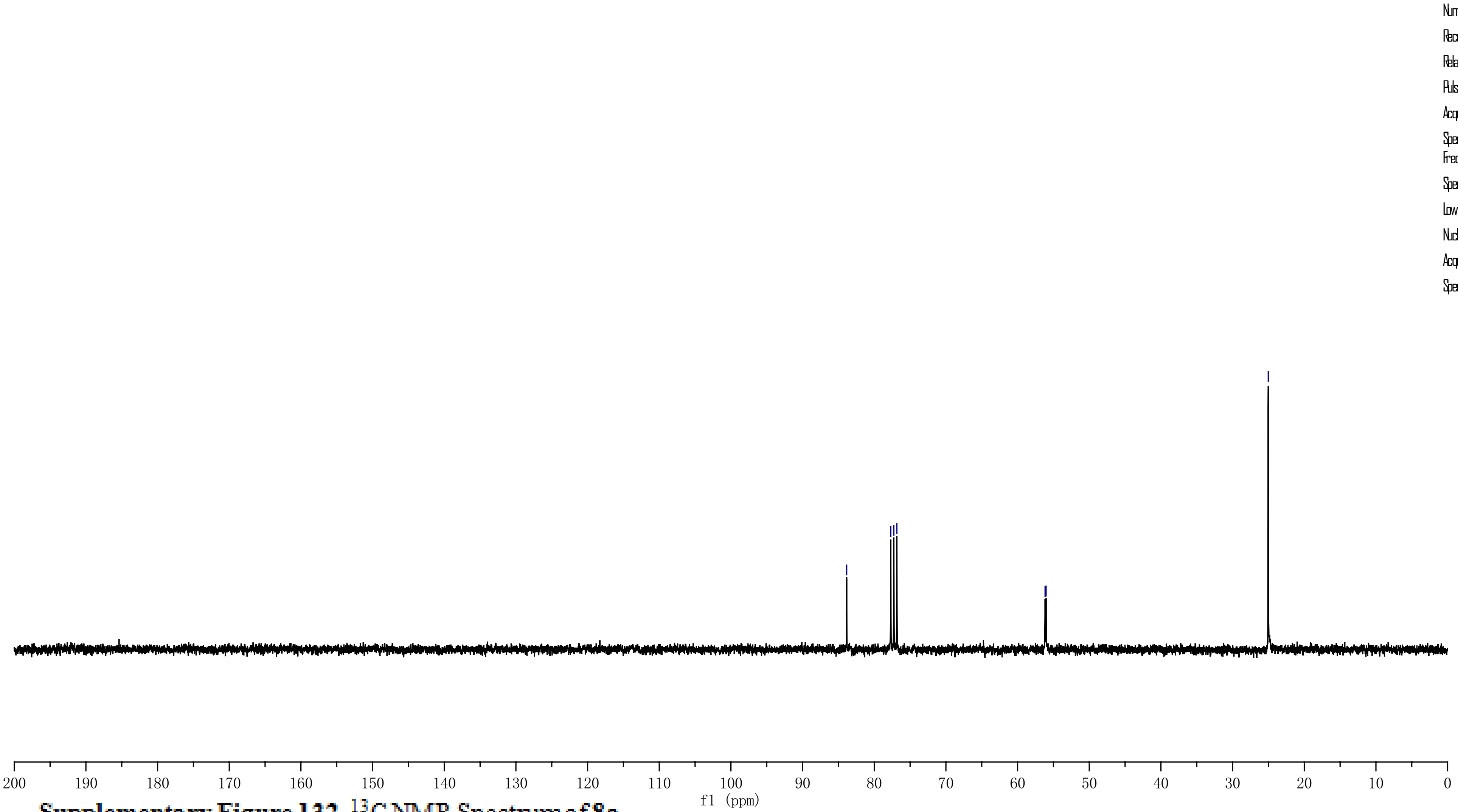

Parameter	Value
Title	crf-7-38-C-1214
Comment	13C OBSERVE
Origin	Varian
Owner	
Site	
Spectrometer	mercury
Author	omc
Solvent	CDCl3
Temperature	[illegible]
Pulse Sequence	s2pul
Number of Scans	[illegible]
Receiver Gain	34
Relaxation Delay	1.0000
Pulse Width	0.0000
Acquisition Date	2015-12-14T11:17:17
Spectrometer Frequency	75.45
Spectral Width	[illegible]
Lowest Frequency	[illegible]
Nucleus	13C
Acquired Size	[illegible]
Spectral Size	65536

Supplementary Figure 132. ^{13}C NMR Spectrum of **8a**.

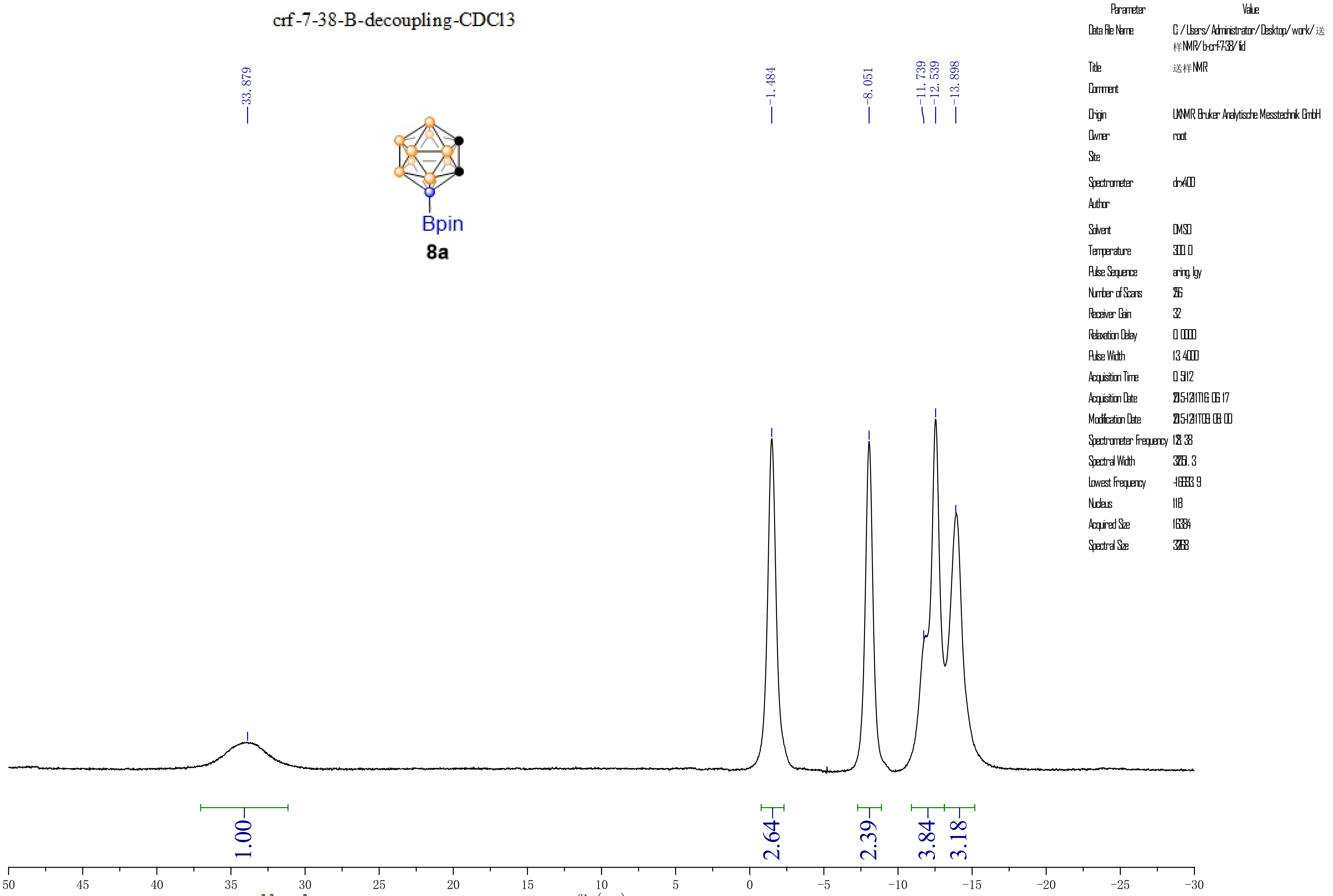

Supplementary Figure 133. $^{11}B\{^{1}H\}$ NMR Spectrum of **8a**.

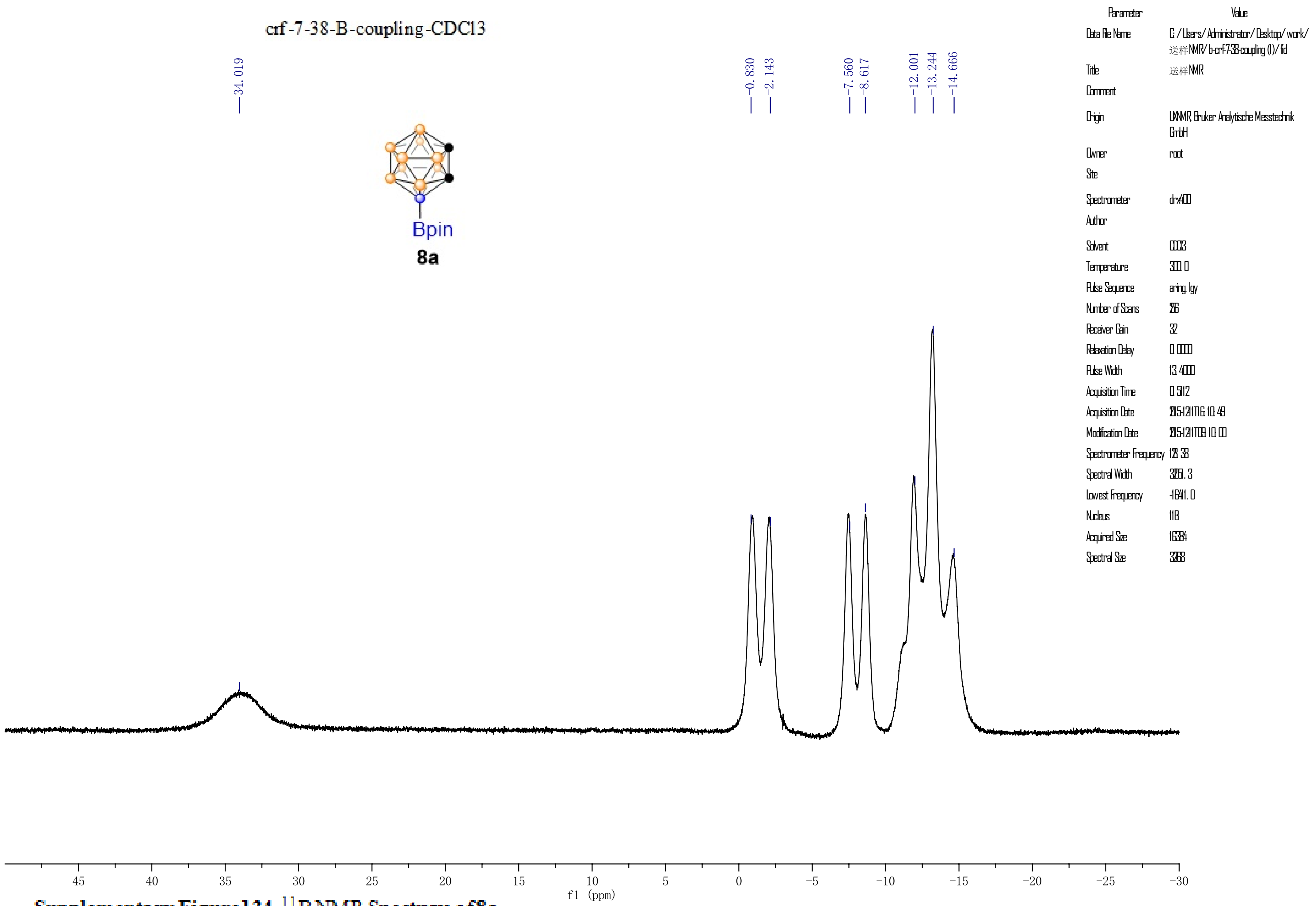

Supplementary Figure134. ^{11}B NMR Spectrum of **8a**.

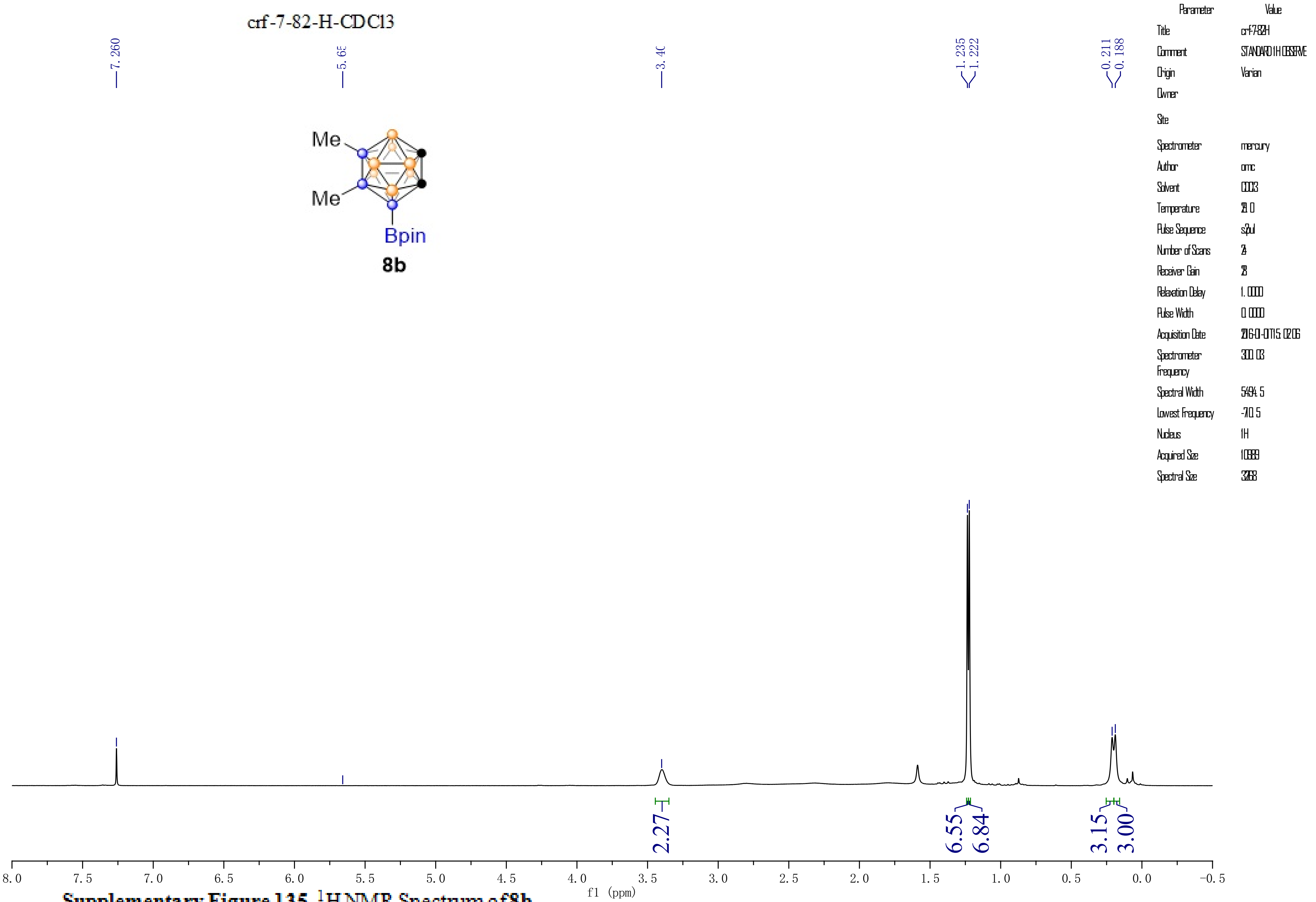

Supplementary Figure 135. ^{1}H NMR Spectrum of **8b**.

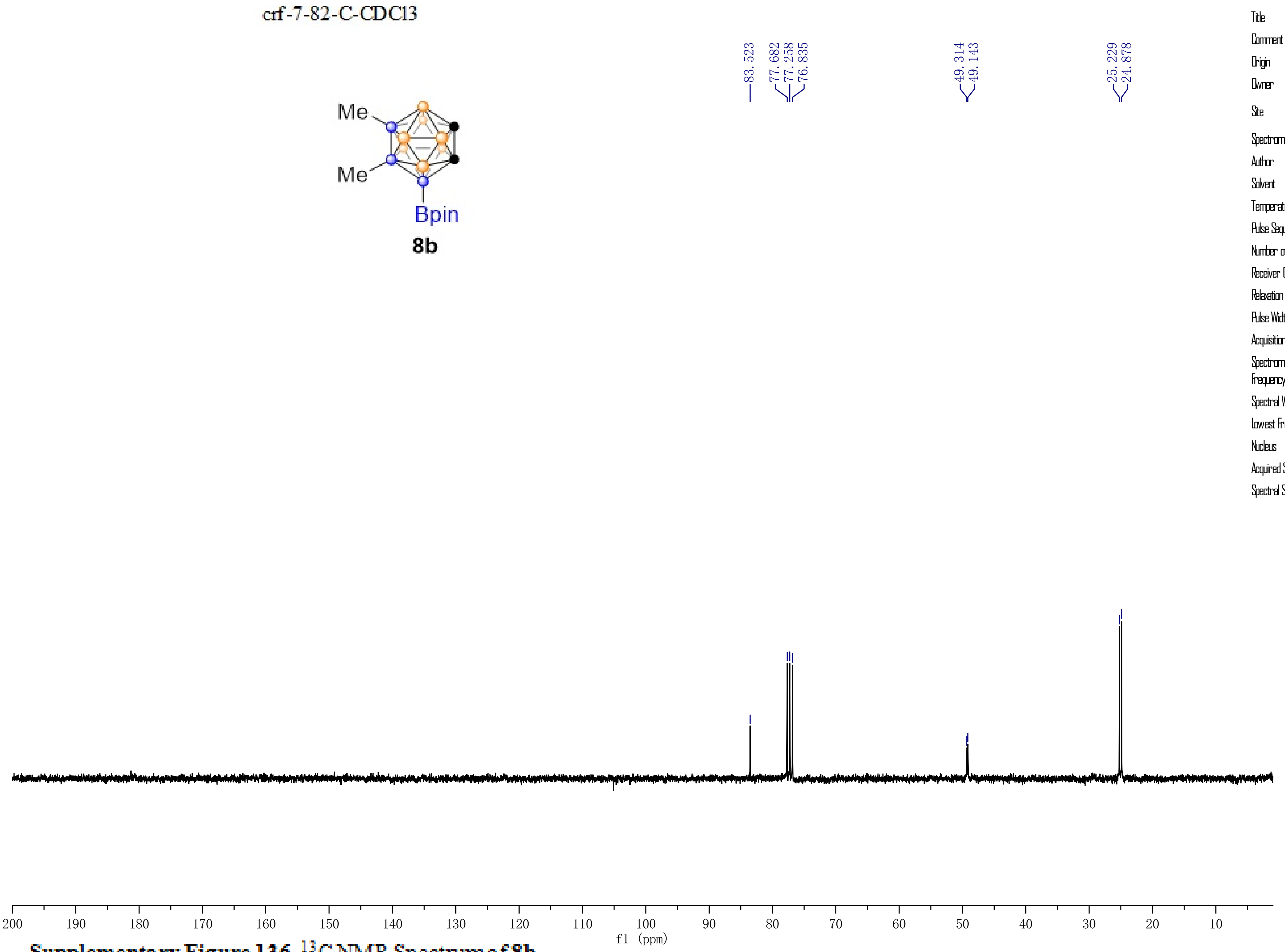

Parameter	Value
Title	crf-7-82C
Comment	13C OBSERVE
Origin	Varian
Owner	
Site	
Spectrometer	mercury
Author	omc
Solvent	CDCl3
Temperature	[illegible]
Pulse Sequence	s2pul
Number of Scans	168
Receiver Gain	38
Relaxation Delay	1.0000
Pulse Width	0.0000
Acquisition Date	2016-01-01T15:03:14
Spectrometer Frequency	75.45
Spectral Width	20491.8
Lowest Frequency	-2815.2
Nucleus	13C
Acquired Size	30738
Spectral Size	65536

Supplementary Figure 136. ^{13}C NMR Spectrum of **8b**.

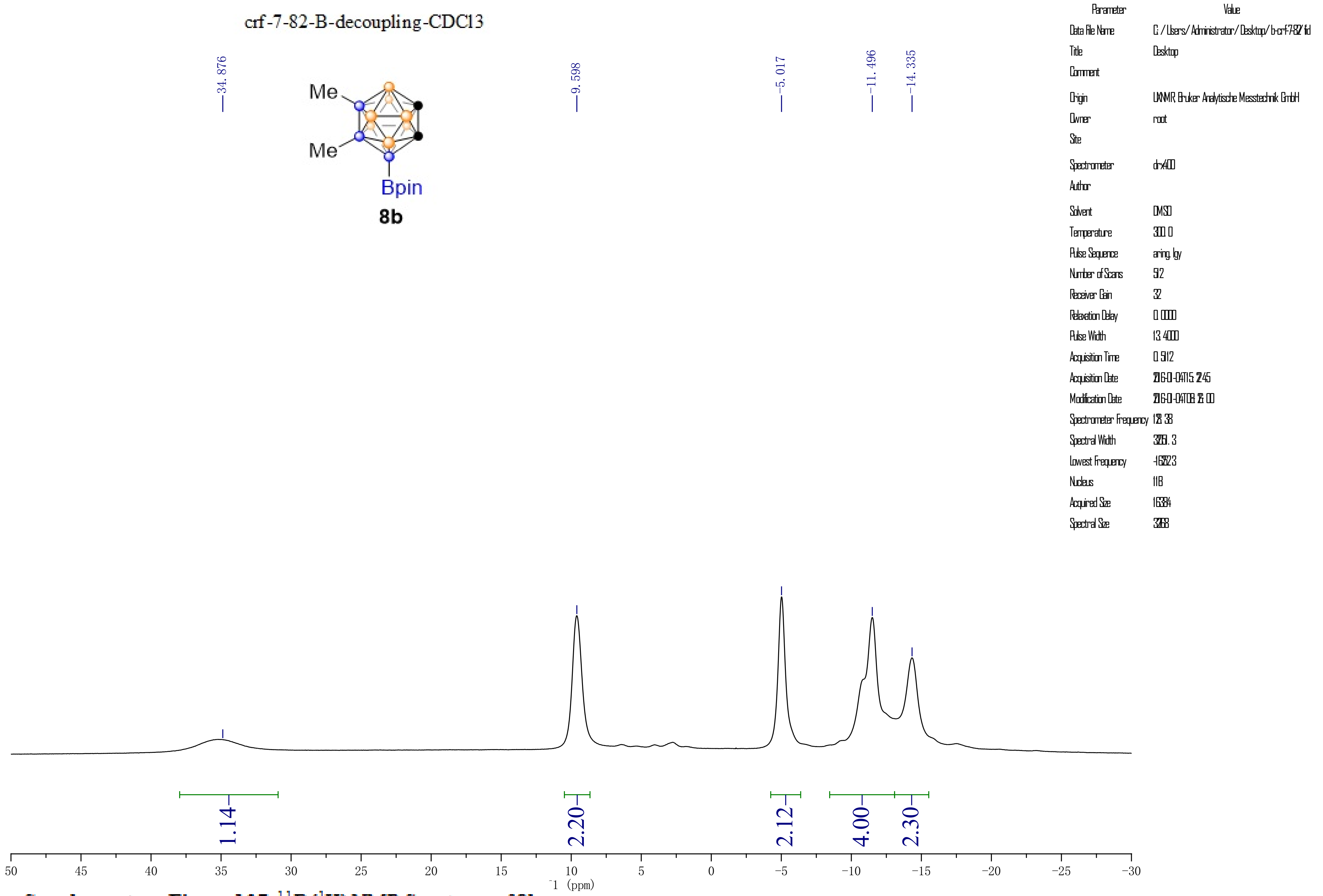

Supplementary Figure 137. $^{11}B\{^{1}H\}$ NMR Spectrum of **8b**.

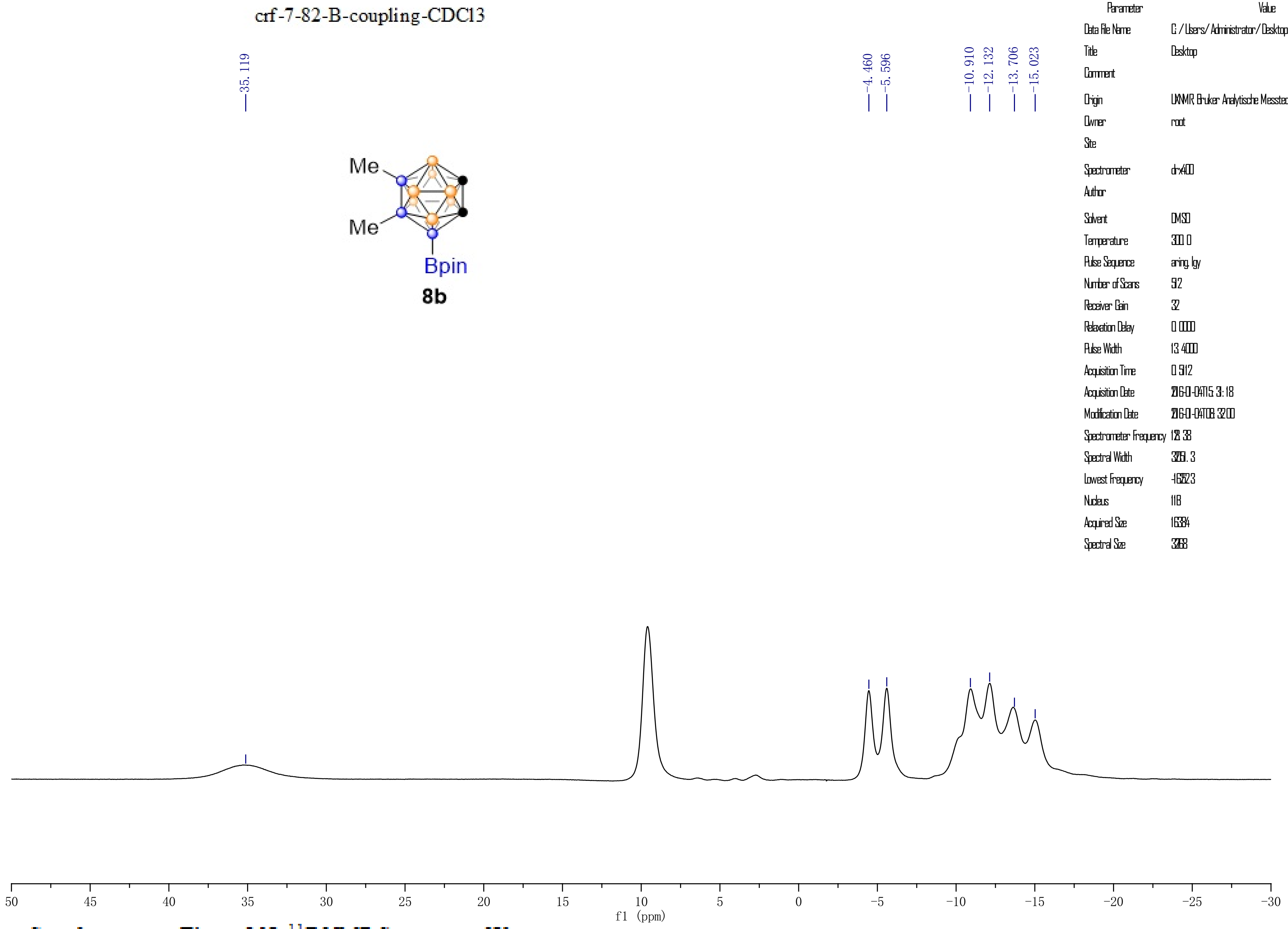

8b

Parameter	Value
Data File Name	C:/Users/Administrator/Desktop/b-crf-7-82coupling/fid
Title	Desktop
Comment	
Origin	UXNMR, Bruker Analytische Messtechnik GmbH
Owner	root
Site	
Spectrometer	drx400
Author	
Solvent	DMSO
Temperature	300.0
Pulse Sequence	aring.lgy
Number of Scans	512
Receiver Gain	32
Relaxation Delay	0.0000
Pulse Width	13.4000
Acquisition Time	0.5112
Acquisition Date	2016-01-04T15:31:18
Modification Date	2016-01-04T08:32:00
Spectrometer Frequency	128.38
Spectral Width	32051.3
Lowest Frequency	-16252.3
Nucleus	11B
Acquired Size	16384
Spectral Size	32768

Supplementary Figure 138. ^{11}B NMR Spectrum of **8b**.

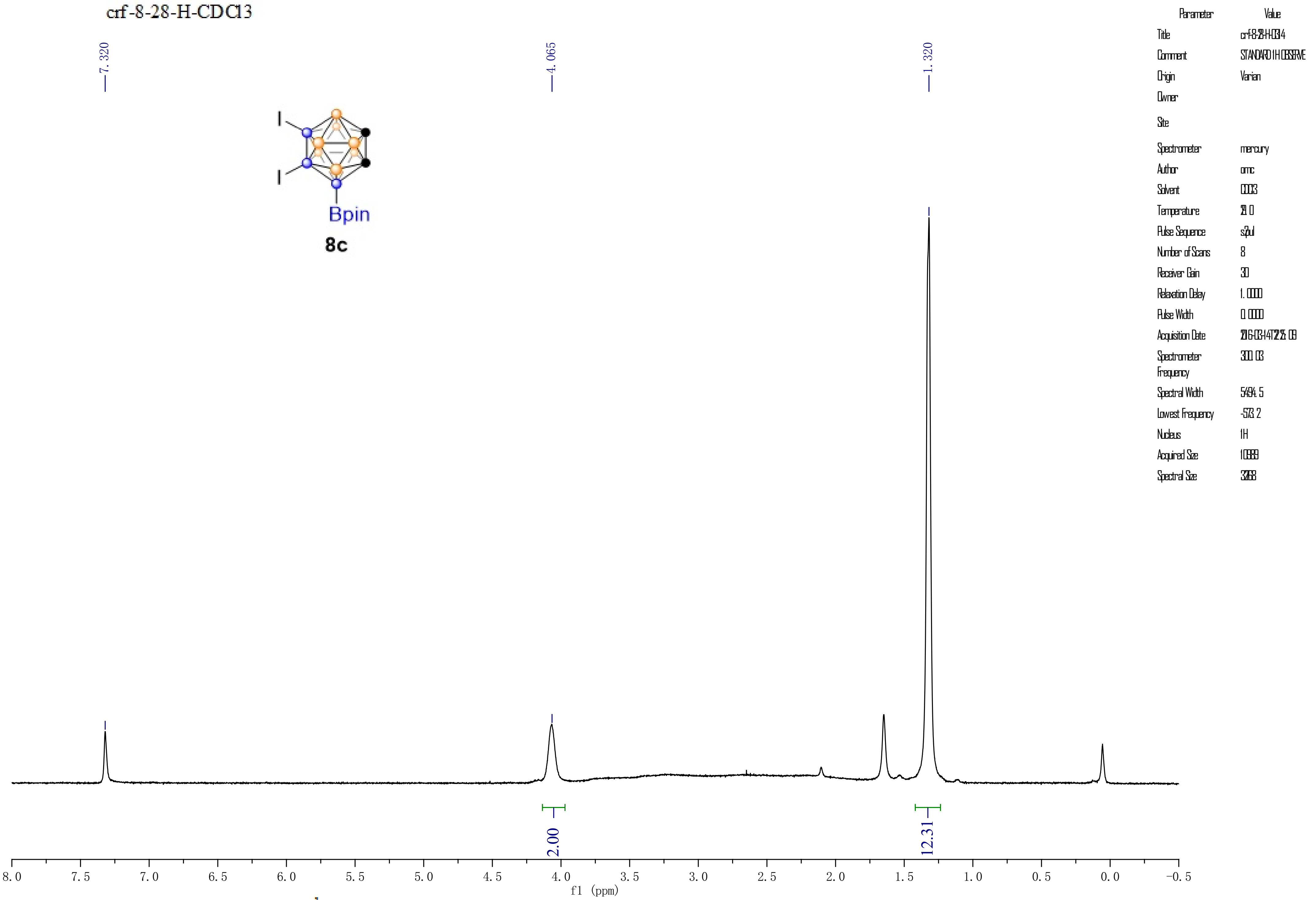

Supplementary Figure 139. ^{1}H NMR Spectrum of **8c**.

crf-8-28-C-CDCl3

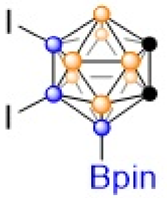

8c

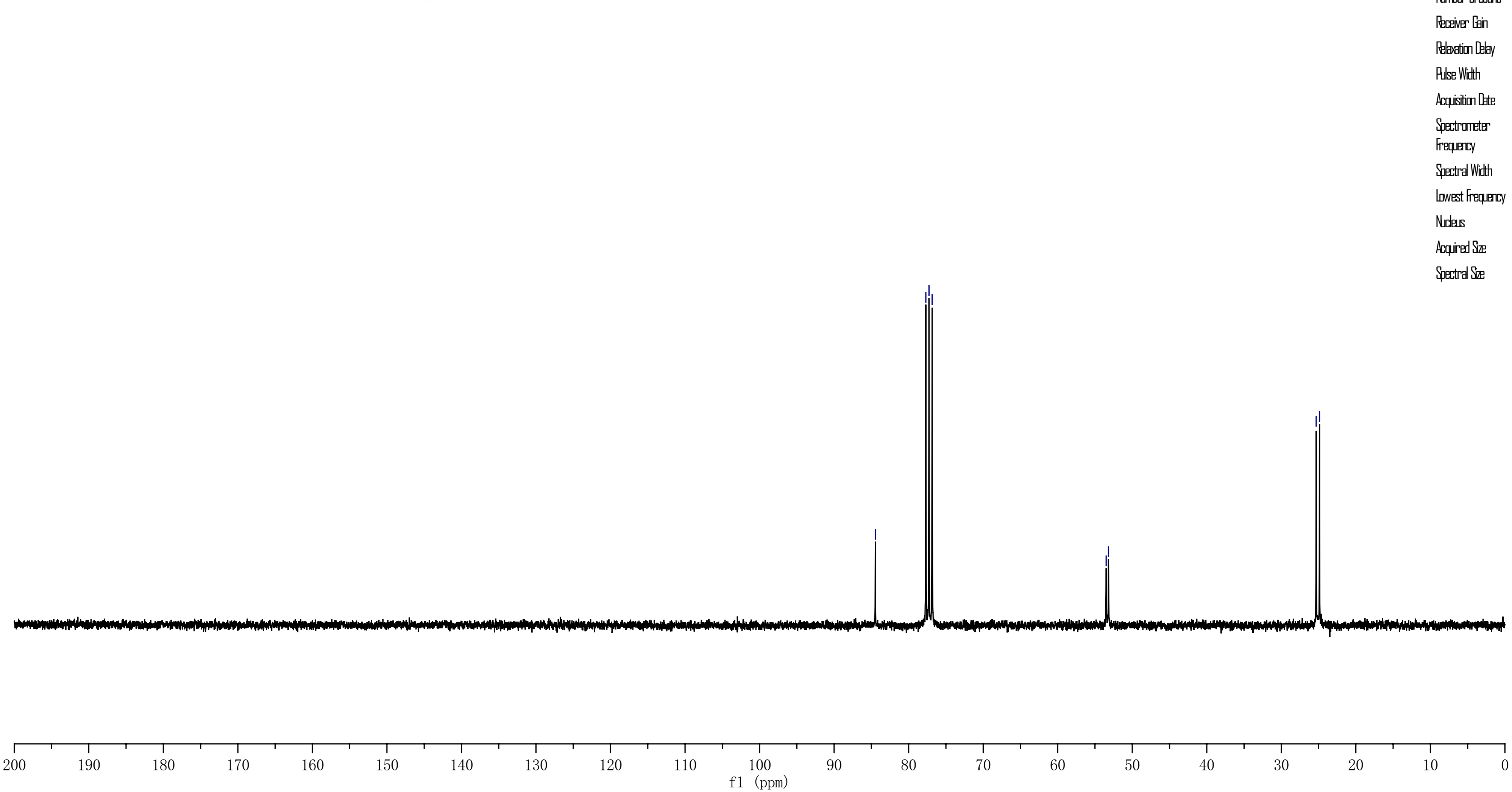

Parameter	Value
Title	crf-8-28-C-0314
Comment	13C OBSERVE
Origin	Varian
Owner	
Site	
Spectrometer	mercury
Author	omc
Solvent	CDCl3
Temperature	21.0
Pulse Sequence	s2pul
Number of Scans	1368
Receiver Gain	34
Relaxation Delay	1.0000
Pulse Width	0.0000
Acquisition Date	2016-03-14T22:30:28
Spectrometer Frequency	75.45
Spectral Width	19011.4
Lowest Frequency	-1735.0
Nucleus	13C
Acquired Size	28517
Spectral Size	65536

Supplementary Figure 140. ^{13}C NMR Spectrum of **8c**.

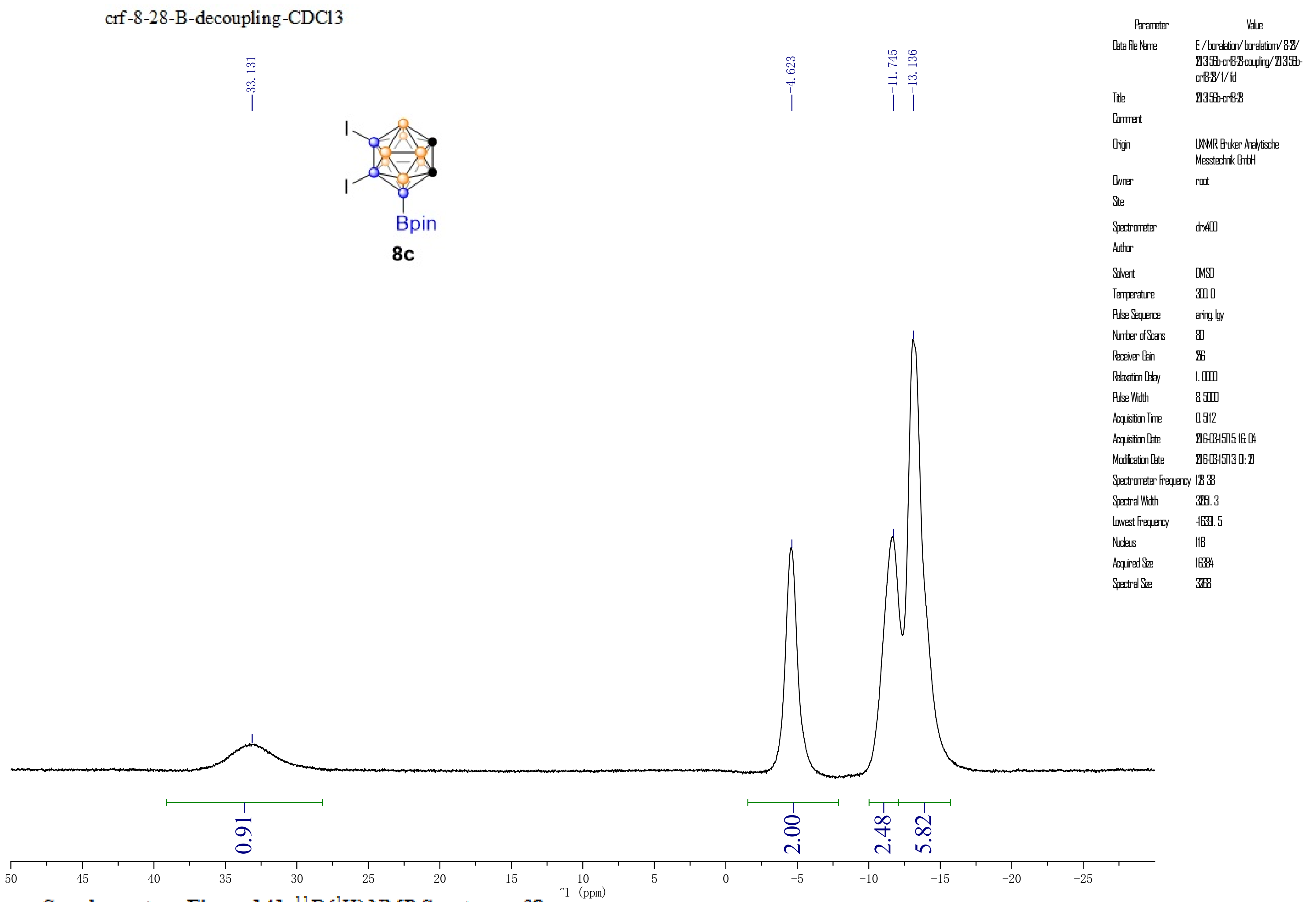

Supplementary Figure 141. $^{11}B\{^{1}H\}$ **NMR Spectrum of 8c.**

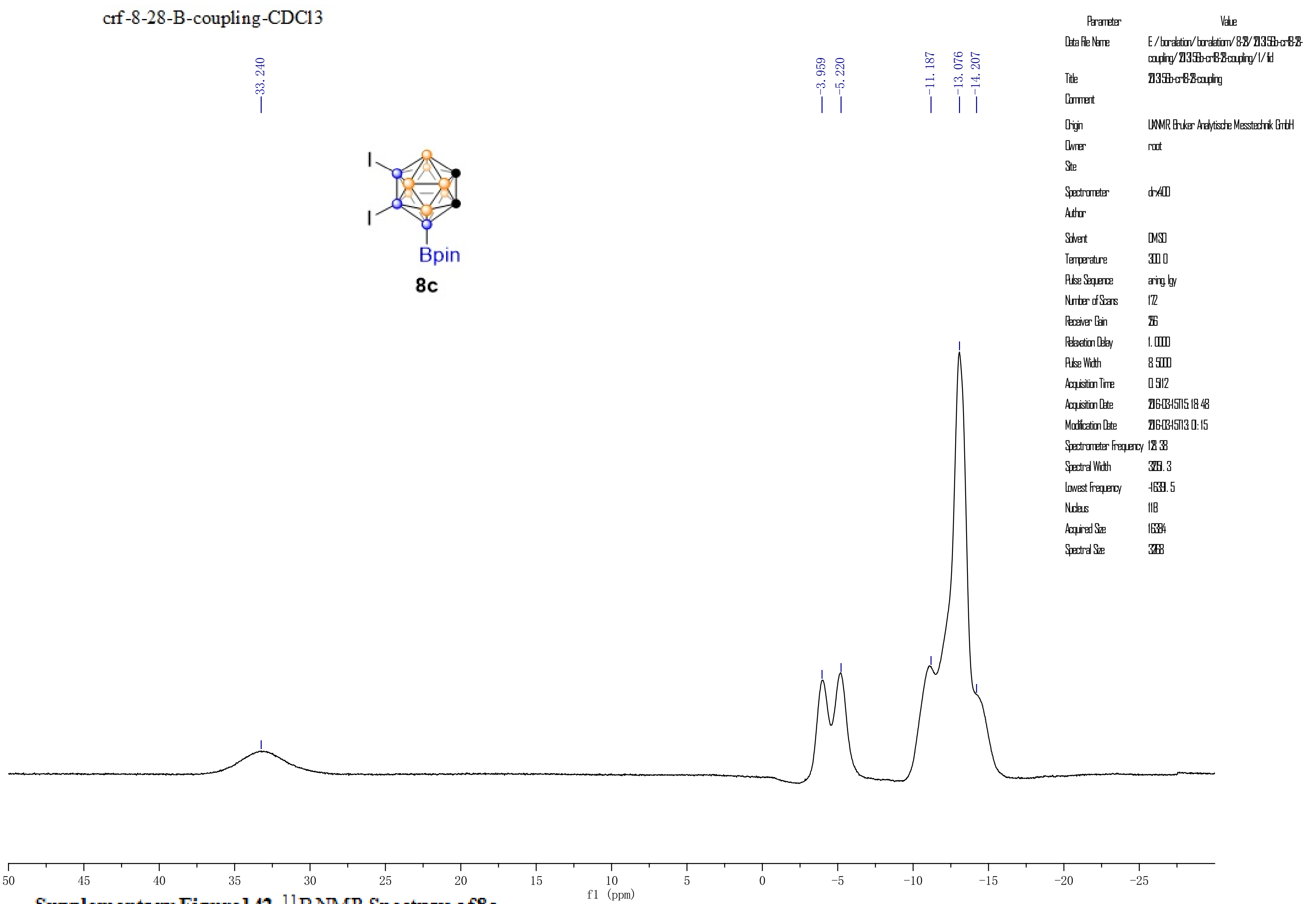

Supplementary Figure142. ^{11}B NMR Spectrum of **8c**.

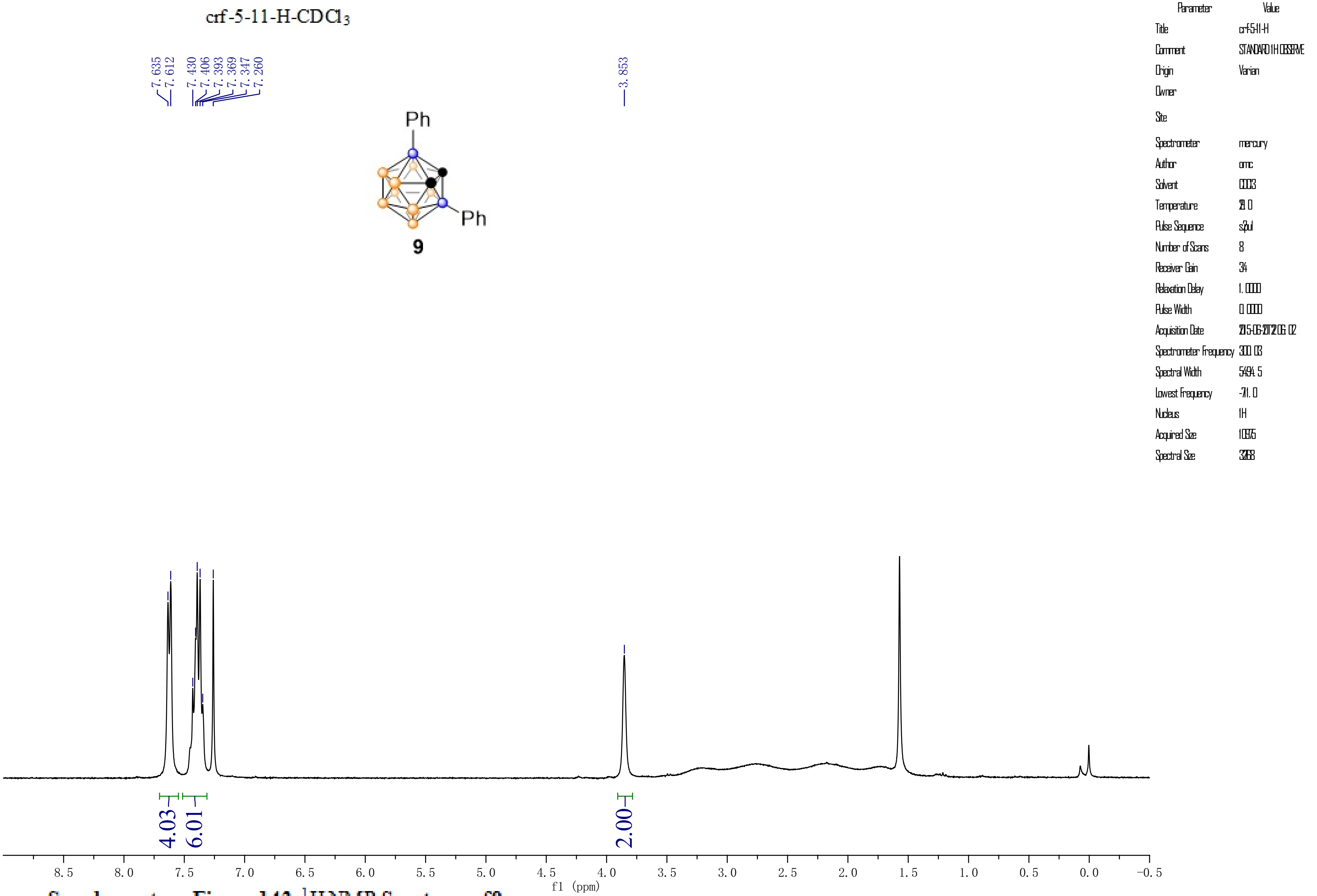

Supplementary Figure 143. ^{1}H NMR Spectrum of **9**.

crf-5-11-C-$CDCl_3$

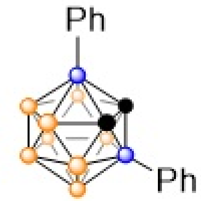

9

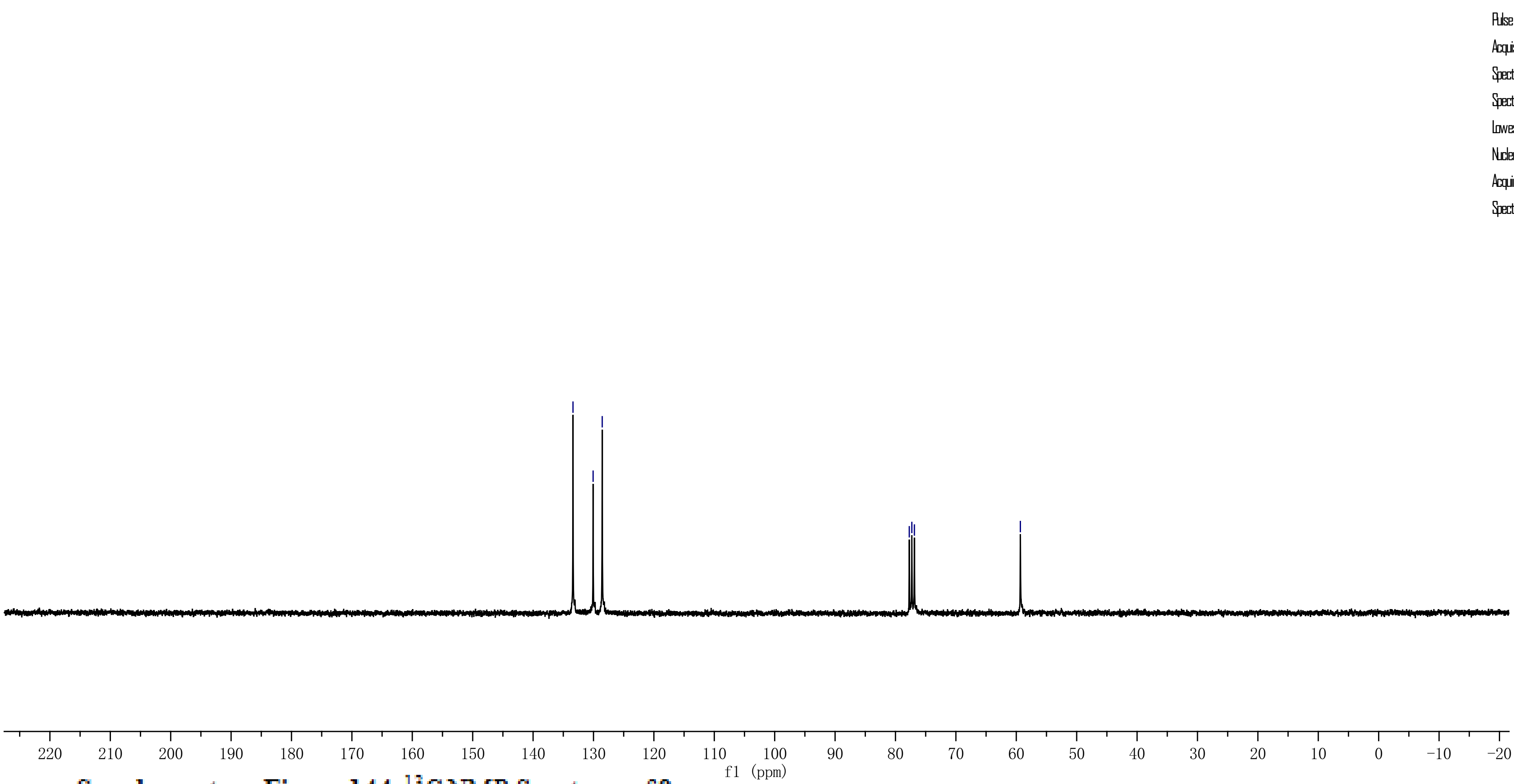

Parameter	Value
Title	crf-5-11-C
Comment	13C OBSERVE
Origin	Varian
Owner	
Site	
Spectrometer	mercury
Author	omc
Solvent	CDCl3
Temperature	21.0
Pulse Sequence	s2pul
Number of Scans	404
Receiver Gain	34
Relaxation Delay	1.0000
Pulse Width	0.0000
Acquisition Date	[illegible]
Spectrometer Frequency	75.45
Spectral Width	18797.0
Lowest Frequency	-1627.8
Nucleus	13C
Acquired Size	[illegible]
Spectral Size	65536

Supplementary Figure 144. ^{13}C NMR Spectrum of **9**.

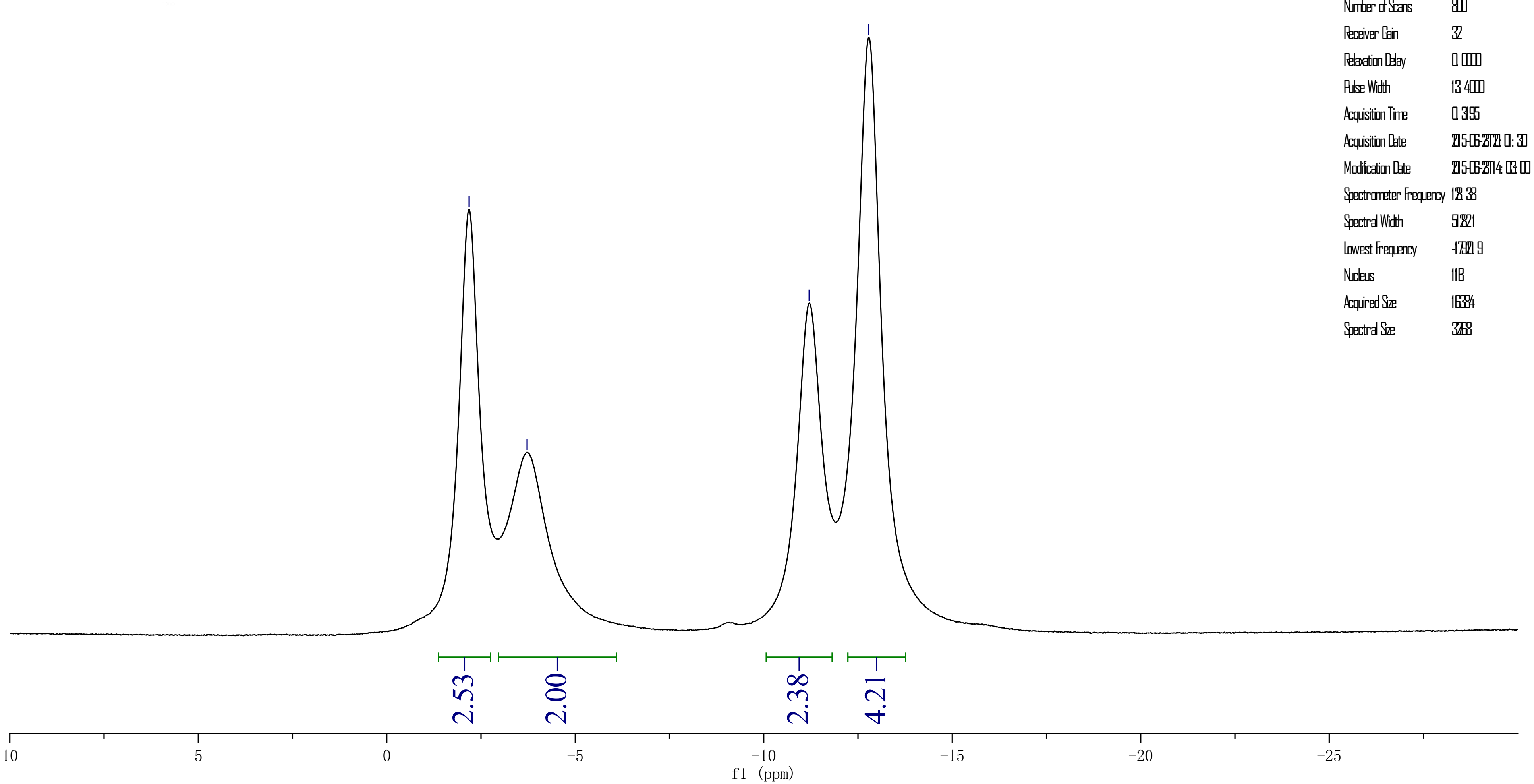

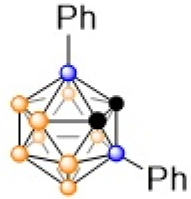

9

Parameter	Value
Data File Name	C:/Users/Administrator/Desktop/work/送样NMR/boralatiom/5-11/b-crf-5-11/fid
Title	5-11
Comment	
Origin	UXNMR, Bruker Analytische Messtechnik GmbH
Owner	root
Site	
Spectrometer	drx400
Author	
Solvent	DMSO
Temperature	300.0
Pulse Sequence	aring.lgy
Number of Scans	800
Receiver Gain	32
Relaxation Delay	0.0000
Pulse Width	13.4000
Acquisition Time	0.3195
Acquisition Date	2015-06-28T21:01:30
Modification Date	2015-06-28T14:03:00
Spectrometer Frequency	128.38
Spectral Width	51282.1
Lowest Frequency	-17920.9
Nucleus	11B
Acquired Size	16384
Spectral Size	32768

Supplementary Figure 145. $^{11}B\{^{1}H\}$ NMR Spectrum of **9**.

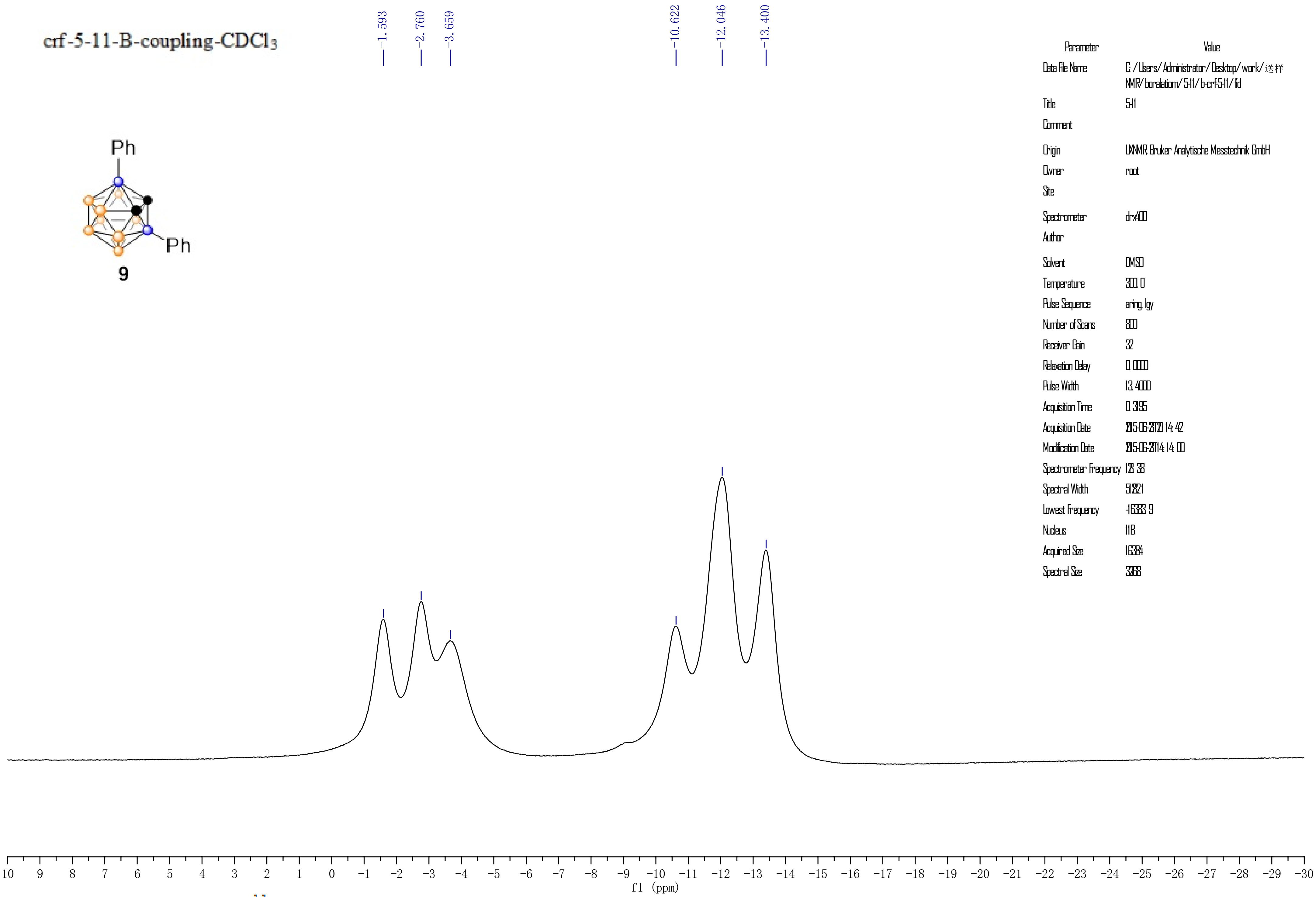

Supplementary Figure 146. ^{11}B NMR Spectrum of **9**.

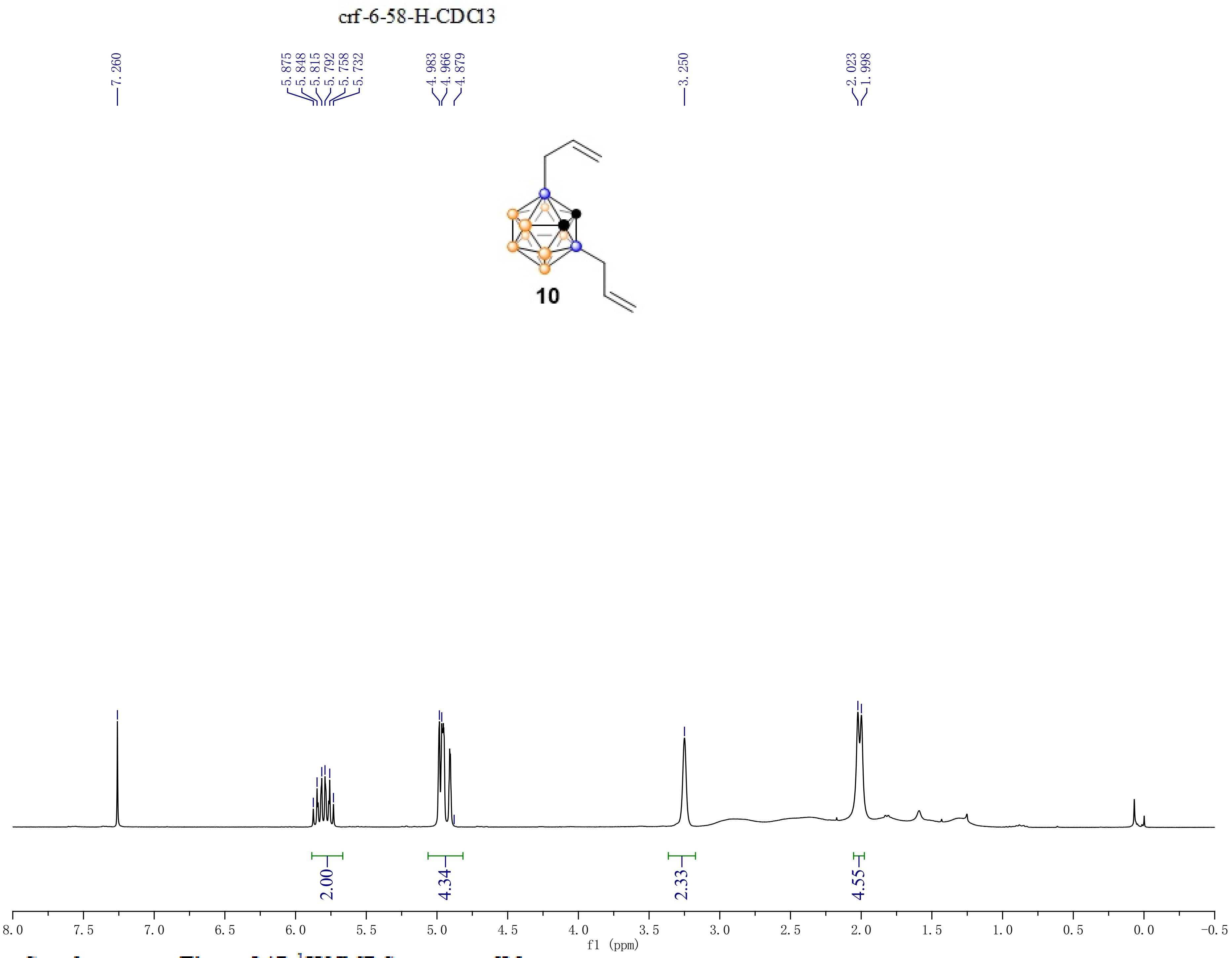

Parameter	Value
Title	crf-6-58-H-CDCl3-1013
Comment	STANDARD 1H OBSERVE
Origin	Varian
Owner	
Site	
Spectrometer	mercury
Author	omc
Solvent	CDCl3
Temperature	21.0
Pulse Sequence	s2pul
Number of Scans	8
Receiver Gain	24
Relaxation Delay	1.0000
Pulse Width	0.0000
Acquisition Date	2015-10-13T16:30:13
Spectrometer Frequency	300.03
Spectral Width	5494.5
Lowest Frequency	-71.0
Nucleus	1H
Acquired Size	10975
Spectral Size	32768

Supplementary Figure 147. ^{1}H NMR Spectrum of **10**.

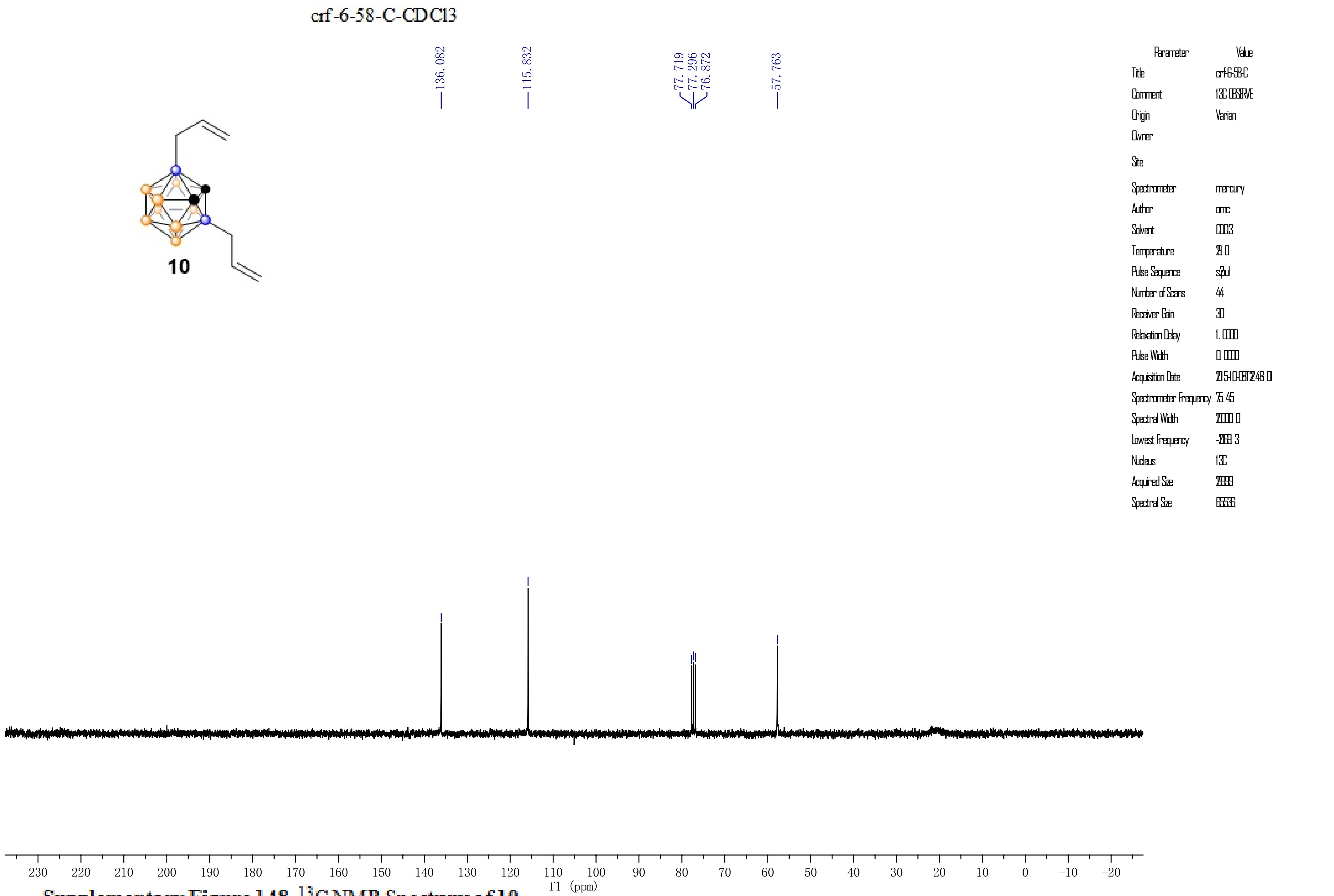

Supplementary Figure 148. ^{13}C NMR Spectrum of **10**.

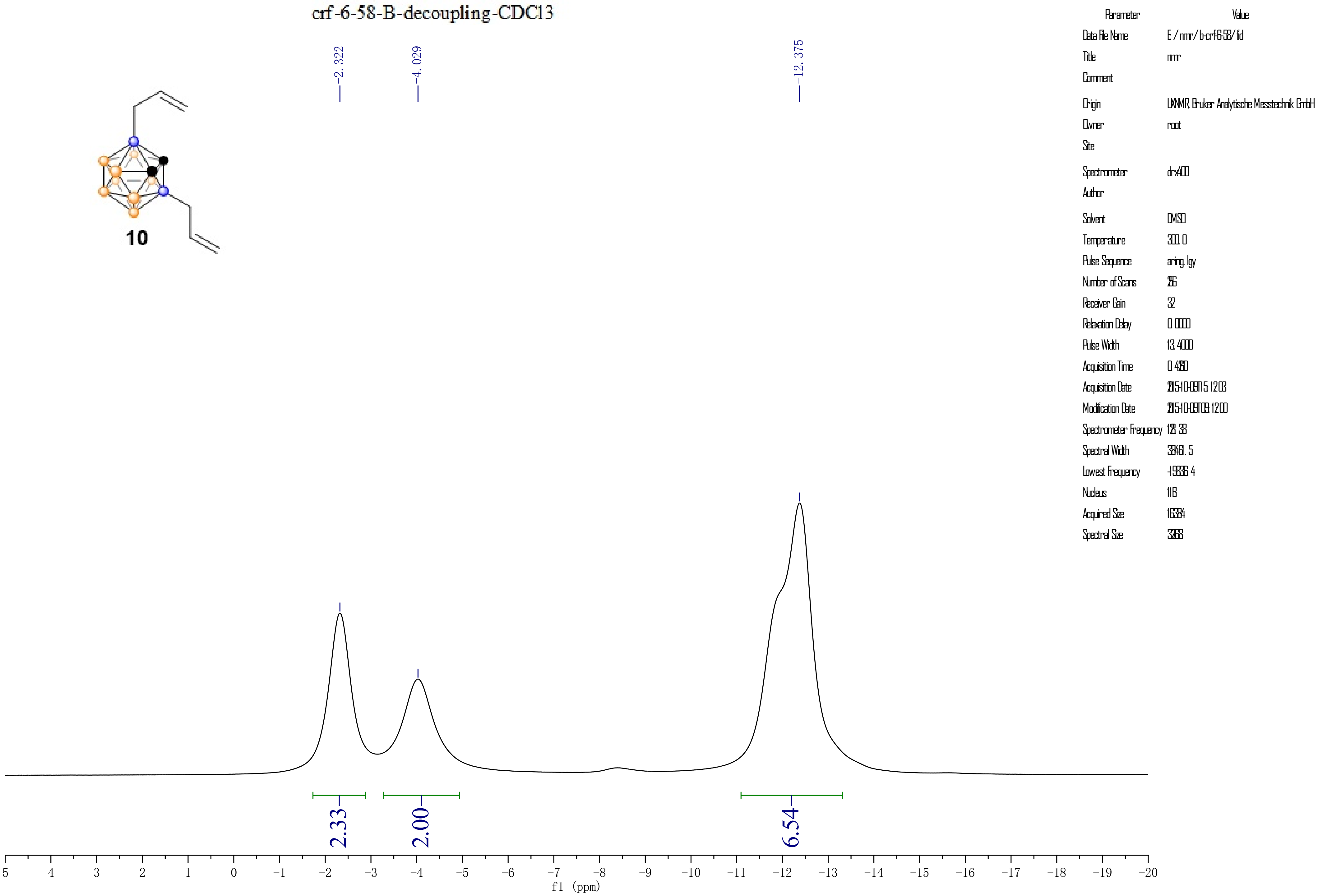

Supplementary Figure 149. $^{11}B\{^{1}H\}$ NMR Spectrum of **10**.

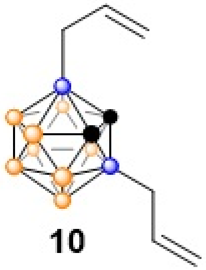

10

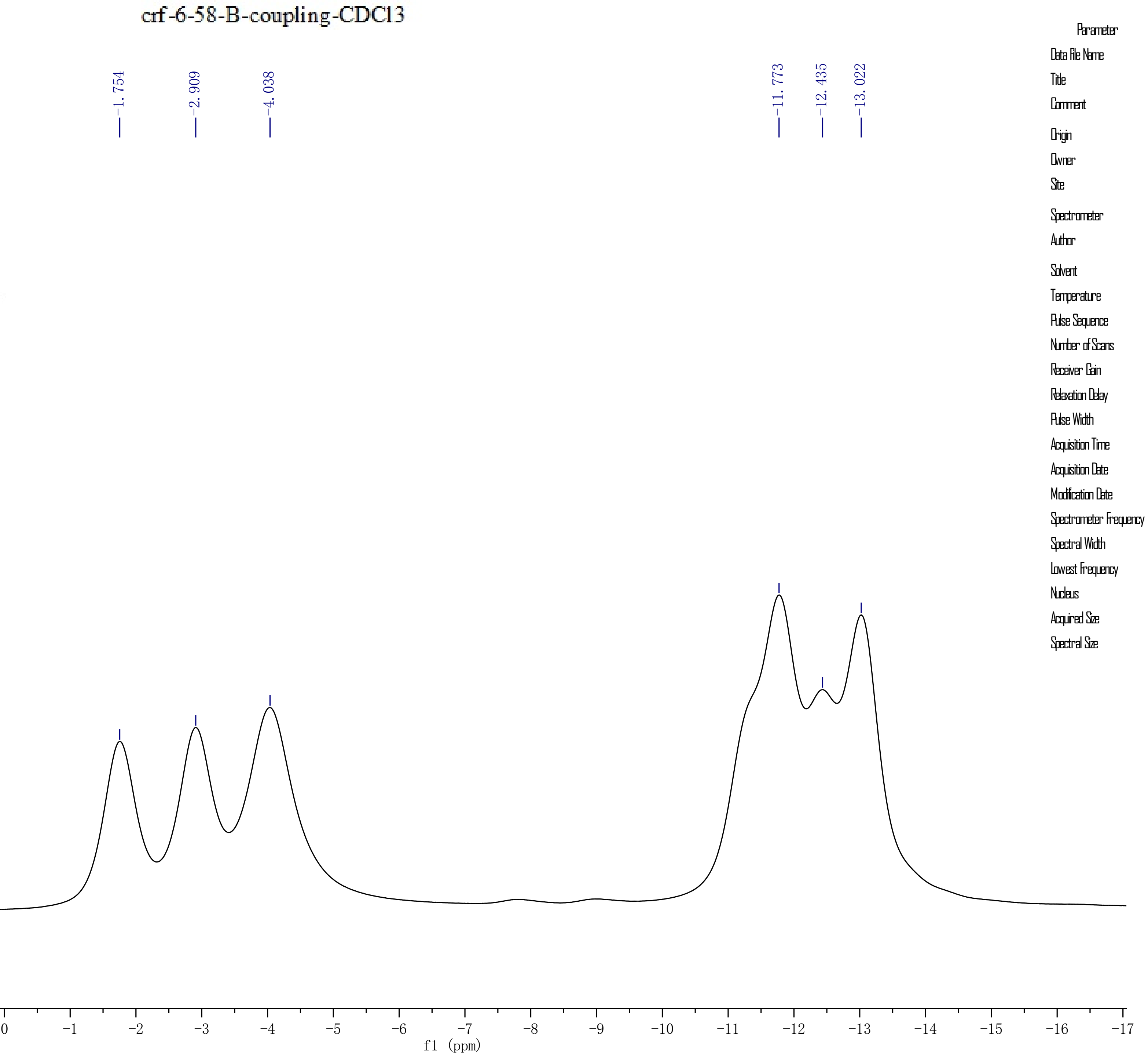

Parameter	Value
Data File Name	E:/nmr/b-crf-6-58-coupling/fid
Title	nmr
Comment	
Origin	UXNMR, Bruker Analytische Messtechnik GmbH
Owner	root
Site	
Spectrometer	drx400
Author	
Solvent	DMSO
Temperature	300.0
Pulse Sequence	aring.lgy
Number of Scans	512
Receiver Gain	32
Relaxation Delay	0.0000
Pulse Width	13.4000
Acquisition Time	0.4260
Acquisition Date	2015-10-09T15:14:27
Modification Date	2015-10-09T09:18:00
Spectrometer Frequency	128.38
Spectral Width	38461.5
Lowest Frequency	-19810.3
Nucleus	11B
Acquired Size	16384
Spectral Size	32768

Supplementary Figure 150. ^{11}B NMR Spectrum of **10**.

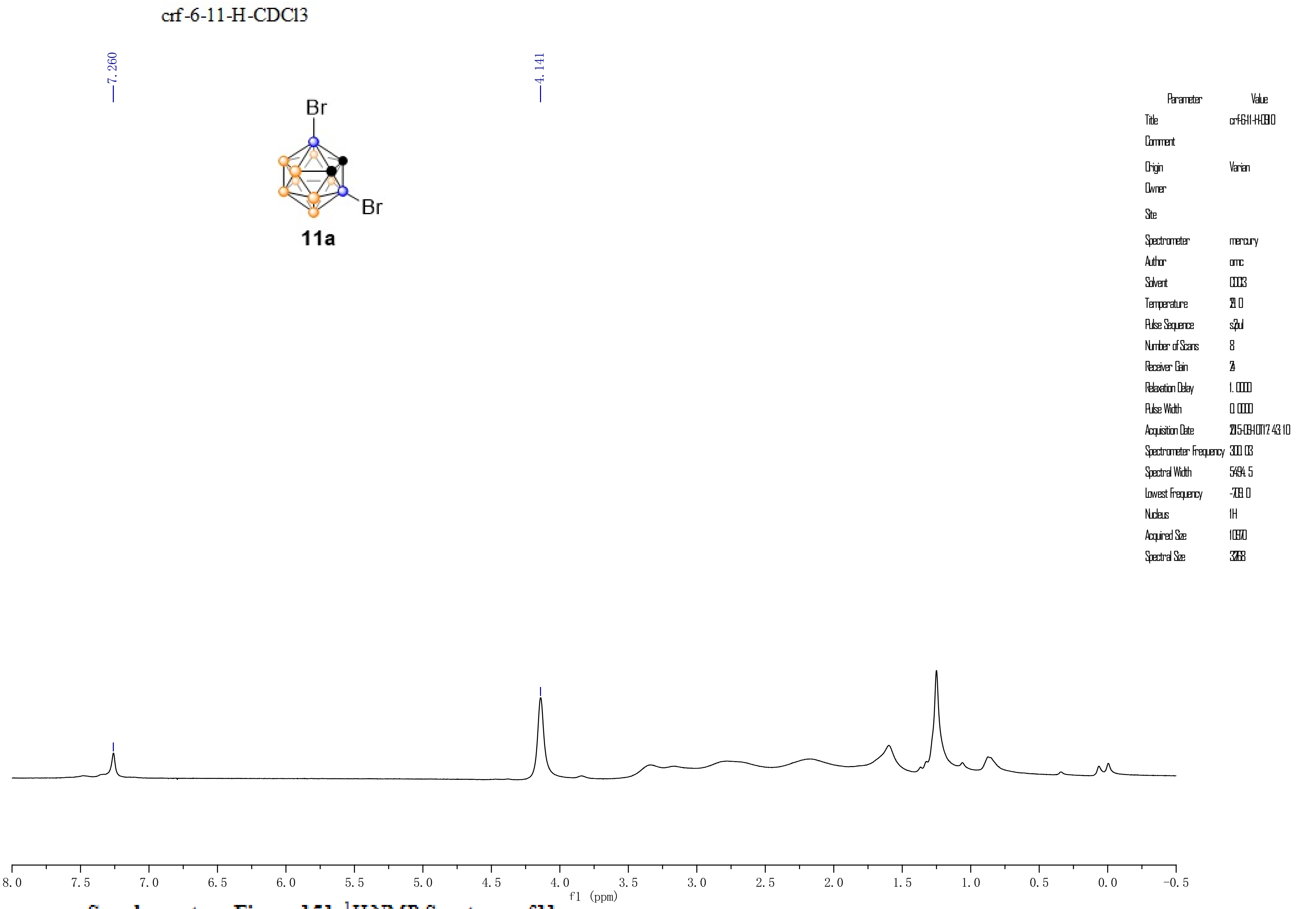

Supplementary Figure 151. ^{1}H NMR Spectrum of **11a**.

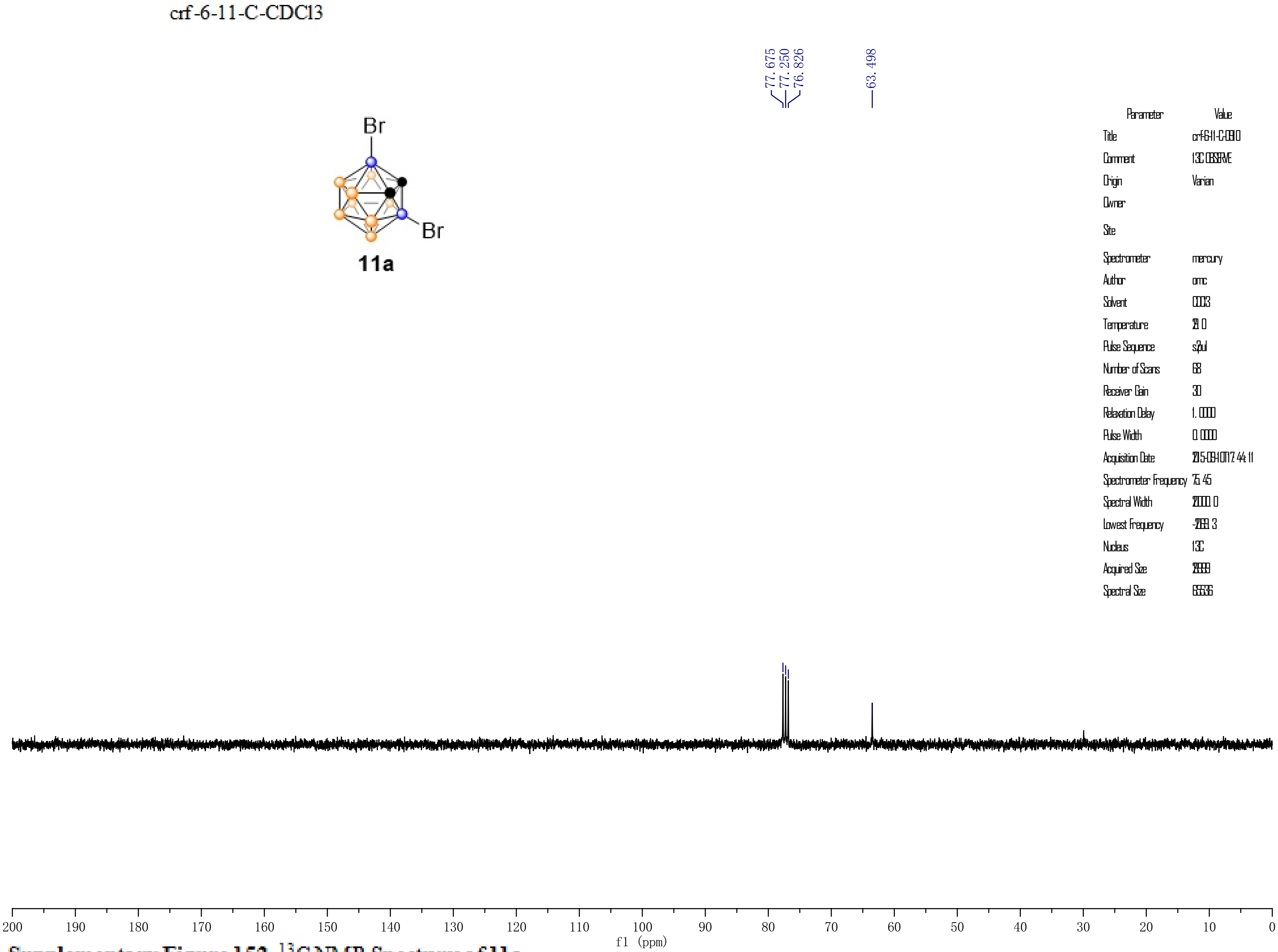

11a

Supplementary Figure 152. ^{13}C NMR Spectrum of **11a**.

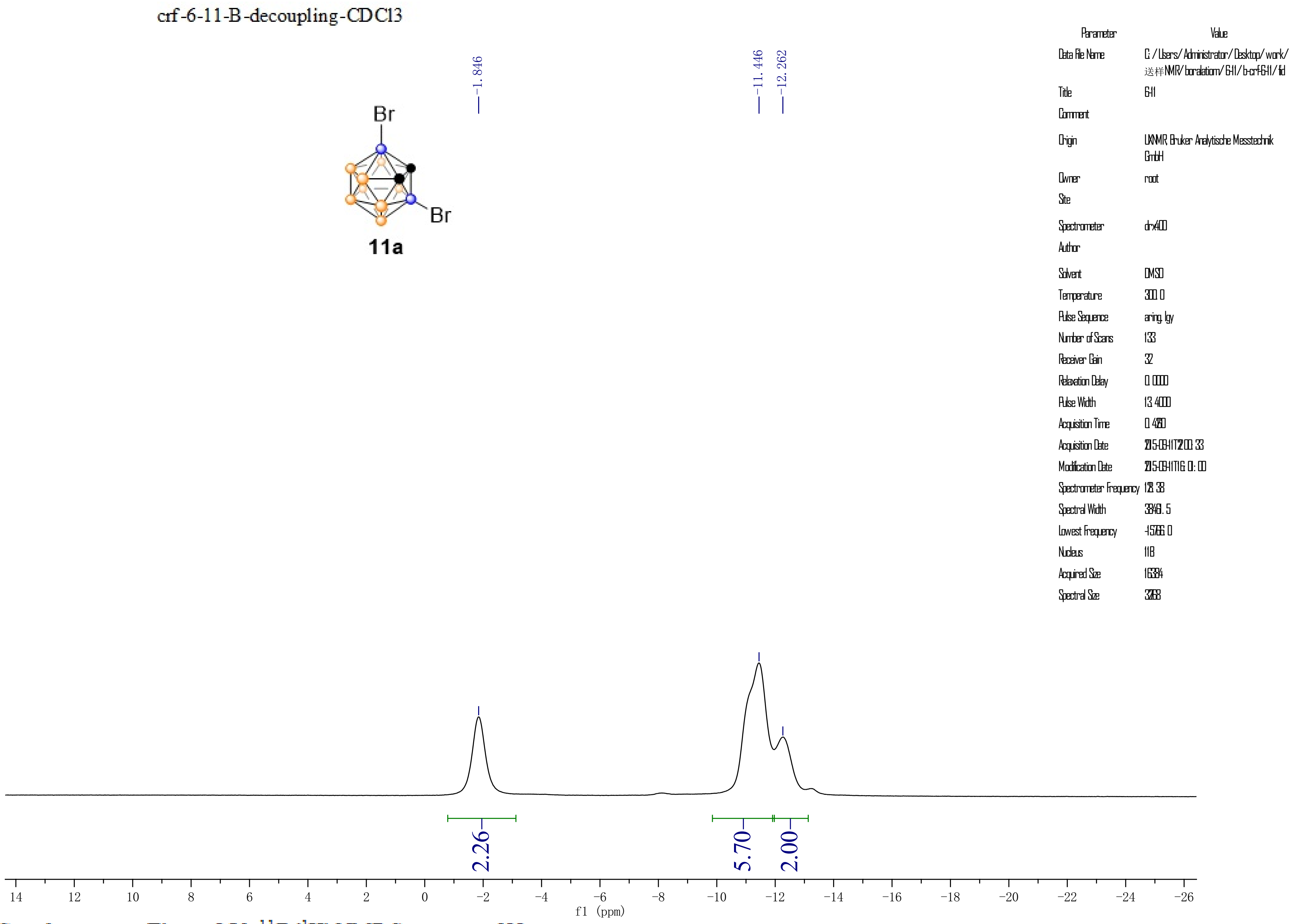

Supplementary Figure 153. $^{11}B\{^{1}H\}$ NMR Spectrum of **11a**.

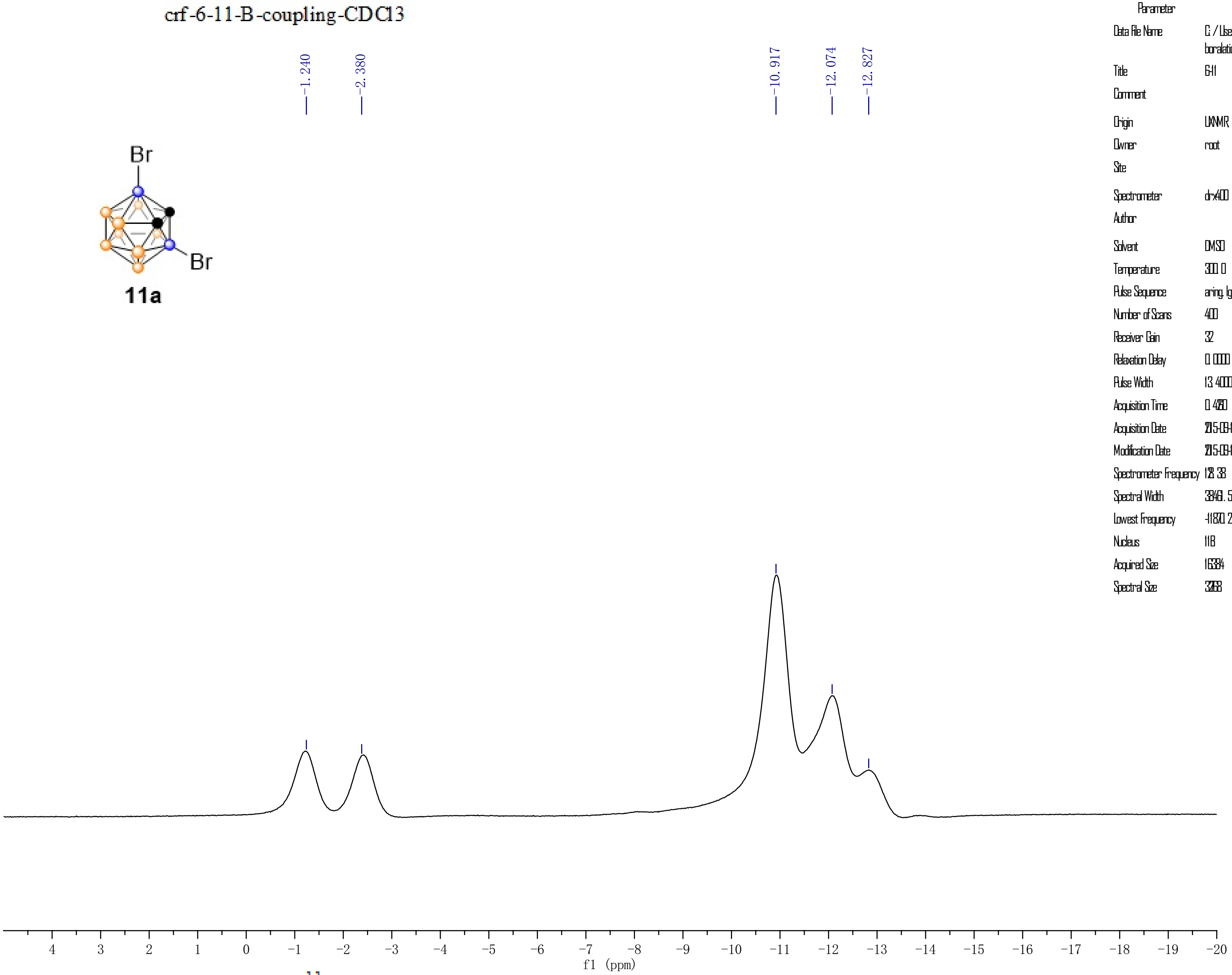

Parameter	Value
Data File Name	C:/Users/Administrator/Desktop/work/送样NMR/boralatiom/6-11/b-crf-6-11-coupling/fid
Title	6-11
Comment	
Origin	UXNMR, Bruker Analytische Messtechnik GmbH
Owner	root
Site	
Spectrometer	drx400
Author	
Solvent	DMSO
Temperature	300.0
Pulse Sequence	aring.lgy
Number of Scans	400
Receiver Gain	32
Relaxation Delay	0.0000
Pulse Width	13.4000
Acquisition Time	0.4260
Acquisition Date	2015-09-11T22:04:03
Modification Date	2015-09-11T16:04:00
Spectrometer Frequency	128.38
Spectral Width	38461.5
Lowest Frequency	-11870.2
Nucleus	11B
Acquired Size	16384
Spectral Size	32768

Supplementary Figure154. ^{11}B NMR Spectrum of **11a**.

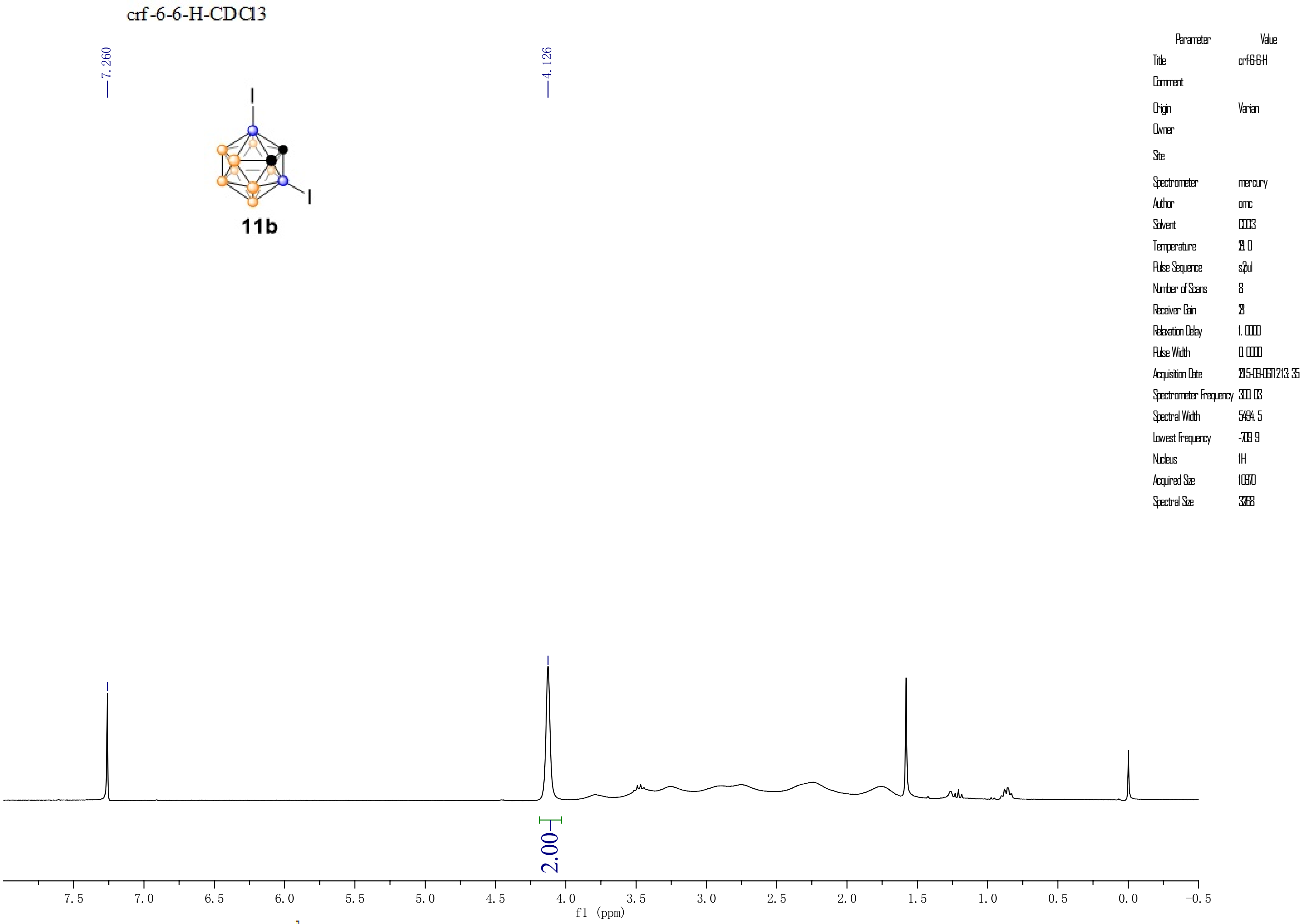

Supplementary Figure 155. ^{1}H NMR Spectrum of **11b**.

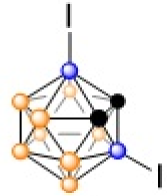

11b

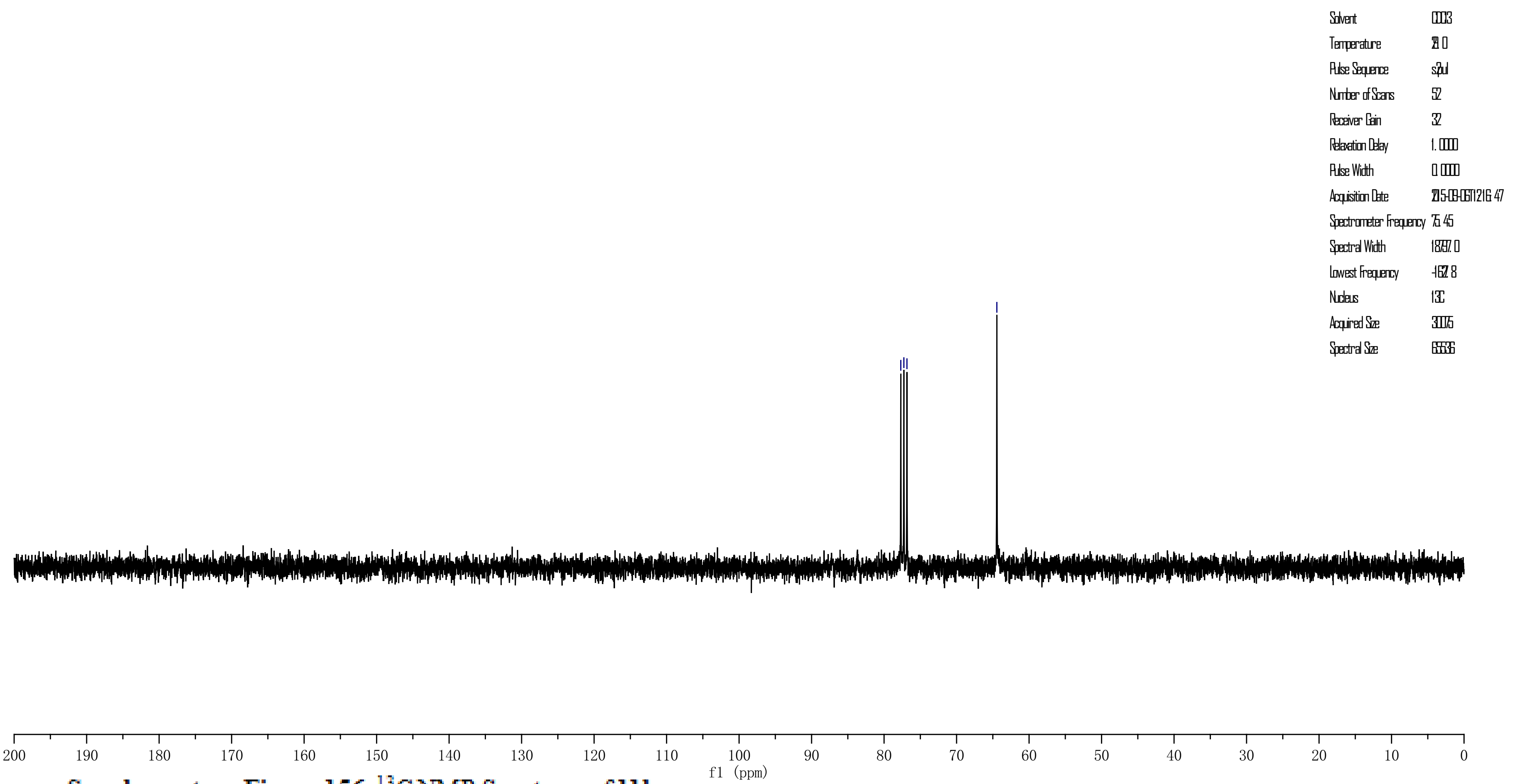

Supplementary Figure 156. ^{13}C NMR Spectrum of **11b**.

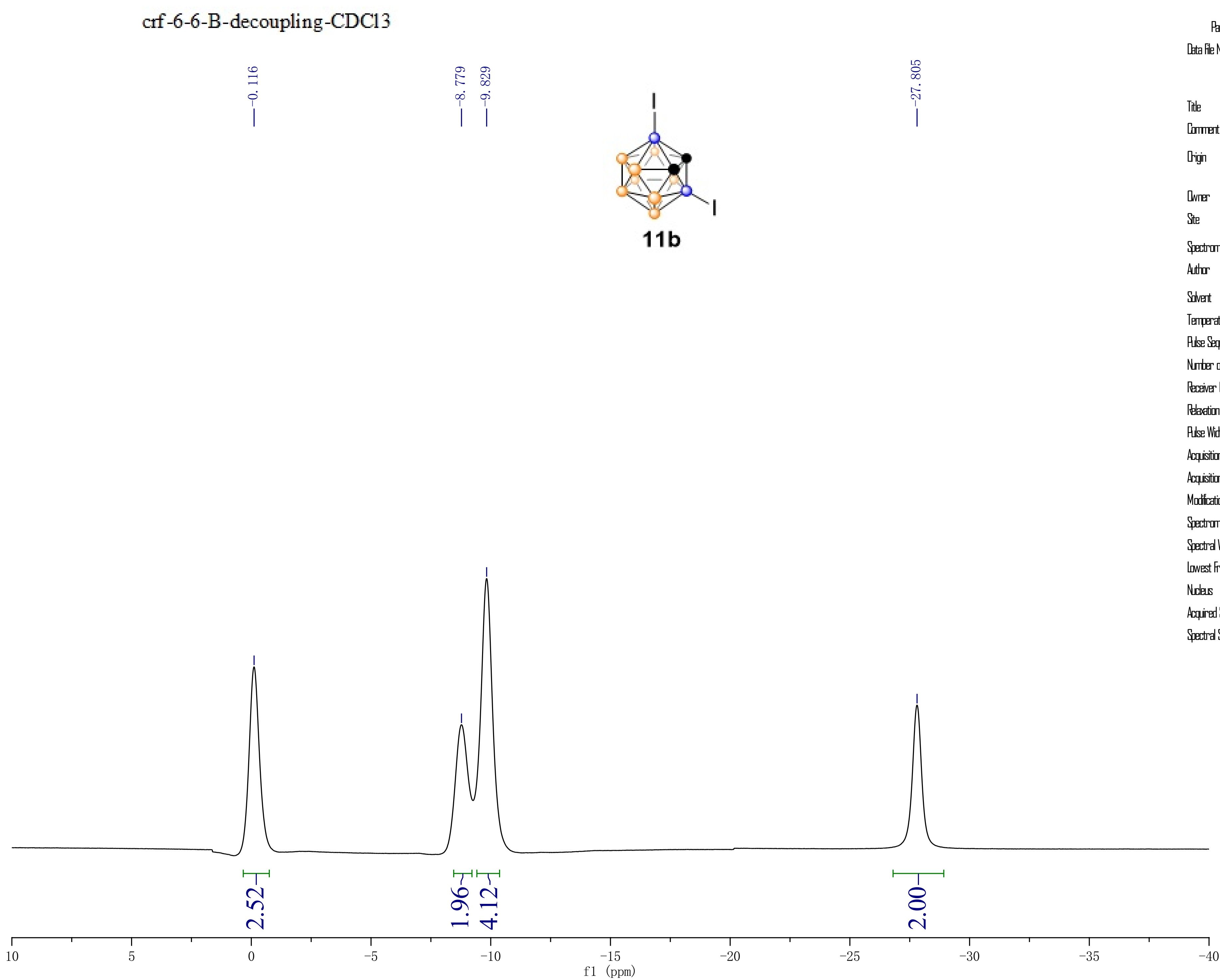

Parameter	Value
Data File Name	C:/Users/Administrator/Desktop/2013156b-crf-6-6/2013156b-crf-6-6/1/fid
Title	2013156b-crf-6-6
Comment	
Origin	UXNMR, Bruker Analytische Messtechnik GmbH
Owner	root
Site	
Spectrometer	drx400
Author	
Solvent	DMSO
Temperature	300.0
Pulse Sequence	aring.lgy
Number of Scans	400
Receiver Gain	32
Relaxation Delay	0.0000
Pulse Width	13.4000
Acquisition Time	0.4260
Acquisition Date	2015-09-07T15:50:56
Modification Date	2015-09-07T10:56:15
Spectrometer Frequency	128.38
Spectral Width	38461.5
Lowest Frequency	-15766.0
Nucleus	11B
Acquired Size	16384
Spectral Size	32768

Supplementary Figure 157. $^{11}B\{^1H\}$ NMR Spectrum of **11b**.

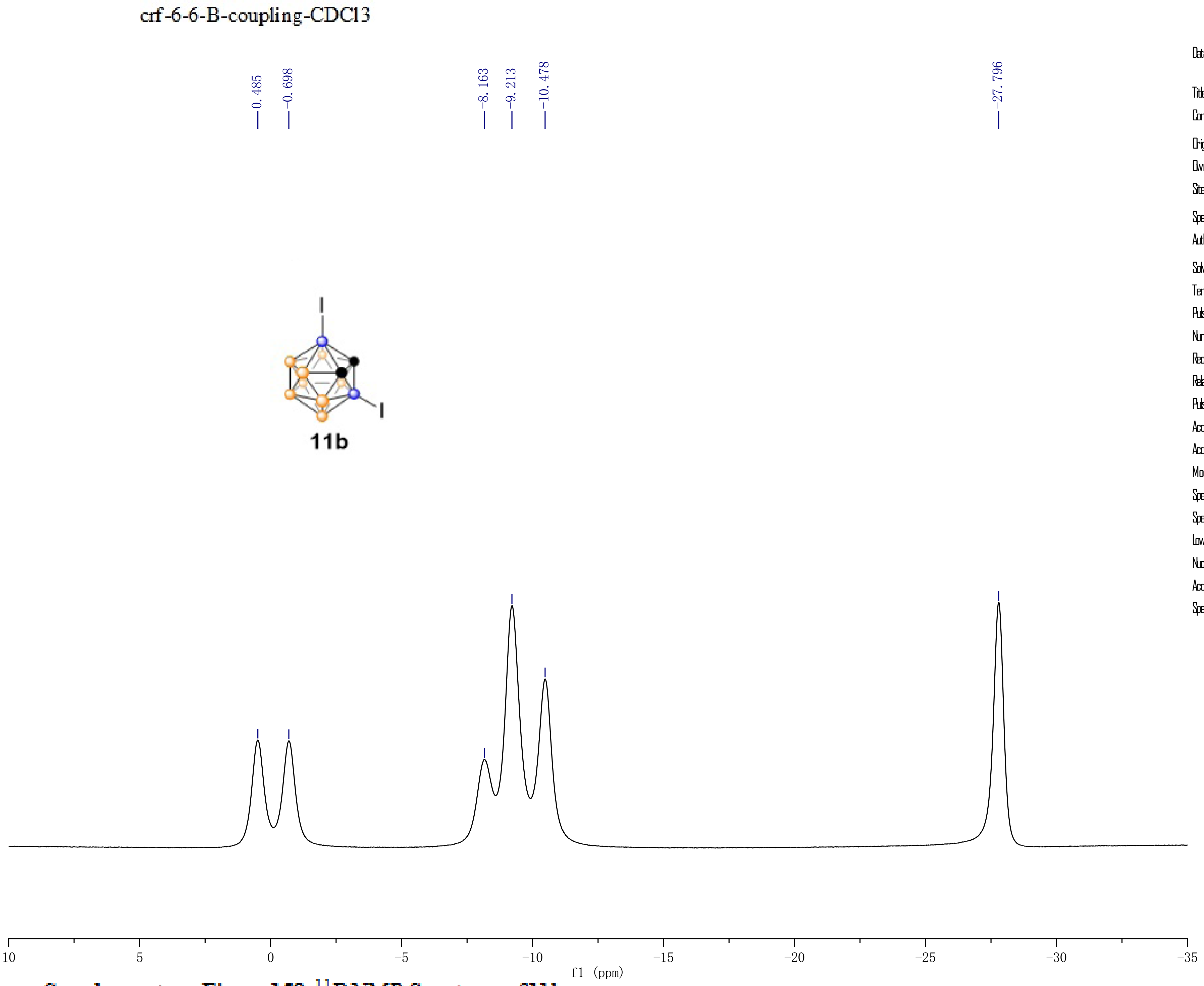

Parameter	Value
Data File Name	C:/Users/Administrator/Desktop/20131566b-crf-6-6/20131566b-crf-6-6-coupling/1/fid
Title	20131566b-crf-6-6-coupling
Comment	
Origin	UXNMR, Bruker Analytische Messtechnik GmbH
Owner	root
Site	
Spectrometer	drx400
Author	
Solvent	DMSO
Temperature	300.0
Pulse Sequence	aring.lgy
Number of Scans	400
Receiver Gain	32
Relaxation Delay	0.0000
Pulse Width	13.4000
Acquisition Time	0.4260
Acquisition Date	2015-09-07T15:54:18
Modification Date	2015-09-07T10:56:10
Spectrometer Frequency	128.38
Spectral Width	38461.5
Lowest Frequency	-11870.2
Nucleus	11B
Acquired Size	16384
Spectral Size	32768

Supplementary Figure158. ^{11}B NMR Spectrum of **11b**.

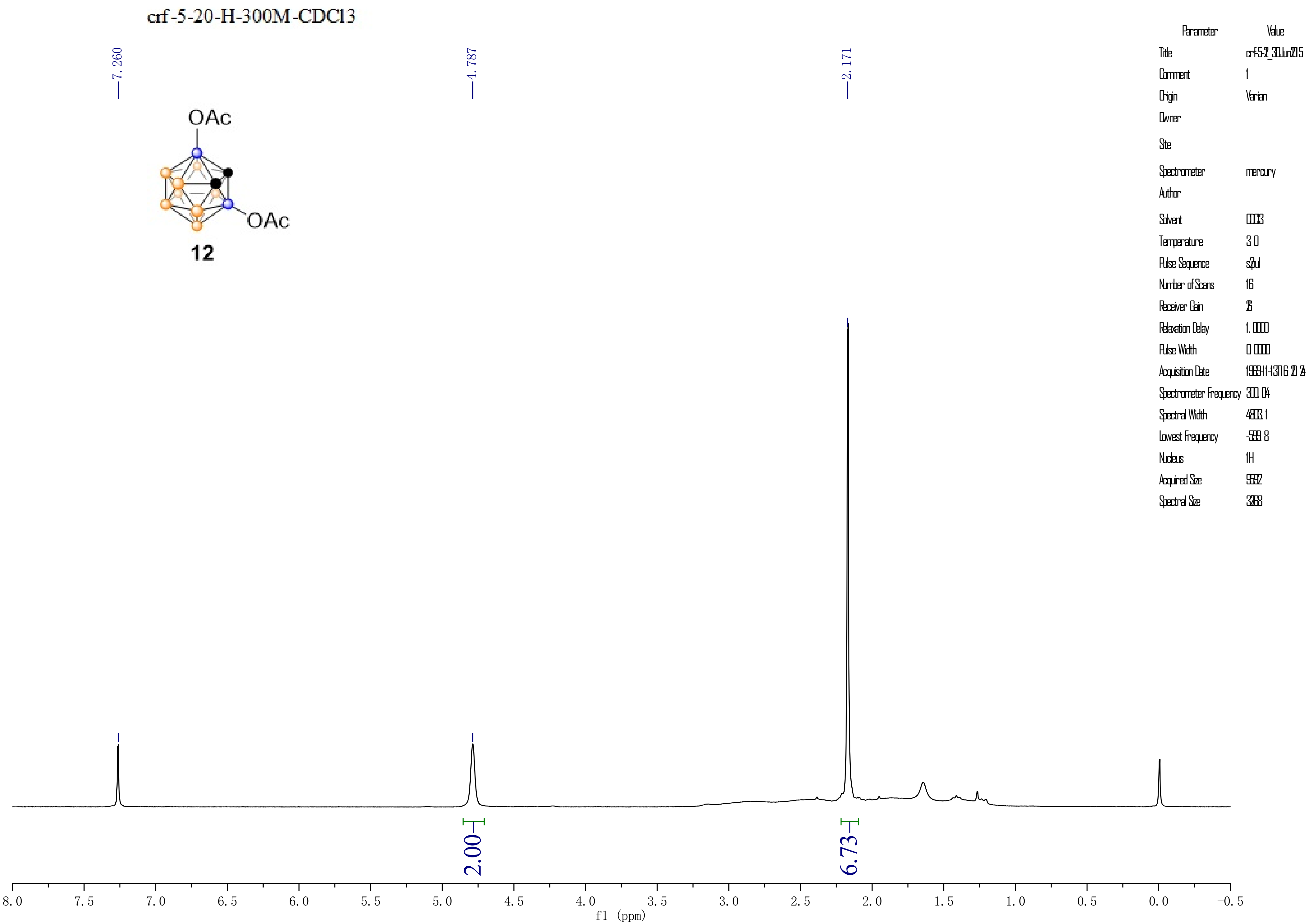

Supplementary Figure 159. ^{1}H NMR Spectrum of **12**.

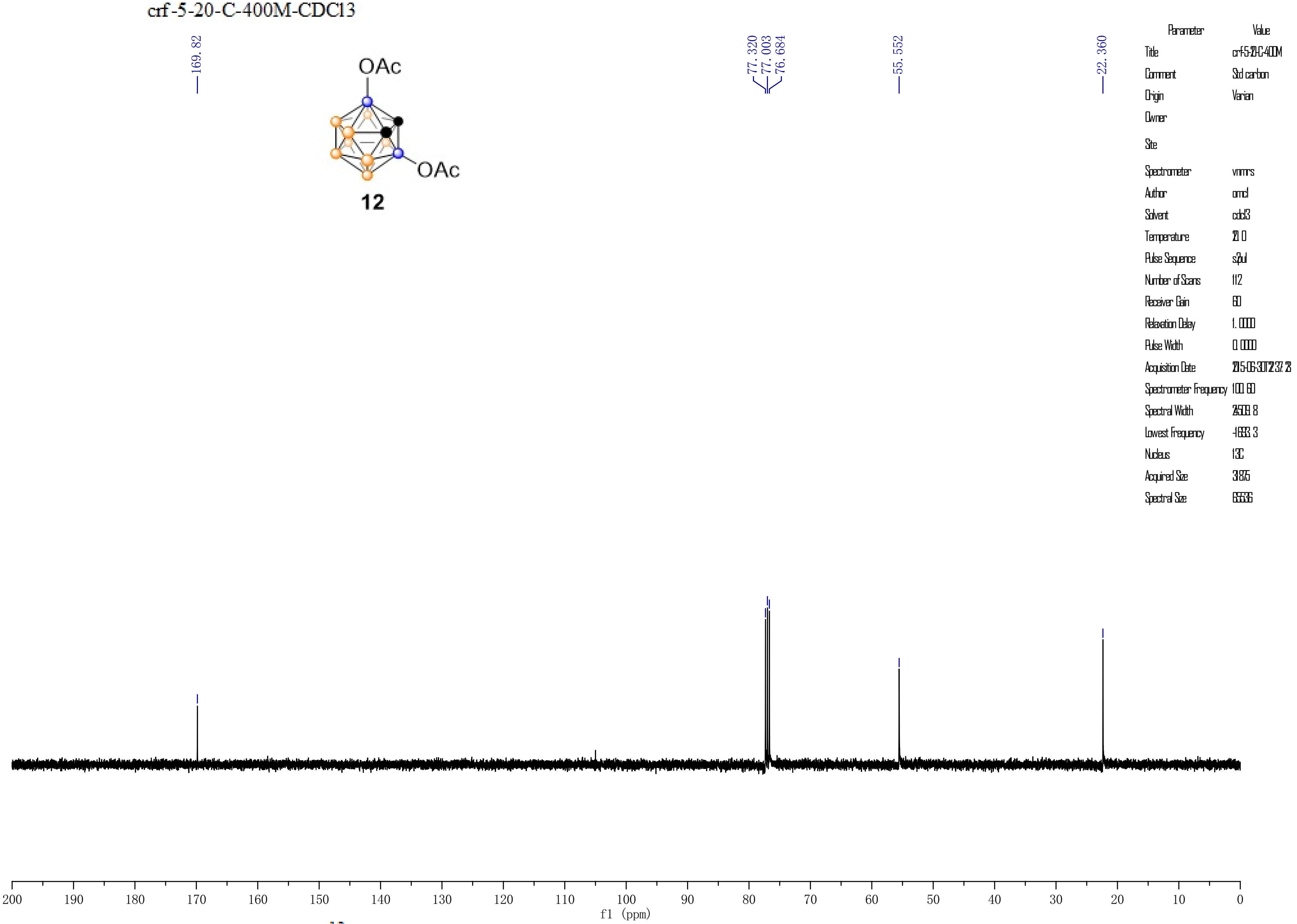

Supplementary Figure 160. ^{13}C NMR Spectrum of **12**.

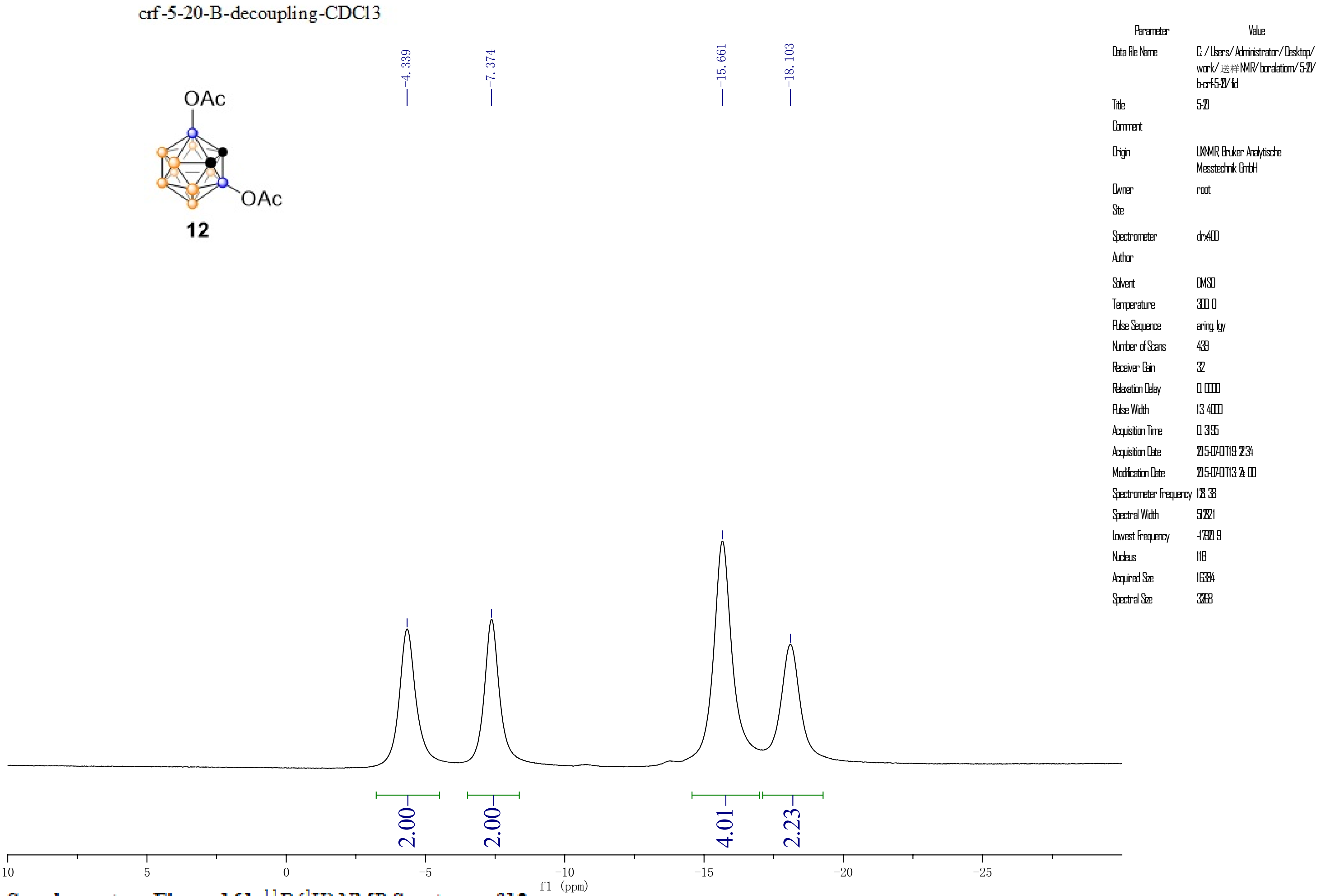

Supplementary Figure 161. $^{11}B\{^1H\}$ NMR Spectrum of **12**.

crf-5-20-B-coupling-CDCl3

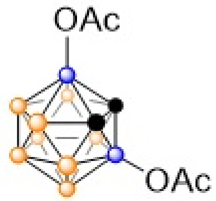

12

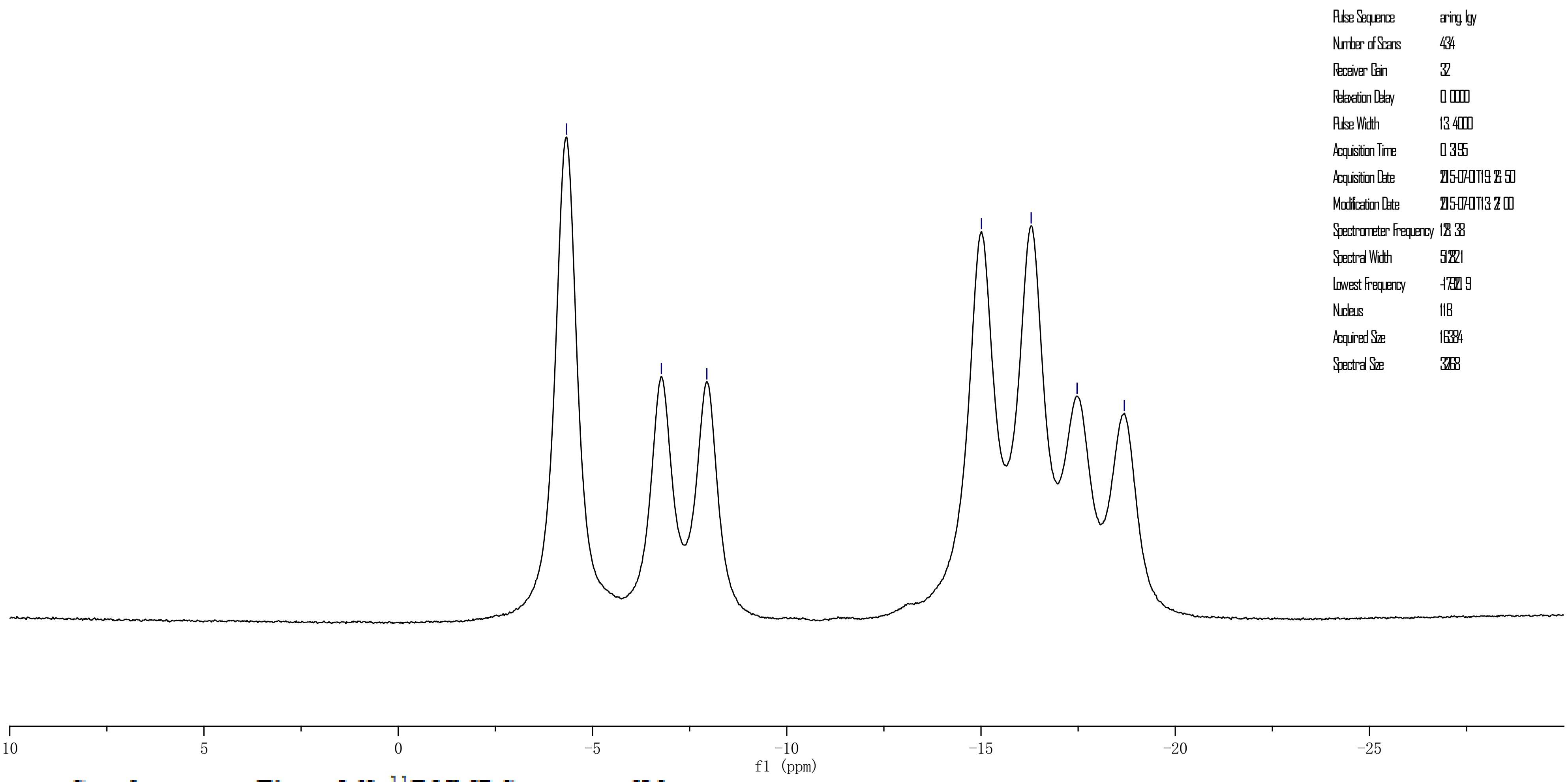

Parameter	Value
Data File Name	C:/Users/Administrator/Desktop/work/送样NMR/boralatiom/5-20/b-crf-5-20-coupling/fid
Title	5-20
Comment	
Origin	UXNMR, Bruker Analytische Messtechnik GmbH
Owner	root
Site	
Spectrometer	drx400
Author	
Solvent	DMSO
Temperature	300.0
Pulse Sequence	aring.lgy
Number of Scans	434
Receiver Gain	32
Relaxation Delay	0.0000
Pulse Width	13.4000
Acquisition Time	0.3195
Acquisition Date	2015-07-01T19:26:50
Modification Date	2015-07-01T13:27:00
Spectrometer Frequency	128.38
Spectral Width	51282.1
Lowest Frequency	-17920.9
Nucleus	11B
Acquired Size	16384
Spectral Size	32768

Supplementary Figure162. ^{11}B NMR Spectrum of **12**.

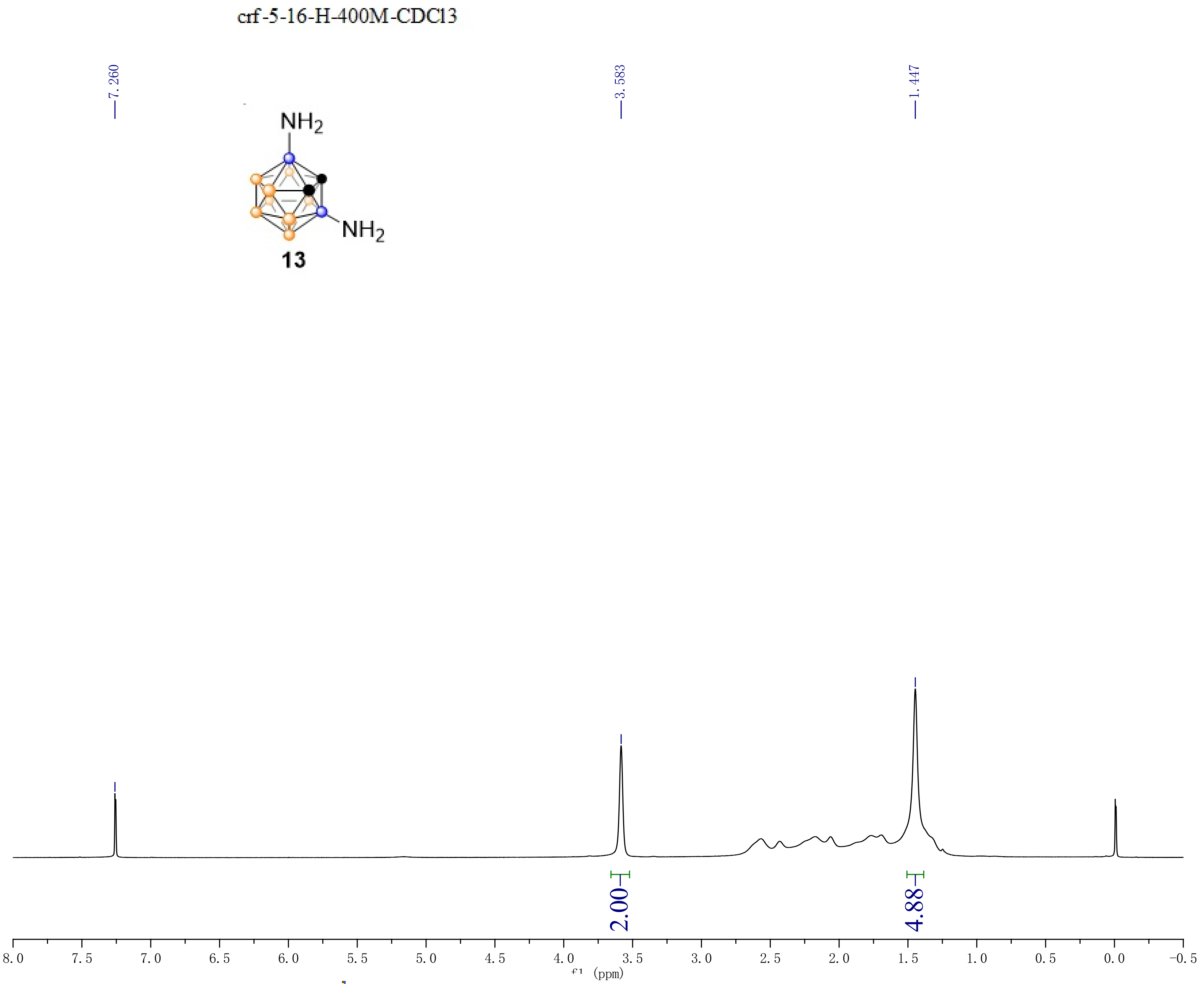

Parameter	Value
Title	crf-5-16-H-400M
Comment	
Origin	Varian
Owner	
Site	
Spectrometer	vnmrs
Author	
Solvent	CDCl3
Temperature	19.0
Pulse Sequence	s2pul
Number of Scans	8
Receiver Gain	36
Relaxation Delay	1.0000
Pulse Width	0.0000
Acquisition Date	2015-06-27T06:18:35
Spectrometer Frequency	399.72
Spectral Width	6410.3
Lowest Frequency	-808.0
Nucleus	1H
Acquired Size	19231
Spectral Size	65536

Supplementary Figure 163. ^{1}H NMR Spectrum of **13**.

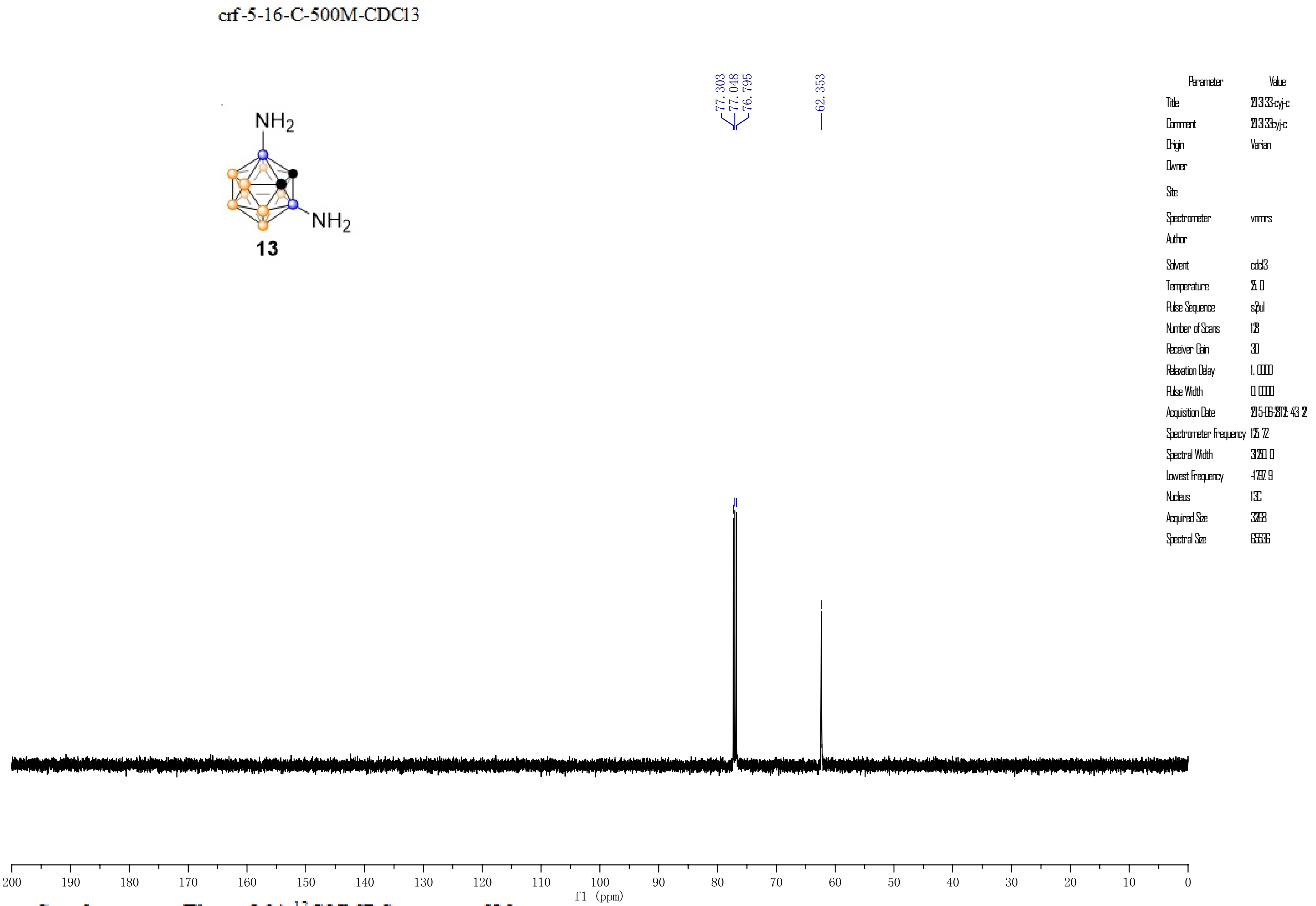

Supplementary Figure 164. ^{13}C NMR Spectrum of **13**.

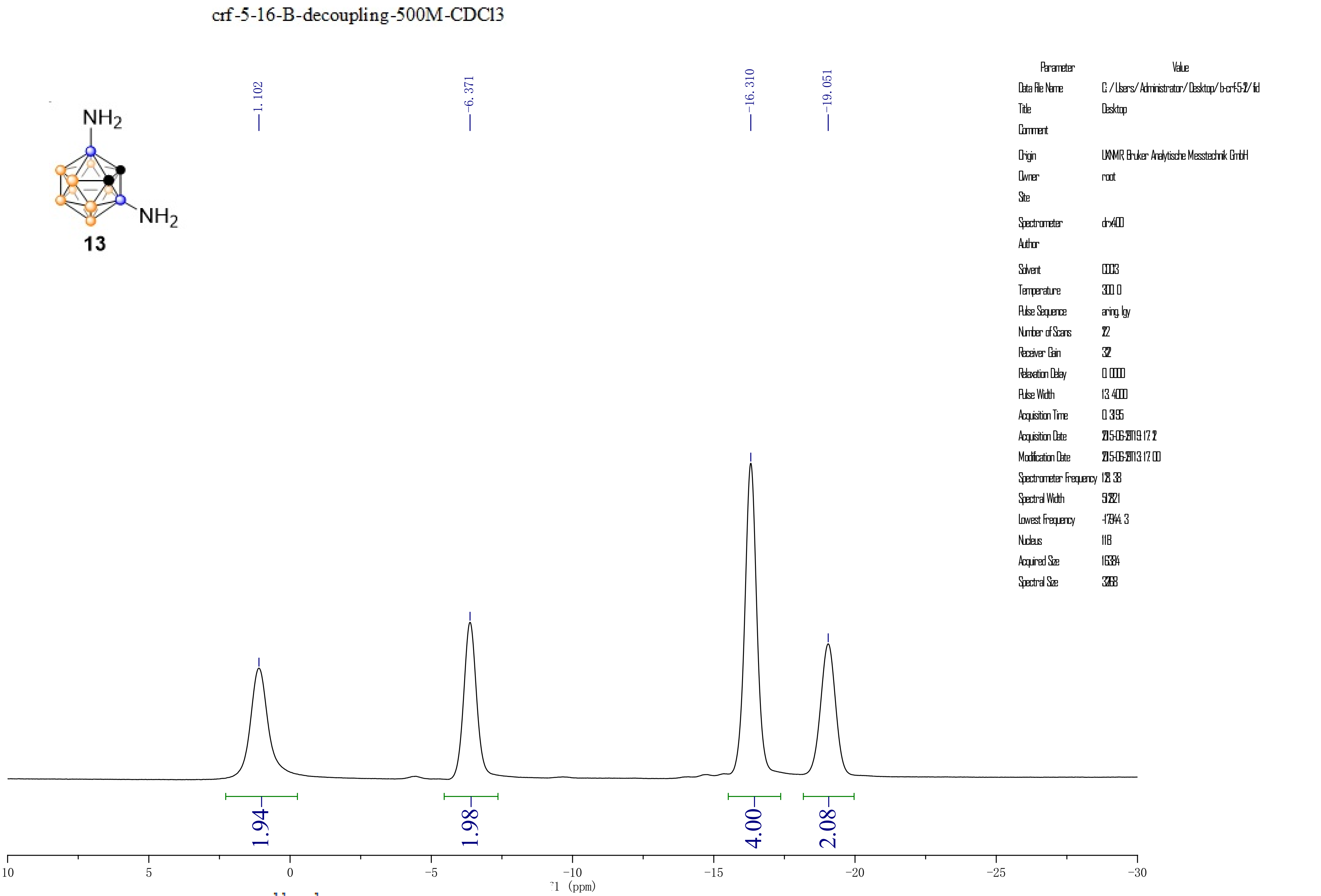

Supplementary Figure 165. $^{11}B\{^1H\}$ NMR Spectrum of **13**.

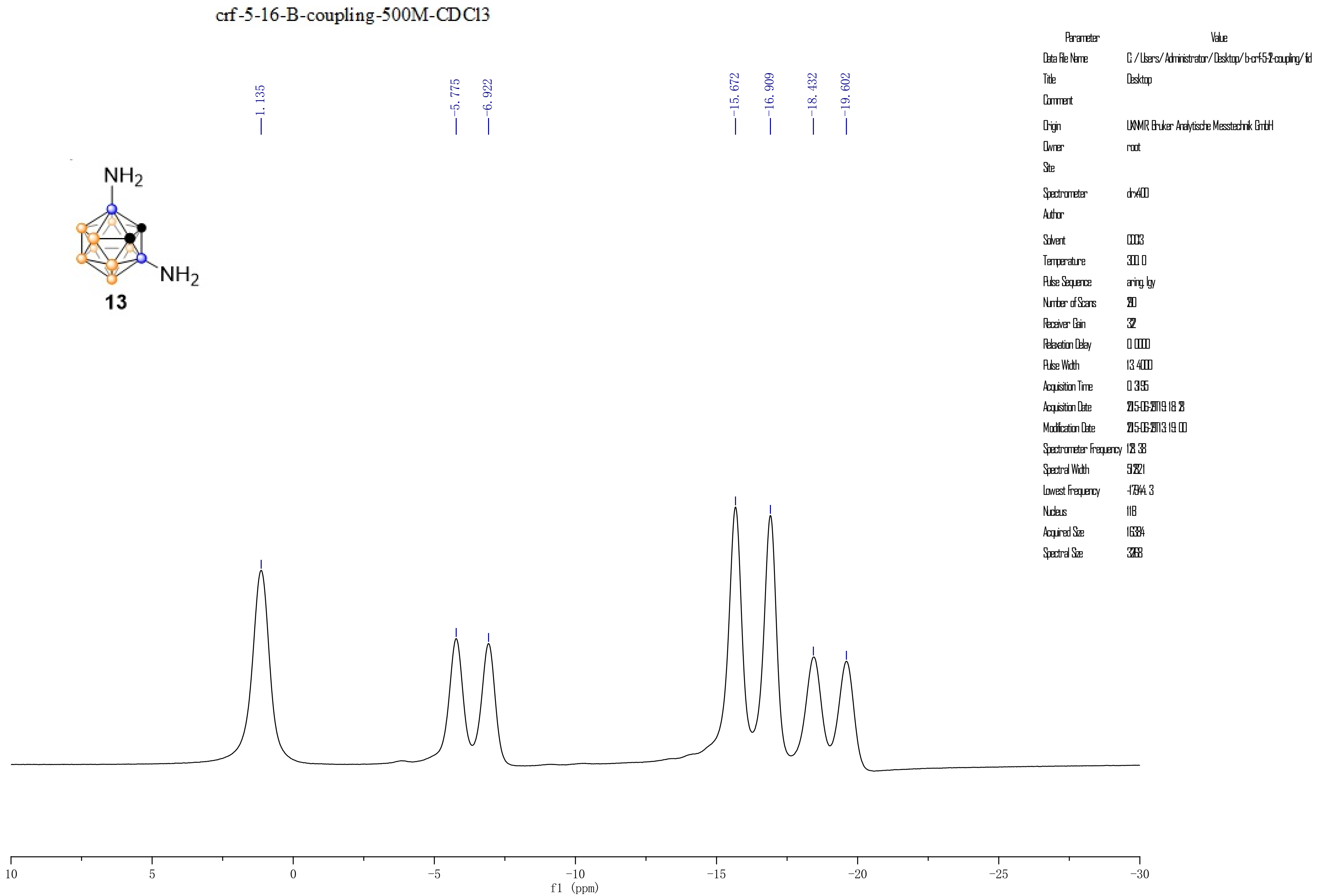

Supplementary Figure166. ^{11}**B NMR Spectrum of 13.**

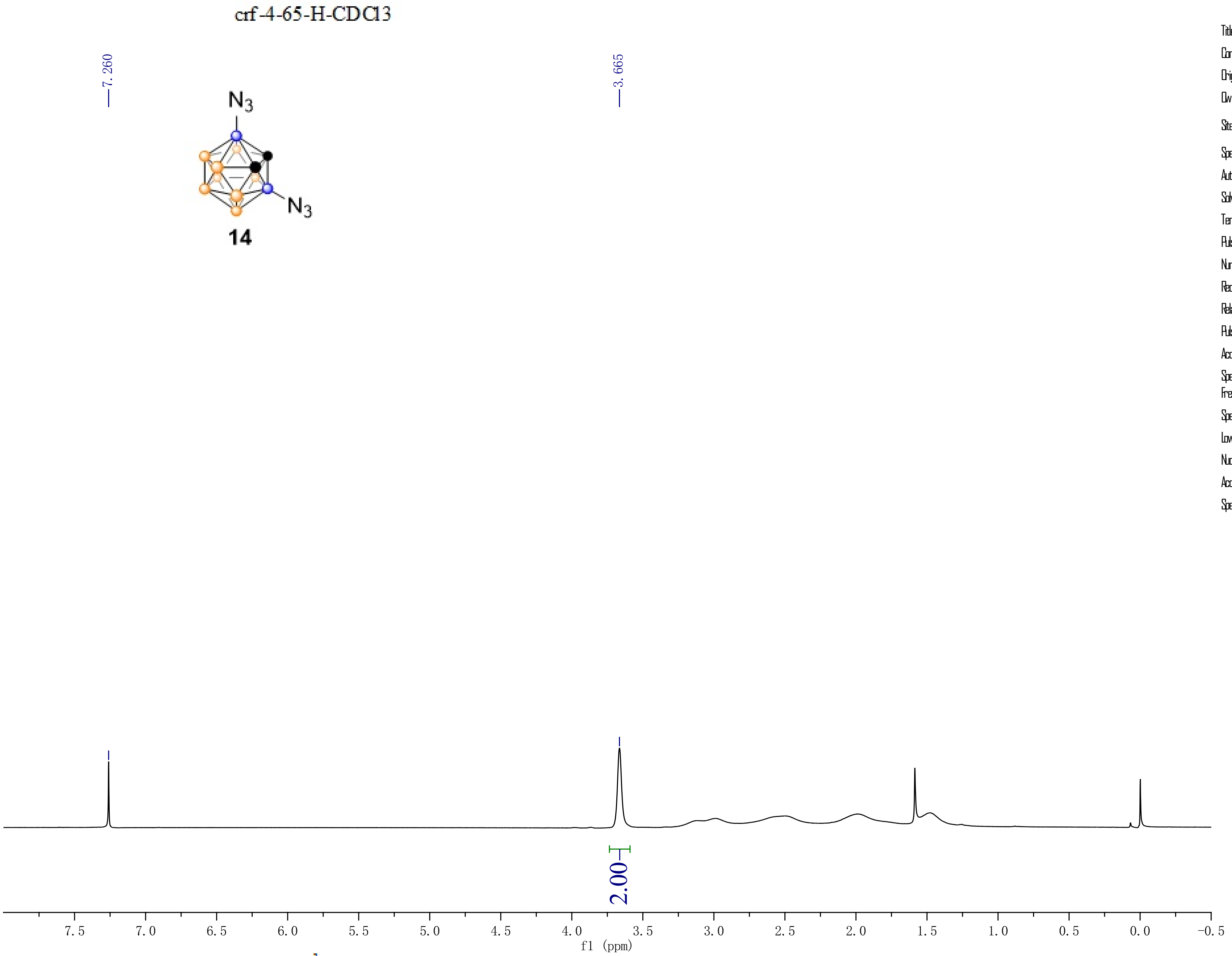

14

Parameter	Value
Title	crf-4-65-H-0615
Comment	STANDARD 1H OBSERVE
Origin	Varian
Owner	
Site	
Spectrometer	mercury
Author	omc
Solvent	CDCl3
Temperature	21.0
Pulse Sequence	s2pul
Number of Scans	8
Receiver Gain	28
Relaxation Delay	1.0000
Pulse Width	0.0000
Acquisition Date	2015-06-15T16:37:00
Spectrometer Frequency	300.03
Spectral Width	5494.5
Lowest Frequency	-711.9
Nucleus	1H
Acquired Size	10975
Spectral Size	32768

Supplementary Figure 167. ^{1}H NMR Spectrum of **14**.

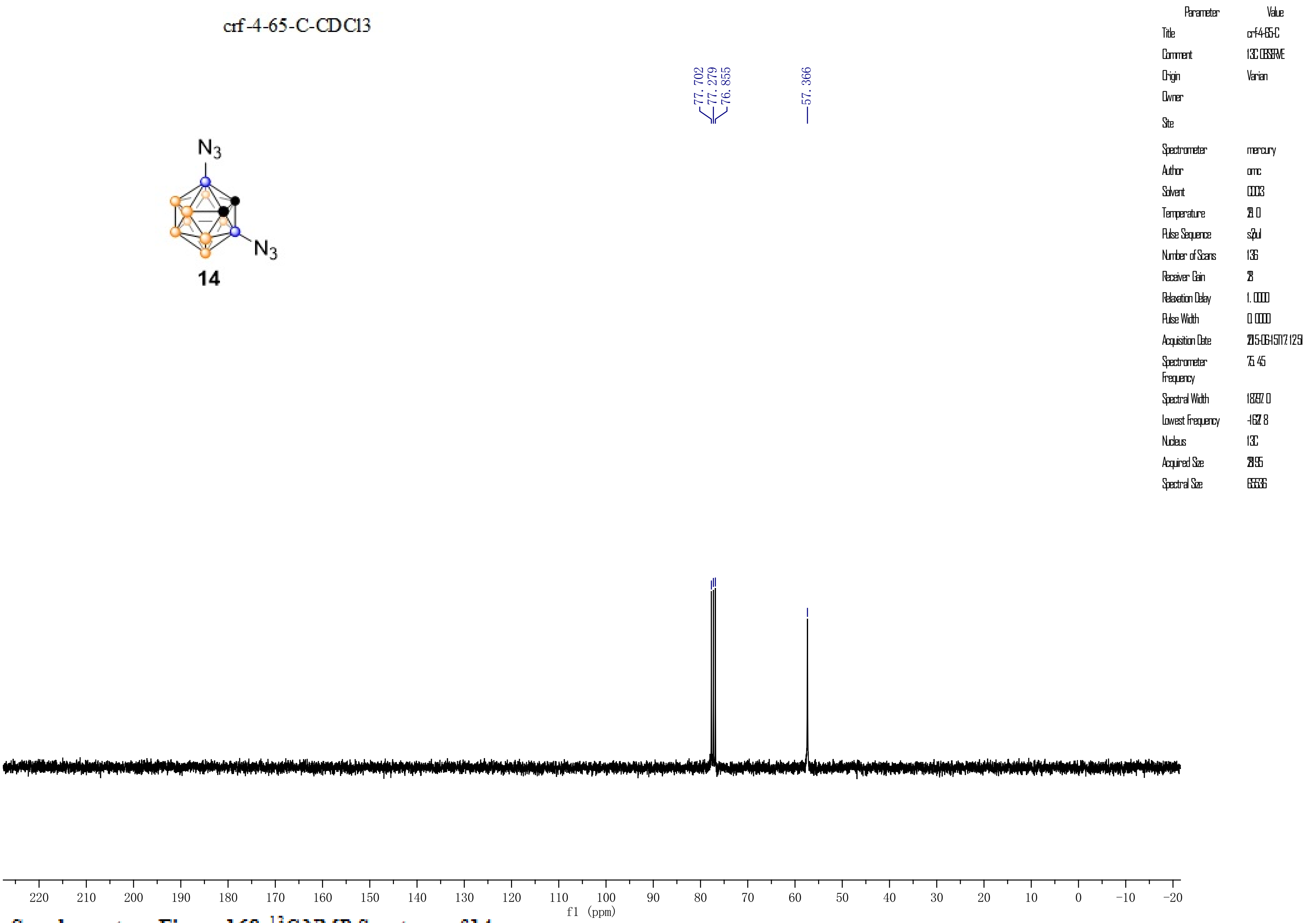

Supplementary Figure 168. ^{13}C NMR Spectrum of **14**.

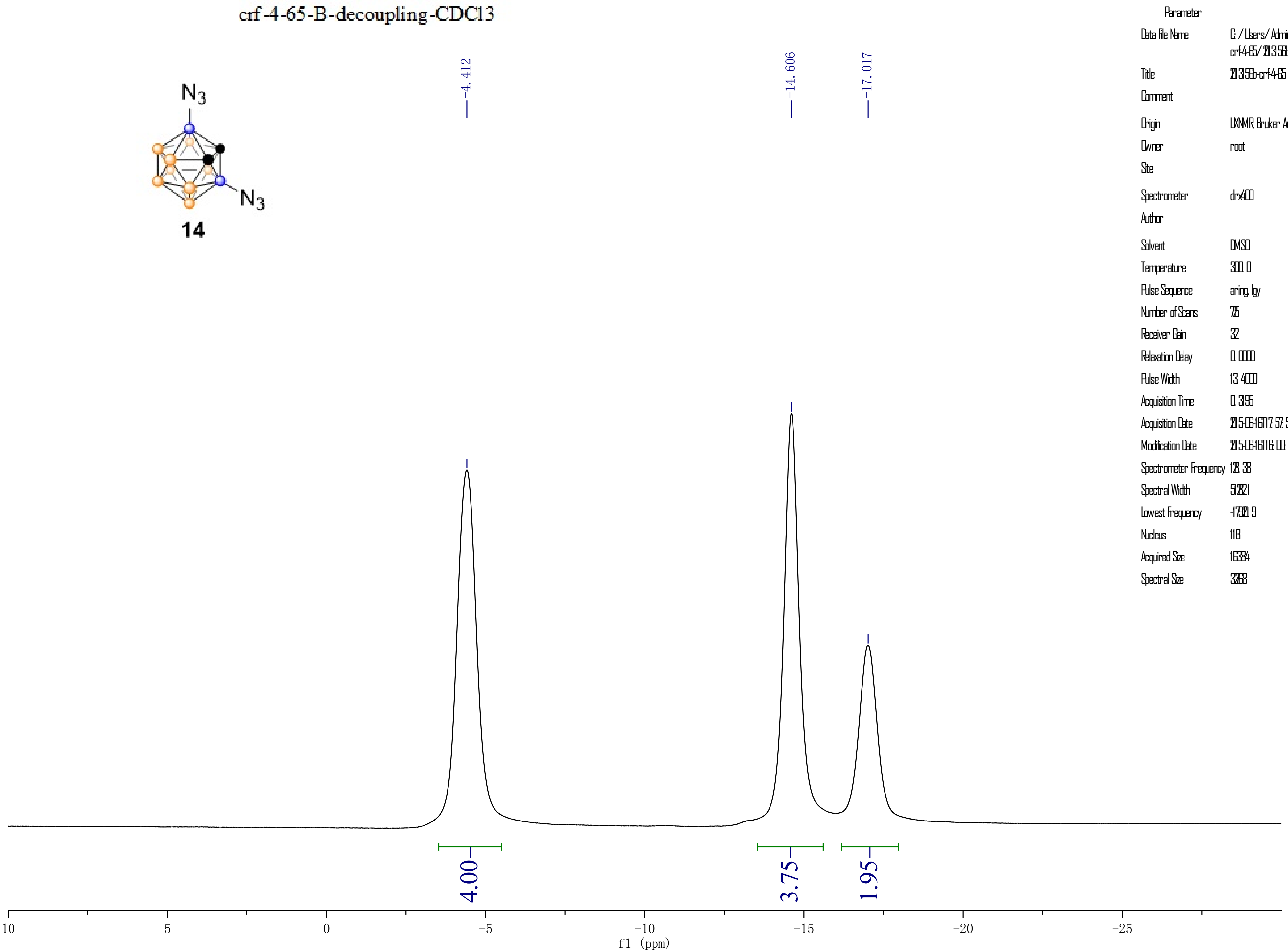

Parameter	Value
Data File Name	C:/Users/Administrator/Desktop/2013156b-crf4-65/2013156b-crf4-65/1/fid
Title	2013156b-crf4-65
Comment	
Origin	UXNMR, Bruker Analytische Messtechnik GmbH
Owner	root
Site	
Spectrometer	drx400
Author	
Solvent	DMSO
Temperature	300.0
Pulse Sequence	aring.lgy
Number of Scans	725
Receiver Gain	32
Relaxation Delay	0.0000
Pulse Width	13.4000
Acquisition Time	0.3195
Acquisition Date	2015-06-16T17:57:50
Modification Date	2015-06-16T16:00:25
Spectrometer Frequency	128.38
Spectral Width	51282.1
Lowest Frequency	-17920.9
Nucleus	11B
Acquired Size	16384
Spectral Size	32768

Supplementary Figure 169. $^{11}B\{^1H\}$ NMR Spectrum of **14**.

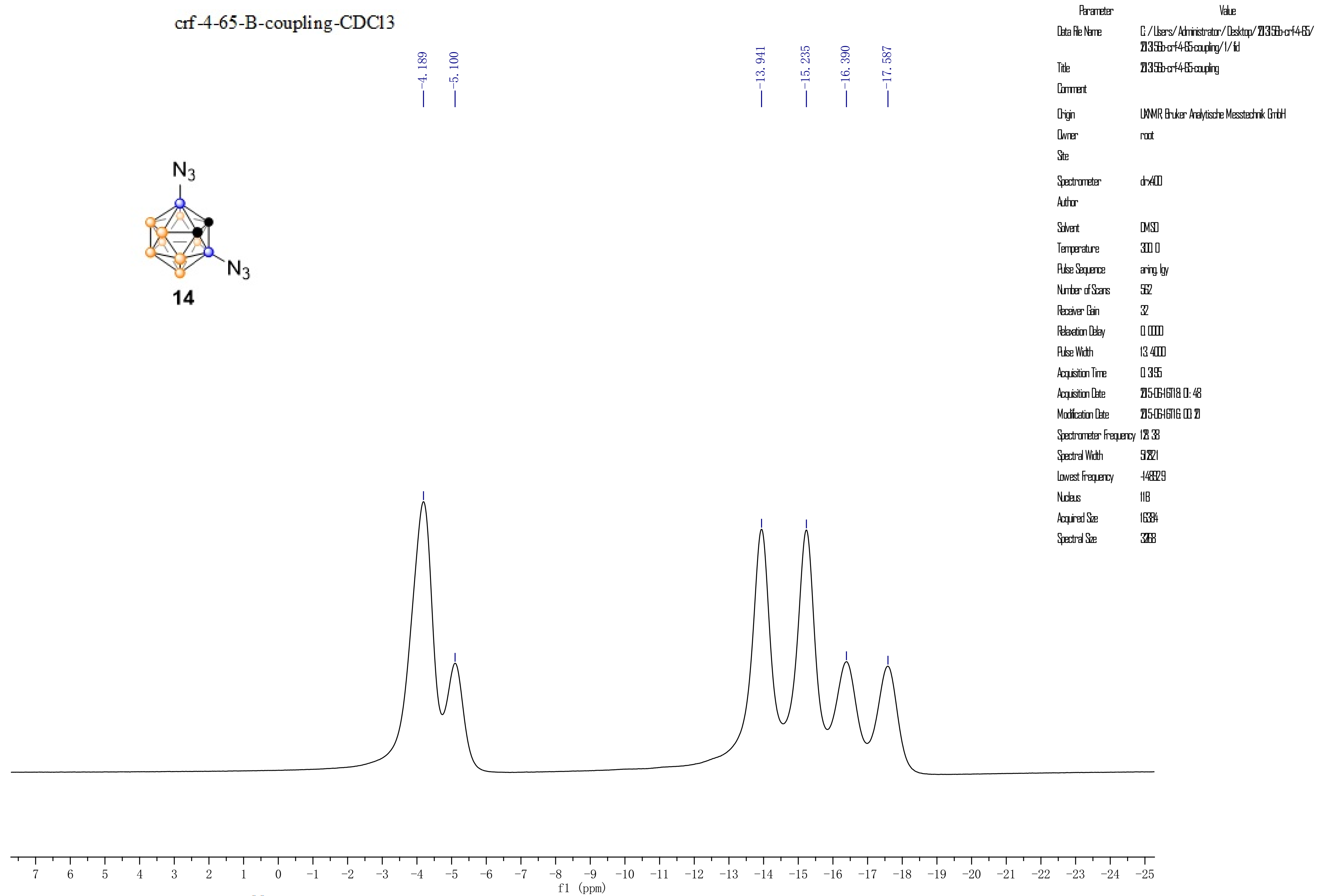

Supplementary Figure 170. ^{11}B NMR Spectrum of 14.

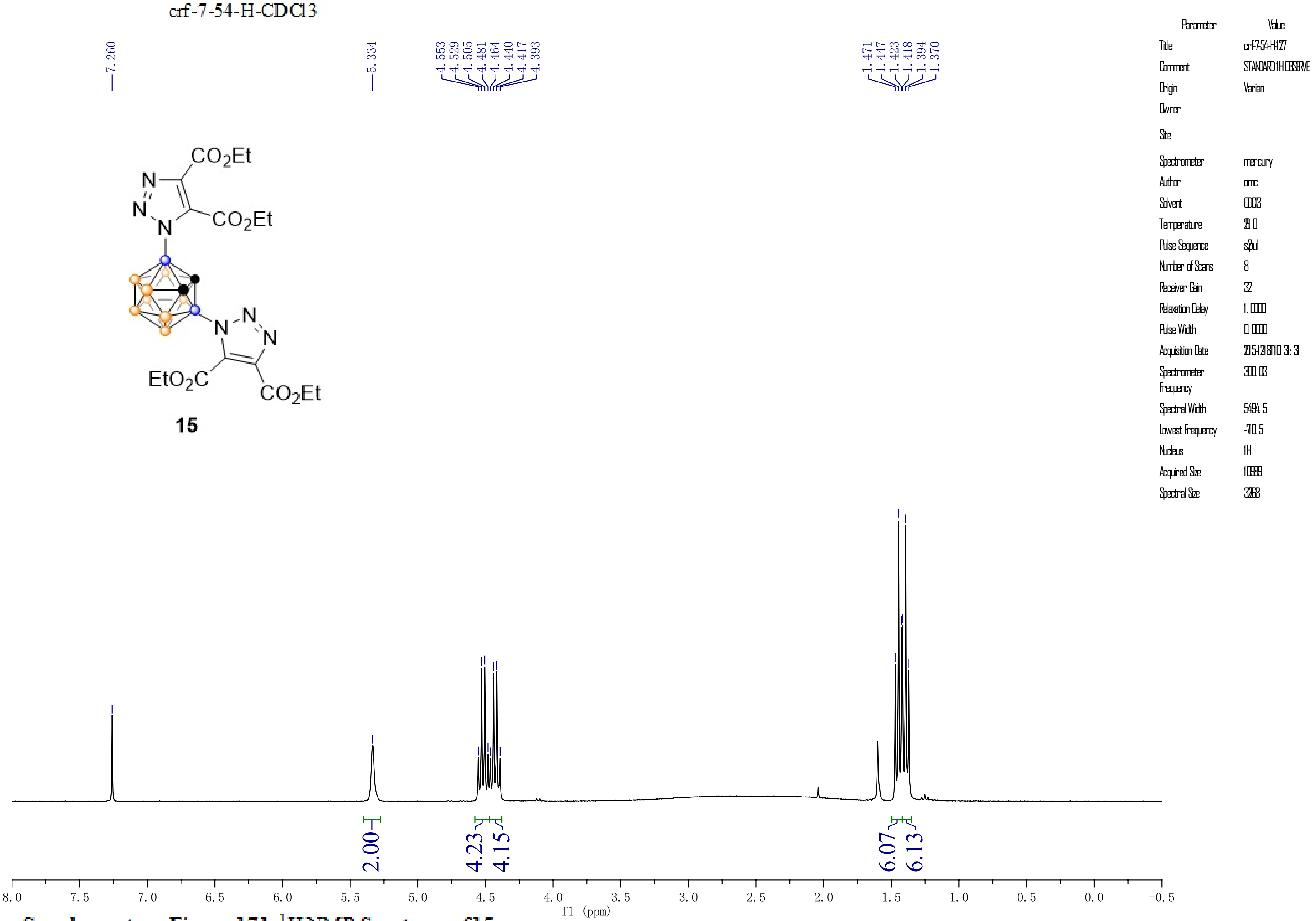

Supplementary Figure 171. ^{1}H NMR Spectrum of **15**.

crf-7-54-C-CDCl3

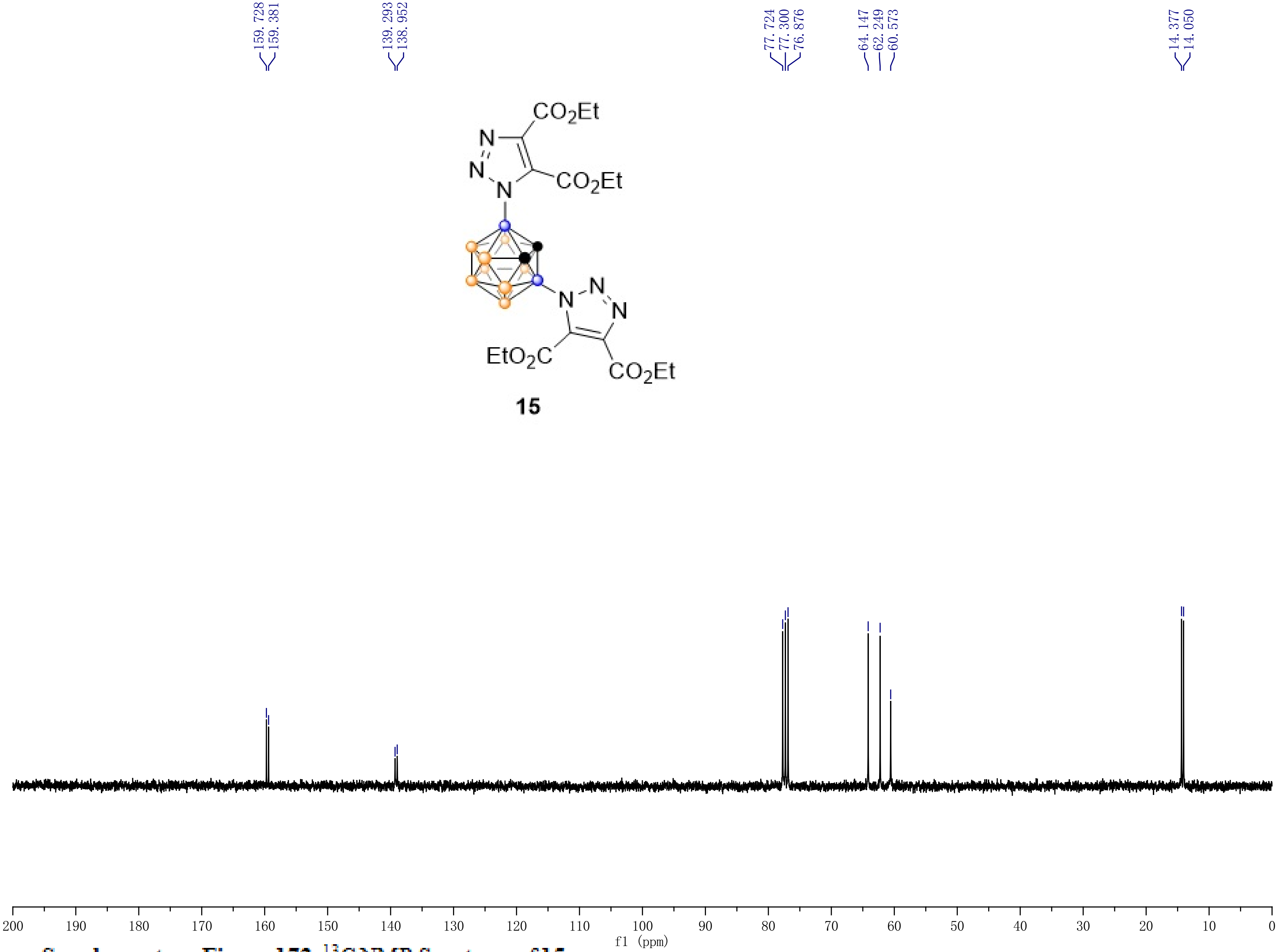

Parameter	Value
Title	crf-7-54-C
Comment	13C OBSERVE
Origin	Varian
Owner	
Site	
Spectrometer	mercury
Author	omc
Solvent	CDCl3
Temperature	21.0
Pulse Sequence	s2pul
Number of Scans	200
Receiver Gain	38
Relaxation Delay	1.0000
Pulse Width	0.0000
Acquisition Date	2015-12-18T11:13:31
Spectrometer Frequency	75.45
Spectral Width	22491.8
Lowest Frequency	-2815.2
Nucleus	13C
Acquired Size	30738
Spectral Size	65536

Supplementary Figure 172. ^{13}C NMR Spectrum of **15**.

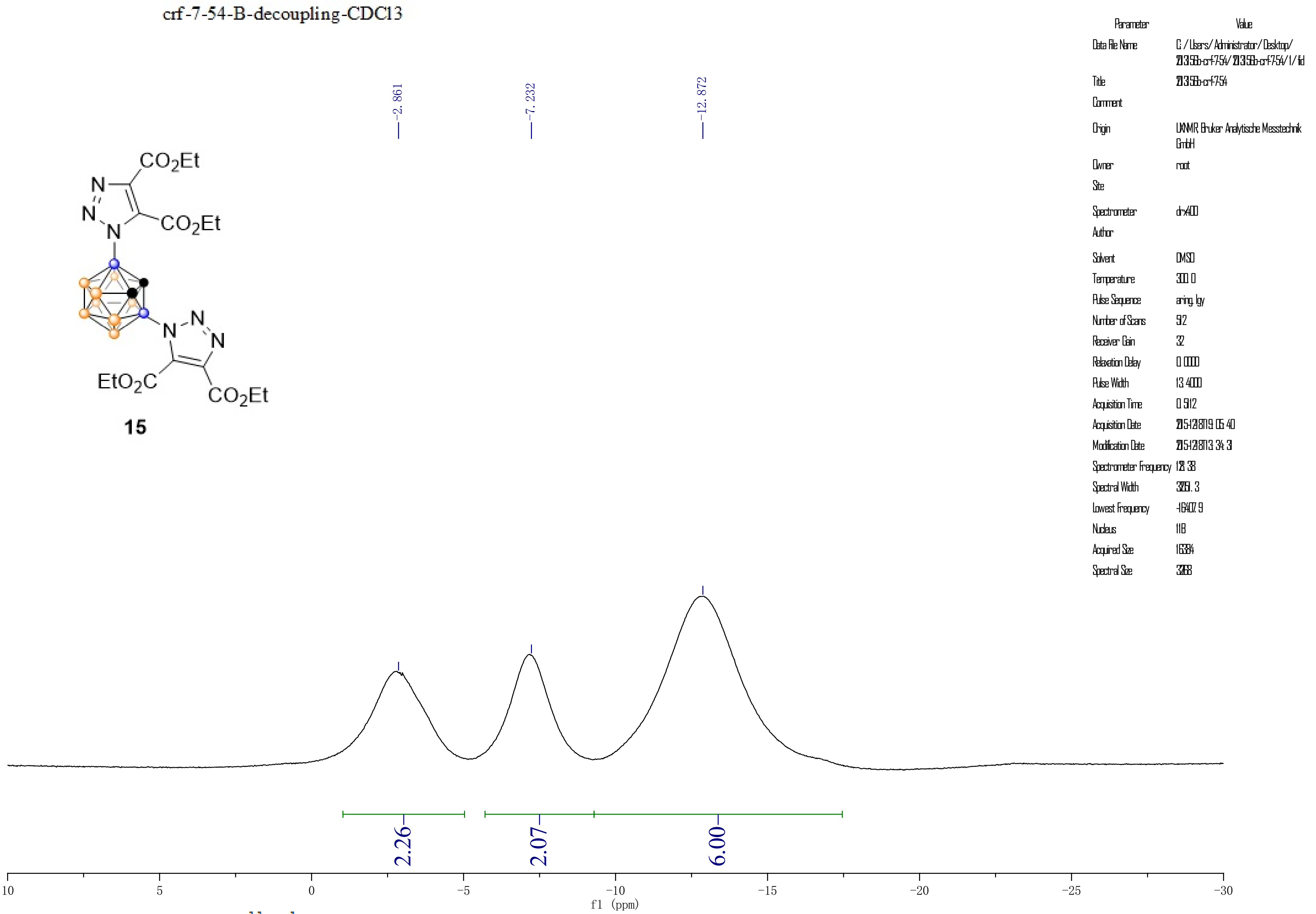

Supplementary Figure 173. $^{11}B\{^1H\}$ NMR Spectrum of **15**.

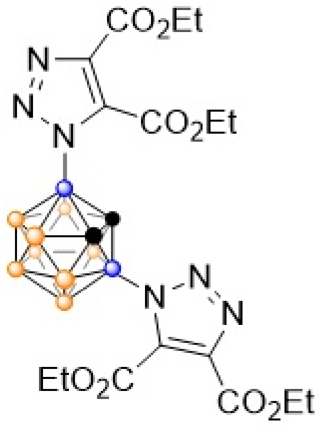

15

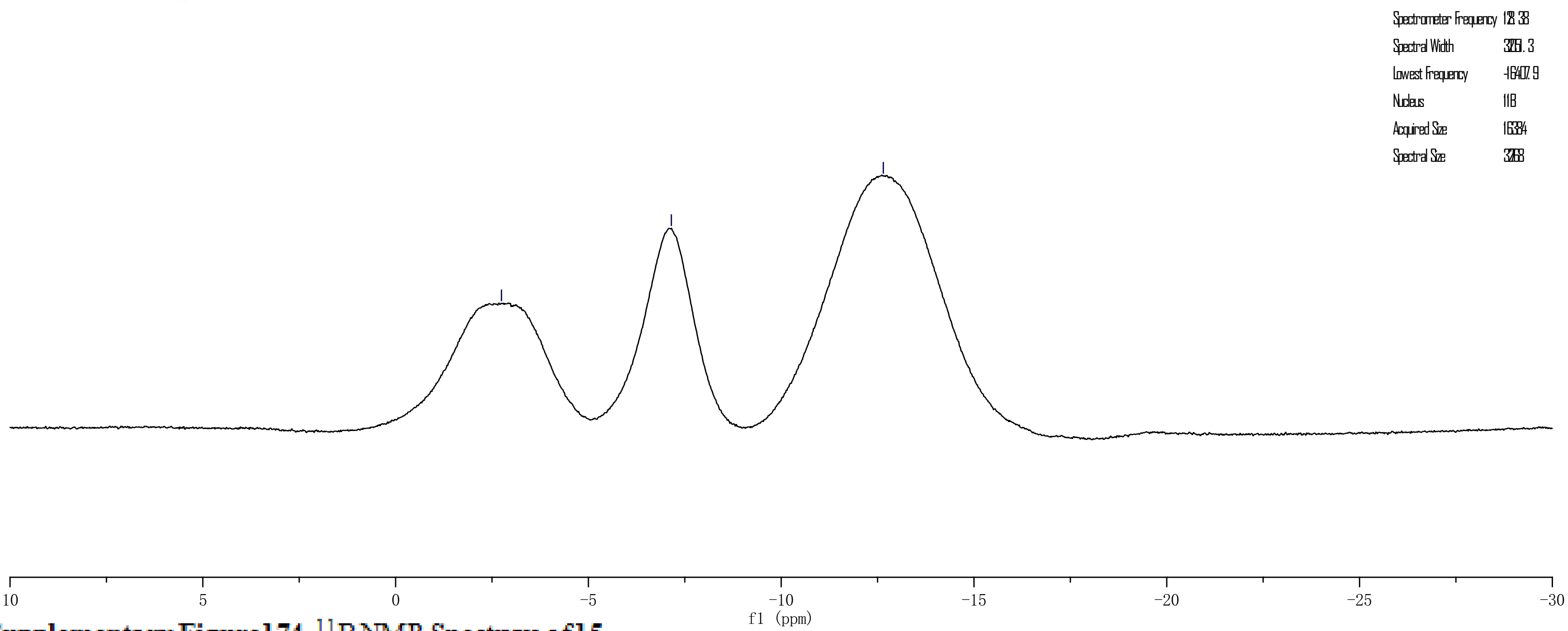

Parameter	Value
Data File Name	C:/Users/Administrator/Desktop/20131556b-crf-7-54/20131556b-crf-7-54-coupling/1/fid
Title	20131556b-crf-7-54-coupling
Comment	
Origin	UXNMR, Bruker Analytische Messtechnik GmbH
Owner	root
Site	
Spectrometer	drx400
Author	
Solvent	DMSO
Temperature	300.0
Pulse Sequence	aring.lgy
Number of Scans	512
Receiver Gain	32
Relaxation Delay	0.0000
Pulse Width	13.4000
Acquisition Time	0.512
Acquisition Date	2015-12-28T19:12:15
Modification Date	2015-12-28T13:34:27
Spectrometer Frequency	128.38
Spectral Width	32051.3
Lowest Frequency	-16407.9
Nucleus	11B
Acquired Size	16384
Spectral Size	32768

Supplementary Figure174. ^{11}B NMR Spectrum of **15**.

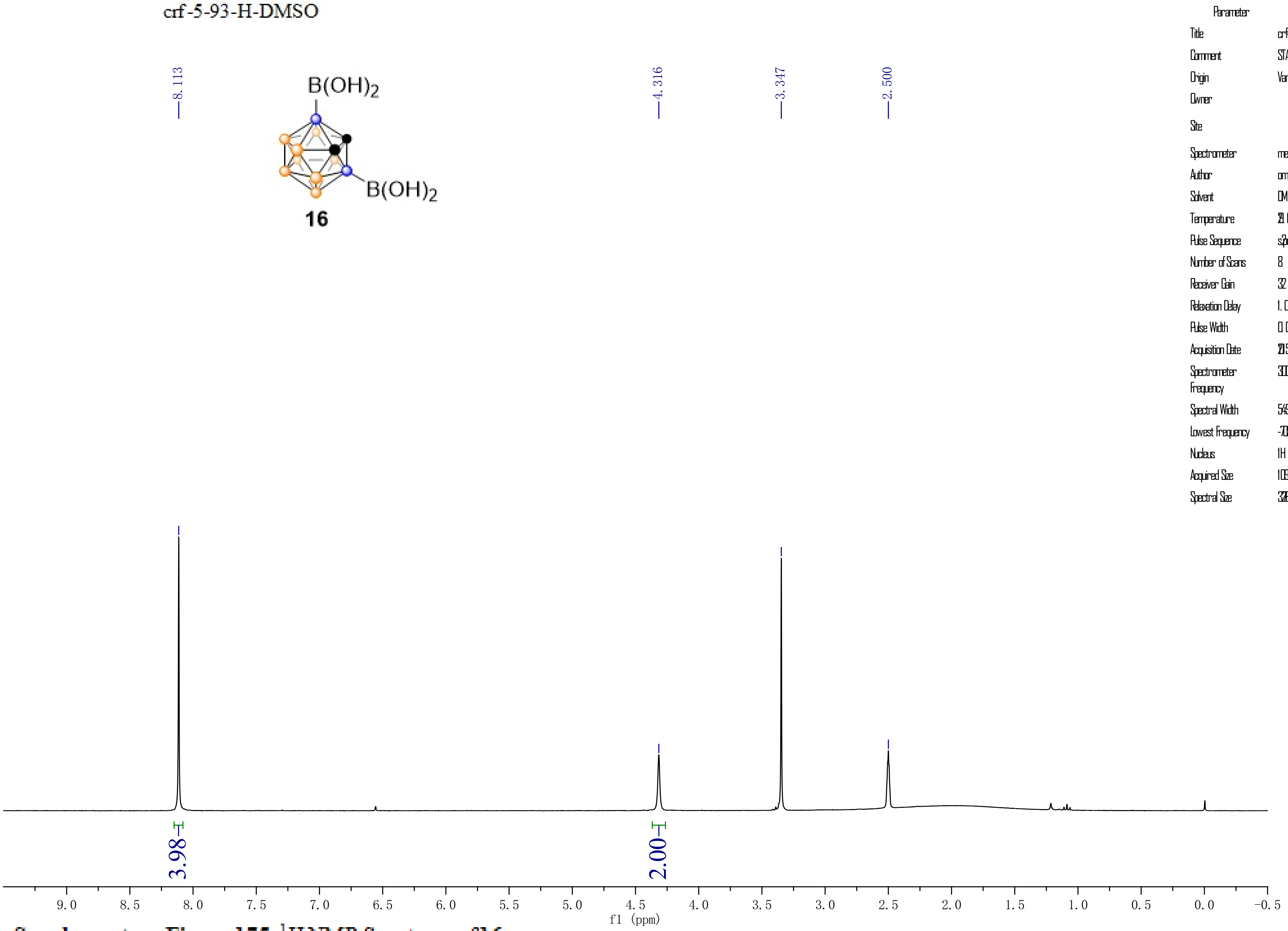

Parameter	Value
Title	crf-5-93-112-H
Comment	STANDARD 1H OBSERVE
Origin	Varian
Owner	
Site	
Spectrometer	mercury
Author	omc
Solvent	DMSO
Temperature	21.0
Pulse Sequence	s2pul
Number of Scans	8
Receiver Gain	32
Relaxation Delay	1.0000
Pulse Width	0.0000
Acquisition Date	2015-11-27T21:50:46
Spectrometer Frequency	300.03
Spectral Width	5494.5
Lowest Frequency	-705.0
Nucleus	1H
Acquired Size	10989
Spectral Size	32768

Supplementary Figure 175. ^{1}H NMR Spectrum of **16**.

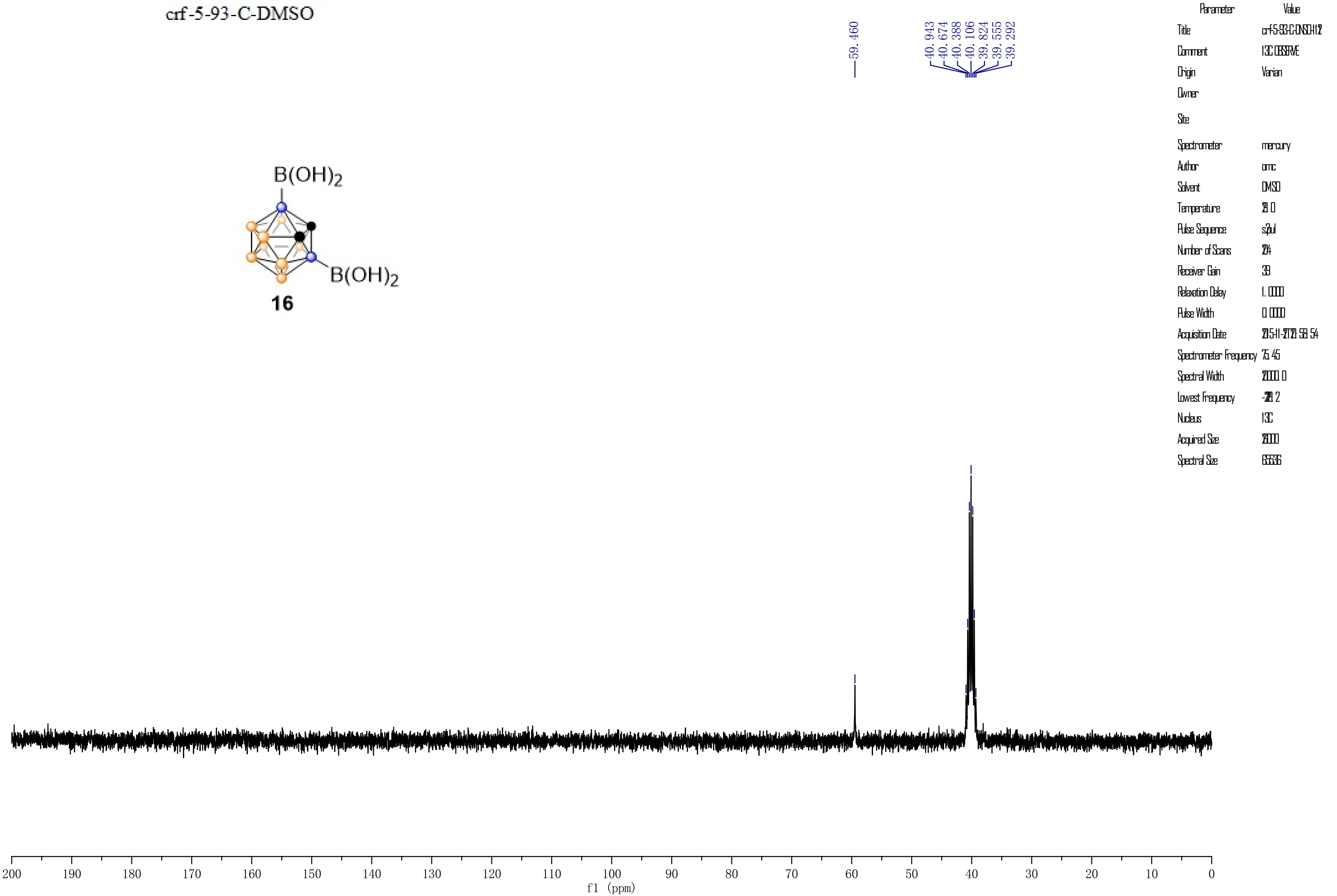

Supplementary Figure 176. ^{13}C NMR Spectrum of **16**.

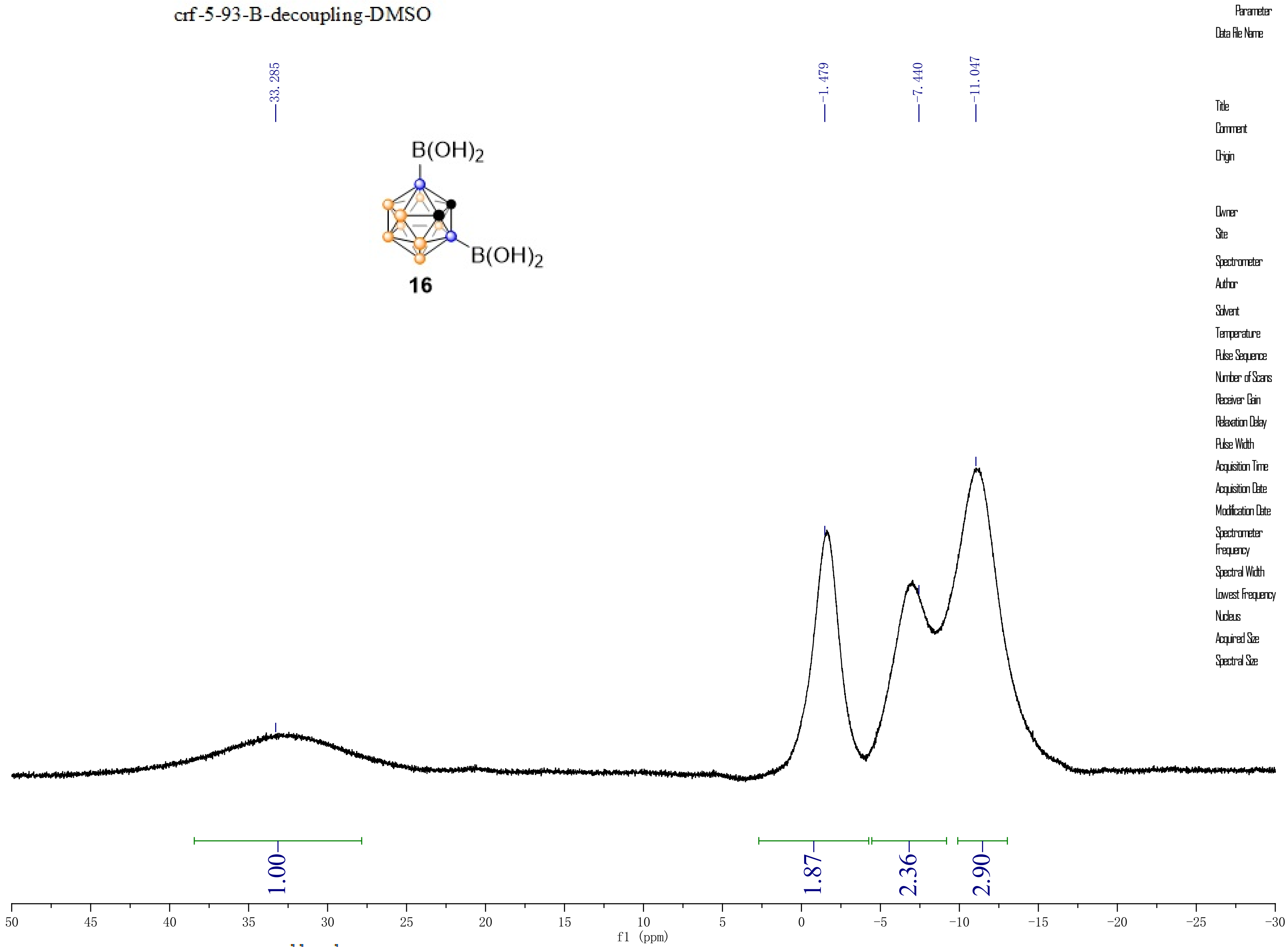

Parameter	Value
Data File Name	C:/Users/Administrator/Desktop/[illegible]-crf-7-2/[illegible]-crf-5-93/1/fid
Title	[illegible]-crf-5-93
Comment	
Origin	UXNMR, Bruker Analytische Messtechnik GmbH
Owner	root
Site	
Spectrometer	drx400
Author	
Solvent	DMSO
Temperature	300.0
Pulse Sequence	aring.lgy
Number of Scans	256
Receiver Gain	32
Relaxation Delay	0.0000
Pulse Width	13.4000
Acquisition Time	0.5112
Acquisition Date	2015-12-04T[illegible]
Modification Date	2015-12-04T14:33:[illegible]
Spectrometer Frequency	128.38
Spectral Width	32051.3
Lowest Frequency	-16631.3
Nucleus	11B
Acquired Size	16384
Spectral Size	32768

Supplementary Figure 177. $^{11}B\{^1H\}$ NMR Spectrum of **16**.

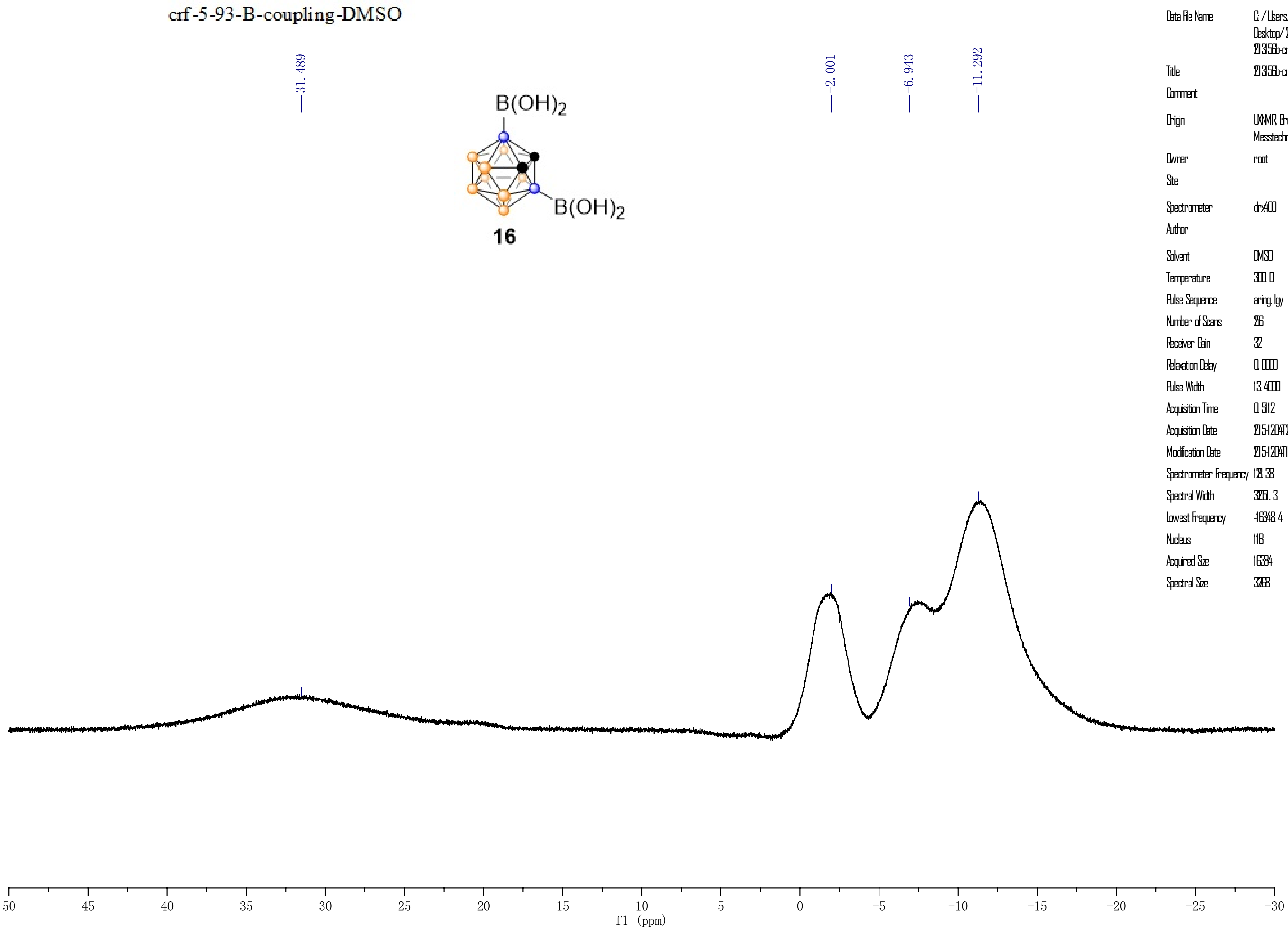

16

Parameter	Value
Data File Name	C:/Users/Administrator/Desktop/20101156-crf-7-12/20131566-crf-5-93-coupling/1/fid
Title	20131566-crf-5-93-coupling
Comment	
Origin	UXNMR, Bruker Analytische Messtechnik GmbH
Owner	root
Site	
Spectrometer	drx400
Author	
Solvent	DMSO
Temperature	300.0
Pulse Sequence	aring.lgy
Number of Scans	256
Receiver Gain	32
Relaxation Delay	0.0000
Pulse Width	13.4000
Acquisition Time	0.5112
Acquisition Date	2015-12-04T21:26:32
Modification Date	2015-12-04T14:33:15
Spectrometer Frequency	128.38
Spectral Width	32051.3
Lowest Frequency	-16348.4
Nucleus	11B
Acquired Size	16384
Spectral Size	32768

Supplementary Figure 178. ^{11}B NMR Spectrum of **16**.
